# Genomes of multicellular algal sisters to land plants illuminate signaling network evolution

**DOI:** 10.1038/s41588-024-01737-3

**Published:** 2024-05-01

**Authors:** Xuehuan Feng, Jinfang Zheng, Iker Irisarri, Huihui Yu, Bo Zheng, Zahin Ali, Sophie de Vries, Jean Keller, Janine M. R. Fürst-Jansen, Armin Dadras, Jaccoline M. S. Zegers, Tim P. Rieseberg, Amra Dhabalia Ashok, Tatyana Darienko, Maaike J. Bierenbroodspot, Lydia Gramzow, Romy Petroll, Fabian B. Haas, Noe Fernandez-Pozo, Orestis Nousias, Tang Li, Elisabeth Fitzek, W. Scott Grayburn, Nina Rittmeier, Charlotte Permann, Florian Rümpler, John M. Archibald, Günter Theißen, Jeffrey P. Mower, Maike Lorenz, Henrik Buschmann, Klaus von Schwartzenberg, Lori Boston, Richard D. Hayes, Chris Daum, Kerrie Barry, Igor V. Grigoriev, Xiyin Wang, Fay-Wei Li, Stefan A. Rensing, Julius Ben Ari, Noa Keren, Assaf Mosquna, Andreas Holzinger, Pierre-Marc Delaux, Chi Zhang, Jinling Huang, Marek Mutwil, Jan de Vries, Yanbin Yin

**Affiliations:** 1 Nebraska Food for Health Center, Department of Food Science and Technology, University of Nebraska-Lincoln, Lincoln, NE USA; 2 Institute of Microbiology and Genetics, Department of Applied Bioinformatics, University of Goettingen, Goettingen, Germany; 3 Campus Institute Data Science, University of Goettingen, Goettingen, Germany; 4 Section Phylogenomics, Centre for Molecular biodiversity Research, Leibniz Institute for the Analysis of Biodiversity Change, Zoological Museum Hamburg, Hamburg, Germany; 5 University of Nebraska-Lincoln, Center for Plant Science Innovation, Lincoln, NE USA; 6 Nanyang Technological University, School of Biological Sciences, Singapore, Singapore; 7 Laboratoire de Recherche en Sciences Végétales, Université de Toulouse, CNRS, UPS, INP Toulouse, Castanet-Tolosan, France; 8 University of Jena, Matthias Schleiden Institute/Genetics, Jena, Germany; 9 Plant Cell Biology, Department of Biology, University of Marburg, Marburg, Germany; 10 Department of Algal Development and Evolution, Max Planck Institute for Biology Tübingen, Tübingen, Germany; 11 Institute for Mediterranean and Subtropical Horticulture ‘La Mayora’, Málaga, Spain; 12 Computational Biology, Department of Biology, Center for Biotechnology, Bielefeld University, Bielefeld, Germany; 13 Northern Illinois University, Molecular Core Lab, Department of Biological Sciences, DeKalb, IL USA; 14 University of Innsbruck, Department of Botany, Research Group Plant Cell Biology, Innsbruck, Austria; 15 Department of Biochemistry and Molecular Biology, Dalhousie University, Halifax, Nova Scotia Canada; 16 University of Goettingen, Albrecht-von-Haller-Institute for Plant Sciences, Experimental Phycology and Culture Collection of Algae at Goettingen University, Goettingen, Germany; 17 University of Applied Sciences Mittweida, Faculty of Applied Computer Sciences and Biosciences, Section Biotechnology and Chemistry, Molecular Biotechnology, Mittweida, Germany; 18 Universität Hamburg, Institute of Plant Science and Microbiology, Microalgae and Zygnematophyceae Collection Hamburg and Aquatic Ecophysiology and Phycology, Hamburg, Germany; 19 Genome Sequencing Center, HudsonAlpha Institute for Biotechnology, Huntsville, AL USA; 20 Department of Energy Joint Genome Institute, Lawrence Berkeley National Laboratory, Berkeley, CA USA; 21 Environmental Genomics and Systems Biology Division, Lawrence Berkeley National Laboratory, Berkeley, CA USA; 22 Department of Plant and Microbial Biology, University of California Berkeley, Berkeley, CA USA; 23 North China University of Science and Technology, Tangshan, China; 24 Boyce Thompson Institute, Ithaca, NY USA; 25 Plant Biology Section, Cornell University, Ithaca, NY USA; 26 University of Freiburg, Centre for Biological Signalling Studies (BIOSS), Freiburg, Germany; 27 The Hebrew University of Jerusalem, The Robert H. Smith Institute of Plant Sciences and Genetics in Agriculture, Rehovot, Israel; 28 University of Nebraska-Lincoln, School of Biological Sciences, Lincoln, NE USA; 29 Department of Biology, East Carolina University, Greenville, NC USA; 30 State Key Laboratory of Crop Stress Adaptation and Improvement, School of Life Sciences, Henan University, Kaifeng, China; 31 University of Goettingen, Goettingen Center for Molecular Biosciences, Goettingen, Germany; 32 Present Address: Zhejiang Lab, Hangzhou, China; 33 Present Address: Germplasm Bank of Wild Species, Kunming Institute of Botany, Chinese Academy of Science, Yunnan, China

**Keywords:** Genomics, Plant molecular biology, Gene expression

## Abstract

Zygnematophyceae are the algal sisters of land plants. Here we sequenced four genomes of filamentous Zygnematophyceae, including chromosome-scale assemblies for three strains of *Zygnema circumcarinatum*. We inferred traits in the ancestor of Zygnematophyceae and land plants that might have ushered in the conquest of land by plants: expanded genes for signaling cascades, environmental response, and multicellular growth. Zygnematophyceae and land plants share all the major enzymes for cell wall synthesis and remodifications, and gene gains shaped this toolkit. Co-expression network analyses uncover gene cohorts that unite environmental signaling with multicellular developmental programs. Our data shed light on a molecular chassis that balances environmental response and growth modulation across more than 600 million years of streptophyte evolution.

## Abstract (web summary)

Genome assemblies of four filamentous Zygnematophyceae and co-expression network analyses shed light on the evolutionary roots of the mechanism for balancing environmental responses and multicellular growth.

## Main

Plant terrestrialization changed the surface of the Earth. The first land plants (Embryophyta) emerged from within the clade of Streptophyta about 550 million years ago^1^. Among six classes of streptophyte algae, the closest relatives of land plants are the Zygnematophyceae^2–4^, algae with more than 4,000 described species^5^ arranged into five orders^6^. So far, genome sequences are available only for unicellular Zygnematophyceae^7–9^.

Zygnematophyceae possess adaptations to withstand terrestrial stressors, such as desiccation, ultraviolet light, freezing and other abiotic stresses^10^. The nature of these stress responses is of deep biological importance: various orthologous groups of proteins once considered specific to land plants have recently been inferred to predate the origin of Embryophyta^11,12^. The accuracy of inferring the developmental and physiological programs of the first land plant ancestors depends on our ability to predict them in its sister group.

In this Article, we report on the first four genomes of filamentous Zygnematophyceae, including the first chromosome-scale assemblies for any streptophyte algae. By using comparative genomics, we pinpoint genetic innovations of the earliest land plants. Our network analyses reveal co-expression of genes that were expanded and gained in the last common ancestor (LCA) of land plants and Zygnematophyceae. We shed light on the deep evolutionary roots of the mechanism for balancing environmental responses and multicellular growth.

## Results

### First chromosome-level genomes for streptophyte algae

**Fig. 1:**
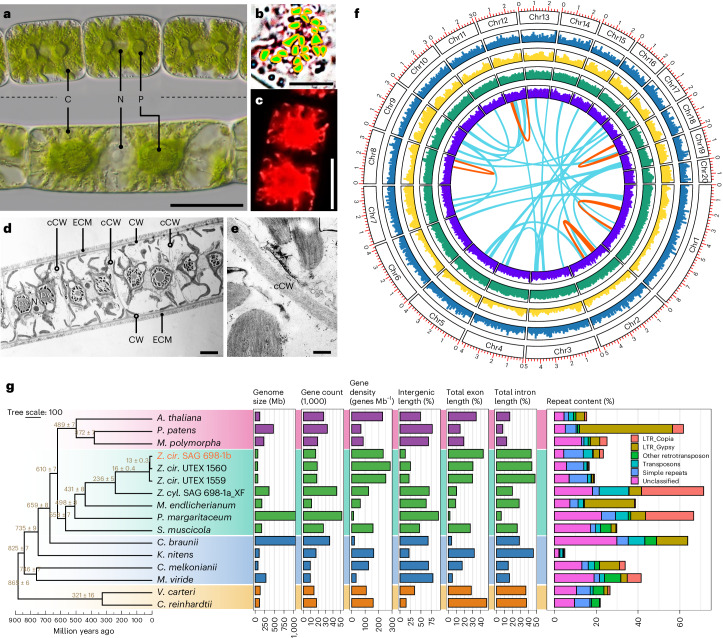
*Zygnema.* **a**, Three cells of a vegetative filament of SAG 698-1b (top) compared with one cell of a vegetative filament of SAG 698-1a (bottom, both samples of 1 month old cultures). Scale bar, 20 μm. C, chloroplast; N, nucleus; P, pyrenoid. One-cell filament contains two chloroplasts and one nucleus. **b**, Chromosome counting on light micrographs of SAG 698-1b fixed and stained with acetocarmine at prophase (0.5 months old); count was also performed in metaphase and telophase (Supplementary Fig. 1). The green dots represent the 20 chromosomes that were counted after rendering a stack of ~100 images. Scale bar, 10 μm. See Supplementary Fig. 1 for the original images. A minimum of ten cells each from three independent cell cultures were analyzed. **c**, A confocal laser scanning image of one SAG 698-1b cell (0.5 months). Scale bar, 20 μm. **d**, Transmission electron micrographs illustrating the filamentous nature of *Z. circumcarinatum* (SAG 698-1b). Left: overview showing that the cells are connected by extremely thin cross cell walls (cCW), while the outer cell wall (CW) is surrounded by a pectinous extracellular matrix (ECM); within the individual cells, pyrenoids (Py) and the nucleus (N) are clearly depictable. Scale bar, 5 µm. **e**, A detailed view of the cross wall separating two cells where chloroplast lobes are visible. Scale bar, 0.5 µm. Transmission electron micrographs (**d** and **e**) derived from the analysis of ≥15 algal filaments each for two independent cell cultures. **f**, Chromosome-level assembly of the SAG 698-1b genome. Concentric rings show chromosome (Chr) numbers, gene density (blue), repeat density (yellow), RNA-seq mapping density log_10_(fragments per kilobase of transcript per million mapped reads) (dark green) and guanine-cytosine content density (violet). The red and green links show respectively intra- and interchromosomal syntenic blocks. **g**, A comparison of genome properties for 13 algal and 3 land plant species. The time-calibrated species tree was built from 493 low-copy genes (all nodes supported by >97% nonparametric bootstrap; numbers at branches are estimated divergence times in million years (mean ± standard deviation) (see Supplementary Table 1f for details). Data for the bar plot can be found in Supplementary Table 1i,j. Source data

The nuclear and organellar genomes of four *Zygnema* strains (*Zygnema circumcarinatum* SAG 698-1b, UTEX 1559 and UTEX 1560 and *Z.* cf. *cylindricum* SAG 698-1a_XF; Fig. 1a–c) were assembled (Supplementary Table 1a–d). *Zygnema* cells are arranged in multicellular filaments containing two chloroplasts per cell (Fig. 1a,c,d) and much thinner (~400 nm) cross walls than outer walls (~1 µm; Fig. 1d,e and Supplementary Fig. 1), reflecting on their true filamentous body plan. Using chromatin conformation data (Dovetail Hi-C), we scaffolded the *Z. circumcarinatum* SAG 698-1b genome (N50, 4 Mb; Table 1 and Supplementary Table 1c) into 20 pseudo-chromosomes (Fig. 1f), which were supported by cytological chromosome counting^13^ (Fig. 1b and Supplementary Fig. 1). The total assembly size (71 Mb) was close to sizes estimated by flow cytometry, fluorescence staining^14^ and *k*-mer frequency analysis (Supplementary Fig. 2 and Supplementary Table 1b). The high mapping rates of UTEX 1559 and UTEX 1560 Illumina reads to the SAG 698-1b genome (97.16% and 97.12%, respectively) show that the overall genome structure was stable in the separate strain copies (Supplementary Text 1). UTEX 1559 and UTEX 1560 assemblies also have 20 pseudo-chromosomes. The three new *Z*. *circumcarinatum* genomes represent the first chromosome-level assemblies for any streptophyte alga (Table 1).

**Table 1:** Genome assembly statistics for the new *Zygnema* genomes and available streptophyte algae (see Supplementary Table 1b–e for further details)

Species (strain)	Assembly size (Mb)	BUSCO (%)	N50 (kb)	Number of scaffolds (pseudochromosomes)	RNA-seq mapping rate (%)
*Z. circumcarinatum* SAG 698-1b	71.0	89.8	3,958.3	90 (20)	97.2
*Z. circumcarinatum* UTEX 1559	71.3	88.2	3,970.3	614 (20)	98.3
*Z. circumcarinatum* UTEX 1560	67.3	87.9	3,792.7	514 (20)	95.9**
*Z.* cf. *cylindricum* SAG 698-1a_XF	359.8	70.6	213.9	3,587	88.3
*Mesotaenium endlicherianum* SAG 12.97	163	78.1	448.4	13,861	94.4
*Penium margaritaceum* SAG 2640	3,661	49.8	116.2	332,786	96.8
*Spirogloea muscicola* CCAC 0214	174	84.7	566.4	17,449	95.2
*Chara braunii* S276	1,430	78.0	2,300	11,654	89.5
*Klebsormidium nitens* NIES-2285	104	94.9	134.9	1,814	98.1
*Chlorokybus melkonianii* CCAC 0220	74	93.3	752.4	3,809	96.5
*Mesostigma viride* CCAC 1140	281	59.2	113.2	6,924	84.3

**The mapping rate of *Z. circumcarinatum* UTEX 1560 was calculated by using SAG 698-1b RNA-seq reads mapped to the genome of UTEX 1560.

The nuclear genome assembly of SAG 698-1a_XF is five times larger (360 Mb) than those of *Z. circumcarinatum* (Table 1 and Fig. 1g). The marked genome size differences further support the notion that SAG 698-1a_XF and SAG 698-1b are two different species (Table 1 and Fig. 1a). Following a recent study^14^, we refer to SAG 698-1a_XF as *Z.* cf. *cylindricum* (Fig. 1g, Table 1, Supplementary Table 1e,f and Supplementary Figs. 3–5).

### The smallest sequenced streptophyte genome

The three *Z. circumcarinatum* genomes reported here are the smallest among all streptophyte algae sequenced thus far (Table 1, Fig. 1g and Supplementary Table 1i). The genome of SAG 698-1b contains 23.4% repeats, while *Z.* cf. *cylindricum* SAG 698-1a_XF contains 73.3% (Supplementary Table 1j). No evidence for whole genome duplication (WGD) was found in *Zygnema* (Supplementary Fig. 6); *Z.* cf. *cylindricum* is probably polyploid (Supplementary Fig. 2).

Our phylogenetic analyses show that SAG 698-1b and UTEX 1560 are closer to each other than to UTEX 1559 (Fig. 1g and Supplementary Fig. 7). Gauch^15^ reported that UTEX 1559 was a nonfunctional mating type (+) whereas UTEX 1560 and SAG 698-1b were functional mating type (−); indeed, our conjugation experiments failed to conjugate UTEX 1559 with UTEX 1560 or SAG 698-1b. Whole genome alignments (Supplementary Fig. 8) found chromosomes 20, 13 and 16 to differ the most among the three genomes, suggesting that they might contain sex/mating determination loci. *Zygnema* mating loci are so far unknown, and we did not identify homologs of the sex hormone proteins (protoplast release-inducing protein (PR-IP) and its inducer) described in *Closterium* (Supplementary Table 1k). The recently identified^8^ *Cp*Minus1, an RWP-RK domain-containing protein that determines the mating type in heterothallic *Closterium*, does have homologs in *Zygnema*, but they are considerably longer (172 amino acids in *Cp*Minus1 versus 641 in Zci_02303 and 785 in Zci_08682). A total of 17,644 genes were shared by all three *Z. circumcarinatum* genomes (Supplementary Fig. 8f).

### Enriched orthogroups and domains in Zygnematophyceae

**Fig. 2:**
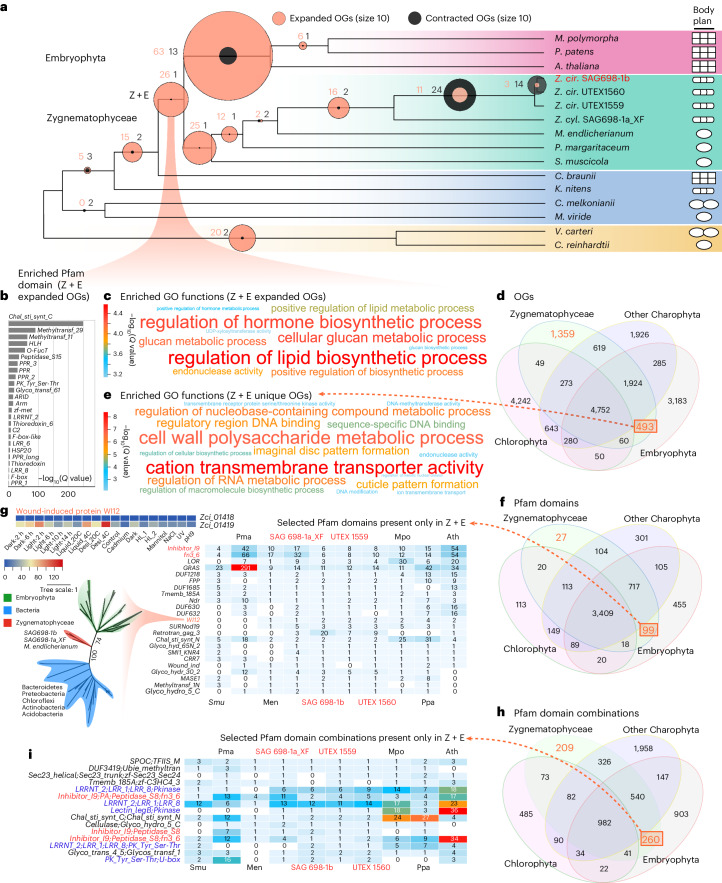
Comparative genomics of algal and land plant genomes. **a**, Gene family expansion and contraction patterns estimated by CAFE using Orthofinder-identified OGs and the time-calibrated phylogeny of Fig. 1g. Key nodes are indicated on the tree and significant expansions and contractions are shown. The circles are proportional to expanded/contracted OGs; the numbers next to the circles indicate the numbers of expanded (orange) and contracted (dark gray) OGs. *Z. cir*., *Zygnema circumcarinatum*; *Z. cyl*., *Zygnema* cf. *cylindricum*. Icons indicate body plans: parenchymatous (box of tissue), filamentous (chain of cells), unicellular (single round cell) and sarcinoid/colonial (two round cells). **b**, Pfam domain enrichment for genes on the node leading to Zygnematophyceae and Embryophyta (Z + E). **c**, Functional (GO) enrichment for the Z + E node. **d**, OGs overlap among Chlorophyta, Embryophyta, Zygnematophyceae and other streptophyte algae. **e**, Enriched GO terms in the 493 OGs exclusive to Zygnematophyceae and Embryophyta. **f**, Pfam domain overlap among Chlorophyta, Embryophyta, Zygnematophyceae and other streptophyte algae. **g**, Exclusive Pfam domains found only in Zygnematophyceae and Embryophyta. One Pfam family WI12 was studied with phylogenetic analysis, suggesting a possible HGT from bacteria and expression response to stresses. **h**, Pfam domain combination overlap among Chlorophyta, Embryophyta, Zygnematophyceae and other streptophyte algae. **i**, Exclusive Pfam domain combinations in Zygnematophyceae and Embryophyta. Smu, *Spirogloea muscicola*; Pma, *Penium margaritaceum*; Men, *Mesotaenium endlicherianum*; SAG 698-1a_XF, SAG 698-1b, UTEX 1559 and UTEX 1560, the four here sequenced *Zygnema* spp.; Mpo, *Marchantia polymorpha*; Ppa, *Physcomitrium patens*; Ath, *Arabidopsis thaliana*. Source data

For the LCA of Zygnematophyceae + Embryophyta (Z + E) we infer an overrepresentation of Pfam domains (Fig. 2a,b), including (1) Chal_sti_synt_C (found in the key enzyme of the flavonoid pathway chalcone synthase), (2) Methyltransf_29 (found in *Arabidopsis* AT1G19430, required for cell adhesion^16^), (3) pentatricopeptide repeat (PPR) domains involved in organellar RNA binding and editing, and (4) domains related to plant immunity such as leucine-rich repeat (LRR) and Peptidase_S15, PK_Tyr_Ser-Thr and thioredoxins^17^; some overrepresentations could be gains via horizontal gene transfer (HGT), including Chal_sti_synt_C^18^ and O-FucT (Supplementary Fig. 9). The Z + E LCA had enriched Gene Ontology (GO) terms related to biosynthesis of phytohormones, lipids and glucan (Fig. 2c), with 493 orthogroups (OGs) exclusive to Z + E (Fig. 2d), enriched in ‘cation transmembrane transporter’ and ‘cell wall polysaccharide metabolic’ (Fig. 2e).

A total of 3,409 Pfam domains were present in at least one representative of Cholorophyta, Embryophyta, Zygnematophyceae and other streptophyte algae; 99 were exclusive to Z + E, and 27 to Zygnematophyceae (Fig. 2f). Some domains exclusive to Z + E could be the result of HGT (Supplementary Table 2). For example, Inhibitor_I9 and fn3_6 domains are among the most abundant in Z + E (Fig. 2g) and often co-exist with Peptidase_S8 domain in plant subtilases (SBTs; Fig. 2i), reportedly acquired from bacteria^19,20^; the same goes for the WI12 domain, named after the cell wall protein WI12 induced by diverse stressors^21^ and key for pathogen defense^22^ (Fig. 2g).

Combining existing protein domains is a powerful mechanism for functional innovation, as shown for cell adhesion, cell communication and differentiation^23^ (Fig. 2h). A total of 982 Pfam domain combinations are shared by all studied genomes; 260 are unique to Z + E and 209 to Zygnematophyceae. Among those exclusive to Z + E (Fig. 2i), we found Lectin_legB and Pkinase domains (Supplementary Table 2) that were only combined into the same protein in the Z + E ancestor (despite individually having older evolutionary origins): for example, Zci_10218 (an L-type lectin receptor-like kinase (LecRLKs) family protein), featuring an extracellular Lectin_legB domain, an intracellular Pkinase domain and a middle transmembrane domain^24^.

### Increased sophistication and resilience via expansions

We inferred 26 significantly expanded OGs in the LCA of Z + E (Fig. 2a and Supplementary Table 3a,b), three of which are related to phytohormone signaling^9,10,25^. Several expansions suggest more sophisticated gene networks featuring cornerstones in plant stress response and environmental signaling^26,27^, and transmembrane transporters, including those involved in biotic interactions.

The LCA of Zygnematophyceae displayed 25 significantly expanded OGs (Fig. 2a and Supplementary Table 3b). Most expanded are alpha-fucosyltransferases (OG 89) involved in xyloglucan fucosylation^28^. We found genes encoding ethylene sensors and histidine kinase-containing proteins (OG 94), bolstering the idea that two-component signaling is important and active in filamentous Zygnematophyceae^29,30^. Several OGs were associated with typical terrestrial stressors; for example, *Zygnema* has an expected^31^ set of phenylpropanoid enzyme-coding homologs (Supplementary Fig. 10). Several expanded OGs relate to development: expanded signaling and transport, possibly related to filamentous growth. A dynein-coding homolog (OG 72) was significantly contracted in Zygnematophyceae (Supplementary Table 3b), in line with the loss of motile gametes in Zygnematophyceae and OG 72 contraction in the Z + E ancestor.

*Zygnema*’s stress resilience is renowned; it thrives in extreme habitats such as the Arctic^32^. We recover 16 significantly expanded OGs for the LCA of all four *Zygnema* strains (Fig. 2a and Supplementary Table 3b), including PP2C-coding genes (OG 548) often involved in abiotic stress signaling; the expansions of PP2Cs are shared among *Zygnema* spp. but independent of the radiation of PP2CAs in land plants (Supplementary Fig. 10). Along these lines, expanded OGs further included photoprotective early light-inducible proteins (ELIPs; OG 97)—probably the result of gene duplications in *Zygnema* cf. *cylindricum* (35 homologs versus 5 in *Z. circumcarinatum* SAG 698-1b or 2 in *Arabidopsis* *thaliana*)—and low-CO_2_ inducible *LciC*s (OG 459). Like other Zygnematophyceae^33,34^, *Zygnema* has neochromes (Supplementary Fig. 11). Two OGs were significantly contracted: genes for GTP binding elongation factor *Tu* family (OG 251) and seven transmembrane MLO family protein (OG 320). On balance, the evolution of gene families reflects *Zygnema*’s resilience in the face of challenging habitats.

The LCA of *Z. circumcarinatum* displays reduction of expanded OGs (Fig. 2a and Supplementary Table 3b) aligning with its genomic streamlining (see also Supplementary Figs. 12–16).

### Multicellularity and protein domain combinations

Our micrographs of *Zygnema* support previous descriptions^35^, showing cells of a filament separated by very thin cross walls (Fig. 1d,e) that develop after cell division by cleavage, centripetally from the outside. Cells are surrounded by a homogalacturonan-rich extracellular matrix^36^ (ECM; Fig. 1d), while *Zygnema* lack plasmodesmata, diverse cross cell walls have been described in Zygnematophyceae, including in filamentous Desmidiaceae^37^. *Zygnema* lacks rhizoids and rarely branches; short branching occurs in other Zygnematophyceae such as *Zygogonium*^38^. These observations indicate that true multicellularity occurs in Zygnematophyceae. Indeed, we infer for the LCA of Zygnematophyceae several expanded OGs related to development (Supplementary Table 3b). Expanded signaling and transport, which may relate to filamentous growth, include genes for calcium signaling (OG 56), zinc-induced facilitators (OG 258), cysteine-rich fibroblast growth factor receptors found in the Golgi apparatus (OG 518), and cation/H^+^ antiporters (OG 809) related to *At*NHX5/6 acting in pH and ion homeostasis in the endosome, key for membrane trafficking in the *trans*-Golgi network^39,40^, and development by influencing auxin gradients^41^.

**Fig. 3:**
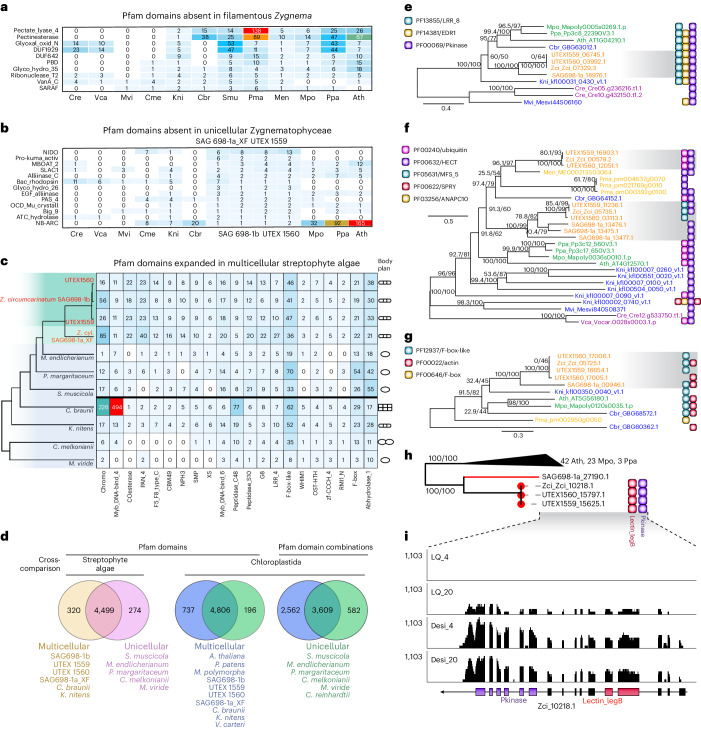
Protein domains in unicellular and multicellular species in the green lineage. **a**, Selected Pfam domains that are absent in the four filamentous *Zygnema* genomes. **b**, Selected Pfam domains that are absent in the three unicellular Zygnematophyceae genomes. **c**, A heatmap of selected Pfam domains that are significantly expanded in multicellular streptophyte algae; icons indicate body plans. **d**, Venn diagrams showing shared and exclusive Pfam domains and domain combinations in multicellular versus unicellular species. **e**–**g**, Phylogenetic trees of selected OGs and the corresponding protein domain architecture for each sequence. Phylogeny of Raf-related kinases bearing a combination of EDR1, LRR_8 and Pkinase domains (**e**). Phylogeny of HECT domain-containing ubiquitin protein ligases (**f**). Phylogeny of F-box-like domain-containing actin-related proteins (**g**). **h**, Phylogeny of Zci_10218.1, a gene encoding L-type LecRLK with Lectin_legB domain in the N-terminus, Pkinase in the C-terminus and a TM domain in the middle. **i**, RNA-seq read mapping of Zci_10218.1. LQ, liquid; Desi, dessication; 4 and 20, temperature in Celsius; 1,103, the highest read counts (*y* axis). Cre, *Chlamydomonas reinhardtii*; Vca, *Volvox carteri*; Mvi, *Mesostigma viride*; Cme, *Chlorokybus melkonianii*; Kni, *Klebsormidium nitens*; Cbr, *Chara braunii*; Smu, *Spirogloea muscicola*; Pma, *Penium margaritaceum*; Men, *Mesotaenium endlicherianum*; SAG 698-1a, SAG 698-1b, UTEX 1559 and UTEX 1560, the four here sequenced *Zygnema* spp.; Mpo, *Marchantia polymorpha*; Ppa, *Physcomitrium patens*; Ath, *Arabidopsis thaliana*. Source data

There have been multiple gains and losses of multicellularity in Zygnematophyceae^6^, but overall, it seems that gene gains are not the main drivers for multicellularity in filamentous *Zygnema* (Fig. 3a,b, Supplementary Table 4 and Supplementary Text 2). Significant domain expansions in multicellular streptophyte algae inlcude CHROMO (PF00385; particularly in *Chara braunii*), a domain integrating chromatin association with increased regulatory complexity^42^ (Fig. 3c), F-box (PF00646), F-box like (PF12937), Myb_DNA-bind_4 (PF13837), Myb_DNA-bind_6 (PF13921), COesterase (PF00135) and LRR_4 (PF12799; Fig. 3c). Expansions in protein-coding genes for F-box and MYB TFs suggest diversified regulatory and signaling processes, including phytohormone signaling processes^30,43–47^. Despite these expansions, Pfam domain repertoires of unicellular and multicellular streptophyte algae showed 94% similarity (Fig. 3d).

Next, we investigated streptophyte protein domain combinations exclusive to multicellular algae and land plants compared with unicellular algae. The combination of EDR1, LRR_8 and Pkinase domains (PF14381, PF13855 and PF00069) probably evolved in the streptophyte LCA (Fig. 3e), occurring in the *Arabidopsis* putative Raf-related kinase (AT1G04210), involved in SnRK2 activation and osmotic stress response^48^, E3 ubiquitin ligase interaction in regulating programmed cell death^49^, and MAPK cascade activation^50^. The ubiquitin–homologous to the E6-AP carboxyl terminus (HECT) combination (PF00240 and PF00632) (Fig. 3f) occurs in *Arabidopsis* UPL5 (AT4G12570), which is intertwined with intracellular signaling—featuring jasmonate and H_2_O_2_—in development and leaf senescence^51^. All multicellular algae and embryophyte species possess this domain combination in at least one ortholog. It thus probably dates back to a deep LCA, suggesting secondary loss in unicellular algae. ARP8 (AT5G56180; Fig. 3g) stands out by combining F-box like (PF12937) and actin (PF00022) domains. It is involved in the ubiquitin E3 SCF complex, cell cycle regulation and chromatin remodeling via ubiquitin–proteosomal degradation^52^. This combination is exclusive to multicellular streptophyte algae and land plants and probably emerged in their LCA (Fig. 3g). A prominent combination particular for *Zygnema* is the Lectin_legB domain, one of the many lectin families^53^ important for plant immunity and development. We found Lectin_legB with other domains in 26 different combination architectures, often lineage-specific and differentially expressed (Fig. 3h,i).

Key to the elaborate multicellular development of land plants are Type II MADS-domain (or MIKC-type) transcription factors (TFs), featuring a keratin-like (K) domain for forming floral quartet-like complexes (FQCs)^54,55^. The increase and diversification of these TFs is tightly associated with evolutionary novelties^55^. Each *Zygnema* genome encodes one MADS-domain TF. They form a clade (Supplementary Fig. 16) and lack K domains. In transcriptomes^4,56^, however, we found MADS-box genes encoding a K domain in other Zygnematophyceae including also a *Zygnema* species, forming a second separate clade, that was apparently lost in the *Zygnema* species sequenced here (Supplementary Fig. 16). This suggests the presence of two MADS-domain TFs in the Zygnematophyceae ancestor: (1) an ancestral Type II without a K domain (probably unable to form FQCs), and (2) the MIKC type, with (in vitro) demonstrated ability to form FQCs^57^.

Overall, several protein domain combinations that are exclusive to multicellular species seem associated with fine-tuned regulation of cell division and differentiation. New protein domain combinations might have arisen through gene fusions, many of which occurred already in the LCA of the green lineage (Chloroplastida). On balance, the number of specific genes and domain combinations was humble. These patterns align with proposed concepts on the evolution of multicellularity in green algae^58,59^. It is rather the regulation of a conserved set of genes that underpins multicellularity than a burst of novelty, combined with secondary losses. To such regulation, we turn later in this study.

### Gene gains facilitated major cell wall innovations

**Fig. 4:**
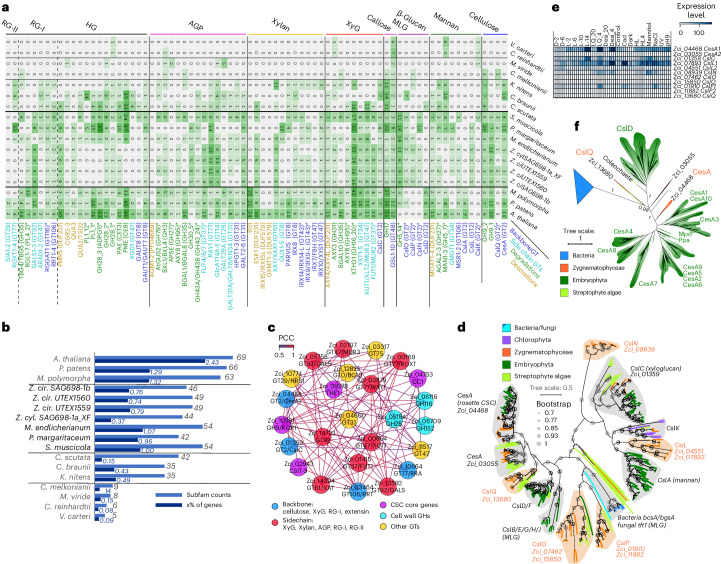
Cell wall innovations revealed by protein family analyses. **a**, A heatmap of homolog presence in 77 enzyme subfamilies (rows) across 17 plant and algal genomes (except for *Coleochaete scutata,* for which we used its transcriptome). The enzyme subfamilies are grouped by polysaccharide, and the colors indicate their biochemical roles; phylogenetic patterns compatible with gene gains that might have involved HGT are indicated with asterisks. **b**, Counts of subfamilies and gene percentages (with respect to the total annotated genes) across the 17 species. Shown in the plot is the gene percentage × 100. **c**, The co-expression network of SAG 698-1b containing 25 genes (most belonging to the 77 analyzed subfamilies) involved in cell wall polysaccharide syntheses. **d**, The phylogeny of GT2 across the 17 species. Major plant CesA/Csl subfamilies are labeled by the SAG 698-1b homolog, and newly defined subfamilies are in red. Ten bacterial β-glucan synthase (BgsA) and fungal mixed-linkage glucan (MLG) synthase (Tft1) homologs are included to show their relationships with plant CesA/Csl subfamilies. **e**, Gene expression of 11 SAG 698-1b GT2 genes across 19 experimental conditions (3 replicates each). **f**, The phylogeny of GT2 with ZcCesA1 (Zci_04468) homologs retrieved by BLASTP against the protein non-redundant (nr) database of the National Center for Biotechnology Information (NCBI) (E-value <1 × 10^−10^); colors follow **d**, and >5,000 bacterial homologs from >8 phyla are collapsed (blue triangle). See Supplementary Data 1–13 for details. *Z. cir./Z. ci*, *Zygnema circumcarinatum*; *Z. cyl*., *Zygnema* cf. *cylindricum*. Source data

The cellulosic fibrils of the cell wall are a biophysical denominator in multicellular morphogenesis of plants, forming the first layer of protection from the environmental stressors that also the earliest land plants had to overcome^60^. We reconstructed the evolutionary history of 38 cell wall-related enzyme families (Supplementary Table 1l), which were further split into 77 well-supported subfamilies (Fig. 4a and Supplementary Table 1m). Most subfamilies belong to carbohydrate active enzyme (CAZyme) families known for the synthesis and modifications of celluloses, xyloglucans, mixed-linkage glucans, mannans, xylans, arabinogalactan proteins (AGPs) and pectins (Fig. 4a and Supplementary Table 1l,m). Analyzing the 77 enzyme subfamilies (Supplementary Text 3 and Supplementary Data 1) revealed the following: (1) Z + E share all the major enzymes for the synthesis and modifications of the diverse polysaccharide components, including those for sidechains and modifications (Fig. 3b; 42–54 subfamilies in Zygnematophyceae versus 63–69 in Embryophyta). (2) Many of the enzymes for cell wall innovations, especially for polysaccharide backbone synthesis, have older evolutionary origins in the LCA of Klebsormidiophyceae and Phragmoplastophyta (Fig. 4b; 35–69 subfamilies versus 8–9 in Chlorokybophyceae and Mesostigmatophyceae). Many of such subfamilies are expanded in Zygnematophyceae (Fig. 4b; for example, GH16_20, GT77, CE8 and CE13 in Fig. 4a). (3) Genes involved in the syntheses of different cell wall polymers (backbones and sidechains) are co-expressed in SAG 698–1b (Fig. 4c). (4) Phylogenetic patterns suggest that some of the enzymatic toolbox for cell wall polysaccharide metabolism originated via HGT (Fig. 4a), pronounced for degradation enzymes (for example, GH5_7, GH16_20, GH43_24, GH95, GH27, GH30_5, GH79, GH28, PL1 and PL4), but it is also observed for GT enzymes. (5) Frequent gene loss creates scattered distributions of homologs in Streptophyta (Fig. 4a; for example, *Zygnema* lacks entire families or some subfamilies of GH5_7, GH35, GT29, GT8, CE8, GH28 and PL1).

We scrutinized the GT2 family, which contains major cell wall synthesis enzymes such as cellulose synthase (CesA) and Csl (CesA-like) for hemicellulose backbones (Fig. 4d and Supplementary Text 3). Among the 11 SAG 698-1b *CesA*/*Csl* homologs, Zc*CesA1* (Zci_04468), Zc*CslL1* (Zci_07893), Zc*CslC* (Zci_01359), Zc*CslN* (Zci_08939) and Zc*CslP1* (Zci_0910) are induced by various stresses^61^ (Fig. 4e). The two *CesA* homologs in SAG 698-1b (Fig. 4d,f) and all other Zygnematophyceae homologs are (co-)orthologs of land plant *CesA*. Zc*CesA1* (Zci_04468) is co-expressed with four known plant primary cell wall cellulose synthase complex (CSC) component core genes: *KOR* (Zci_10931), *CC1* (Zci_04753), *CSI1* (Zci_02943) and *THE* (Zci_09278) (Fig. 4c). This extends previous observations^62^ suggesting that co-expression of CSC component genes is evolutionarily conserved since the common ancestor of Zygnematophyceae and land plants.

Overall, the phylogenetic analyses of key cell wall enzymes (Supplementary Table 1l and Supplementary Text 3) highlight the importance of ancient HGTs contributing to evolutionary innovations of cell walls, similarly to what has been proposed for other traits^9,18^.

### Co-expression connects environment and multicellular growth

**Fig. 5:**
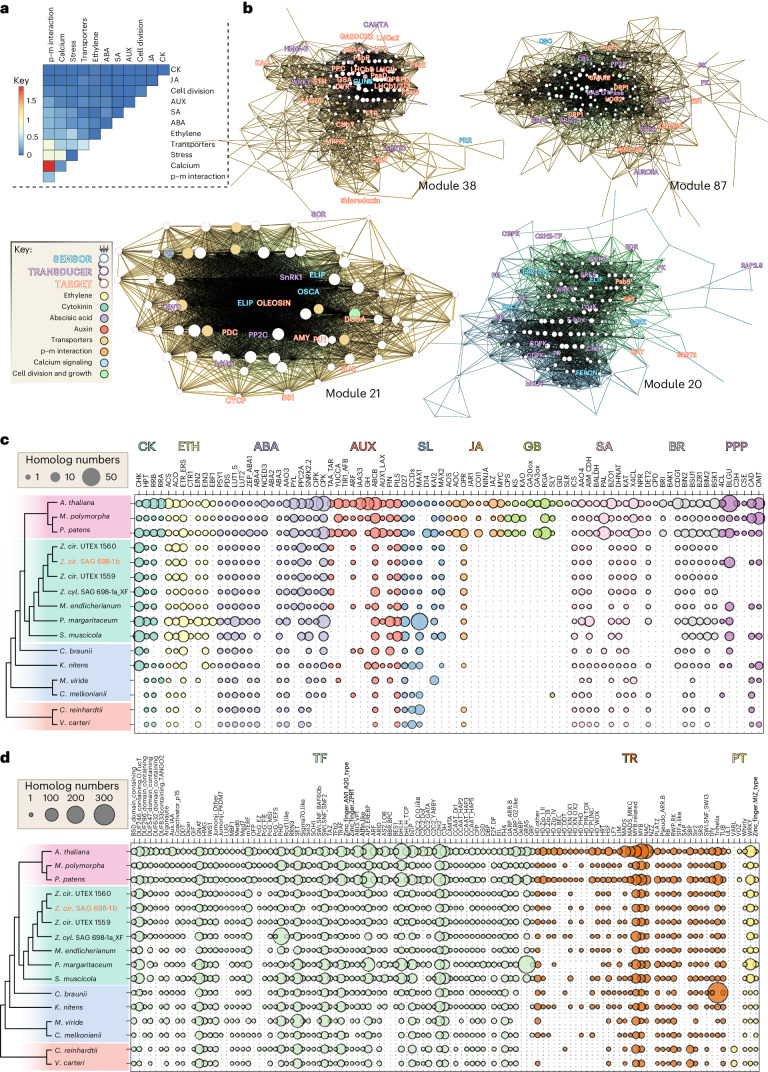
Gene co-expression modules and phylogenetic distribution of land plant signature specialized metabolism and TFs. **a**, Heatmap of per-module co-occurrence frequencies among genes associated with plant–microbe (p–m) interaction, calcium signaling, stress, transporters, cell division and diverse phytohormones (see abbreviations below); based on 150 out of 406 total gene co-expression modules showing co-occurrence of at least two functional categories. **b**, Modules 20, 21, 38 and 87 discussed in the main text; node (gene) sizes are proportional to number of neighbors and edge (co-expression) widths are proportional to Pearson’s correlation coefficient whereas colors are those of interconnected genes; egde gradient colors highlight the two dominant gene categories as indicated in the key. Font colors indicate genes’ likely roles in establishing a flow of information. The full gene co-expression results can be accessed in our online portal (https://zygnema.sbs.ntu.edu.sg/). The gene names are based on homology and the proteins they likely encode. **c**, The phylogenetic distribution of genes coding for proteins involved in phytohormone biosynthesis, signaling and phenylpropanoid biosynthesis. **d**, The phylogenetic distribution of genes coding for TFs. CK, cytokinin; ETH, ethylene; AUX, auxin; SL, strigolactone; JA, jasmonic acid; GB, gibberellic acid; SA, salicylic acid; BR, brassinosteroids; PPP, phenylpropanoid; TR, transcriptional regulators; PT, putative transcription-associated proteins. Note that the high number of homologs found in *Penium margaritaceum* are probably due to the large genome of 3.6 Gb and >50,000 annotated proteins. *Z. cir*., *Zygnema circumcarinatum*; *Z. cyl*., *Zygnema* cf. *cylindricum*. Source data

We computed co-expression networks and searched for homologs related to (1) cell division and development, (2) multicellularity, (3) stress response, (4) transporters, (5) phytohormones (see also Supplementary Figs. 17–20), (6) calcium signaling and (7) plant–microbe interaction (Supplementary Table 3). A total of 150 out of 406 modules showed co-occurrence of at least two such functional categories, the most frequent co-occurrence being plant–microbe interaction and calcium signaling, followed by plant–microbe interaction and stress (Fig. 5a). To understand the cohorts of genes that can establish the flow of information from external stimuli to the adjustment of internal programs, we additionally explored the above 150 modules for the layered system of (1) sensors, (2) signal transducers and (3) internal programs such as cell division and growth. Sensors co-express with transducers such as protein kinases and TFs (for example, modules 2, 20, 21, 23, 126, 147 and 173). Several such modules (Fig. 5b and Supplementary Text 4) contain *ELIP*s, coding for proteins that respond to light stimulus and can reduce photooxidative damage by scavenging free chlorophyll^63^ under cold stress (module 21; Fig. 5c and Supplementary Fig. 21)—as shown for other streptophyte algae^10,64^—but also under high light (HL), expressed alongside a chaperon-coding gene and *PsbS* (key for NPQ; module 20). Module 38 features an *OLEOSIN* homolog, bolstering their importance in zygnematophytes^65^.

Signal transduction and processing featured genes for kinases (calcium-dependent and LRR receptor-like kinases), PP2C and TFs (for example, modules 13, 57, 96, 107, 121, 130, 148, 161 and 170) and their frequent co-expression with well-known downstream genes for cell division (for example, modules 10, 22, 52, 87, 117, 128 and 179) and stress response (for example, modules 38, 74, 88, 90, 123 and 151). For example, module 87 features genes for calcium-dependent, cyclin-dependent and receptor-like protein kinases and Ras-related signaling proteins (for example, RAB GTPases) that are involved in cell growth, CHK histidine kinase of the cytokinin signaling network and downstream genes for cell growth and division such as microtubule-associated proteins, dynamins or kinesins. Module 38 features genes for SCR TF and protein kinases. These co-express with phytohormone genes of the abscisic acid (ABA) pathway (*ABA4* and *LUT2*), an auxin-response factor (*ARF10*) ortholog, and a gibberellin 20 oxidase (*GA20OX2*) homolog—despite the lack of gibberelins in *Zygnema*; all in addition to cell growth and division-related genes such as kinesin, transglutaminases and many photosynthesis-related genes.

An example for the tight link of calcium signaling and biotic interaction (Fig. 5a) is the co-expression of genes for LRR proteins with the calcium sensor and kinase (CPK; Zci_12352) in module 128 and CDPKs in module 117 (Supplementary Fig. 21). The most connected node in module 117 is an *LRR* and it also features *PP2C*s. While calcium signaling has recently been proposed to link plant pattern- and effector-triggered immunity^66,67^, it is also important in mutualistic interactions^68^.

The frequent overlaps between sensors and transducers and between transducers and downstream targets suggest a hierarchy where environmental cues are received, transmitted and processed, allowing a complex downstream response that integrates a variety of extrinsic and intrinsic signals. This aligns with the idea that the biology of plant cells hinges on a molecular information-processing network^69^. Our co-expression analyses recover joint action of genes for first sensing the environment and then modulating growth and stress response mechanisms in *Z. circumcarinatum*. We interpret some of these joint actions as signatures for a homologous genetic network that dates back (at least) to an ancestor of Zygnematophyceae and land plants.

The symbiotic association with fungi was one of the key innovations that allowed plants to colonize land^70^. All four genes involved in symbiotic functions were found in *Zygnema*: *DMI2/SYMRK* pro-ortholog (Zci_05951), *DMI1/POLLUX* (Zci_12099), *DMI3/CCaMK* (Zci_01672) and *IPD3/CYCLOPS* (Zci_13230; Supplementary Figs. 13–15). These genes belong in different modules (134, 78, 172 and 159, respectively), suggesting that the evolution of symbiosis in land plants recruited genes from diverse pathways rather than directly co-opting an existing pathway into a new function^71^.

A comprehensive analysis of transcription-associated proteins (TAPs) with TAPscan^72^ v.3 revealed higher numbers of TFs in land plants than in algae, as expected due to their more complex bodies and *Zygnema* species having comparatively more TAPs than other algae (Fig. 5d and Supplementary Table 3c). To further investigate the evolution of coordinated multicellular growth, we compiled a list of 270 genes with experimental evidence for roles in cell division (Supplementary Table 3d), finding that *Zygnema* lost microtubule plus tip proteins CLASP and SPIRAL1, potentially associated with the loss/reduction of rhizoids and phragmoplast-mediated cell division (cleavage instead). Various gene modules (Supplementary Fig. 21) reflect cell division by co-expression of genes for proteins such as phragmoplastin (*DRP1*) (module 87), kinesin motors (for example, modules 52 and 87), spindle assembly (module 52; Supplementary Fig. 21), RAB GTPases (modules 10 and 87), SNARE (modules 52 and 87), cargo complex components (modules 10 and 87) and cell division-related kinases. Genes that probably originated in the Z + E LCA are *UGT1*, *SUN1/SUN2* and *LONESOME HIGHWAY*. The clearest cases of genes originating in the Z + E LCA code for GRAS TFs^9^, including pro-orthologs of *SCARECROW* (*SCR*), *SCARECROW*-like and *SHORTROOT* (Supplementary Fig. 12), regulators of embryophyte cell division orientation and tissue formation—but also abiotic stress responses^73–76^. *Zygnema* GRAS homologs co-express with genes involved in cell division, cell cycle regulation and cell wall functions (modules 147, 38 (Fig. 5b) and 93). All three modules contain genes associated with abiotic stress responses, such as an *ELIP* homolog (OG 97; expanded in *Zygnema*), β-glucosidase (OG 85; expansion in Z + E LCA), calcium cation channel (DMI1/POLLUX/CASTOR) and other calcium signaling components. The involvement of GRAS TFs in developmental and environmental signaling speaks of a complex network to coordinating growth and stress since the LCA of Z + E.

### Evolution of phytohormone pathways

Phytohormone biosynthesis and signaling networks have deep evolutionary roots. While gibberellins and jasmonates probably originated in land plants^30^, other phytohormone pathways were at least partly present in algal ancestors (Fig. 5c and Supplementary Text 5). Land plants have more phytohormone-associated homologs than algae, as expected for their more complex signaling pathways^77^, and Zygnematophyceae are overall similar to other streptophyte algae (Fig. 5c).

For example, despite the ABA biosynthesis pathway being incomplete, we detected 1.01 ± 0.13 ng g^−1^ ABA in SAG 698-1b by liquid chromatography–mass spectrometry (Supplementary Fig. 17). The presence of diverse carotenoid cleavage dioxygenases (Supplementary Figs. 18–20) might point to alternative biosynthetic routes; perhaps via an ABA1-independent pathway starting upstream of zeaxanthin as suggested earlier^78^. Major aspects of the ABA signaling network are conserved across land plants^79^. The four new *Zygnema* genomes contain a complete set of homologous genes to the ABA signaling cascade, including the receptors, corroborating previous data on Zygnematophyceae^9,10^. Functional data showed that *Zc*PYL regulates PP2C in an ABA-independent manner^25^.

### Microexons evolved during plant terrestrialization

**Fig. 6:**
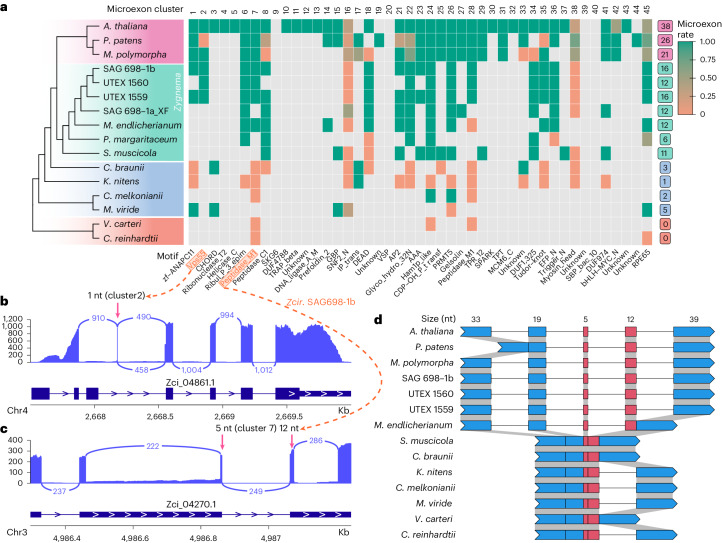
Microexon prediction in 16 plant and algae genomes. **a**, Heatmap of 45 conserved microexon-tags predicted by MEPmodeler. Microexon rate is the rate of true microexons among all predicted results in the cluster. For example, green cells indicate that 100% microexons with two flanking introns are present, orange indicates all microexon sequences are parts of large exons and none of them could be considered as microexons, and the others are between 0 and 1. A gray cell indicates missing data (a microexon-tag could not be found). The numbers on the right column indicate the predicted clusters containing at least one true microexon (see ref. ^80^ for more detail). **b**, RNA-seq evidence of the 1 bp microexon in cluster 2 (*x* axis the genomic location, and *y* axis the read count). **c**, RNA-seq evidence of two adjacent microexons, 5 (cluster 7) and 12 bp (cluster 28). In **b** and **c**, the RNA-seq of condition p881sControl2 was used; RNA-seq read depth (blue numbers) and gene annotation are shown; blue arcs indicate introns (exon–exon junctions), and the numbers indicate the junction read counts supporting the introns. The pink arrows point to microexons. **d**, Exon–intron structures of microexon-tag clusters 7 and 28 in 14 plant genomes. The structure was predicted by relaxing the stringency in *M. viride* genome and by doing TBLASTX search in *S. muscicola* genome (all three copies are intronless in this microexon-tag), respectively. The others are predicted with default parameters. Source data

Microexons (~1–15 bp) can be evolutionarily conserved and crucial for plant gene functions^80^. We predicted 45 microexon-tags in 16 plant genomes using MEPmodeler^80^. Land plants typically have >20 of 45 microexon-tag clusters. In Zygnematophyceae, we found 10–20 microexon-tag clusters (6 in *Penium margaritaceum* probably due to the fragmented genome assembly; Table 1), <5 in other streptophytes and none in Chlorophyta (Fig. 6). Zygnematophyceae and land plants have the most microexons. For example, a 1 bp microexon of cluster 2 was found in *Vps55* (Zci_4861) (Fig. 6b). Two adjacent microexons, 5 bp (cluster 7) and 12 bp (cluster 28) were found in a Peptidase M1 family gene (Zci_04270), which were overlooked by the de novo gene annotation (annotated as UTR and missed a Peptidase M1 motif) but verified by RNA sequencing (RNA-seq) (Fig. 6c). The two adjacent microexons are in the context of a 108 bp coding region spanning five exons in the *Arabidopsis* gene (AT1G63770.5). The five-exon structure is only conserved in land plants and *Zygnema* (Fig. 6d), whereas in *Mesotaenium* *endlicherianum* the last two exons (including the 112 bp) are fused, and all other algae have two or three exons with two adjacent microexons of clusters 7 and 28 always fused. It appears that, during terrestrialization, at least for this Peptidase M1 family gene, there was a gradual intronization process that created more microexons in land plants.

## Discussion

We generated chromosome-level genome assemblies for four filamentous algal sisters to land plants and performed comprehensive comparative genomics and co-expression network analyses. We found molecular innovations for signaling, environmental response and growth, and pinpoint their evolutionary history by tracing gene family expansions along the phylogenetic backbone of streptophytes. The reconstruction of ancestral gene content is a powerful means to explain the evolution of plant form and function as well as biological novelty^81^. Our data indicate the dynamics in Zygnematophyceae genome evolution (Fig. 2a), highlighting the need for a phylodiverse species set and the integration of complementary comparative approaches to understand the nature of the LCA of land plants and algae.

Throughout their evolutionary history, Zygnematophyceae have transitioned several times between multicellular and unicellular body plans^6^. A parsimonious explanation is that streptophytes share an ancient toolkit for multicellularity^82,83^, which comes to bear in filamentous genera but is still lingering as genetic potential in zygnematophyte unicells. And indeed, our data on shared OG expansions recover several important regulatory genes for increasing cellular complexity in the LCAs both of Z + E and of Zygnematophyceae. While we recover some specific protein domain gains, losses and combinations that might underpin actualization of filamentous growth, it appears more likely that the regulation of the shared toolkit for multicellularity is the critical factor in the evolution of filamentous algal bodies.

A defining feature of land plants is the plastic development of their multicellular bodies, ever adjusting to environmental conditions. High connectivity between genes involved in multicellularity and environmental stress response establishes the foundation for an adaptive advantage of multicellular morphogenesis, where cell differentiation can be fine-tuned for acclimation to environmental cues.

Genes that are co-expressed are often functionally related and concertedly act in genetic programs. We recover programs of an intrinsic nature, such as growth and development, cell division and cell wall biosynthesis/remodeling and genes that act in environmental sensing and signaling, triggered by an extrinsic input. In an interconnected module, there is an implicit directionality (outside/environment to inside). By their nature, signaling proteins must act in a genetic hierarchy (transduction through kinase cascades), and so do TFs (there must be an upstream and downstream). Both are co-expressed with intrinsic growth programs, thus revealing links between internal and external, suggesting joint actions of genes to sense the environment and modulate growth and reveal the genetic network underpinning molecular information processing in both plant and algal cells. This network has deep evolutionary roots, dating back at least to the ancestor of Z + E (Supplementary Text 4 and Supplementary Fig. 21).

Our data demonstrate a deep evolutionary origin of plant signaling cascades for acclimation to environmental cues and suggest a deep conservation of interconnections with regulation of growth—connections between extrinsic environmental input and intrinsic developmental programs that were drawn before Embryophyta began their conquest of land.

## Methods

### Algal strains

*Z. circumcarinatum* SAG 698-1b and *Z*. cf. *cylindricum* SAG 698-1a were obtained from the Culture Collection of Algae at Göttingen University (SAG) (https://sagdb.uni-goettingen.de); from 698-1a, a single filament was isolated and used to establish a new culture that we coined 698-1a_XF and deposited at SAG. *Z. circumcarinatum* UTEX 1559 and UTEX 1560 were obtained from the UTEX Culture Collection of Algae at the University of Texas Austin (https://utex.org/). For the history of these strains, see Supplementary Text 1.

### Transmission electron microscopy

Transmission electron microscopy was essentially performed as previously described^84^ using two independent cell cultures and each time ≥15 algal filaments. One-month-old cultures of *Zygnema circumcarinatum* (SAG 698-1b) and 3-month-old cultures of *Z.* cf. *cylindricum* (SAG 698-1a) were fixed in 2.5% glutaraldehyde (in 20 mM cacodylate buffer, pH 6.8) for 1.5 h and rinsed with 20 mM cacodylate buffer, embedded in 3% agarose and post fixed in 1% OsO_4_ (in 20 mM cacodylate buffer) at 4 °C overnight and dehydrated in increasing ethanol concentrations. Samples were transferred in propylene oxide and embedded in modified Spurr’s resin and sectioned with a Reichert Ultracut (Leica Microsystems). The ultrathin sections were stained with 2% uranyl acetate and Reynold’s lead citrate. Transmission electron micrographs were taken on a Zeiss Libra 120 transmission electron microscope (Carl Zeiss AG) at 80 kV, which was equipped with a TRS 2k SSCCD camera and operated by ImageSP software (Albert Tröndle Restlichtverstärker Systeme).

### DNA and RNA sequencing

Detailed protocols for DNA and RNA extraction have been published elsewhere^14,61,85^ and are, together with more details on genome and transcriptome sequencing and assembly, detailed in Supplementary Materials and Methods. For RNA-seq, we subjected *Z. circumcarinatum* SAG 698-1b to 19 growth and stress conditions, after which RNA-seq was obtained for the construction of a gene co-expression network. Stress and RNA-seq experiments were done in three baches. The first batch followed Pichrtová et al.^86^ and de Vries et al.^10^ with modifications. Three-week algae were subcultured in 12 flasks of liquid Bold’s Basal Medium (BBM) with 0.02% l-arginine and grown for 2 weeks under standard conditions: 16 h/8 h of light/dark cycle at 20 °C and ~50 µmol photons m^−2^ s^−1^. Then, the algae were treated for 24 h under four conditions: (1) 20 °C in liquid medium (standard control), (2) 4 °C in liquid medium, (3) desiccation at 20 °C and (4) desiccation at 4 °C. Four treatments each with three replicates were performed. For desiccation treatments, algae were harvested using a vacuum filtration with Glass Microfiber Filter paper (GE Healthcare, 47 mm) and 20 µl of modified BBM (MBBM) was added on the filter paper. Papers with algae were then transferred onto a glass desiccator containing saturated KCl solution^86^, and the desiccator was sealed with petroleum jelly and placed in the growth chamber under standard culture conditions. Cultures grown in liquid conditions were harvested using a vacuum filtration with Whatman #2 paper (GE Healthcare, 47 mm). After 24 h of treatment, the 12 samples were transferred into 1.5 ml Eppendorf tubes and immediately frozen in liquid nitrogen and stored in −80 °C. For the second batch (six diurnal experiments), the algae were grown with the same control conditions as the above mentioned (16 h/8 h of light/dark cycle, 20 °C, ~50 µmol of quanta per squared meter per second) and samples were collected every 4 h: (5) diurnal dark 2 h, (6) diurnal dark 6 h, (7) diurnal light 2 h, (8) diurnal light 6 h, (9) diurnal light 10 h and (10) diurnal light 14 h. For the third batch (nine stress experiments): SAG 698-1b was precultivated at 20 °C, 16 h/8 h light/dark cycle at 90 µmol photons per squared meter per second on a cellophane disks (folia Bringmann) for 8 days. For certain treatments (NaCl, mannitol and CadmiumCl) the culture was transferred to a new Petri dish where the medium was supplemented with the substances. Algae where then subjected to (11) 150 µM NaCl (Roth) for 24 h, (12) 300 mM mannitol (Roth) for 24 h, (13) 250 µM CadmiumCl (Riedel-de Haën AG) for 24 h, (14) dark treatment for 24 h, (15) high light (HL) treatment at 900 µmol photons per squared meter per second for 1 h, (16) ultraviolet-A at 385 nm, 1,400 µW cm^−^^2^ for 1 h, (17) HL at 4 °C (HL4) at 900 µmol photons per squared meter per second for 1 h, (18) pH 9 for 24 h, and (19) a corresponding control growth at 20 °C on a plate.

### Library preparation and sequencing

The four genomes were sequenced by a combination of PacBio High-Fidelity (HiFi) long reads, Oxford Nanopore long reads and Illumina short reads (Supplementary Table 1a,b). DNA samples were sequenced at the Roy J. Carver Biotechnology Center (University of Illinois, Urbana-Champaign) using Oxford Nanopore and Illumina technologies (Supplementary Table 1a). Oxford Nanopore DNA libraries were prepared with 1D library kit SQK-LSK109 and sequenced with SpotON R9.4.1 FLO-MIN106 flowcells for 48 h on a GridIONx5 sequencer. Base calling was performed with Guppy v1.5 (https://community.nanoporetech.com). Illumina shotgun genomic libraries were prepared with the Hyper Library construction kit (Kapa Biosystems, Roche). Libraries' fragment size averaged at 450 bp (250–500 bp) and were sequenced with 2×250 bp paired-end reads on a HiSeq 2500. Additional DNA samples were sequenced at the Genome Research Core (University of Illinois, Chicago) and Joint Genome Institute (JGI; Berkeley, California). The Illumina shotgun genomic libraries were prepared with the Nextera DNA Flex Library Prep Kit. Fragment sizes averaged at 403 bp and were sequenced with 2 × 150 bp paired-end reads on HiSeq 4000 (Supplementary Table 1a). RNA samples were sequenced at the Genome Research Core (University of Illinois, Chicago). The libraries were prepared by ribosomal RNA (rRNA) depletion with Illumina Stranded Total RNA kit plus Ribo-Zero Plant^87^, and 2 × 150 bp paired-end sequencing was performed on HiSeq 4000. RNA from the third batch of stress experiments were sequenced at the NGS-Integrative Genomics Core Unit of the University Medical Center Göttingen, Germany. Stranded messenger RNA libraries were prepared with the Illumina stranded mRNA kit, and paired-end sequencing of 2×150 bp reads was carried out on an Illumina HiSeq 4000 platform. RNA-seq data for SAG 698-1a and UTEX 1559 have been previously published (Supplementary Table 1a).

### Genome assembly and scaffolding

To assemble the genome of SAG 698-1b, a total of 5.4 Gb (82×) of Oxford Nanopore nuclei DNA reads were assembled with wtdbg (v2)^88,89^. Assembled contigs were polished by Racon^90^ and three iterations of pilon^91^ with Illumina paired-end reads. The polished genome was scaffolded by Dovetail Genomics HiRise software with Hi-C sequencing data (https://dovetailgenomics.com/). Genome contamination was examined by BLASTX against NCBI’s non-redundant (nr) database, and contaminated scaffolds were removed.

To assemble the UTEX 1559 genome, an initial assembly was done with SPAdes^92^ using Illumina paired-end reads (2 × 150 bp), three mate-pair libraries (insert size 3–5 kb, 5–7 kb and 8–10 kb) and Oxford Nanopore reads (Supplementary Table 1a). Assembled contigs were further scaffolded by two rounds of Platanus-allee^93^ with Illumina paired-end reads (2 × 250 bp), three mate-pair libraries (insert size 3–5 kb, 5–7 kb and 8–10 kb) and Oxford Nanopore reads. For the UTEX 1560 genome, Illumina paired-end (2 × 150 bp) and PacBio HiFi reads were used for assembly with SPAdes and further scaffolded with Platanus-allee. Scaffolds with contaminations were identified by BLASTX against NR and removed. The genomes of UTEX 1559 and UTEX 1560 were scaffolded by Dovetail Genomics HiRise software with Hi-C sequencing data from SAG 698-1b.

The genome of SAG 698-1a_XF was sequenced with PacBio HiFi long reads (40 Gb), Nanopore long reads (4 Gb) and Illumina short reads (>100 Gb). The *k*-mer analysis using Illumina reads revealed two peaks in the *k*-mer distribution, suggesting that SAG 698-1a_XF exists as a diploid organism with an estimated heterozygosity rate of 2.22% (Supplementary Fig. 2). All Illumina short reads and the Nanopore reads were first assembled into contigs using SPAdes. Then, WENGAN^94^ was used to assemble HiFi long reads and Illumina paired-end reads (2 × 150 bp) using the SPAdes contigs as the reference. Lastly, the resulting WENGAN contigs were scaffolded and gaps were closed with Platanus-allee using all the Nanopore, HiFi and Illumina reads to derive a consensus pseudo-haploid genome.

To evaluate the quality of assembled genomes (Supplementary Table 1d), raw RNA-seq reads, Oxford Nanopore and Illumina DNA reads were mapped to the assembly with hisat (v2)^95^, minimap (v2)^96^ and bowtie (v2)^97^, respectively. To assess genome completeness, a BUSCO^98^ analysis was performed with the ‘Eukaryota odb10’ and ‘Viridiplantae odb10’ reference sets.

### Genome annotation

In all four genomes, protein coding genes were predicted by the MAKER-P pipeline^99^, which integrates multiple gene prediction resources, including ab initio prediction and homology- and transcripts-based evidence. First, repetitive elements were masked by RepeatMasker with a custom repeat library generated by RepeatModeler. Rfam with infernal and tRNA-Scan2 were used to analyze noncoding RNA and transfer RNA (tRNA). For the transcript evidence, a total of 103,967 transcripts were assembled by Trinity (reference-free) and StringTie (reference-based) from the respective RNA-seq data. Transcriptome assembly was used to generate complete protein-coding gene models using the tool Program to Assemble Spliced Alignments (PASA). Proteins from *Mesotaenium* *endlicherianum*, *Spirogloea* *muscicola* and *Arabidopsis* *thaliana* (TAIR10) were used for homology-based evidence. Then, the resulting protein-coding gene models from the first iteration of the MAKER-P pipeline were used as the training data set for SNAP and Augustus models, which were fed into MAKER for the second iteration of annotation. After three rounds of gene prediction, MAKER-P combined all the protein-coding genes as the final annotated gene set.

### Plastome and mitogenome assembly and annotation

NOVOPlasty 3.8.2 (refs. ^100,101^) was used to assemble plastomes. The contiguity of assembled plastomes was examined in Geneious (https://www.geneious.com/)^102^ with read mapping. For SAG 698-1b mitogenome assembly, Oxford Nanopore reads were assembled with Canu^103^, where one long mitogenome contig of 238,378 bp was assembled. This contig was circularized and polished with three rounds of pilon^91^, which was further corrected with Illumina raw reads and compared with the mitogenome of UTEX 1559 (MT040698)^85^ in Geneious. For SAG 698-1a_XF, PacBio HiFi reads were used for the assembly of its mitogenome.

Plastome and mitogenome annotation was performed with GeSeq^104,105^. For plastome annotation, BLAT search and HMMER profile search (Embryophyta chloroplast) were used for coding sequence, rRNA and tRNA prediction; ARAGORN v1.2.38, ARWEN v1.2.3 and tRNAscan-SE v2.0.5 were used for tRNA annotation. For mitogenome annotation, Viridiplantae was used for BLAT reference sequences. The annotated gff files were uploaded for drawing circular organelle genome maps on OGDRAW^106,107^.

The plastome of SAG 698-1b is identical to those of UTEX 1559 (GenBank ID MT040697)^85^ and UTEX 1560. The mitogenomes of SAG 698-1b (OQ319605; Supplementary Fig. 3) and UTEX 1560 are identical in sequence but slightly longer than that of UTEX 1559 (MT040698, 215,954 bp)^85^ (Supplementary Fig. 4). The plastome of SAG 698-1a was available^108^. Its mitogenome (OQ316644) (Supplementary Fig. 5), at 323,370 bp size, is the largest known among streptophyte algae (Supplementary Table 1g,h).

### Repeat annotation and analysis

Repetitive DNA was annotated using the homology strategy with repeat libraries generated with RepeatModeler. RepeatModeler integrates RepeatScout, RECON, LTRharvest and LTR_retriever tools (version 2.0.1; refs. ^109,110^). The miniature inverted-repeat transposable elements (MITE) library was identified with MITE-tracker^111^. These two identified libraries were combined and incorporated into RepeatMasker (v.4.0.9; http://www.repeatmasker.org/) for repeat annotation. SAG 698-1b contains mostly simple repeats (6.4%) and transposable elements of the MITE (4.3%), Gypsy (2.9%) and Copia (1.9%) families. The *Z.* cf. *cylindricum* SAG 698-1a_XF genome has Copia (29.8%), MITE (11.6%), Gypsy (5.9%) and simple repeats (2.1%)

### Comparative genomics analysis

Sixteen representative genomes were selected, including chlorophytes (*Volvox carteri*^58^ and *Chlamydomonas reinhardtii*^112^), Zygnematophyceae (*Z. circumcarinatum* SAG 698-1b, UTEX 1559, UTEX 1560, *Z*. cf. *cylindricum* SAG 698-1a_XF, *Mesotaenium endlicherianum*^9^, *Penium margaritaceum*^7^ and *Spirogloea muscicola*^9^), additional streptophyte algae (*Chara braunii*^11^, *Klebsormidium nitens*^113^, *Chlorokybus melkonianii*^114,115^ (a strain formerly known as *C. atmophyticus*) and *Mesostigma viride*^114^), bryophytes (*Marchantia polymorpha*^116^ and *Physcomitrium patens*^117^) and a vascular plant (*Arabidopsis thaliana*^118^).

OGs were inferred with Orthofinder^119^. Time-calibrated species phylogeny was built with low-copy OGs (≤3 gene copies per species). Divergence time estimation was carried out with MCMCTree. Expanded and contracted gene families were identified with CAFE and the species phylogeny. For microexon analyses, MEPmodeler^80^ was used^120^.

For comparative genomics analyses of multicellularity, the 16 genomes were classified into two groups, unicellulars (*Chlamydomonas* *reinhardtii*, *Chlorokybus* *melkonianii*, *Mesostigma* *viride*, *Spirogloea* *muscicola*, *Mesotaenium* *endlicherianum* and *Penium* *margaritaceum*) and multicellulars (*Volvox* *carteri*, *Klebsormidium* *nitens,* *Chara* *braunii*, SAG 698-1a_XF, SAG 698-1b, UTEX 1559 and UTEX 1560, *Marchantia* *polymorpha*, *Physcomitrium* *patens* and *Arabidopsis* *thaliana*). Proteins in the 16 genomes were annotated against the Pfam database to find functional domains. Domain occurrences (presense/absence) and abundances in each genome were recorded and compared between the two groups to infer domain gain, loss and combination.

Comparative genomics were performed with 16 representative green algal and plant genomes. Annotated proteins were clustered into OGs by OrthoFinder. A total of 4,752 OGs contained proteins from at least one representative of Chlorophyta, Embryophyta, Zygnematophyceae and other streptophyte algae (Fig. 2a–d).

A total of 1,359 OGs were Zygnematophyceae specific, with enriched GO terms ‘phosphorylation’, ‘pyrophosphatase activity’, ‘transmembrane receptor protein serine/threonine kinase activity’, ‘cellular response to abscisic acid stimulus’ and ‘polysaccharide biosynthetic process’, speaking of an elaboration of the molecular chassis for signaling cascades and cell wall biosynthesis.

We inferred expanded and contracted gene families with CAFE^121^ using OGs from Orthofinder^119^. Among the 24 significantly contracted OGs are the light-harvesting complex (OG 43), ELIPs (OG 97; expanded in the *Zygnema* ancestor), RuBisCO small chain protein (OG 57), cell wall-related proteins such as expansins (OG 20), glycosyl transferases (OG 115) or glycoproteins (OG 182). The 11 expanded OGs feature lipases (OG 319), an uncharacterized protein with a methyltransferase domain (OG 637), a selenoprotein with a possible antioxidant activity (OG 1159), plant–microbe interaction proteins (OG 1170) and TFs (OG 777 and OG 1250).

We investigated protein domains and domain combinations that are gained, lost and significantly expanded in multicellular streptophyte algae. The top families present in filamentous *Zygnema* but absent in the three investigated unicellular Zygnematophyceae (Fig. 3b) include nidogen homology sequence (NIDO), which is present in animal glycoproteins but absent in land plants; Pro-kuma_activ, which corresponds to Peptidase S53, MBOAT_2, a domain in Wax synthase, involved in drought resistance; Alliinase, which is involved in auxin biosynthesis; Bac_rhodopsin, which is present in light-dependent ion pumps and sensor proteins; Glyco_hydro_26, which is present in β-mannanase; and the NB-ARC domain known from plant disease resistance gene families. For most of these families, gene loss in unicellular Zygnematophyceae is more likely than a gain in filamentous *Zygnema*, because they are present in algae outside of Phragmoplastophyta. Exceptions are the peptidase Pro-kuma_activ and the β-mannanase domain Glyco_hydro_26.

### CAZyme and gene family phylogenetic analysis

CAZyme families were identified with dbCAN2 (ref. ^122^) with default parameters (E-value <1^−10^ and coverage >0.35). Whenever needed, dbCAN2 was rerun by using more relaxed parameters. The experimentally characterized cell wall enzymes were manually curated from the literature (Supplementary Data 1 and Supplementary Table 1l). Reference genes were included into the phylogenies to infer the presence of orthologs across the 16 genomes and guide the split of large families into subfamilies. Phylogenetic trees were built by using FastTree initially, and for some selected families, RAxML^123^ and IQ-TREE^124^ were used to rebuild phylogenies to verify topologies.

### Co-expression network

The highest reciprocal rank co-expression network for *Z. circumcarinatum* (SAG 698-1b) was built from all RNA-seq samples (19 growth conditions), and the *Zygnema* database was established using the CoNekT framework^125^. The gene co-expression clusters were identified using the Heuristic Cluster Chiseling Algorithm with standard settings^126^.

We explored functional gene modules in *Z*. *circumcarinatum* SAG 698-1b by inferring gene co-expression networks from RNA-seq data of 19 growth conditions (see above). We obtained 406 clusters (modules) containing 17,881 out of the 20,030 annotated gene isoforms. Candidate genes were drawn from the literature and the set of expanded OGs.

### Statistics

To identify possible WGDs, Ks and 4dtv values were calculated for each genome. First, all paralog pairs were identified using the Reciprocal Best BLAST Hit (RBBH) method using protein sequences (E-value <1 × 10^−6^), following the method described by Bowman et al.^116^. RBBH paralog pairs were aligned with MAFFT^127^, and the corresponding nucleotide alignments were generated. Using RBBH alignments of paralog pairs, KaKs_Calculator2.0 (ref. ^128^) with the YN00 model and the calculate_4DTV_correction.pl script were run to calculate Ks and 4dtv values for each alignment, respectively. Values with Ks of 0 and 4dtv of 0 were filtered. The Ks and 4dtv distributions were fitted with a Gaussian kernel density model using the seaborn package. For the SAG 698-1b chromosome-level genome, MCscan^129^ was run to identify syntenic block regions with default parameters.

For the species phylogeny and divergence time analysis, a phylogenetic tree was built using RAxML v.8 (ref. ^123^) with the ‘-f a’ setting and the PROTGAMMAJTT model, and branch support with 100 pseudoreplicates of nonparametric bootstrap. The tree was rooted with Chlorophyta as outgroup. Using the above methodology, additional phylogenetic analyses were performed with (1) the four *Zygnema* strains and (2) the seven Zygnematophyceae genomes, to obtain a higher number of single-copy loci, 5,042 and 204, respectively (Supplementary Fig. 7). Divergence time estimation was carried out with MCMCTree implemented in the PAML package version 4.10.0j (ref. ^130^). The 493 low-copy OG protein sequence alignment was converted to the corresponding nucleotide alignment for MCMCTree, in which ten Markov chain Monte Carlo (MCMC) chains were run, each for 1,000,000 generations (Supplementary Table 1f). Three calibration were set in the reference tree according to Morris et al.^131^, on the nodes Viridiplantae (972.4 to 669.9 Ma), Streptophyta (890.9 to 629.1 Ma) and Embrophyta (514.8 to 473.5 Ma).

OG expansion and contraction were inferred with CAFE v.5 (ref. ^121^) using OGs inferred with Orthofinder^119^ v.2.4.0 and the previously inferred time-calibrated species phylogeny. CAFE v.5 was run with default settings (base) using the inferred OGs and a calibrated species phylogeny. Two independent runs arrived to the same final likelihood and lambda values. The first eight OGs (OG0–7) were excluded from the analysis due to too drastic size changes between branches that hampered likelihood calculation; excluded OGs were mostly exclusive to a single *Zygnema* or *Chara* genome and probably represented transposable elements, as judged by results of BLASTP against NR.

The gene co-expression clusters were identified using the Heuristic Cluster Chiseling Algorithm with standard settings^126^.

For phylogenies of gene families related to symbiosis, tree reconstruction was performed using IQ-TREE v2.1.2 (ref. ^132^) based on the Bayesian Information Criterion (BIC)-selected model determined by ModelFinder^133^; branch supports was estimated with 10,000 replicates each of both SH-aLRT^134^ and UltraFast bootstraps^135^. For the other phylogenies, homologs were aligned with MAFFT v7.453 using the L-INS-I approach^127^ and maximum likelihood phylogenies computed with IQ-TREE (v.1.5.5)^124^, with 100 nonparametric bootstrap pseudoreplicates and BIC-selected model with ModelFinder^133^.

### Reporting summary

Further information on research design is available in the Nature Portfolio Reporting Summary linked to this article.

## Online content

Any methods, additional references, Nature Portfolio reporting summaries, source data, extended data, supplementary information, acknowledgements, peer review information; details of author contributions and competing interests; and statements of data and code availability are available at 10.1038/s41588-024-01737-3.

### Supplementary information

Supplementary Information Overview of supplementary contents and Supplementary Figs. 1–21, materials and methods, and Text 1.

Reporting Summary

Peer Review File

Supplementary Table Supplementary Tables 1–4.

Supplementary Data

### Source data

Source Data Fig. 1 Values plotted in Fig. 1g.

Source Data Fig. 2 Values plotted in Fig. 2b,c,e,g.

Source Data Fig. 3 Values plotted in Fig. 3i.

Source Data Fig. 4 Values plotted in Fig. 4b,c,e.

Source Data Fig. 5 Values plotted in Fig. 5a,c,d.

Source Data Fig. 6 Values plotted in Fig. 6a.

## Data Availability

The four *Zygnema* genomes, raw DNA reads and rRNA-depleted RNA-seq of SAG 698-1b can be accessed through NCBI BioProject PRJNA917633. The raw DNA read data of UTEX 1559 and UTEX 1560 sequenced by the Joint Genome Institute can be accessed through BioProjects PRJNA566554 and PRJNA519006, respectively. RNA-seq data of UTEX 1559 can be accessed through BioProject PRJNA524229. Poly-A enriched RNA-seq data of SAG 698-1b can be accessed through BioProject PRJNA890248 and the Sequence Read Archive (SRA) under the accession SRR21891679 to SRR21891705. *Zygnema* genomes are also available through the PhycoCosm portal^136^ (https://phycocosm.jgi.doe.gov/SAG698-1a (ref. ^137^), https://phycocosm.jgi.doe.gov/SAG698-1b (ref. ^138^), https://phycocosm.jgi.doe.gov/UTEX1559 (ref. ^139^) and https://phycocosm.jgi.doe.gov/UTEX1560 (ref. ^140^)). Data files are available via Figshare at 10.6084/m9.figshare.22568197 (ref. ^141^) and via Mendeley at 10.17632/gk965cbjp9.1 (ref. ^142^). Source data are provided with this paper.

## Code availability

No original code was used; all computational analyses were performed with published tools and are cited in Methods and Supplementary Materials and Methods.
