# Supplementary material for: Genomes of multicellular algal sisters to land plants illuminate signaling network evolution
Source: Nat Genet. 2024 May 1;56(5):1018–31. doi: 10.1038/s41588-024-01737-3 (PMC11096116; doi:10.1038/s41588-024-01737-3)
Supplement: Supplementary file 1 — Overview of supplementary contents and Supplementary Figs. 1–21, materials and methods, and Text 1. [file 41588_2024_1737_MOESM1_ESM.pdf]

nature genetics

Article

https://doi.org/10.1038/s41588-024-01737-3

Genomes of multicellular algal sisters to land plants illuminate signaling network evolution

In the format provided by the authors and unedited

SUPPLEMENTARY MATERIAL

Supplementary Data 1. CAZyme gene family analysis.

Supplementary Materials and Methods. Detailed account of materials and methods.

Supplementary Text 1. History of *Zygnema* strains.
Supplementary Text 2. Domain combinations.
Supplementary Text 3. Cell wall complexity and CAZymes.
Supplementary Text 4. Gene co-expression modules.
Supplementary Text 5. Phytohormone biosynthesis and signaling pathways.

Supplementary Table 1. Data on genome sequencing, annotation, divergence times, *Closterium* sex hormone homologs and cell wall-related enzymes. (a) Genome and transcriptome sequence data, (b) Genome size estimation, (c) Summary of genome assembly, (d) Completeness, evaluation of four *Zygnema* genomes, (e) BUSCO evaluation of other genomes, (f) Divergence time estimation of the representative genomes, (g) Gene annotation of SAG 698-1a_XF mitogenome, (h) Comparison of introns and exons between mitogenomes of 698-1a_XF and SAG 698-1b, (i) Summary of gene content in 16 representative genomes, (j) Repeat elements annotation, (k) *Closterium* sex hormone protein search, (l) Cell wall-related enzyme subfamilies.
Supplementary Table 2. Domain combinations in the Zygnematophyceae and Embryophyta ancestor.
Supplementary Table 3. (a) Orthogroup expansions and contractions (full CAFE results), (b) Significant orthogroup expansions and contractions for the ancestors of Embrophyta + Zygematophyceae, Zygnematophyceae, and *Zygnema*, (c) Results of TAPscan, (d) Identification of cell-division genes in *Zygnema* and across the green lineage.
Supplementary Table 4. Domain changes associated with multicellularity. (a) Streptophyte algal genomes domain statistic, (b) Streptophyte algal genomes domains shared, (c) Streptophyte algal genomes domains only in multicellular organisms, (d) Streptophyte algal genomes domains only in unicellular organisms, (e) Streptophyte algal genomes all shared domain combinations, (f) Streptophyte algal genomes domain combinations only in multicellular organisms, (g) Streptophyte algal genomes domain combinations only in unicellular organisms, (h) Chloroplastida genomes domain statistic, (i) Chloroplastida genomes domains shared, (l) Chloroplastida genomes domains only in multicellular organisms, (k) Chloroplastida genomes domains only in unicellular organisms, (l) Chloroplastida genomes all shared domain combinations, (m) Chloroplastida genomes domain combinations only in multicellular organisms, (n) Chloroplastida genomes domain combinations only in unicellular organisms.

Supplementary Figure 1. Light micrographs of *Zygnema circumcarinatum* SAG 698-1b fixed and stained with aceto-carmine and transmission electron micrographs of SAG 698-1a and 698-1b. A minimum of 15 algal filaments each for two biological replicates (cell cultures) were analyzed.
Supplementary Figure 2. K-mer plots for genome size estimation.
Supplementary Figure 3. Gene annotation of *Zygnema circumcarinatum* SAG 698-1b mitogenome.
Supplementary Figure 4. Mauve alignment of mitogenomes of UTEX 1559 and SAG 698-1b.
Supplementary Figure 5. Gene annotation of *Zygnema* cf. *cylindricum* SAG 698-1a_XF mitogenome.
Supplementary Figure 6. Polyploidy analysis of SAG 698-1b chromosome-level genome and comparison with *Physcomitrium patens*.
Supplementary Figure 7. Maximum likelihood trees inferred from orthogroups obtained from Zygnematophyceae and *Zygnema* genomes.
Supplementary Figure 8. Comparisons of the three *Z. circumcarinatum* chromosome-level genomes.
Supplementary Figure 9. Phylogeny of the O-FucT family.
Supplementary Figure 10. Phylogenies of genes salient to the production of phenylpropanoid-derived specialized metabolites and PP2C homologs.
Supplementary Figure 11. Maximum Likelihood phylogeny of phytochrome homologs.
Supplementary Figure 12. Maximum likelihood phylogenies of GRAS homologs and the transducin / WD-40 orthogroup and domain architecture.
Supplementary Figure 13. Maximum likelihood phylogeny of CASTOR and POLLUX homologs.
Supplementary Figure 14. Maximum likelihood phylogeny of CYCLOPS homologs.
Supplementary Figure 15. Maximum likelihood phylogenies of CCAMK homologs.
Supplementary Figure 16. Maximum Likelihood phylogeny of MADS-domain proteins.
Supplementary Figure 17. Identification of ABA in SAG 698-1b.
Supplementary Figure 18. Phylogeny of CCD proteins.
Supplementary Figure 19. Phylogeny of CCD7 homologs.
Supplementary Figure 20. Phylogeny of CCD8 homologs.
Supplementary Figure 21. Selected gene co-expression modules.

Supplementary Materials and Methods

Resource table

REAGENT or RESOURCE	SOURCE	IDENTIFIER
**Critical Commercial Assays**		
DNeasy Plant Pro Kit	QIAGEN, Hilden, Germany	Cat# 69204
Dneasy PowerClean Cleanup Kit	QIAGEN, Hilden, Germany	Cat# 12877
Nanopore Ligation Sequencing Kit	Oxford Nanopore, Oxford, UK	Cat# SQK-LSK109
DNAse I	Thermo Fisher, Waltham, MA, USA	
NEB mRNA stranded Library preparation kit	New England Biolabs, Beverly, MA, USA	
Trizol	Thermo Fisher, Waltham, MA, USA	
**Deposited Data**		
Amborella trichopoda genome	Amborella genome project, 2013 [1]	https://phytozome.jgi.doe.gov/pz/portal.html#!info?alias=Org_Atrichopoda
Arabidopsis thaliana genome TAIR V10	TAIR	http://www.arabidopsis.org
Azolla filiculoides genome	Li et al., 2018 [2]	https://www.fernbase.org
Bathycococcus prasinos genome	Moreau et al., 2012 [3]	https://phycocosm.jgi.doe.gov/Batpra1/Batpra1.info.html
Brachypodium distachyon	The International *Brachypodium* Initiative 2010 [4]	https://phytozome-next.jgi.doe.gov/info/Bdistachyon_v3_1
Chara braunii S276 genome	Nishiyama et al., 2018 [5]	https://bioinformatics.psb.ugent.be/orcae/overview/Chbra
Chlamydomonas reinhardtii genome v5.5	Merchant et al., 2007 [6]; Blaby et al., 2014 [7]	https://phytozome.jgi.doe.gov/pz/portal.html#!info?alias=Org_Creinhardtii
Chlorokybus atmophyticus genome	Wang et al., 2020 [8]	https://www.ncbi.nlm.nih.gov/assembly/GCA_009103225.1/
Coccomyxa subellipsoidea genome v2.0	Blanc et al., 2012 [9]	https://phytozome.jgi.doe.gov/pz/portal.html#!info?alias=Org_CsubellipsoideaC_169
Coleochaete scutata SAG 50.90 transcriptome	de Vries et al., 2018 [10]	https://www.ncbi.nlm.nih.gov/Traces/wgs/wgsviewer.cgi?val=GFXZ00000000
Supplementary Data 1, alignments	This study	https://data.mendeley.com/datasets/gk965cbjp9/draft?a=0c754a46-0efb-4133-bc1a-befc286d27da

Klebsormidium nitens genome v1.1	Hori et al., 2014 [11]	http://www.plantmorphogenesis.bio.titech.ac.jp/~algae_genome_project/klebsormidium/
Klebsormidium nitens SAG2307 transcriptome	de Vries et al., 2018 [10]	https://www.ncbi.nlm.nih.gov/Traces/wgs/wgsviewer.cgi?val=GFXY00000000
Marchantia polymorpha genome v3.1	Bowman et al., 2017 [12]	https://phytozome.jgi.doe.gov/pz/portal.html#!info?alias=Org_Mpolymorpha
Mesostigma viride genome	Wang et al., 2020 [8]	https://www.ncbi.nlm.nih.gov/assembly/GCA_009103195.1/
Mesotaenium endlicherianu genome	Cheng et al., 2019 [13]	https://www.ncbi.nlm.nih.gov/assembly/GCA_009602735.1/
Oryza sativa Nipponbare genome v7.0	Kawahara et al., 2013 [14]	https://phytozome.jgi.doe.gov/pz/portal.html#!info?alias=Org_Osativa
Ostreococcus lucimarinus genome v2.0	Palenik et al., 2007 [15]	https://phytozome.jgi.doe.gov/pz/portal.html#!info?alias=Org_Olucimarinus
Penium margaritaceum genome	Jiao et al., 2020 [16]	http://bioinfo.bti.cornell.edu/cgi-bin/Penium/home.cgi
Physcomitrium patens genome v3.3	Lang et al., 2018 [17]	https://phytozome.jgi.doe.gov/pz/portal.html#!info?alias=Org_Ppatens
Picea abies genome	Nystedt et al., 2013 [18]	https://plantgenie.org/FTP?dir=Data%2FconGenIE%2Fpicea_abies%2Fv1.0
Salvinia cucullata genome	Li et al., 2018 [2]	https://www.fernbase.org
Selaginella moellendorffii genome	Banks et al., 2011 [19]	https://phytozome-next.jgi.doe.gov/info/Smoellendorffii_v1_0
Sphagnum fallax v0.5 genome	Obtained from Phytozome with permission	https://phytozome-next.jgi.doe.gov/info/Sfallax_v0_5
Spirogloea muscicola genome	Cheng et al., 2019 [13]	https://www.ncbi.nlm.nih.gov/assembly/GCA_009602725.1
Ulva mutabilis genome	De Clerck et al., 2018 [20]	https://bioinformatics.psb.ugent.be/orcae/overview/Ulvmu
Volvox carteri genome v2.1	Prochnik et al., 2010 [21]	https://phytozome.jgi.doe.gov/pz/portal.html#!info?alias=Org_Vcarteri
Zygnema cf. cylindricum SAG 698-1a transcriptome	de Vries et al., 2018 [10]	https://www.ncbi.nlm.nih.gov/bioproject/PRJNA399177
Zygnema circumcarinatum UTEX 1559 transcriptome	Fitzek et al., 2019 [22]	https://www.ncbi.nlm.nih.gov/sra/?term=SRX5449751
Zygnema cf. Cylindricum SAG 698-1a_XF genome	This study	https://phycocosm.jgi.doe.gov/SAG698-1a

Zygnema circumcarinatum SAG 698-1b genome	This study	https://phycocosm.jgi.doe.gov/SAG698-1b
Zygnema circumcarinatum SAG 1559 genome	This study	https://phycocosm.jgi.doe.gov/UTEX1559
Zygnema circumcarinatum SAG 1560 genome	This study	https://phycocosm.jgi.doe.gov/UTEX1560
Zygnema circumcarinatum SAG 698-1b co-expression networks	This study	https://zygnema.sbs.ntu.edu.sg/
**Organisms and strains**		
Zygnema circumcarinatum SAG 698-1b	Bohemia, ditch at meadow Poselteich (Poselský rybnik) near Hirschberg (Doksy), 50.552702 / 14.669362 (800m), isolated by V. Czurda in 1929. Obtained from the Culture Collection of Algae at the University of Göttingen, Germany (SAG)	Maintained at the Culture Collection of Algae at the University of Göttingen, Germany (SAG) (https://sagdb.uni-goettingen.de/detailedList.php?str_number=698-1b)
Zygnema circumcarinatum UTEX 1559	Spontaneous mutant of IUCC 42 (UTEX B 42) with increased size and numbers of chloroplasts per cell, generated by H. Gauch, deposited in 1966. Obtained from the Culture Collection of Algae at University of Texas Austin	Maintained at the Culture Collection of Algae at the University of Texas at Austin (UTEX). (https://utex.org/products/utex-1559?variant=30991929671770)
Zygnema circumcarinatum UTEX 1560	Spontaneous mutant of IUCC 43 (UTEX B 43) with increased size and numbers of chloroplasts per cell, generated by H. Gauch, deposited in 1966. Obtained from the Culture Collection of Algae at University of Texas Austin	Maintained at the Culture Collection of Algae at the University of Texas at Austin (UTEX). (https://utex.org/products/utex-1560?variant=30991202058330)
Zygnema cf. *cylindricum* SAG 698-1a	Bohemia, ditch at meadow Poselteich (Poselský rybnik) near Hirschberg (Doksy),	Maintained at the Culture Collection of Algae at the University of Göttingen, Germany (SAG) (https://sagdb.uni-goettingen.de/detailedList.php?str_number=698-1a)

	50.552702 / 14.669362 (800m), isolated by V. Czurda in 1929. Obtained from the Culture Collection of Algae at the University of Göttingen, Germany (SAG)	
Zygnema cf. *cylindricum* SAG 698-1a_XF	Re-isolate based on picking one filament from SAG 698-1a (see above) and establishment of a new culture	Maintained at the Culture Collection of Algae at the University of Göttingen, Germany (SAG)
**Software, Algorithms, and Bioinformatic Tools**		
ARWEN v1.2.3	Laslett and Canbäck, 2008 [23]	http://www.mybiosoftware.com/arwen-1-2-3-trna-detection-in-metazoan-mitochondrial-sequences.html
Bowtie2	Langmead and Salzberg, 2012 [24]	http://bowtie-bio.sourceforge.net/bowtie2/index.shtml
BUSCO v.5.2.2	Seppey et al. 2019 [25]	https://busco.ezlab.org
CAFE v5.0	Mendes et al., 2021 [26]	https://github.com/hahnlab/CAFE
canu	Koren et al., 2017 [27]	https://github.com/marbl/canu
dbCAN2	Zhang et al., 2018 [28]	https://github.com/linnabrown/run_dbcan
FASTQC	Babraham Institute [29]	www.bioinformatics.babraham.ac.uk/projects/fastqc
FastTree 2.1	Price et al., 2009 [30]	http://www.microbesonline.org/fasttree/
Gblocks v0.91b	Castresana, 2000 [31]	http://molevol.cmima.csic.es/castresana/Gblocks_server.html
Geneious	Kearse et al., 2012 [32]	https://www.geneious.com/
Geqseq	Tillich et al., 2017 [33]	https://chlorobox.mpimp-golm.mpg.de/geseq.html
GenomeScope	Vurture et al, 2017 [34]	http://qb.cshl.edu/genomescope/
HISAT2	Kim et al., 2019 [35]	https://daehwankimlab.github.io/hisat2/
hmmer	Finn et al., 2011 [36]	http://hmmer.org/
IQ-Tree v1.5.5	Nguyen et al., 2015 [37]	http://www.iqtree.org
iTOL v4.2.3	Letunic and Bork, 2021 [38]	https://itol.embl.de/
kakscalculator2	Wang et al., 2010 [39]	https://github.com/hahnlab/CAFE
kmergenie	Chikhi and Medvedev, 2014 [40]	http://kmergenie.bx.psu.edu/
MAFFT v7.3.10	Katoh and Standley, 2013 [41]	https://mafft.cbrc.jp/alignment/software/source.html
MAKER-P v3.01.03	Campbell et al., 2014 [42]	http://www.yandell-lab.org/software/maker-p.html
MCScanX	Wang et al., 2012 [43]	https://github.com/wyp1125/MCScanX
MITE-tracker	Crescente et al., 2018 [44]	https://github.com/INTABiotechMJ/MITE-Tracker
minimap2	Li, 2018 [45]	https://github.com/lh3/minimap2
MUMmer v4.0.0	Marçais et al., 2018 [46]	https://github.com/mummer4/mummer

NOVOPlasty 3.8.2	Dierckxsens et al., 2017 [47]	https://github.com/ndierckx/NOVOPlasty
OGDRAW	Greiner et al., 2019 [48]	https://chlorobox.mpimp-golm.mpg.de/OGDraw.html
Orthofinder v2.5.2	Emms and Kelly, 2019 [49]	https://github.com/davidemms/OrthoFinder
PAML v4.10.0j	Yang, 2007 [50]	http://abacus.gene.ucl.ac.uk/software/paml.html
PASA v2.4.1	Haas et al., 2003 [51]	https://github.com/PASApipeline/PASApipeline
Pilon	Walker et al., 2014 [52]	https://github.com/broadinstitute/pilon
Phytools	Revell 2012 [53]	https://cran.r-project.org/web/packages/phytools/index.html
Platanus_allele	Kajitani et al., 2019 [54]	http://platanus.bio.titech.ac.jp
Posterior Mean Site Frequency Profiles	Wang et al., 2018 [55]	Implemented in IQ-Tree http://www.iqtree.org
Spades	Antipov et al., 2016 [56]	https://github.com/ablab/spades
Racon	Vaser et al., 2017 [57]	https://gith--ub.com/isovic/racon
RAxML v8	Stamatakis, 2014 [58]	https://cme.hits.org/exelixis/web/software/raxml/index.html
RepeatMasker 4.0.9	Bergman and Quesneville, 2007 [59]	http://www.repeatmasker.org/
RepeatModeler v2.0.1	Robert Hubley & Arian Smit	https://www.repeatmasker.org/RepeatModeler/
Re-routing method according to Yang 1995	Yang et al., (1995) [60]	n/a
Rfam 13.0	Kalvari et al., 2018 [61]	https://rfam.org/
StringTie	Pertea et al., 2016 [62]	https://ccb.jhu.edu/software/stringtie/
Transcdecoder v.5.5.0	Brian J. Haas	https://github.com/TransDecoder/TransDecoder/releases
Trimal v1.4.rev15	Capella-Gutierrez et al. (2009) [63]	http://trimal.cgenomics.org
Trimmomatic v0.36	Bolger et al., 2014 [64]	http://www.usadellab.org/cms/?page=trimmomatic
Trinity v2.9.0	Grabherr et al., 2011 [65]	https://github.com/trinityrnaseq/trinityrnaseq
tRNAscan-SE v2.0	Lowe and Chan, 2016 [66]	http://lowelab.ucsc.edu/tRNAscan-SE/
WENGAN v0.2	Di Genova et al., 2021[67]	https://github.com/adigenova/wengan
wtdbg2	Ruan and Li, 2020 [68]	https://github.com/ruanjue/wtdbg2

Algal strains

Z. circumcarinatum SAG 698-1b and *Z. cf. cylindricum* SAG 698-1a were obtained from the Culture Collection of Algae at Göttingen University (SAG) (https://sagdb.uni-goettingen.de). *Z. circumcarinatum* UTEX 1559 and UTEX 1560 were obtained from the UTEX Culture Collection of Algae at UT-Austin (https://utex.org/). From *Z. cf. cylindricum* SAG 698-1a, a single filament was picked and a new culture was established, coined *Z. cf. cylindricum* SAG 698-1a_XF; *Z. cf. cylindricum* SAG 698-1a_XF was used for genome sequencing. The algae were cultured with Bold's basal medium (BBM) or modified Bold's basal medium (MBBM), supplemented with 0.02% L-arginine, 0.1% peptone and 0.5% sucrose [69]. The filaments were grown for two or three weeks on a rotary shaker platform (Fermentation Design, 125 rpm) in Plant Growth Chambers (Conviron PGR15) with conditions: 16:8 of light: dark cycle, 20°C, ~50 µmol of quanta m-2 ·s-1 [69]. Some cultures were also maintained on 1% agar or liquid MBBM in the same conditions.

Chromosome counting of *Z. cricumcarinatum* SAG 698-1b

Z. circumcarinatum strain SAG 698-1b was grown in liquid Bold's basal medium (BBM) in axenic culture under controlled conditions (20°C, ~50 µmol photons m-2 s-1). Exposition to a 10:14 h light-dark cycle led to synchronization of the cell cycle. The period where most cell divisions took place was from the last hour of the light cycle to the first 5 h of the dark cycle, as previously reported cell division occurs almost solely in the dark phase in *Zygnema*[70]. After a two-week incubation, algal filaments were harvested and pretreated for 1 h at RT (Room Temperature), followed by 1 h at 4°C in 2 mM 8-hydroxychinolin (Roth, Karlsruhe, Germany) in darkness, resulting in depolymerization of microtubules and an increased condensation of chromosomes. Cells were fixed in 1:3 glacial acetic acid:ethanol solution (Carnoy's fluid) for 12 h until all chlorophyll was extracted. Fixed cells were stored in 70% ethanol at -20°C until staining, which was performed by boiling in acetocarmine (99% acetic acid, 1% carmine; Morphisto, Offenbach, Germany) for 5 min. To maximize the visualization of the chromosomes the filaments on the prepared slides were slightly crushed, and stacks of 50 to 100 images per filaments with well stained chromosomes were taken at a Zeiss Axiovert 200 M microscope (100x, 1.3 NA, objective lens) with a Zeiss Axiocam HRm Rev.3 camera (Carl Zeiss, Jena, Germany). Stacked models of 100-150 images were rendered by the software Helicon Focus (HeliconSoft Ltd., Kharkiv, Ukraine). Final count of chromosomes was done with ImageJ (Fiji, open source). A minimum of 10 cells each from three independent cell cultures were analyzed.

DNA extraction

Detailed protocols have been published elsewhere[22,69,71]. Briefly, algae were grown for two weeks and harvested using a vacuum filtration with Whatman #2 paper (GE Healthcare 47 mm), washed with distilled water (three times), frozen in liquid nitrogen and stored in -80°C. Frozen algae were lyophilized overnight and total genomic DNA was extracted with DNeasy PowerPlant Pro Kit (Qiagen, Germany) using the following workflow: lyophilized algae were first chopped with a spatula into fine powder and mixed well with beads solution and RNase A. The mixture was homogenized on a vortex adapter at maximum speed for 5 min. Then the DNeasy PowerPlant Pro Kit protocol was followed and the extracted DNA was further purified with DNeasy PowerClean CleanUp Kit (Qiagen, Germany).

Before DNA extraction, to reduce chloroplast and mitochondria derived DNA (up to > 60% of total DNA), a modified nucleus isolation method was used[72]. Briefly, fresh algae tissues were grinded into fine powder in liquid nitrogen with precooled mortar and pestle. After that, the powder was transferred into a beaker containing nucleus isolation buffer. This mixture was homogenized well on ice, and then were vacuum filtered with two layers of miracloth (Thermo Fisher Scientific, USA). The remained nuclei were pelleted by centrifugation at 800 g and 4°C for 10 min, and extracted with DNeasy PowerPlant Pro Kit (Qiagen, Germany). After DNA extraction, quality and quantity of purified DNA was evaluated by using 1% agarose gel electrophoresis, NanoDrop 2000/2000c Spectrophotometers, and Qubit 3.0 Fluorometer (Thermo Fisher Scientific).

RNA extraction

Frozen algae were lyophilized overnight for RNA extraction with a modified CTAB method described in Chang et al.[73] and Bekesiova et al.[74]. Specifically, the tissue was chopped with spatula into fine powder, and then 500 µl of CTAB buffer (2% CTAB, 100 mM Tris-HCl pH 8.0, 25 mM EDTA, 2 M NaCl, 2% Polyvinylpyrrolidone, 1% β-mercaptoethanol) was added and mixed well. The mixture was incubated in heating block at 65°C for 15 min. After the tubes cooled down, the solution was extracted with Chloroform: Isoamyl alcohol 24:1 twice. The supernatant was precipitated with 0.3 volume of 10 M LiCl that was incubated in -20°C for 30 min. The pellet was washed with 75% ethanol twice and vacuum dried for 15 min. RNA was resuspended in 50 µl of 0.1% DEPC (diethylpyrocarbonate) water. RNA samples were treated with RNase-Free DNase I (Promega) at 37°C for 30 min to remove any DNA residue. Quality and quantity of purified RNA was evaluated by using 1% agarose gel electrophoresis, NanoDrop 2000/2000c Spectrophotometers, Qubit 3.0 Fluorometer (Thermo Fisher Scientific) and RNA Integrity Number (RIN) (Agilent).

Transcriptome assembly

Raw RNA-seq reads (Table S1A) were quality checked with FastQC v.0.11.9 (http://www.bioinformatics.babraham.ac.uk/projects/fastqc/)[29], trimmed with TrimGalore (https://github.com/FelixKrueger/TrimGalore), and were inspected again with FastQC. All reads were combined, and *de novo* assembled with Trinity version 2.9.0 [65,75].

K-mer frequency analysis

The trimmed DNA Illumina reads were filtered out with BLASTP using plastomes and mitogenomes from *Zygnema* as references. Remaining (putatively nuclear) were used to predict the best k-mer size by kmergenie (http://kmergenie.bx.psu.edu/)[40]. The histogram of the best k-mer was then uploaded to GenomeScope for viewing the genome plot (http://qb.cshl.edu/genomescope/)[34] (Supplementary Table 1b and Supplementary Figure 2).

Comparison of *Z. circumcarinatum* genomes (SAG 698-1b, UTEX 1559, UTEX 1560)

Two approaches were used to compare the three genomes (Supplementary Figure 8). The first approach was based on the whole genome alignment (WGA) by using MUMMER. The parameters "--maxmatch -c 90 -l 40" were set to align the three genomes and then "-i 90 -l 1000" were set to filter out the smaller fragments. The second approach focused on the gene content comparisons. Orthofinder was used to obtain ortholog groups (orthogroups) from genomes' annotated proteins. Orthofinder results led to a Venn diagram with unique genes (orthogroups with genes from only one genome), cloud genes (orthogroups with genes from only 2 genomes), and core genes (orthogroups with genes from all three genomes), which collectively form the pan-genome. Orthofinder could have failed to detect homology between very rapidly evolved orthologous genes, which leads to an under-estimation of core genes. Also, gene prediction may have missed genes in one genome but found them in other genomes. To address these issues, the raw DNA reads of each genome were mapped to the unique genes and cloud genes using BWA. This step was able to push more unique genes and cloud genes into core genes or push some unique genes into cloud genes. The following criteria were used to determine if an orthogroup needed to be re-assigned: (i) the number of reads and coverage calculated by bedtools are >10 and >0.8 for a gene, respectively, and (ii) >60% coverage of genes in the orthogroup find sequencing reads from the other genomes. After this step the final Venn diagram was made (Supplementary Figure 8f), showing the counts of the final core genes, cloud genes, and unique genes.

Dataset for species phylogeny and divergence time analysis

Sixteen representative genomes were selected, including two chlorophytes (*Volvox carteri, Chlamydomonas reinhardtii*), seven Zygnematophyceae (*Zygnema circumcarinatum* SAG 698-1b, UTEX 1559, UTEX 1560, *Z.* cf. *cylindricum* SAG 698-1a_XF, *Mesotaenium endlicherianum*, *Penium margaritaceum*, *Spirogloea muscicola*), four additional streptophyte algae (*Chara braunii, Klebsormidium nitens, Chlorokybus atmophyticus, Mesostigma viride*), two bryophytes (*Marchantia polymorpha,*

Physcomitrium patens) and a vascular plant (*Arabidopsis thaliana*). Orthogroups were generated by OrthoFinder version 2.5.2[49] and 493 low-copy orthogroups containing ≤ 3 gene copies per genome were aligned with MAFFT v7.310 [41]. Gene alignments were concatenated and gaps were removed by Gblocks version 0.91b[31].

Phytohormones

Proteins involved in phytohormone biosynthesis and signaling were identified by BLASTP against annotated proteomes (e-value<1e-6) using *Arabidopsis* genes as queries. For genes with ubiquitous domains (e.g. CIPK, CPK3, SNRK2, CDG1, BAK1), hits were filtered by requiring BLASTP coverage ≥50% of the query. Significant hits were then aligned (MAFFT auto) and maximum likelihood gene trees were inferred in IQ-Tree using best-fit models and 1000 replicates of SH-like aLRT branch support ('-m TEST -msub nuclear -alrt 1000'). The final sets of homologs were identified by visually inspecting gene trees and identifying the most taxonomically diverse clade (with high support of SH-aLRT>0.85) that included the characterized *Arabidopsis* proteins. A bubble plot was generated with ggplot2 in R.

Constructing the co-expression network and establishing the Zygnema database

The Highest Reciprocal Rank (HRR) co-expression network for *Z. circumcarinatum* SAG 698-1b was built from all samples (19 growth conditions) and the Zygnema database was established using the CoNekT framework[76]. The gene co-expression clusters were identified using the Heuristic Cluster Chiseling Algorithm (HCCA) with standard settings[77].

Screening for symbiotic genes and phylogenetic analysis

Symbiotic genes were screened against a database of 211 plant species across Viridiplantae lineage using proteins of the model plant *Medicago truncatula* as queries in BLASTP v2.11.0+[78] searches with default parameters and an e-value < 1e-10. Initial alignments of all identified homologs was performed using the DECIPHER package [79] in R v4.1.2 (R Core Team). Positions with >60% gaps were removed with trimAl v1.4[63] and a phylogenetic analysis was performed with FastTree v2.1.11[30]. Clades corresponding to *M. truncatula* orthologs queries were extracted and a second phylogeny was performed. Proteins were aligned using MUSCLE v3.8.1551[80] with default parameters and alignments cleaned as described above. Tree reconstruction was performed using IQ-Tree v2.1.2[81] based on BIC-selected model determined by ModelFinder[82]. Branch supports was estimated with 10,000 replicates each of both SH-aLRT[83] and UltraFast bootstraps[84]. Trees were visualized and annotated with iTOL v6[38]. For the GRAS family, a subset of 42 species representing the main lineages of Viridiplantae was selected and GRAS putative proteins screened using the HMMSEARCH program with default parameters and the PFAM domain PF03514 from HMMER3.3[85] package. Phylogenetic analysis was then conducted as described above.

Screening for CCD homologs and phylogenetic analysis

We used annotated proteins from 21 land plant genomes (*Amborella trichopoda, Anthoceros agrestis, Anthoceros punctatus, Arabidopsis lyrata, Arabidopsis thaliana, Azolla filiculoides, Brachypodium distachyon, Brassica rapa, Lotus japonicus, Marchantia polymorpha, Medicago truncatula, Oryza sativa, Physcomitrium patens, Picea abies, Pisum sativum, Salvinia cucullata, Selaginella moellendorffii, Sphagnum fallax, Spinacia oleracea, Gnetum montanum, Crocus sativus*), 7 streptophyte algal genomes (*Spirogloea muscicola, Penium_margaritaceum, Mesotaenium endlicherianum, Mesostigma viride, Klebsormidium nitens, Chlorokybus melkonianii, Chara braunii, Zygnema circumcarinatum*), 6 chlorophyte genomes (*Ulva mutabilis, Ostreococcus lucimarinus, Micromonas pusilla, Micromonas sp., Chlamydomonas reinhardtii, Coccomyxa subellipsoidea, Chlorella_variabilis*), 5 cyanobacterial genomes (*Trichormus azollae, Oscillatoria acuminata, Nostoc punctiforme, Gloeomargarita lithophora, Fischerella thermalis*), as well as the transcriptome of *Coleochaete scutata*[10]. The representative *A. thaliana* protein was used as query for BLASTP searches against the above annotated proteins (E-value < 1e-5). Homologs were aligned with MAFFTv7.453 L-INS-I approach[41] and maximum likelihood phylogenies computed with IQ-Tree v.1.5.5[37], with 100 bootstrap replicates and BIC-selected model (WAG+R9) with ModelFinder[82].

Functional residue analyses were done based on published structural analysis[86], and the alignments were plotted with ETE3[87].

Phylogeny of MADS-box genes

MADS-domain proteins were identified by Hidden Markov Model (HMM) searches[88] on annotated protein collections. MADS-domain sequences of land plants and opisthokonts were taken from previous publications[89,90]. MADS domain proteins of other streptophyte algae were obtained from the corresponding genome annotations and transcriptomic data (One Thousand Plant Transcriptomes Initiative, 2019)[91]. Additional MADS-domain proteins of Zygnematophyceae were identified by BLAST against transcriptome data available at NCBI's sequence read archive (SRA)[92]. MADS-domain-protein sequences were aligned using MAFFTv7.310[41] with default options. Sequences with bad fit to the MADS domain were excluded and the remaining sequences realigned, and trimmed using trimAl[63] with options "-gt .9 -st .0001". A maximum likelihood phylogeny was reconstructed using RAxMLv8.2.12[58] on the CIPRES Science Gateway[93].

LC-MS/MS analysis of abscisic acid

Abscisic acid (ABA) was determined in *Physcomitrium patens* samples using the LC-MS/MS system which consisted of Nexera X2 UPLC (Shimadzu) coupled QTRAP 6500+ mass spectrometer (Sciex). Chromatographic separations were carried out using the Acclaim RSLC C18 column (150×2.1 mm, 2.2µm, Thermo Scientific) employing acetonitrile/water+0.1% acetic acid linear gradient. The mass spectrometer was operated in negative ESI mode. Data was acquired in MRM mode using following transitions: 1) ABA 263.2->153.1 (-14 eV), 263.2->219.1 (-18 eV); 2) ABA -D6 (IS) 269.2->159.1 (-14 eV), 269.2->225.1 (-18 eV); declustering potential was -45 V. Freeze-dried moss samples were ground using the metal beads in homogenizer (Bioprep-24) to a fine powder. Accurately weighted (about 20 mg) samples were spiked with isotopically labeled ABA -D6 (total added amount was 2 ng) and extracted with 1.5 ml acetonitrile/water (1:1) solution acidified with 0.1% formic acid. Extraction was assisted by sonication (Elma S 40 H, 15 min, two cycles) and solution was left overnight for completion of extraction. Liquid was filtered through 0.2 µm regenerated cellulose membrane filters, evaporated to dryness upon a stream of dry nitrogen and redissolved in 100 µl extraction solution.

Supplementary Text 1: History of *Zygnema* strains

A *Zygnema* sample was collected by V. Czurda in 1929 from a ditch at meadow Poselteich (Poselský rybnik) near Hirschberg (Doksy), Bohemia. From the original samples, a mating type (mt) + and mt – were created, which gave rise to Pringsheim's Prague strains 208 and 209, respectively. Pringsheim's strain 208 (mt +) was then transferred to the Culture Collection of Algae and Protozoa (CCAP), Scotland under CCAP698/1A and from there to the Culture Collection of Algae at Göttingen University (SAG) under accession SAG 698-1a and to the Culture Collection of Algae at UT-Austin (UTEX) under accession UTEX 42 (=former IUCC 42). A "spontaneous mutant with increased size and numbers of chloroplasts per cell" in UTEX 42 was described by Gauch (1966)[94], deposited under accession UTEX 1559. Pringsheim's strain 209 (mt -) was deposited as CCAP 689/1B and from there into SAG 698-1b and UTEX 43. Again, Gauch (1966)[94] identified a "spontaneous mutant of UTEX 43 with increased size and numbers of chloroplasts per cell", which was deposited under accession UTEX 1560. Therefore, all four *Zygnema* isolated should derive from the same field sample obtained by V. Czurda in 1929. This contrast with our findings that SAG 698-1a is in fact *Zygnema* cf. *cylindricum*, which points to an event of contamination or mislabeling in algal collections at some point in the last ~100 years. The known strain history, however, explains the closer affinity of *Z. circumcarinatum* UTEX 1559 and SAG 698-1b compared to UTEX 1560.

Supplementary Text 2: Domain combinations

We identified which Pfam domains are lost in the four filamentous *Zygnema* genomes compared to other streptophyte algae (**Fig. 3a**). The top families include Pectate_lyase_4, Pectinesterase, Glyoxal_oxid_N, DUF1929, DUF642, Glyco_hydro_35 families. All these are related to pectin degradation/modification, which is critical for plant cell wall and extracellular matrix (ECM) remodeling. For example, AT1G19900 (RUBY) with a Glyoxal_oxid_N domain is responsible for the oxidation of galactose in the side chains of pectins to strengthen the cell-cell adhesion[95]. And some of these cell wall degradation/remodeling families are significantly expanded in unicellular Zygnematophyceae and land plants, while others are also found in other green algae. Complete loss of these families in *Zygnema* may relate to the evolution of the thin cross walls connecting cells to form linear filaments in the four filamentous *Zygnema* (**Fig. 1d, e**).

Considering unique combinations, *Zygnema circumcarinatum* strains have a second set of co-orthologs that combine a HECT (PF00632) and a sugar transporter (PF05631) but no Ubiquitin, suggesting functional divergence. Other peculiar combinations included those revolving around the Lectin_legB domain (**Fig. 2i, Fig. 3h**). For example, Lectin_legB combines with F5_F8_type_C only in *Zygnema*, with Peptidase_C1 only in Zygnematophyceae, and with Pkinase only in *Zygnema* and land plants.

Supplementary Text 3: Cell wall complexity and innovation

The earliest land plants had to overcome stressors such as drastic temperature change, UV, high light, and pathogens not faced in aquatic habitats[96]. To adapt to the land environment, the common ancestors of Streptophyta must have made some major molecular innovations in their cell wall structures[97]. Cell walls are the first layer of natural barriers that protect plant cells from various environmental stresses, which differ significantly between aquatic and land environments. Cell walls also can constantly restructure during active cell growth, cell division, and in response to biotic or abiotic stresses. Streptophyta primary cell walls consist of polysaccharides: celluloses are tethered with various hemicelluloses (xylans, xyloglucans, mannans, and beta-glucans) and then embedded within the matrix of highly complex pectic polysaccharides, including homogalacturonan (HG), rhamnogalacturonan I (RG-I), rhamnogalacturonan II (RG-II), and arabinogalactan proteins (AGP)[98]. Celluloses are linear but most hemicelluloses and pectins have very complex sidechains. Besides being the main component of cell walls, some of these polysaccharides can be secreted to the outside of cells to from extracellular matrix and mucilage, critical for the algae to form biofilms, trap water, prevent desiccation, and interact with soil microbes[99,100].

Carbohydrate active enzymes (CAZymes) are responsible for the syntheses, degradations, and modifications of cell wall polysaccharides[101]. CAZymes include glycosyl hydrolase (GH), glycosyl transferase (GT), carbohydrate esterase (CE), and polysaccharide lyase (PL) classes, carbohydrate binding modules (CBMs) and auxiliary activities (AAs). Comparing the CAZyme repertories between 16 genomes through phylogenetic analyses could reveal the evolutionary innovations of cell wall syntheses and modifications from algae to plants (Fig. 4a) (e.g., ref.[102,103]). Previous immunocytochemical and biochemical studies suggest that the later-branching streptophyte algae (especially Zygnematophyceae) had already evolved the terrestrial pre-adapted cell walls and presented similar compositions and properties with land plants[98,104,105]. However, a holistic understanding of cell wall innovations has been missing.

Recent research highlighted gene gains, potentially via horizontal gene transfer (HGT), as a source for genetic innovations underpinning adaptive traits[13,106]. We hypothesized that HGT may have also contributed to the cell wall innovations. Therefore, we have focused our phylogenetic analyses on CAZymes involved in the syntheses and modifications of cellulose, mannan, beta-glucan, xyloglucan, xylan, AGP, and pectins. Our results revealed that: (i) the cell walls of embryophyte and Zygnematophyceae share all these major polysaccharide components including many of the sidechains and modifications; (ii) much of the cell wall innovations, especially for the synthesis of polysaccharide backbones, occurred after the common ancestor of Klebsormidiophyceae and Phragmoplastophyta; (iii) horizontal gene transfer (HGT) has played a major role in the origin of the enzymatic toolbox for the cell wall polysaccharide remodeling; (iv) gene loss is also very common in the cell wall gene families creating scattered distribution in sequenced streptophyte algal genomes.

Cellulose: Cellulose is the major component of plant cell walls, and the most abundant biopolymer on Earth. Cellulose is widely present in various organisms including algae. Plant cellulose synthase (CesA of GT2) subunits form the hexameric rosette CesA complex, responsible for the synthesis of cellulose microfibers (multiple β-1,4-glucose chains tightly bundled together by hydrogen bonds) and support plant growth. Previous studies have suggested that the rosette CesA complex has already evolved in the last common ancestor of Zygnematophyceae, Klebsormidiophyceae and Charophyceae[107] (Fig. 4a). A recent

phylogenetic analysis using Zygnematophyceae transcriptomes[22] suggested that the ortholog of land plant rosette CesAs first appeared in the Zygnematophyceae and land plant ancestor. Now, with the assembled genomes, the phylogeny clearly (Fig. 4f, Supplementary Data 1-1) showed that SAG 698-1b has two CesAs: ZcCesA1 (Zci_04468) together with CesAs of all other sequenced Zygnematophyceae are the direct orthologs of land plant CesA; all Zygnematophyceae genomes also have a second copy of CesAs, e.g., ZcCesA2 (Zci_03055), which are clustered with CesAs of Klebsormidiophyceae and Charophyceae. The presence of land plant-like CesA only in Zygnematophyceae suggests that the cellulose synthase complex (CSC) in a six-subunit rosette evolved first in the Zygnematophyceae and land plant ancestor. This agrees with earlier experimental observations (electron microscopy and isotope labeling) that Zygnematophyceae but not other algae have the hexameric rosette CSC [108]. The two CesA clades were further clustered with a newly defined CslQ clade (Zci_13680), which is also present in streptophyte algae.

The CslD (of GT2) clade, containing the Arabidopsis β-1,4-glucan synthase CSLD3 [109], does not have orthologs in any streptophyte algae except for *Coleochaete* (Fig. 4f). The phylogeny with NCBI-nr hits indicates that the CslQ clade represents the ancestor of the CesA and CslD clades, and was gained by an ancient HGT from bacteria into the common ancestor of streptophyte algae (Fig. 4f). Among the three SAG 698-1b CesA/CslQ homologs, the ZcCesA1 (Zci_04468) has much higher expression than the other two and responds to various stresses (Fig. 4e).

The GH9 endo-β-1,4 glucanase KORRIGAN (KOR), a membrane-bound cellulase, has been shown to be important for cellulose synthesis or assembly[107]. Arabidopsis GH9 proteins have been classified into three structural subclasses according to what functional domains they have: A (cytosolic domain, transmembrane domain, and catalytic domain), B (signal peptide and catalytic domain) and C (signal peptide, catalytic domain, linker region and cellulose binding domain CBM49)[110]. A has transmembrane regions so is located on the membrane, such as KOR. B and C has signal peptide so is likely secreted and degrades cellulose extracellularly. Our phylogenetic analysis with plant and algal GH9 proteins shows that there are two major clades (Supplementary Data 1-2), where clade 1 contains most Arabidopsis subclass B proteins and all subclass C proteins, and clade 2 contains all subclass A proteins and the rest of subclass B proteins. Clade 1 is restricted to land plants and Zygnematophyceae, while clade 2 first appeared in a Klebsormidiophyceae and Pragmoplastophyta ancestor (Supplementary Data 1-2).

SAG 698-1b has eight GH9 genes. Five of them belong to clade 1 and they all have CBM49 domains and signal peptides, and therefore belong to subclass C. Three genes belong to clade 2 (Zci_02466, Zci_12102, Zci_10931) and have much higher expression under various conditions than the five clade 1 genes. Zci_02466 has a CBM49 domain so belongs to subclass C, which might degrade cellulose extracellularly. Zci_12102 and Zci_10931 have TM region but no CBM49 so belong to subclass A (Supplementary Data 1-2), which may be involved in cellulose synthesis.

Mannan: Mannans are widely present in the cell walls of various algae (e.g., chlorophytes and streptophytes) and plants[111]. Mannan has a β-1,4-mannose backbone, while its variant glucomannan has β-1,4-linked glucose mixed in the mannose backbone[112] (Supplementary Data 1-3a). Galactomannan and galactoglucomannan are branched mannans with galactose sidechains. The CslA subfamily of GT2 are responsible for the synthesis of land plant mannan backbones[112]. The land plant CslA clade is phylogenetically clustered with a Zygnematophyceae-specific clade CslL (Zci_04551 and Zci_07893) and

the CslK clade (Fig. 4d, Supplementary Data 1-1). ClsK was initially thought to be only present in chlorophytes[113,114] but recently found to contain sequences from both chlorophytes and streptophyte algae[102]. This CslK clade may be the ancestor of CslA (mannan backbone), CslL, and CslC (xyloglucan backbone), but this needs further evidence. Including NCBI-nr hits in the phylogeny suggested a very ancient origin of CslK, likely via HGT from bacteria into ancient Archaeplastida (Supplementary Data 1-3b).

CslK and CslL are likely responsible for the mannan backbone synthesis in chlorophyte and streptophyte algae given their closer relationship with CslA than CslC (Supplementary Data 1-1, Fig. 4d). AlphaFold predicted protein structure of CslL are more similar to predicted protein structure of CslA (TM-score between Zci_07893 and AT2G35650: 0.85) than to CslC (TM-score between Zci_07893 and AT3G28180: 0.71). The predicted Zci_07893 structure was superimposed with the experimentally solved CesA of bacterial bcsA protein structure (4HG6) which contains its substrate UDP-glucose (Supplementary Data 1-4). The GDP-mannose (substrate of CslA) was docked into the superimposed structures showing that GDP-mannose fits well in the binding pocket interacting with the key motifs characterized in 4HG6 interacting with UDP-glucose (substrate of CesA). The candidate mannan synthase (Zci_07893, ZcCslL1) has as a high expression comparable to ZcCesA1 (Zci_04468); the other candidate (Zci_04551, ZcCslL2) is also expressed under various treatments at a lower level (Fig. 4e). This is supported by the notion that mannan is an abundant hemicellulose in algae[112].

MSR1 and MSR2 (GT106) are cofactors of ClsAs, and play a role in the synthesis of glucomannan. GT106 contains the pectic RG-I rhamnosyltransferases (RRTs), which share sequence homology with MSR1 and MSR2. The close homologs of MSR1 and MSR2 are present in Zygnematophyceae and most distant ones in Klebsormidiophyceae (Supplementary Data 1-5).

GMGT (MUCI10) of GT34 has been characterized as α-1,6-galactosyltransferase for the addition of galactose sidechain to mannans. GMGTs are only found in Zygnematophyceae and land plants (Supplementary Data 1-6), and are clustered with xyloglucan α-1,6-xylosyltransferases (XXTs) in the GT34 phylogeny. The phylogeny revealed that GT34 homologs are also present in other chlorophyte and streptophyte algae, but it was until in Zygnematophyceae that XXTs and GMGTs were separated due to gene duplications in ancient Zygnematophyceae.

For modifications, MOAT1-4 (DUF231) are mannan acetylation enzymes. The DUF231 phylogeny shows that MOAT1-4 first appeared Charophyceae (Supplementary Data 1-7). The acetylation enzymes for xylans (XOATs) and pectins (PMR5) also belong to the DUF231 family. The phylogeny shows that XOATs and PMR5 are separated in land plants and their orthologs are present in Zygnematophyceae and Coleochaetophyceae.

For degradations, MAN1-3 are characterized as endo-β-1,4-mannanases (GH5_7)[115]. The homologs of MAN1-3 are missing in the Zygnema genus but are present in chlorophytes, streptophyte algae, and land plants (Supplementary Data 1-8). This is consistent with the wide distribution of mannans in plant and green algae. A phylogeny with NCBI-nr hits suggests an ancient origin of plant and green algal mannanases from bacteria through HGT (Supplementary Data 1-9). AGAL2 and AGAL3 (GH27) have an α-galactosidase activity and may act on the cleaving off galactose sidechains; their orthologs first appeared

in the Klebsormidiophyceae and Phragmoplastida ancestor and may have gained from fungi through HGT (Supplementary Data 1-10).

Mixed linkage glucan and other beta-glucan: MLG in monocots (grasses and cereals) consists of unbranched and interspersed chains of β-1,3;1,4-glucose polymer[116]. MLG is absent in other angiosperms but also present in ferns, brown and green algae, fungi, and bacteria[117]. The CslF and CslH of GT2 are responsible for the synthesis of MLGs in monocots but are absent in Zygnematophyceae and other non-monocots. In fungi, *Aspergillus fumigatus* Tft1 (XP_748682.1) has been characterized as the enzyme for the synthesis of MLG, which is a component of fungal cell walls[118]. In bacteria, *Sinorhizobium meliloti bgsBA* operon encodes a MLG synthase (*bgsA*, SM_b20391) and a putative transporter (*bgsB*) [119]. The *bgsA* gene was shown to be phylogenetically closer to β-1,3-glucan synthase *crdS* than to bacterial CesA *bcsA/celA*[119].

From the phylogeny of GT2 (Fig. 4d), there are three newly defined Csl clades consisting of almost exclusively streptophyte algae. CslO (Zci_07462 and Zci_15850) and CslP (Zci_01910 and Zci_11882) are phylogenetically separated from CslA/C/K/L and CesA/CslD/CslQ. Including NCBI-nr hits found that CslO is also present in non-seed land plants such as moss, spike moss, and fern (Supplementary Data 1-11). CslO is further clustered with the fungal Tft1, suggesting that CslO is likely transferred from fungi (Fig. 4d, Supplementary Data 1-11). CslO also contains *Physcomitrium patens* Pp3c12_24670 (PNR44324.1)[120], which has been functionally characterized for the synthesis of a novel polysaccharide called arabinoglucan (AGlc) currently only found in *P. patens*. Therefore, it is also possible that CslO functions as AGlc synthase in streptophyte algae.

Including NCBI-nr hits found that CslP is absent in land plant, but present in various microalgae such as streptophyte and chlorophyte algae (Fig. 4d, Supplementary Data 1-11). Interestingly, CslP and CslO are phylogenetically close to each other, and further clustered with known CesAs from oomycetes, tunicates, amoeba, and red algae[121-124]. The microalgal CslP proteins (average 1,474 amino acids) and the micro-eukaryotic CesAs (average 1,252 amino acids) are much longer than CslO proteins (average 659 amino acids) (Supplementary Data 1-12). The closest bacterial homologs of CslP/O proteins are predominantly from Cyanobacteria and Chloroflexi, which include known Cyanobacterial CesA proteins[125,126] (Supplementary Data 1-12). An additional clade next to cyanobacterial CesAs in the phylogeny include CesAs from Proteobacteria, Planctomycetota, Actinobacteria. These CesAs have the hallmark c-di-GMP-binding PilZ domain of the well characterized bacterial cellulose synthase A subunit (bcsA)[127] (Supplementary Data 1-12). Therefore, the phylogenetic clustering with various bacterial CesAs suggests that the eukaryotic CslP/O proteins have originated via HGT from bacterial CesAs (Fig. 4d, Supplementary Data 1-12). After that, new functions of CslO evolved in different eukaryotic organisms (e.g., MLG synthesis in fungi and likely also in various microalgae, AGlc synthesis in mosses) while the cellulose synthesis function conserved in oomycetes, tunicates, amoeba, red algae, and possibly also in green algae with CslP. Clearly, the CslP/O-like CesAs had a distinct origin compared to the land plant CesAs, an example of convergent evolution. Among the four SAG 698-1b CslO/P proteins, Zci_01910 (CslP) shows relatively high expression and responses to different stresses (Fig. 4e).

CslN (Zci_08939) also shows responses to different stresses (Fig. 4e). CslN is closer to CslA/C/K/L, and uniquely found in Zygnematophyceae. Searching against NCBI-nr found no hits in any other algae and

plants. The top hits are all bacterial proteins mostly from Actinobacteria, Proteobacteria, and Chloroflexi, suggesting CslN could have originated from bacteria via HGT (Fig. 4d, Supplementary Data 1-13). However, none of the close bacterial homologs have characterized functions. Interestingly, in addition to the C-terminal GT2 domain, most of these CslN-like bacterial proteins also have an N-terminal PleD region (Supplementary Data 1-13). PleD contains receiver domains (RECs) that respond to phosphorylation by dimerization, and a diguanylate cyclase domain (DGC or GGDEF) that is activated by dimerization leading to the production of an important secondary messenger c-di-GMP[128]. The GT2 domain fused with PleD in these CslN-like proteins is likely a c-di-GMP effector domain. In bcsA proteins, c-di-GMP binds to their C-terminal PilZ domain to activate the N-terminal cellulose synthesis domain[129]. Unlike bcsA, these CslN-like bacterial proteins do not have the PilZ domains. However, some CslN-like proteins have genes usually found in the gene neighborhood of bcsA (e.g., bcsC, bcsQ). For example, in the *Polaromonas eurypsychrophila* genome, the CslN-like gene (GGB02139) has an upstream TRP domain-containing bcsC homolog (GGB02133)[127]. In addition, GT2 domain could also be found in enzymes without the PilZ domains, e.g., pgaC for the synthesis of poly-N-acetylglucosamine (PNAG), a major component of bacterial extracellular polysaccharide matrix for biofilm formation[129]. Therefore, the CslN-like bacterial proteins could function as cellulose synthases or PNAG synthases. Given that PNAG is not present in streptophyte, we infer that CslN in Zygnematophyceae is also a cellulose synthase.

Xyloglucan (XyG): XyG is an important hemicellulose that contain complex sidechains (Supplementary Data 1-14), whose compositions are highly variable among different land plants[102]. XyG released from plants can act as soil particle aggregator, which could modify the soils suitable for early plant terrestrialization[130]. The four *Zygnema* genomes contain the XyG backbone synthases (CslC of GT2, Supplementary Data 1-1, Fig. 4a), and the sidechain xylosyltransferases (XXT of GT34, Supplementary Data 1-15), galactosyltransferase (XLT/MUR3 of GT47, Supplementary Data 1-16, Supplementary Data 1-17), galacturonosyltransferase (XUT of GT47, Supplementary Data 1-17), fucosyltransferase (FUT of GT37, Supplementary Data 1-18). Interestingly, in addition to the FUT clade consisting of only embryophyte and Zygnematophyceae, GT37 family is significantly expanded in Zygnematophyceae with a separate clade that only contains sequences from Zygnematophyceae. GT37 also has distant homologs in *Chara* and *Klebsormidium*, as well as in many Mortierellaceae fungi [131] (Supplementary Data 1-19), which are saprotrophs in the soil. This indicates that HGT between fungi and streptophyte algae may have contributed to the fucosyltransferase origin. Notably, although CslC is also present in *Chara* and *Klebsormidium*, GT34 and GT47 are restricted in Zygnematophyceae and embryophytes.

For modifications, the XyG backbone degradation enzyme XTH (GH16_20) family is significantly expanded in Zygnematophyceae (Supplementary Data 1-20), while it also has homologs in Klebsormidiophyceae and Coleochaetophyceae. XTH might have been involved in HGT between fungi and ancient streptophyte algae (Supplementary Data 1-21)[132]. The fucosidase (AXY8, GH95), involved in the degradation of fucosylated xyloglucans and AGP[133,134], might have gained from bacteria into Coleochaetophyceae and Zygnematophyceae through HGT (Supplementary Data 1-22). The xyloglucan β-galactosidase (BGAL, GH35, Supplementary Data 1-23) appears to be present only in land plants. The α-xylosidase (AXY3, GH31, Supplementary Data 1-24) seems conserved across different streptophytes and chlorophytes. All these agree with recent studies[102,135] suggesting that the backbone of XyG might have originated earlier, while the full enzymatic system for XyG has evolved in the Zygnematophyceae and embryophyte ancestor.

Callose: Callose is a polymer of β-1,3-glucan[136] (Supplementary Data 1-25). It is a minor component of cell walls in most plant cells but plays a vital role in cell plate maturation and cytokinesis[137]. Callose is found in chlorophyte and streptophyte algae[137,138]. Unlike other β-glucans, callose is not synthesized by GT2 proteins. Instead, GSL (glucan synthase-like) enzymes of GT48 are involved in the callose synthesis and deposition. The phylogeny showed that Arabidopsis GSLs form three clades, each with some homologs from streptophyte algae (Supplementary Data 1-26). GSL homologs in chlorophytes are clustered with fungi, brown alga and Omycetes (Supplementary Data 1-27). GH17 family is responsible for callose degradation. Previous phylogenetic analysis has identified three clades of embryophyte proteins within GH17[139]: alpha and gamma are closer and thought to be restricted in embryophytes, while beta also contain Streptophyte algae. Our phylogeny (Supplementary Data 1-28) confirmed that alpha and gamma clades are separated and expanded in embryophytes, while share ancestors in Streptophyte algae but not earlier than Klebsormidiophyceae. Beta is also present in streptophyte algae as early as Klebsormidiophyceae. The previous study[139] also found GH17 present in fungi and close to beta clade, suggesting possible HGT between fungi and ancient streptophyte algae.

GH5_14 is a subfamily of GH5[140], which contain two experimentally characterized activities: exo-β-1,3-glucosidase (EC 3.2.1.58) and β-glucosidase (EC 3.2.1.21) according to http://www.cazy.org/GH5_14_characterized.html. Therefore, GH5_14could be involved in the degradation of beta-glucans such as callose, MLG, and cellulose. The rice GH5_14 protein GH5BG was involved in cell wall recycling and stress response. GH5_14 was thought to be restricted to embryophytes[140], but was recently found to be prevalent in oomycetes[141]. In our search against the sequenced streptophyte genomes using the GH5_14 HMM from dbCAN as query[142] (E-value < 1e-10), only Zygnematophyceae and land plants have GH5_14 hits, and Zygnematophyceae has significantly more GH5_14 hits than land plants (9 in SAG 698-1b vs. 2 in moss, Supplementary Data 1-29). When extend the research to NCBI-nr (E-value < 1e-15), still no other algal hits were found. The close non-streptophyte hits are from oomycetes, fungi, amoeba, and Actinobacteria (Supplementary Data 1-30). The phylogeny suggests that GH5_14 in these eukaryotes might have been gained from bacteria through HGT. Interestingly, most streptophyte GH5_14 proteins have a functionally unknow N-terminal fascin-like domain in addition to the GH5_14 domain[140], while non-streptophyte GH5_14 homologs. The combination of Fascin and GH5_14 domains first occurred in Zygnematophyceae (Supplementary Data 1-31).

Xylan: Xylans are the most abundant hemicellulose in vascular plants with β-1,4-xylose backbone (Supplementary Data 1-32). The xylose can be decorated with glucuronic acids (GlcA) at O-2 sites to form glucuronoxylan (GX), with L-arabinose at the O-2 or O-3 sites to form arabinoxylan (AX), and with both modifications to form glucuronoarabinoxylan (GAX). Much has been known for the GTs involved in xylan synthesis[143]. GT43 and GT47 contain enzymes responsible for the xylan backbone synthesis. Specifically, IRX10 and XYS1 (IRX10L) of GT47, and IRX9, IRX9L, IRX14 and IRX14L of GT43, form a protein complex. GT47 is a large protein family in streptophyte (Supplementary Data 1-33). The GT47 phylogeny shows that IRX10 and XYS1 have orthologs in Zygnematophyceae and Klebsormidiophyceae but not in other streptophyte algae (Supplementary Data 1-34). The presence of IRX10 and XYS1 orthologs in Klebsormidiophyceae agrees with the verified β-1,4-xylan synthase activity of *Kf*XYS1 from *K. flaccidum*[144]. The clade of IRX10/XYS1 is phylogenetically next to a clade containing IRX7 (FRA8) and

F8H (for the synthesis of xylan backbone at the reducing end), which is only present in land plants and Klebsormidiophyceae (Supplementary Data 1-34).

The GT43 (IRX9, IRX9L, IRX14 and IRX14L) family (Supplementary Data 1-35) is present in Zygnematophyceae, Coleochaetophyceae, Klebsormidiophyceae and Charophyceae[145], but significantly expanded with a clade unique to Zygnematophyceae (Supplementary Data 1-35). The phylogeny with NCBI-nr hits indicates that GT43 is also widely present in animals encoding β-1,3-glucuronyltransferase for heparan synthesis (Supplementary Data 1-36). A small number of *Rhodotorula* fungal hits are found phylogenetically closer to streptophyte than to animals, suggesting the possibility of HGT between fungi and streptophyte algae.

GT8 proteins are also involved in xylan backbone (IRX8 and PARVUS) and sidechain (GUX1-5) synthesis (Supplementary Data 1-37). IRX8 and PARVUS (GATL1) have orthologs in Zygnematophyceae (PARVUS absent in *Zygnema*, Supplementary Data 1-38), Coleochaetophyceae, Klebsormidiophyceae and Charophyceae. GUX1-5, which add the GlcA sidechain, has orthologs only in Zygnematophyceae (but not in *Zygnema*, Supplementary Data 1-38). Two GT61 proteins (XAX1 and XAT) are involved in α-1,2- and α-1,3-arabinosyl transfer onto xylan backbone to form arabinoxylan (Supplementary Data 1-39a). The GT61 phylogeny reveals three major clades (Supplementary Data 1-39b). The previously defined clades A and B[146] can be merged into one clade, which contain land plant XAX1 and XAT orthologs, as well as proteins from Zygnematophyceae, Coleochaetophyceae, Klebsormidiophyceae. This is confirmed by a larger phylogeny with NCBI-nr hits (Supplementary Data 1-39c). The clade C contains AT5G55500 experimentally characterized for the synthesis of N-glycans (2.4.2.38), which is clustered with characterized animal O-GlcNAcylation enzymes (2.4.2.255). There is a new clade D defined here that only contains proteins from Zygnematophyceae (Supplementary Data 1-39b). However, the larger phylogeny with NCBI-nr hits shows that clade D contains numerous bacteria proteins, which have not yet been experimentally characterized (Supplementary Data 1-39c).

The GlcA and xylose in xylans can be methylated or acetylated. The homologs of GlcA methyltransferase GXMT1-3 (DUF579) are present in Zygnematophyceae (only in *Spirogloea muscicola*) and Charophyceae (Supplementary Data 1-40), but these algal sequences are clustered separately with the GXMT1-3 clades, suggesting an unknown function. The xylose acetyltransferase ESK1 (DUF231) is present in Coleochaetophyceae and Zygnematophyceae (Supplementary Data 1-41). Additionally, the arabinose in the sidechain can be linked to coumaric and ferulic acids (phenolic phytochemicals) catalyzed by BADH (coumaric and ferulic acid transferase). The BADH (PF02458) was found to be restricted to monocot plants[146]. Lastly, the deacetylation enzymes BS1 (backbone xylosyl deacetylase) and DARX1 (sidechain arabinosyl deacetylase) seem to be present in Klebsormidiophyceae, Coleochaetophyceae and Zygnematophyceae (Supplementary Data 1-42).

Altogether, phylogenetic analyses suggest that enzymes for the synthesis of xylan backbones have already evolved in the Klebsormidiophyceae and Phragmoplastophyta ancestor, while the synthesis of sidechains and modifications have evolved later. For example, GUX1-5 for the GlcA sidechain likely appeared in the Zygnematophyceae and land plant ancestor, and XAX1 and XAT for the addition of arabinose sidechains are restricted to land plants.

Arabinogalactan protein (AGP): In plant cell walls, arabinogalactan-proteins (AGPs) are highly glycosylated hydroxyproline-rich glycoproteins (Supplementary Data 1-43). AGPs have been shown in seed plants to play important roles in plant growth, morphogenesis, cell division, apoptosis, pattern formation, abiotic and biotic stresses, sexual reproduction, and plant-microbe interactions[147]. AGPs in bryophytes and ferns contain highly branched galactan and unusual monosaccharides such as acofriose (3-O-Me-Rha)[148]. Recently, significant modifications of AGPs were found in *Spirogyra pratensis* of the Zygnematophyceae to have less arabinoses but much more rhamnoses in the sidechains[105], leading to the proposed presence of "rhamnogalactan-protein" or RGP.

At least five GT families are known for the synthesis of AGP backbone and sidechains (Supplementary Data 1-43)[149,150]. Our phylogenetic analyses (Supplementary Data 1-44) suggest that the enzymes for the synthesis of the β-1,3-galactan backbone and β-1,6-galactan sidechain of AGP are present in Zygnematophyceae, Klebsormidiophyceae, Coleochaetophyceae, and Charophyceae. These enzymes include: (1) GALT2-6 and HPGT1-3 (GT31), which add β-1,3-galactan to Hyp residues of protein backbone, (2) GALT8 and KNS4 (GT31) that elongate the β-1,3-galactan backbone, and (3) GALT31A (GT31) that elongates the β-1,6-galactan sidechain (Supplementary Data 1-45). GALT29A (GT29) is also involved in elongation of β-1,6-galactan but is absent in Zygnematophyceae (Supplementary Data 1-46). The β-1,6-galactan sidechains can be highly decorated (Supplementary Data 1-43) with glucuronic acid (GlcA), fucose (Fuc), rhamnose (Rha), arabinose (Ara) and xylose (Xyl). The decorations vary significantly among different plants and algae. The arabinosyltransferase (RAY1, GT77, Supplementary Data 1-47) and β-glucuronosyltransferases (GlcAT14A-E, GT14, Supplementary Data 1-48) first appeared in the Klebsormidiophyceae and Phragmoplastophyta ancestor, but were significantly expanded and diversified in Zygnematophyceae. The α-1,2-fucosyltransferases (FUT4,6,7, GT37) first appeared and significantly expanded in Zygnematophyceae (Supplementary Data 1-49). Arabinogalactan methylesterases (AGM1 and AGM2), which methylate the GlcA residues in the sidechains, do not have homologs in Zygnematophyceae except for *Spirogloea muscicola* (Supplementary Data 1-50). However, the three sequences of *S. muscicola* are clustered separately with AGM1-2 in the NCBI-nr phylogeny which might indicate a different function in this species (Supplementary Data 1-40). The lack of methylation of the acidic GlcA may explain the excessive mucilage in *Zygnema*. All these suggest that the biosynthesis machinery for AGP has already evolved in Charophyceae but undergone significant expansion in Zygnematophyceae, which explains the much higher structural complexity present in Zygnematophyceae and land plants.

Plants also encode GH enzymes to break glycosidic linkages for modifying cell wall structures. At least seven GH families are found in Zygnematophyceae for AGP modifications. The AGP β-1,3-galactan backbone can be broken by GH43A and GH43B (GH43_24, Supplementary Data 1-51), which are present in Zygnematophyceae, Coleochaetophyceae, and Charophyceae. Phylogenetic analysis shows that GH43_24 evolved from bacteria through HGT (Supplementary Data 1-52). For the β-1,6-galactan sidechain degradation, GH30_5 and GH35 are responsible. Interestingly, GH30_5 is only found in Zygnematophyceae, mosses, and liverworts, and absent in any other streptophyte and algae (Supplementary Data 1-53). More interestingly, including NCBI-nr hits in phylogenetic analysis shows that streptophyte and arbuscular mycorrhizas (AM) fungal (Glomeromycota) GH30_5 homologs are next to each other, and may might have been acquired through HGT from bacteria (Supplementary Data 1-54). The beta-galactosidases (BGALs) of GH35, on the other hand, seem to be only present in land plants, while other GH35 homologs are found in streptophyte algae (but not in *Zygnema*) (Supplementary Data 1-55).

As for sidechain decorations, α-arabinofuranosidases XYL1 and XYL4 (GH3) are found in all plants and algae including Chlorophyta (Supplementary Data 1-56). The β-l-arabinopyranosidases AGALs (GH27) first appeared in the Klebsormidiophyceae and Phragmoplastida ancestor (Supplementary Data 1-57), while the other β-l-arabinopyranosidases APSE (GH27) are found in lands plants and Mesostigmatophyceae. Including NCBI-nr hits in the phylogeny shows that HGT from bacteria is a plausible origin of APSE in Streptophyta (Supplementary Data 1-58). GH95 can specifically cleave α-1,2- linage of fucose and first appeared in Coleochaetophyceae and Zygnematophyceae; our phylogenetic analysis suggests that it might have been gained from bacteria through HGT (Supplementary Data 1-22). GUS2 (GH79) has been demonstrated to cleave GlcA from AGP, and first appeared in Klebsormidiophyceae (Supplementary Data 1-59), and seems to have an origin from bacteria via HGT (Supplementary Data 1-60).

Pectin: Pectins include a class of the most complex cell wall polysaccharides[151]. Pectins play a variety of roles in plant growth, development, cell wall plasticity, response to abiotic stress, cell-cell adhesion and communication, and innate immunity. Pectins are loosely classified into three groups: homogalacturonan (HG), rhamnogalacturonan I (RG-I), and rhamnogalacturonan II (RG-II). HGs exist mostly without sidechains, but in some plants or algae, there are HGs with one single xylose or apiose sidechains, called xylogalacturonan (XGA) and apiogalacturonan (AGA).

HG is a polymer of α-1,4 galacturonic acid (GalA), and makes up 65% of pectins in plant cell walls (Supplementary Data 1-61). AtGAUT1 and AtGAUT7 of GT8 form a protein complex responsible for the HG biosynthesis (Supplementary Data 1-37). AtGAUT1-7 orthologs first appeared in the ancestor of Klebsormidiophyceae and Phragmoplastophyta (Supplementary Data 1-62), although HG was not chemically detected in *Klebsormidium*[98]. AtGAUT8 (QUA1) is also involved in HG biosynthesis and has orthologs in Coleochaetophyceae and Zygnematophyceae (not in *Zygnema* though). GalA in HGs is often methylated or acetylated. The HG methyltransferase QUA2 is absent in *Zygnema* but present in other Zygnematophyceae, as well as Coleochaetophyceae and Charophyceae (Supplementary Data 1-63), while QUA3 is only found in Zygnematophyceae and land plants. CGR2 and CGR3 are also methyltransferases but their homologs are absent in Zygnematophyceae (homologs in Coleochaetophyceae, Charophyceae, and Mesostigmatophyceae, Supplementary Data 1-64). The HG acetyltransferase PMR5 first appeared in Charophyceae (Supplementary Data 1-65). These findings indicated that the full machinery for HG synthesis has already evolved in Charophyceae, which is consistent with the immunocytochmemical and chemical evidence by [98]. The β-1,3-xylosyltransferase XGD1 (GT47, Supplementary Data 1-33) for XGA synthesis appears to be present in Coleochaetophyceae and Charophyceae but absent in Zygnematophyceae (Supplementary Data 1-66). Pectin methylesterases (PMEs) of CE8 and acetylesterases (PAEs) of CE13 can release the methyl and acetyl groups from the pectins, respectively. Homologs of PMEs have been found in *Klebsormidium*, and were reported to have been gained from bacteria through HGT[106]. The land plant PME genes form two groups in our CE8 phylogeny (Supplementary Data 1-67), and have many orthologs in Klebsormidiophyceae and Phragmoplastophyta. However, PME is entirely absent in the genus *Zygnema* (Supplementary Data 1-67). PAEs are also found in Klebsormidiophyceae and Phragmoplastophyta (including *Zygnema*) (Supplementary Data 1-68). The phylogeny with NCBI-nr hits shows that PAEs in plants are closer to bacteria and chlorophytes than to streptophyte algae, suggesting ancient HGT from bacteria (Supplementary Data 1-69). The PAEs from streptophyte algae are instead

clustered with a group of marine invertebrates (e.g., corals, starfish, sea anemone, jellyfish, mussels) and haptophyte microalgae (e.g., *Emiliania huxleyi*) (Supplementary Data 1-69).

RG-I constitutes 20–35% of pectin and has a backbone composed of repeating units of GalA and rhamnose (Rha). Its sidechains contain arabinans (α-1,5-linked) and galactans (β-1,4-linked) and/or arabinogalactans (Supplementary Data 1-70). RG-I rhamnosyltransferases AtRRT1-4 and galaturonosyltransferase AtRGGAT1/MUCI70 are responsible for the synthesis of RG-I backbone [152,153]. AtRRT belongs to a large GT106 family (Supplementary Data 1-71), which also contain other characterized proteins for the mannan and arabinogalatan syntheses (Supplementary Data 1-5). AtRRT orthologs are found in Zygnematophyceae, Coleochaetophyceae, and Klebsormidiophyceae (Supplementary Data 1-71). AtRGGAT1 and their homologs define a new GT family GT116 (DUF616), and were phylogenetically classified into five clades[152]. AtRGGAT1 belongs to clade A (Supplementary Data 1-72), which contains orthologs in Zygnematophyceae, Coleochaetophyceae, Charophyceae, and Klebsormidiophyceae. Although other clades may have different functions, the GT116 family has NCBI-nr hits restricted within streptophytes and bacteria, suggesting plant GT116's origin from bacteria via HGT (Supplementary Data 1-73).

The ARAD1 and ARAD2 of GT47 are involved in the synthesis of the sidechain α-1,5-arabinan of RG-I. They appeared first in the ancestor of Klebsormidiophyceae and Phragmoplastophyta but absent in *Zygnema* (Supplementary Data 1-74, S33). GALS1-3 (GT92) are responsible for the sidechain β-1,4-galactan synthesis [154]. Their orthologs first appeared in the ancestor of Klebsormidiophyceae and Phragmoplastophyta and significantly expanded in the four *Zygnema* genomes (9 genes in each genome) (Supplementary Data 1-75). GT92 phylogeny also revealed a separate cluster with proteins almost exclusively from Zygnematophyceae. The significantly increased GT92 family size in *Zygnema* may be related to the high mucilage content in Zygnematophyceae to facilitate water fixation.

TBG4/5 (GH35) specifically hydrolyzes RG-I β-galactan sidechains[155] and are present only in land plants (Supplementary Data 1-55). Polysaccharide lyase family 4 subfamily 2 (PL4_2) can cleave α-1,4 backbone of RG-I. PL4_2 contains seven *Arabidopsis* genes (AtRGL1-7). Our phylogeny shows that streptophyte PL4_2 homologs form two clades: AtRGL1-7 are in clade A, which only contain land plants and Zygnematophyceae (Zci_02033 in SAG 698-1b), and clade B contains liverworts and streptophyte algae as early as Klebsormidiophyceae (Supplementary Data 1-76). Including NCBI-nr hits (Supplementary Data 1-77) confirms that clade B represents the ancestral RGL clade, which originated from bacteria via HGT, and clade A remains to be present only in land plants and Zygnematophyceae.

RG-II is the most structurally complex class of pectins and has the same backbone as HG (Supplementary Data 1-78). RG-II contains up to 13 different mono-sugars and more than 20 different glycosidic linkages, forming highly diverse sidechains. Enzymes for most of the glycosidic linkages in RG-II are currently unknown except for three GT families. GT8 (GAUT1 and GAUT7) has been discussed above for HG backbone synthesis (Supplementary Data 1-62). GT77 (RGXT1-4) has been shown to possess α-1,3-xylosyltransferase activity, which adds xylose to fucose in the sidechain. AtRGXT1-4 have orthologs in Klebsormidiophyceae and Zygnematophyceae except for *Zygnema* (Supplementary Data 1-79). GT37 fucosyltransferase (FUT) can synthesize α-1,2 linked Gal-Fuc disaccharide structure in RG-II sidechain, which is also present in XyG and AGP. FUT first appeared in and significantly expanded in the

Zygnematophyceae and land plant ancestor (Supplementary Data 1-49). SIA1 and SIA2 (GT29) can transfer rare sugars Dha or Kdo onto the RG-II sidechains[156] but their orthologs are only found in land plants (Supplementary Data 1-80).

GH28 family contains 67 polygalacturonases (PGs) for pectin backbone degradation in *Arabidopsis*, which were classified into endo-PGs, exo-PGs, and rhamono-PGs[157,158]. Phylogenetic analyses in these previous studies have revealed six clades, where clade E is the only one that contain algal sequences and corresponds to rhamono-PGs. Our phylogeny (Supplementary Data 1-81) suggests that GH28 in Viridiplantae should be reclassified into three classes: (i) class 1 contains not only proteins from land plants (including AT3G57790 and Zci_08289), streptophyte algae, but also chlorophyte algae, and represents the earliest GH28 class in green plants; (ii) class 2 contains most clade E proteins from land plants and streptophyte algae (Zci_09024) as early as Klebsormidiophyceae; (iii) class 3 contains proteins of other previous defined clades (A-D,F)[157,158] from streptophytes as early as Charophyceae, but is absent in *Zygnema*. Phylogenies were also built to include NCBI-nr hits (Supplementary Data 1-82). The three classes have different origins. Class 1 (Zci_08289) is also present in chlorophytes and likely evolved from bacteria via HGT. Class 2 (Zci_09024) is found in more diverse algae (e.g., diatoms, chlorophytes) and bacterivorous marine flagellates, and also might have evolved from bacteria via HGT. Class 3 (ADPG1/At3g57510) is clustered with fungi, so it might have acquired from fungi via HGT.

According to the CAZy database (www.cazy.org), PL1_1 and PL_12 are pectate lyases and present in plants. Three plant PL1_1 enzymes have been characterized to play important roles in fruit ripening, rhizobial infection, cell elongation and differentiation[159-161]. *Arabidopsis* has 24 PL1_1 genes and 2 PL_12 genes (Supplementary Data 1-83a). These 26 genes form two clades in the phylogeny (Supplementary Data 1-83b), which shows that PL1_1 is first present in *Chara* and might be gained from bacteria via HGT (Supplementary Data 1-84a). PL1_12 (At3G09540 and At3G55140) has a different origin than PL1_1 but might be also gained bacteria via HGT (Supplementary Data 1-84b). There is also a different clade significantly expanded in *Penium*, which is also found in Coleochaetophyceae and early diverging land plant lineagess (e.g., *Magnoliopsida, Amborella, Selaginella,* bryophytes). Interestingly, PLs are absent from the four *Zygnema* genomes.

In conclusion, pectic polysaccharides have deep evolutionary origins in ancestral streptophyte algae and later evolved into the complex structures that resemble to land plants. The gradually evolved cell wall structures paved the way from water to terrestrial environments.

Supplementary Text 4: Gene co-expression modules.

Connectivity among signaling pathways

Module 13: It is expressed mostly during dark conditions. Co-expression of PYL (Zci_14749.1) and PP2CA (Zci_08054.1) homologs that take part in ABA signaling in land plants, together with CHK (Zci_14717.1) the upstream cytokinin receptor and an homolog of the temperature sensor protein (PHY-B; Zci_14452.2). These might represent sensors of environmental conditions. Several co-expressed transcription factors (TFs) that might function as downstream signals modulating a high number of processes including a MYB TF that has been involved with cell division (Zci_02625.1), a bHLH TF (Zci_01323.1) and PSRP1 (Zci_02860.1). Several CDPKs (Zci_10340.1-2, Zci_12959.1, Zci_02615.1) might represent signal transducers by calcium signaling. The regulatory factor (TZF) of mRNA stress granule formation (Zci_00238.1), which form by stalled mRNA during translation, might be involved in modulating the transcriptional response. Two metabolic proteins are also co-expressed, phosphoenolpyruvate carboxykinase (PEPCK; Zci_07284.1) involved in gluconeogenesis and pyruvate orthophosphate dikinase (PPDK; Zci_10550.1) involved in pyruvate metabolic processes. A gamete membrane fusogen (HAP2; Zci_09502.1), likely involved in sexual reproduction is also co-expressed, but its function remains unclear.

Module 21: Expressed mostly in cold liquid conditions. Two ELIP homologs (Zci_05110.1, Zci_10821.1) associated with LHC proteins and acting against photooxidative damage in cold stress also in *Spirogyra*[162] and LEA stress signaling (cold); SnRK1 kinase complex (Zci_04668.1) is a metabolic sensor of declining energy levels[163]; oleosin homolog (Zci_13615.1) involved in lipid droplet formation. A member of the CHK cytokinin receptor (Zci_01725.4) family is also co-expressed. PP2Cs (Zci_11367.1) are likely a *Zygnema*-specific expansion; ABI1/ABI2 might be involved in ABA biosynthesis. We also find a cell cycle regulatory protein (CYCP; Zci_00417.1) and two genes related to calcium signaling: calcium-permeable channel (OSCA; Zci_03379.1) and components of the SnRK1 kinase complex (Zci_04668.1). Liquid cold condition seem to also affect carbon (starch) metabolism, since several enzymes are present in this cluster, including pyruvate decarboxylase (PDC; Zci_02661.1) which might play a role in fermentation during anoxia, starch synthase (SSI; Zci_08721.1), sucrose synthase (SUS; Zci_02211.1) starch-debranching pullulanase (PU1; Zci_13261.2), alpha amylase (AMY; Zci_13243.1), glucan water dikinase (GWD; Zci_10596.1), or a subunit of the pyruvate dehydrogenase E1 complex (Zci_03990.1) that might act as a glucose metabolism regulator. Several transporters of sugar are also found (Zci_10714.1-3, Zci_10716.1, Zci_01877.1, Zci_10438.2) as well as transporters of ions (Zci_11202.1, Zci_09619.1) and a metal chelator transporter (ZIF/TOM; Zci_07518.1) from OG 258 that is expanded in Zygnematophyceae. The co-expression of the serine carboxypeptidase (Zci_07069.1-2) might indicate a link with stress response, since this enzyme is involved in specialized metabolism of land plants. Similarly to other clusters in this set, we find a bHLH TF (Zci_05570.1).

Module 57: It is expressed mostly under desiccation conditions (at 4ºC and 20ºC). Homologs of ethylene response factors (Zci_06095.1, Zci_06551.1), which have been involved in cell division (Supplementary Table 3d), are co-expressed with PP2Cs (Zci_11373.1) involved in ABA and stress signaling and LRR receptor like Ser/Thr PKs (Zci_12836.1), as well as CDPKs (Zci_00963.1, Zci_06373.1, Zci_12935.1) that are involved in calcium signaling and cell division (Supplementary Table 3d). Co-expression of bHLH TF (Zci_04798.1) might indicate regulation of many downstream processes. Ubiquitin ligase (Zci_03205.1)

that is involved in stress response and is expanded in the Zygnematophyceae + Embryophyta ancestor (OG 78).

Module 79: An RRB homolog (Zci_10020.1) is expressed together with genes D27 homolog (XPF; Zci_11659.1) (strigolactone pathway) and genes involved in stress signaling such as a member (Zci_07396.2) of a methyltransferase family expanded in Zygnematophyceae (OG 269) and an homolog (Zci_04762.1) putatively involved in ROS scavenging that belongs to an expanded gene family (OG 942) and components of the DNA repair machinery (Zci_03237.2), carotenoid isomerase (Zci_11659.1) and carotenoid beta ring hydroxylase (Zci_06424.1). Proteins involved in photosynthesis (LPA3 homolog Zci_15251.1 involved in photosystem-II assembly and lysine M-methyltransferase involved in Rubisco regulation Zci_12586.1), cell division (extension homolog Zci_06787.3), and mTERF transcription factors (Zci_10727.1-2).

Module 117: It is expressed mostly under stresses (osmotic, cold, high light, desiccation, high pH) and darkness conditions. Signaling cascades: PP2C (Zci_09733.2) and calcium signaling CDPKs (Zci_02946.1, Zci_02275.1); biotic stress with LRR-receptor-like Ser/Thr PK *Nodulation receptor kinase* homolog (Zci_15062.1). Several genes related to cell division and cell wall production (callose synthase Zci_11576.1, Zci_11575.1). The gene with the most connections is a LRR-rich protein (Zci_14397.1).

Module 155: Homologs of the AHP (Zci_15778.1) that act as cytokinin receptor in land plants is co-expressed with ethylene responsive element (ERE)-binding factor homolog (Zci_09057.1), a calcium sensor (CML homolog; Zci_15281.1) and several other stress-responsive elements such as WRKY TF (Zci_07707.1) and PP2As (Zci_00613.2).

LRR, plant-microbe interaction, and immunity

Module 107: Various receptor proteins containing LRR (Zci_10118.3, Zci_13403.2, Zci_13802.1, Zci_14960.1) are co-expressed with protein kinases (Zci_14960.1) and ROP-activating protein (Zci_12487.4[164]) involved in signaling cascades, as well as with TOM2B (Zci_03483.1), which in *Arabidopsis thaliana* is necessary for the multiplication of tobamoviruses[165]. This cluster is expressed the most during dark or stressful (UV, Mannitol, high light) conditions.

Module 128. Expressed highly under desiccation conditions at 4ºC. At least seven LRR-domain containing proteins (Zci_00066.1, Zci_06052.1, Zci_06073.1, Zci_06280.1, Zci_12747.2, Zci_02469.1, Zci_06051.1, etc.) and various receptor-like proteins (Zci_01085.1, Zci_02469.1, Zci_03265.1, Zci_03270.1, Zci_08788.1) co-express with calcium sensor and kinase (CPK; Zci_12352.1) and genes involved in cell division (including some containing LRR domains such as BAK1 Zci_06073.1 or SRF3 Zci_03265.1).

Module 163. At least three LRR proteins (Zci_06264.1, Zci_10217.1, Zci_13925.1) and immunity suppressor (SOBER/TIPSY) of effector-triggered immunity (ETI) network (Zci_13211.1) co-expresses with a plastidial protease (EGY; Zci_01876.1) and a protein involved in photosystem-II assembly (LPA1; Zci_08073.1), which might suggest a link with photosynthesis response as this cluster is most strongly expressed under UV and other stresses.

Ethylene, stress, and growth

Module 31: It is expressed mostly during darkness (2h and 6h) and to a lesser extent under salty or high pH conditions. The ethylene-responsive transcription factor ERF056 homolog (Zci_08934.1) is co-expressed with several proteins involved in cell wall such as myricetin 3-O-rhamnosyltransferase UGT77B2 (Zci_15629.1), beta-1,3-arabinofuranosyltransferase (RAY) (Zci_01127.1) and beta-glucosidase 40 (Zci_11532.1) that might be involved in xyloglucan biosynthesis. We also observe coexpression with photosynthetic enzymes such as carboxylesterases (Zci_11534.1, Zci_12551.1, Zci_11538.1, Zci_12552.1) as shown by Van de Poel[166]. There is a co-expression with Subtilisin-like protease SBT1.7 (Zci_03084.1) involved in stress response, as well as various members of the calcium signaling pathway including calcium-dependent PKs (Zci_12156.1) and calcium-permeable channel OSCA (Zci_03062.1). Finally, we observe expression of several transporters (Zci_08992.1-2, Zci_06764.2-4), multicopper oxidases (Zci_10048.1, Zci_11530.1, Zci_11536.1, Zci_14613.1), as well as a Nodulin-26-like intrinsic protein (NIP; Zci_13075.1) that in land plants are expressed in the nodules and transport small solutes like glycerol.

Module 110: Expressed mostly during stress conditions. ACS (key regulatory enzyme in ethylene biosynthesis) is co-expressed with several cell division genes and the circadian clock activation factor (LWD; Zci_12354.1). Immunity genes (response to biotic stress). Some genes are involved in photosynthesis (PAM68 involved in photosystem-II assembly; Zci_03683.1), starch metabolism (MRC/PII1; Zci_06237.1) and chloroplast-to-nucleus signaling factor (GUN; Zci_09564.1). In relation to plastidial functions, we identify scaffold protein NFU of plastidial SUF system transfer phase (Zci_14524.1) involved in the assembly and transfer of the iron-sulfur cluster, essential for growth and development. We also identify genes that suggest increased transcription, including the regulatory protein (MAS2) of rDNA transcription (Zci_14610.1) and the members of the SPT6-IWS1 transcription elongation complex (Zci_16265.1). Two fucosyltransferase-like proteins are also co-expressed (FUT12; Zci_10608.1, Zci_11120.1).

Module 119: Ethylene receptor ETR1 homologs (Zci_05897.1, Zci_13702.1) co-express with genes involved in cell division such as AUGMIN subunit 5 (Zci_02013.1), U-box TF (Zci_12905.1), microtubule-associated protein (Zci_14445.2), formin-like protein (Zci_07438.1), or homologs of protein kinases involved in cell division (Zci_09465.1, Zci_10880.1, Zci_14390.1). There are several genes related to chloroplast division too, including the plastid division FtsZ assembly factor (MinE; Zci_05088.1), chloroplastic cell division topological specificity factor homolog (Zci_05088.1), regulatory GTPase (OBGL) of thylakoid biogenesis (Zci_02652.1). A circadian clock repression factor (TZP) (Zci_02597.1) is also co-expressed and stress-related protein such as phosphoinositide 3-phosphatase (PTEN; Zci_00158.1)[167], an auxin effector transporter (PIN; Zci_00189.1) and calcium channel (OSCA; Zci_06929.1), which suggest a relationships to biotic or abiotic stress. Finally, a regulatory protein (SIEL) of plasmodesmata intercellular trafficking (Zci_01783.2-3) is found along with various transporters (Zci_06494.1, Zci_13581.1).

Cytokinin, stress, and growth

Module 22: A CHK homolog (Zci_01726.2) is expressed along with several genes involved in cell division and development, including master regulators (CYCA cell cycle regulator Zci_07128.2, SAGA TF Zci_02679.1, ubiquitination activator proteins CDC20 Zci_04452.1, GTPase Ran Zci_00360.1), genes directly involved in cell division (plate maturation factor AIR9 Zci_13210.1) and cell wall (cellulose

synthase interactive protein Zci_02379.1), but also in DNA replication (DNApol Zci_04461.2, DNA double-strand break transducer kinases or ATMs Zci_04846.1, Zci_04846.3, nucleoporin NUP160 Zci_01440.1), RNA transcription (Zci_01435.3 RQC complex, organellar RNApol Zci_09104.1) and a high number of transporters (subfamily ABCC transporters Zci_00407.1, purine transporter (AZG) Zci_15121.1, glutathione S-conjugate transporters Zci_00407.3), and MADs-box TF (Zci_13806.1).

Module 76: Another CHK homolog (Zci_10812.1) co-expresses with several genes involved in cell division and development including substrate adaptor FBX of SCF E3 ubiquitin ligase complexes (Zci_05197.1-3) and ribosome assembly GTPase (LSG1) (Zci_13272.1), as well as genes involved in DNA replication (DNA pol Zci_05608.1), RNA translation (RNA helicase (Prp16) Zci_05123.1, PRP40C Zci_04671.1). A WRKY TF (Zci_04595.1) involved in stress response and specialized metabolism is also co-expressed.

Module 151: Coexpression of a ARRB homolog (Zci_12313.1) with genes involved in cell division and growth, including Cell division control protein 2 homolog (Zci_15818.1), Exocyst complex component SEC15B (Zci_08043.1), Polyadenylate-binding protein-interacting protein 11 (Zci_07200.1), ATPase family AAA domain-containing protein 1-B (Zci_07210.1), and Peroxisome biogenesis protein 5 (Zci_15347.1).

Cell division

Module 10: At least 28 genes related to cell division and development are expressed, including RAB GTPases (Zci_06045.2, Zci_09204.1, Zci_10774.1), various protein kinases (MAST; Zci_04486.1 LRR-IX, Zci_09278.1; S6K Zci_10847.1; PBL27/RLCK185; Zci_06709.1), subunit alpha of coat protein complex (Zci_04821.1), cargo receptor (Zci_07289.1, Zci_07538.1, Zci_01007.1, Zci_03751.1, Zci_04600.1), phosphometabolite transporter (Zci_01863.1, Zci_04009.1), and nucleotide sugar transporters (GONST1/2/3/4 Zci_02365.1; UTR7 Zci_05941.1). Several genes related to cell wall function are also co-expressed, such as components CesA of cellulose synthase complex (Zci_04468.1-3), 1,4-beta-glucan synthase (CSLC; Zci_01359.1), 1,2-alpha-fucosyltransferase (FUT; Zci_01415.1), UDP-L-arabinose mutase (Zci_03317.1), rhamnosyltransferase (RRT; Zci_03464.1), beta-1,3-arabinofuranosyltransferase (RAY; Zci_03879.1), beta-1,2-arabinosyltransferase (RRA; Zci_10664.1), UDP-L-rhamnose synthase (RHM; Zci_10782.1). We also find co-expression of beta-glucosidase (Zci_13484.1) involved in xyloglucan biosynthesis for the cell wall or plant chemical defense[168] that was found to be expanded in the Zygnematophyceae + Embryophyta ancestor. The coordinated division of plastids with cells is suggested by the co-expression of the component FtsZ1 of plastid division (Zci_03618.1). Several transporters are also found, including those for ions (Zci_00439.1, Zci_00696.1, Zci_01321.1, Zci_04295.1), nucleic acids or nucleotide sugar transport (Zci_01636.1, Zci_01762.1, Zci_05941.1), amino acids (Zci_05903.1), and fatty acids (Zci_12117.1). Finally, we find co-expression of a methyltransferase putatively involved in stress and member of a gene family expanded in the Zygnematophyceae ancestor (Zci_15213.1; OG 369) as well as a protein involved in photosystem-II assembly (HCF173; Zci_00865.1).

Module 52: It is mostly expressed during long (14h) diurnal light conditions, probably reflecting conditions that are favorable for cell division. At least 17 genes known to be related to cell division, including several motor proteins (Kinesin-4 Zci_09690.2-3; Kinesin-7 Zci_04776.1; Kinesin-10 Zci_01966.1, Zci_14745.1;

Kinesin-12 Zci_03783.1; Kinsein-14 Zci_10412.2-3), the strubbelig-receptor family (Zci_05247.2), SYP1-group SNARE component (Zci_08036.1), or various protein kinases (Zci_14388.2, Zci_03733.3) or the spindle assembly checkpoint protein (MAD3/BUBR1; Zci_08931.1). Importantly, two proteins of the cell cycle are co-expressed: a cyclin regulatory gene (CYCA; Zci_06721.2) and Cyclin-dependent protein kinase (CDKB; Zci_10930.1). A coordinated division of plastids and cells is suggested by the coexpression of several genes involved in plastid division such as MinD (Zci_00690.1), MinE (Zci_03796.1), regulatory protein ARC6 of plastid division FtsZ assembly (Zci_15616.1), component of the FtsZ2 of plastid division FtsZ prokaryotic-tubulin filaments (Zci_08565.1). A member of the alpha-fucosyl transferase family (Zci_02280.1), belonging to the orthogroups OG 89 expanded in Zygnematophyceae and involved in xyloglucan fucosylation in the cell wall, is also found. Similarly to module 10, a methyltransferase potentially involved in stress response (Zci_07987.1) is co-expressed, which belongs to the same orthogroups OG 369 expanded in Zygnematophyceae. We also identify several genes involved in the DNA packaging and segregation such as DNA topoisomerase (TOP2; Zci_01326.2), members of the DNA helicase complex (Zci_09643.1), various chromatin remodeling factors (DDM1s Zci_09776.1-3, ERCC6 Zci_06390.2), several components of the condensing I and II complexes (Zci_04539.2, Zci_07988.2, Zci_07988.3, Zci_14672.1, Zci_06340.1), or components of the ORC1 of origin recognition complex (Zci_04778.1), NDC80 outer kinetochore complex (Zci_07612.4) and cohesin regulator complex (Zci_04764.4).

Module 87: It has at least 27 genes involved in cell division and development, including phragmoplastin (DRP1) homologs (Zci_00226.1, Zci_00382.1), kinesin-14 (Zci_01429.1), RAB GTPases (Zci_02001.1, Zci_02165.1), ARF-GTPase (ARFC1; Zci_04499.1), light chain of clathrin triskelion (Zci_04290.1), SNARE components (Zci_04323.1, Zci_00014.1, Zci_06454.1), E3 ubiquitin ligase (LOG2/LUL; Zci_03693.1) and components of the SKP1-CUL1-FBX (SCF) E3 ligase complexes (Zci_06777.1), cargo adaptor complexes (Zci_08039.1, Zci_09188.1), MT plus-end-tracking protein (EB1; Zci_11487.1), component alpha type-3 of 26S proteasome(Zci_14386.1), component MAC3 of non-snRNP MOS4-associated complex (Zci_10069.1), ATPase CDC48 (Zci_01456.1), or protein kinase homologs related to cell division (Zci_10720.1, Zci_04888.1). The relationship with cell cycle is revealed by presence of circadian clock time-of-day-dependent expressed repressor (PRR; Zci_09313.1). Coexpression of calcium sensors (CBL; Zci_04108.1-2, Zci_09428.1) and CDPKs (Zci_06184.1-2) show a relationship with calcium signaling. The presence of a homolog of the regulatory EIN2-stabilizing factor (MHZ3; Zci_06173.1) – involved in ethylene signaling in land plants[169] -might speak of a link to this phytohormone signaling, and coexpression of a PP2A component (Zci_04881.1) might show link to stress.

Gene modules with GRAS domain-containing proteins

Module 93: It is expressed particularly during cadmium treatment but also at lower levels in all other conditions. A scarecrow (SCR) homolog (Zci_05047.1) is co-expressed with a number of proteins that might be upstream activators such as cold shock protein (Zci_05375.1) or many downstream genes involved in a number of functions such as cyclin (Zci_04874.2) and cyclin-dependent protein kinase (Zci_13306.1), RAG GTPase (Zci_03275.1) related to cell cycle, an actin-related protein (Zci_15614.1), ATP-dependent metalloprotease (FtsH4/11; Zci_11579.1), and proteins related to cell wall such as the regulatory protein (KOBITO) of cellulose-hemicellulose network assembly (Zci_07093.1). A regulatory protein (TRP1) of ethylene receptor activity (Zci_00278.1) and an ABA signal transducer (AIP2; Zci_04511.1). In agreement with the role of scarecrow as TF (other TFs are also expressed such as C3H zinc finger TF Zci_07575.1 or

WOX Zci_11970.1), we find several genes related to transcription and translation: components of the transcription initiation and elongation (Zci_06120.1) and basal transcription regulation (Zci_06649.1) complexes, tRNA ligases (Zci_05585.3, Zci_06065.1), proteasome units (Zci_12272.1), RNA splicing factors (Zci_15987.1, Zci_05053.1), components of the RNA quality control exon junction complex (Zci_04684.1), and members of HIRA chaperone complex (Zci_01979.3-4).

Module 38: It is expressed under all tested conditions. One SCR homolog (Zci_05035.2) is co-expressed with genes with a wide range of functions. The most numerous category refers to photosynthetic function, including members of the chlorophyll biosynthetic pathway (Zci_00837.1, Zci_02862.1, Zci_08599.1, Zci_13791.1), components of the light harvesting complex (Zci_00732.1), components of the photosystem-I (Zci_03607.1, Zci_04387.1, Zci_04388.1, Zci_04401.1, Zci_06449.1, Zci_07052.1, Zci_07278.1, Zci_09986.1, Zci_10392.1, Zci_11077.1, Zci_12461.1, Zci_12463.1, Zci_13084.1, Zci_15241.1) and photosystem-II complexes (Zci_02769.1, Zci_04586.1, Zci_04782.1, Zci_04788.1, Zci_05966.1, Zci_05967.1, Zci_05968.1, Zci_05974.1, Zci_07970.1, Zci_08643.1, Zci_09519.1, Zci_10944.1, Zci_11585.1), thylakoid biogenesis factor (CPSFL1; Zci_05979.1), a member of the tylakoid Sec1 translocation systems (Zci_10929.1), plastocyanin electron carrier (Zci_15419.1) , members of the NDH complexes (Zci_02313.1, Zci_05574.1), the chloroplastic SNT7 homolog (chloroplastic Ser/Thr PK; Zci_04200.1) involved in LHCII phosphorylation that affects stability of photosystem I and thylakoid membrane folding[170], plastid RNApol cofactors (Zci_06249.1) and plastid transport complexes Plastid transport (TIC component; Zci_06248.1). The coexpression of ABA4 (Zci_00401.2) LUT2 (Zci_12915.1) homologs might tight photosynthetic function to ABA signaling as well as xantophyll cycle as mitigation strategy under photosynthetic stress. Photooxidative protection is also suggested by the coexpression of photosynthetic acclimation MPH2 acclimation factor (Zci_08642.1) and various redox proteins such as thioredoxins (Zci_01036.1, Zci_01621.1), the plastidial alkenal/alkenone oxidoreductase (AOR; Zci_09126.1) that detoxifies reactive carbonyls, or xylulose-1,5-bisphosphate phosphatase (CbbY; Zci_06107.1) that degrades a rubisco inhibitor[171]. Related to the photosynthetic function, we observed coexpression of the tricarboxylic acid cycle member PEP carboxylase homolog (Zci_01696.1). A potential link to ABA signaling is also supported by an ABA perception modulator (ABAR; Zci_11356.1). Perhaps reflecting the super-regulatory role of SCR TFs, we observe coexpression of several TFs such as WRKY (Zci_14275.1), CAMTA TF (Zci_10968.1) involved in biotic and abiotic stress responses[172], and ARF10 homolog (Zci_13015.1). This latter TF is part of the auxin signaling network. It is also tightly involving DNA bending architectural proteins (HMG-B) that regulate transcription (Zci_05868.1-3) and DNA chromomethylase homolog (Zci_14617.1) involved in regulating gene expression through methylation. We also identify links with gibberellin pathway (GA20OX2; Zci_02249.1), the circadian clock time-of-day-dependent expressed repressor (PRR; Zci_04537.1) and kinesin-like protein (Zci_14393.1) likely involved in cell division. The only gene directly linked to SCR in the network (Zci_04942.1) is an uncharacterized protein with a zinc finger domain (YRD6_CAEEL).

Module 147: It is expressed under all conditions, highest under mannitol. Two proteins of the GRAS family are found: chitin-inducible gibberellin-responsive protein 2 homolog (Zci_03997.1) and SCR (Zci_05048.1). These proteins have been previously associated with cell division (Table S3D) and in fact they are co-expressed with other genes associated to cell division (Zci_03458.1, Zci_03420.1, Zci_09405.1, Zci_15145.1) a component of the cohesion regulator complex (SMC1/TTN8; Zci_01002.2) that is important in cell division and beta-1,3-arabinofuranosyltransferase (Zci_11525.1) from the cell wall

polysaccharides. Beta-glucosidase homolog (Zci_11705.1) might be involved in xyloglucan biosynthesis or plant chemical defense[168] and this family was found to be expanded in the Zygnematophyceae + Embryophyta ancestor. In line with the role of SCR as master regulator, we find co-expression of ELIPs (Zci_05151.2; members of the expanded OG 97 in *Zygnema* ancestor) associated with stress responses, along with a circadian clock component (ELF4; Zci_02304.1) and proteins involved in methylation such as histone methyltransferases (Zci_05266.2, Zci_13743.1) and histone demethylase (Zci_04903.1), as well as CAMTA (Zci_10996.1) and other TFs (Zci_03488.1) components of miRNA biogenesis (Zci_12634.2). The calcium cation channel (DMI1/Pollux/Castor) (Zci_08200.1) and calcium-dependent PKs (Zci_12942.1) and one LRR receptor-like protein kinase (Zci_06706.1).

Supplementary Text 5: Phytohormone biosynthesis and signaling pathways

Cytokinin: Cytokinin is involved in plant growth and stress. In *Zygnema*, one out of four co-orthologs of the *A. thaliana* cytokinin receptor AHK2-4 contains the CHASE domain required for binding cytokinin (Zci_13126). Other CHASE-domain-containing histidine kinase (CHK) homologs result from more ancient duplications in streptophytes, where homologs previously identified *Spirogyra* and *Mougeotia* likely belong[173]. Two *Zygnema* CHKs were found to co-express with cell division and development genes such as cell cycle regulators, plate maturation factors, cell wall-relevant cellulose synthase, DNA replication factors and the only identified MADS-box gene (modules 22, 76). Among response regulators, we found that the *Zygnema* strains lack Type-A *Arabidopsis* response regulators (RRA) and have only two Type-B *Arabidopsis* response regulators (RRB), and their involvement in cytokinin signaling of Zygnematophyceae is unclear; in fact RRA showed low responsiveness to exogenous cytokinin in *Spirogyra pratensis*[173]. Moreover, the paucity of RRA/RRB genes in non-vascular plants supports the notion that this cytokinin-mediated induction of response regulators might be a feature of seed plants[174]. Yet, RRB homologs are co-expressed with genes involved in cell division (module 151) and stress response (module 79).

Ethylene. In accordance with its deep evolutionary roots[175,176], the chassis for ethylene biosynthesis and signaling is present in Zygnematophyceae. EBF1, which binds EIN3 and is responsible for the last step in the signaling cascade, shows an almost land-plant specific distribution, with the exception of EBF1, which is not found in *Zygnema* but homologs are found in *Penium* and *Klebsormidium*. It is not yet known whether these algal homologs bind EIN3 in physiological conditions. In land plants, ethylene triggers cell wall matrix modification, reduces chlorophyll biosynthesis and photosynthesis, and activates abiotic stress responses. Exogenous ethylene treatment in the Zygnematophyte *Spirogyra* triggered similar transcriptomic responses and cell elongation[166] and *Spirogyra* genes can complement *Arabidopsis* ethylene signaling KO lines[176]. Several abiotic stress conditions have been shown to stimulate cell elongation in an ethylene-dependent manner. Congruently, module 110, deployed under stress (high light, osmotic stress, UV, high pH), shows ACS—a key regulator of ethylene biosynthesis—co-expressed with cell division and circadian clock genes, genes related to photosystem II assembly, starch metabolism, and immunity. Two homologs of ethylene receptor ETR1 co-express with genes involved in cell and plastid division, the PIN auxin effector transporter, calcium signaling SIEL, a regulator of plasmodesmata intercellular trafficking (module 119). Module 31, deployed largely under darker growing conditions (2h and 6h) and to a lesser extent under high salt or high pH, shows co-expression of an ethylene-responsive factor with genes involved in cell wall remodeling (transferases and beta-glucosidases), photosynthesis, and stress responses (calcium

signaling genes, subtilisin-like protease). These modules illustrate well the effects of ethylene in modifying the cell wall matrix, downregulating photosynthesis, and activating abiotic stress responses as known from land plants.

Abscisic acid. Major aspects of the ABA signaling network are conserved across land plants[177-179]. The four new *Zygnema* genomes contain a complete set of homologous genes to the ABA signaling cascade, including *Pyrabactin Resistance 1 /PYR1-like /Regulatory Component of ABA Receptor* (PYR/PYL/RCAR) receptors, as previously identified[10,13]. Functional Supplementary Data howed that Zygnematophyceae PYL regulates downstream phosphatases in an ABA-independent manner [180]. Among ABA biosynthetic genes, two key *A. thaliana* enzymes lacked homologs outside of land plants (NCED3, Nine-cis-Epoxycarotenoid dioxygenase 3 and ABA2, SHORT-CHAIN DEHYDROGENASE/ REDUCTASE 1). Yet, we detected about 1.01±0.13 ng/g ABA in SAG 698-1b cultures by LC-MS (**Figure S13**), suggesting that these reactions occur by alternative routes, perhaps *via* an ABA1-independent biosynthetic pathway starting upstream of zeaxanthin as suggested by Jia et al.[181]. An interesting observation is the expansion of clade A PP2Cs in *Zygnema* (5 homologs), akin to the 9 homologs found in *Arabidopsis*. The expansion of PP2Cs was also detected by orthogroup expansion analyses (OG 548 and OG 830), but it must be noted that the high PP2C numbers in *Arabidopsis* and *Zygnema* derive from independent duplications.

Auxin. Auxin is the major morphogenic phytohormone. Its polar distribution—largely based on the PIN proteins[182]—leads to gradients along plant bodies, shaping various developmental processes in all land plants (e.g., refs.[183,184]). While all components of the canonical auxin signaling pathway likely first came together in land plants [185-187], PIN-mediated polar auxin transport might have emerged earlier [188]; that said, not all streptophyte algal PINs localize polarly[189,190]. While TAA homologs involved in auxin biosynthesis are found in several streptophyte algae including *Zygnema*, no YUCCA homologs were found in any Zygnematophyceae. In fact, embryophyte YUCCAs might have been acquired by HGT from bacteria[191]. Similarly, several signaling proteins (e.g., TIR, AUX, GH) are also absent from *Zygnema* and distant homologs (never co-orthologs) are sometimes present in other streptophyte algae. These patterns suggest that auxin signaling might be land plant specific.

Strigolactones. Strigolactone biosynthesis has been well established in land plants[192-195]. The streptophyte alga *Nitella* has been shown to produce and respond to strigolactone although the actual biosynthetic pathway might differ from the canonical one of embryophytes [5,175]. Indeed, no clear orthologs of carotenoid cleavage dioxygenases (CCD7 and CCD8; **Supplementary Figure 14**), key biosynthetic enzymes, were found in its transcriptome nor in the genome of the close relative *Chara braunii*[5]. Strigolactones were also detected in some chlorophyte algal species for which no genomes data are available[196]. We find close homologs of strigolactone biosynthesis genes in all *Zygnema* genomes (including CCD7 orthologs) as well as across the whole Chloroplastida. Yet, all CCD7-8 homologs outside of embryophytes do not show conserved amino acid motifs proposed to be important for substrate specificity (**Supplementary Figures 11, 12**)[86]. The angiosperm strigolactone sensor D14 has likely evolved in the seed or vascular plant ancestors through neofunctionalization of karrikin-sensing F-box proteins[195] and thus no homologs are found in any algae. Our results consolidate the hypothesis that strigolactone biosynthesis and signaling differ between embryophytes and streptophyte algae.

Supplementary References:

1 Project, A. G. *et al.* The Amborella genome and the evolution of flowering plants. *Science* **342**, 1241089 (2013).

2 Li, F.-W. *et al.* Fern genomes elucidate land plant evolution and cyanobacterial symbioses. *Nature plants* **4**, 460-472 (2018).

3 Moreau, H. *et al.* Gene functionalities and genome structure in Bathycoccus prasinos reflect cellular specializations at the base of the green lineage. *Genome biology* **13**, 1-16 (2012).

4 Initiative, T. I. B. Genome sequencing and analysis of the model grass Brachypodium distachyon. *Nature* **463**, 763-768 (2010).

5 Nishiyama, T. *et al.* The Chara genome: secondary complexity and implications for plant terrestrialization. *Cell* **174**, 448-464. e424 (2018).

6 Merchant, S. S. *et al.* The Chlamydomonas genome reveals the evolution of key animal and plant functions. *Science* **318**, 245-250 (2007).

7 Blaby, I. K. *et al.* The Chlamydomonas genome project: a decade on. *Trends in plant science* **19**, 672-680 (2014).

8 Wang, S. *et al.* Genomes of early-diverging streptophyte algae shed light on plant terrestrialization. *Nature Plants* **6**, 95-106 (2020).

9 Blanc, G. *et al.* The genome of the polar eukaryotic microalga Coccomyxa subellipsoidea reveals traits of cold adaptation. *Genome biology* **13**, 1-12 (2012).

10 de Vries, J., Curtis, B. A., Gould, S. B. & Archibald, J. M. Embryophyte stress signaling evolved in the algal progenitors of land plants. *Proceedings of the National Academy of Sciences* **115**, E3471-E3480 (2018).

11 Hori, K. *et al.* Klebsormidium flaccidum genome reveals primary factors for plant terrestrial adaptation. *Nature communications* **5**, 3978 (2014).

12 Bowman, J. L. *et al.* Insights into land plant evolution garnered from the Marchantia polymorpha genome. *Cell* **171**, 287-304. e215 (2017).

13 Cheng, S. *et al.* Genomes of subaerial Zygnematophyceae provide insights into land plant evolution. *Cell* **179**, 1057-1067. e1014 (2019).

14 Kawahara, Y. *et al.* Improvement of the Oryza sativa Nipponbare reference genome using next generation sequence and optical map data. *Rice* **6**, 1-10 (2013).

15 Palenik, B. *et al.* The tiny eukaryote Ostreococcus provides genomic insights into the paradox of plankton speciation. *Proceedings of the National Academy of Sciences* **104**, 7705-7710 (2007).

16 Jiao, C. *et al.* The Penium margaritaceum genome: hallmarks of the origins of land plants. *Cell* **181**, 1097-1111. e1012 (2020).

17 Lang, D. *et al.* The Physcomitrella patens chromosome‐scale assembly reveals moss genome structure and evolution. *The Plant Journal* **93**, 515-533 (2018).

18 Nystedt, B. *et al.* The Norway spruce genome sequence and conifer genome evolution. *Nature* **497**, 579-584 (2013).

19 Banks, J. A. *et al.* The Selaginella genome identifies genetic changes associated with the evolution of vascular plants. *science* **332**, 960-963 (2011).

20 De Clerck, O. *et al.* Insights into the evolution of multicellularity from the sea lettuce genome. *Current Biology* **28**, 2921-2933. e2925 (2018).

21 Prochnik, S. E. *et al.* Genomic analysis of organismal complexity in the multicellular green alga Volvox carteri. *Science* **329**, 223-226 (2010).
22 Fitzek, E. *et al.* Cell wall enzymes in Zygnema circumcarinatum UTEX 1559 respond to osmotic stress in a plant-like fashion. *Frontiers in plant science* **10**, 732 (2019).
23 Laslett, D. & Canbäck, B. ARWEN: a program to detect tRNA genes in metazoan mitochondrial nucleotide sequences. *Bioinformatics* **24**, 172-175 (2008).
24 Langmead, B. & Salzberg, S. L. Fast gapped-read alignment with Bowtie 2. *Nature methods* **9**, 357-359 (2012).
25 Seppey, M., Manni, M. & Zdobnov, E. M. BUSCO: assessing genome assembly and annotation completeness. *Gene prediction: methods and protocols*, 227-245 (2019).
26 Mendes, F. K., Vanderpool, D., Fulton, B. & Hahn, M. W. CAFE 5 models variation in evolutionary rates among gene families. *Bioinformatics* **36**, 5516-5518 (2021).
27 Koren, S. *et al.* Canu: scalable and accurate long-read assembly via adaptive k-mer weighting and repeat separation. *Genome research* **27**, 722-736 (2017).
28 Zhang, H. *et al.* dbCAN2: a meta server for automated carbohydrate-active enzyme annotation. *Nucleic acids research* **46**, W95-W101 (2018).
29 Andrews, S. (Babraham Bioinformatics, Babraham Institute, Cambridge, United Kingdom, 2010).
30 Price, M. N., Dehal, P. S. & Arkin, A. P. FastTree: computing large minimum evolution trees with profiles instead of a distance matrix. *Molecular biology and evolution* **26**, 1641-1650 (2009).
31 Castresana, J. Selection of conserved blocks from multiple alignments for their use in phylogenetic analysis. *Molecular biology and evolution* **17**, 540-552 (2000).
32 Kearse, M. *et al.* Geneious Basic: an integrated and extendable desktop software platform for the organization and analysis of sequence data. *Bioinformatics* **28**, 1647-1649 (2012).
33 Tillich, M. *et al.* GeSeq–versatile and accurate annotation of organelle genomes. *Nucleic acids research* **45**, W6-W11 (2017).
34 Vurture, G. W. *et al.* GenomeScope: fast reference-free genome profiling from short reads. *Bioinformatics* **33**, 2202-2204 (2017).
35 Kim, D., Paggi, J. M., Park, C., Bennett, C. & Salzberg, S. L. Graph-based genome alignment and genotyping with HISAT2 and HISAT-genotype. *Nature biotechnology* **37**, 907-915 (2019).
36 Finn, R. D., Clements, J. & Eddy, S. R. HMMER web server: interactive sequence similarity searching. *Nucleic acids research* **39**, W29-W37 (2011).
37 Nguyen, L.-T., Schmidt, H. A., Von Haeseler, A. & Minh, B. Q. IQ-TREE: a fast and effective stochastic algorithm for estimating maximum-likelihood phylogenies. *Molecular biology and evolution* **32**, 268-274 (2015).
38 Letunic, I. & Bork, P. Interactive Tree Of Life (iTOL) v5: an online tool for phylogenetic tree display and annotation. *Nucleic acids research* **49**, W293-W296 (2021).
39 Wang, D., Zhang, Y., Zhang, Z., Zhu, J. & Yu, J. KaKs_Calculator 2.0: a toolkit incorporating gamma-series methods and sliding window strategies. *Genomics, proteomics & bioinformatics* **8**, 77-80 (2010).

40 Chikhi, R. & Medvedev, P. Informed and automated k-mer size selection for genome assembly. *Bioinformatics* **30**, 31-37 (2014).

41 Katoh, K. & Standley, D. M. MAFFT multiple sequence alignment software version 7: improvements in performance and usability. *Molecular biology and evolution* **30**, 772-780 (2013).

42 Campbell, M. S., Holt, C., Moore, B. & Yandell, M. Genome annotation and curation using MAKER and MAKER‐P. *Current protocols in bioinformatics* **48**, 4.11. 11-14.11. 39 (2014).

43 Wang, Y. *et al.* MCScanX: a toolkit for detection and evolutionary analysis of gene synteny and collinearity. *Nucleic acids research* **40**, e49-e49 (2012).

44 Crescente, J. M., Zavallo, D., Helguera, M. & Vanzetti, L. S. MITE Tracker: an accurate approach to identify miniature inverted-repeat transposable elements in large genomes. *BMC bioinformatics* **19**, 1-10 (2018).

45 Li, H. Minimap2: pairwise alignment for nucleotide sequences. *Bioinformatics* **34**, 3094-3100 (2018).

46 Marçais, G. *et al.* MUMmer4: A fast and versatile genome alignment system. *PLoS computational biology* **14**, e1005944 (2018).

47 Dierckxsens, N., Mardulyn, P. & Smits, G. NOVOPlasty: de novo assembly of organelle genomes from whole genome data. *Nucleic acids research* **45**, e18-e18 (2017).

48 Greiner, S., Lehwark, P. & Bock, R. OrganellarGenomeDRAW (OGDRAW) version 1.3. 1: expanded toolkit for the graphical visualization of organellar genomes. *Nucleic acids research* **47**, W59-W64 (2019).

49 Emms, D. M. & Kelly, S. OrthoFinder: phylogenetic orthology inference for comparative genomics. *Genome biology* **20**, 1-14 (2019).

50 Yang, Z. PAML 4: phylogenetic analysis by maximum likelihood. *Molecular biology and evolution* **24**, 1586-1591 (2007).

51 Haas, B. J. *et al.* Improving the Arabidopsis genome annotation using maximal transcript alignment assemblies. *Nucleic acids research* **31**, 5654-5666 (2003).

52 Walker, B. J. *et al.* Pilon: an integrated tool for comprehensive microbial variant detection and genome assembly improvement. *PloS one* **9**, e112963 (2014).

53 Revell, L. J. phytools: an R package for phylogenetic comparative biology (and other things). *Methods in ecology and evolution*, 217-223 (2012).

54 Kajitani, R. *et al.* Platanus-allee is a de novo haplotype assembler enabling a comprehensive access to divergent heterozygous regions. *Nature communications* **10**, 1702 (2019).

55 Wang, H.-C., Minh, B. Q., Susko, E. & Roger, A. J. Modeling site heterogeneity with posterior mean site frequency profiles accelerates accurate phylogenomic estimation. *Systematic biology* **67**, 216-235 (2018).

56 Antipov, D., Korobeynikov, A., McLean, J. S. & Pevzner, P. A. hybridSPAdes: an algorithm for hybrid assembly of short and long reads. *Bioinformatics* **32**, 1009-1015 (2016).

57 Vaser, R., Sović, I., Nagarajan, N. & Šikić, M. Fast and accurate de novo genome assembly from long uncorrected reads. *Genome research* **27**, 737-746 (2017).

58 Stamatakis, A. RAxML version 8: a tool for phylogenetic analysis and post-analysis of large phylogenies. *Bioinformatics* **30**, 1312-1313 (2014).

59 Bergman, C. M. & Quesneville, H. Discovering and detecting transposable elements in genome sequences. *Briefings in bioinformatics* **8**, 382-392 (2007).
60 Yang, Z., Kumar, S. & Nei, M. A new method of inference of ancestral nucleotide and amino acid sequences. *Genetics* **141**, 1641-1650 (1995).
61 Kalvari, I. *et al.* Non‐coding RNA analysis using the Rfam database. *Current protocols in bioinformatics* **62**, e51 (2018).
62 Pertea, M., Kim, D., Pertea, G. M., Leek, J. T. & Salzberg, S. L. Transcript-level expression analysis of RNA-seq experiments with HISAT, StringTie and Ballgown. *Nature protocols* **11**, 1650-1667 (2016).
63 Capella-Gutiérrez, S., Silla-Martínez, J. M. & Gabaldón, T. trimAl: a tool for automated alignment trimming in large-scale phylogenetic analyses. *Bioinformatics* **25**, 1972-1973 (2009).
64 Bolger, A. M., Lohse, M. & Usadel, B. Trimmomatic: a flexible trimmer for Illumina sequence data. *Bioinformatics* **30**, 2114-2120 (2014).
65 Grabherr, M. G. *et al.* Full-length transcriptome assembly from RNA-Seq data without a reference genome. *Nature biotechnology* **29**, 644-652 (2011).
66 Lowe, T. M. & Chan, P. P. tRNAscan-SE On-line: integrating search and context for analysis of transfer RNA genes. *Nucleic acids research* **44**, W54-W57 (2016).
67 Di Genova, A., Buena-Atienza, E., Ossowski, S. & Sagot, M.-F. Efficient hybrid de novo assembly of human genomes with WENGAN. *Nature Biotechnology* **39**, 422-430 (2021).
68 Ruan, J. & Li, H. Fast and accurate long-read assembly with wtdbg2. *Nature methods* **17**, 155-158 (2020).
69 Feng, X., Holzinger, A., Permann, C., Anderson, D. & Yin, Y. Characterization of two Zygnema strains (Zygnema circumcarinatum SAG 698-1a and SAG 698-1b) and a rapid method to estimate nuclear genome size of zygnematophycean green algae. *Frontiers in Plant Science* **12**, 610381 (2021).
70 Staker, R. D. Control of cell division in the filamentous green alga Zygnema. (1971).
71 Orton, L. M. *et al.* Zygnema circumcarinatum UTEX 1559 chloroplast and mitochondrial genomes provide insight into land plant evolution. *Journal of experimental botany* **71**, 3361-3373 (2020).
72 Zhang, M. *et al.* Preparation of megabase-sized DNA from a variety of organisms using the nuclei method for advanced genomics research. *nature protocols* **7**, 467-478 (2012).
73 Chang, S., Puryear, J. & Cairney, J. A simple and efficient method for isolating RNA from pine trees. *Plant molecular biology reporter* **11**, 113-116 (1993).
74 Bekesiova, I., Nap, J.-P. & Mlynarova, L. Isolation of high quality DNA and RNA from leaves of the carnivorous plant Drosera rotundifolia. *Plant Molecular Biology Reporter* **17**, 269 (1999).
75 Haas, B. J. *et al.* De novo transcript sequence reconstruction from RNA-seq using the Trinity platform for reference generation and analysis. *Nature protocols* **8**, 1494-1512 (2013).
76 Proost, S. & Mutwil, M. CoNekT: an open-source framework for comparative genomic and transcriptomic network analyses. *Nucleic acids research* **46**, W133-W140 (2018).
77 Mutwil, M. *et al.* Assembly of an interactive correlation network for the Arabidopsis genome using a novel heuristic clustering algorithm. *Plant physiology* **152**, 29-43 (2010).

78 Camacho, C. *et al.* BLAST+: architecture and applications. *BMC bioinformatics* **10**, 1-9 (2009).
79 Wright, E. S. DECIPHER: harnessing local sequence context to improve protein multiple sequence alignment. *BMC bioinformatics* **16**, 1-14 (2015).
80 Edgar, R. C. MUSCLE: multiple sequence alignment with high accuracy and high throughput. *Nucleic acids research* **32**, 1792-1797 (2004).
81 Minh, B. Q. *et al.* IQ-TREE 2: new models and efficient methods for phylogenetic inference in the genomic era. *Molecular biology and evolution* **37**, 1530-1534 (2020).
82 Kalyaanamoorthy, S., Minh, B. Q., Wong, T. K., Von Haeseler, A. & Jermiin, L. S. ModelFinder: fast model selection for accurate phylogenetic estimates. *Nature methods* **14**, 587-589 (2017).
83 Guindon, S. *et al.* New algorithms and methods to estimate maximum-likelihood phylogenies: assessing the performance of PhyML 3.0. *Systematic biology* **59**, 307-321 (2010).
84 Hoang, D. T., Chernomor, O., Von Haeseler, A., Minh, B. Q. & Vinh, L. S. UFBoot2: improving the ultrafast bootstrap approximation. *Molecular biology and evolution* **35**, 518-522 (2018).
85 Johnson, L. S., Eddy, S. R. & Portugaly, E. Hidden Markov model speed heuristic and iterative HMM search procedure. *BMC bioinformatics* **11**, 1-8 (2010).
86 Messing, S. A. *et al.* Structural insights into maize viviparous14, a key enzyme in the biosynthesis of the phytohormone abscisic acid. *The Plant Cell* **22**, 2970-2980 (2010).
87 Huerta-Cepas, J., Serra, F. & Bork, P. ETE 3: reconstruction, analysis, and visualization of phylogenomic data. *Molecular biology and evolution* **33**, 1635-1638 (2016).
88 Eddy, S. R. Profile hidden Markov models. *Bioinformatics* **14**, 755-763 (1998).
89 Marchant, D. B. *et al.* Dynamic genome evolution in a model fern. *Nature Plants* **8**, 1038-1051 (2022).
90 Gramzow, L., Ritz, M. S. & Theißen, G. On the origin of MADS-domain transcription factors. *Trends in Genetics* **26**, 149-153 (2010).
91 One thousand plant transcriptomes and the phylogenomics of green plants. *Nature* **574**, 679-685 (2019).
92 Sayers, E. W. *et al.* Database resources of the national center for biotechnology information. *Nucleic acids research* **49**, D10 (2021).
93 Miller, M. A., Pfeiffer, W. & Schwartz, T. in *Proceedings of the 2011 TeraGrid Conference: extreme digital discovery.* 1-8.
94 Gauch, H. G. *Studies on the life cycle and genetics of Zygnema*. (Cornell University, 1966).
95 Sola, K. *et al.* RUBY, a Putative Galactose Oxidase, Influences Pectin Properties and Promotes Cell-To-Cell Adhesion in the Seed Coat Epidermis of Arabidopsis. *Plant Cell* **31**, 809-831 (2019). https://doi.org:10.1105/tpc.18.00954
96 Furst-Jansen, J. M. R., de Vries, S. & de Vries, J. Evo-physio: on stress responses and the earliest land plants. *J Exp Bot* **71**, 3254-3269 (2020). https://doi.org:10.1093/jxb/eraa007

97 Bowles, A. M. C., Williamson, C. J., Williams, T. A., Lenton, T. M. & Donoghue, P. C. J. The origin and early evolution of plants. *Trends Plant Sci* (2022). https://doi.org:10.1016/j.tplants.2022.09.009
98 Sorensen, I. *et al.* The charophycean green algae provide insights into the early origins of plant cell walls. *Plant J* **68**, 201-211 (2011). https://doi.org:10.1111/j.1365-313X.2011.04686.x
99 Herburger, K., Glazowska, S. & Mravec, J. Bricks out of the wall: polysaccharide extramural functions. *Trends Plant Sci* **27**, 1231-1241 (2022). https://doi.org:10.1016/j.tplants.2022.07.008
100 Domozych, D. S. & Bagdan, K. The cell biology of charophytes: Exploring the past and models for the future. *Plant Physiol* **190**, 1588-1608 (2022). https://doi.org:10.1093/plphys/kiac390
101 Lombard, V., Golaconda Ramulu, H., Drula, E., Coutinho, P. M. & Henrissat, B. The carbohydrate-active enzymes database (CAZy) in 2013. *Nucleic acids research* **42**, D490-D495 (2014).
102 Mikkelsen, M. D. *et al.* Ancient origin of fucosylated xyloglucan in charophycean green algae. *Commun Biol* **4**, 754 (2021). https://doi.org:10.1038/s42003-021-02277-w
103 Mikkelsen, M. D. *et al.* Evidence for land plant cell wall biosynthetic mechanisms in charophyte green algae. *Ann Bot* **114**, 1217-1236 (2014). https://doi.org:10.1093/aob/mcu171
104 Domozych, D. S. *et al.* Pectin metabolism and assembly in the cell wall of the charophyte green alga Penium margaritaceum. *Plant Physiol* **165**, 105-118 (2014). https://doi.org:10.1104/pp.114.236257
105 Pfeifer, L. *et al.* Search for evolutionary roots of land plant arabinogalactan-proteins in charophytes: presence of a rhamnogalactan-protein in Spirogyra pratensis (Zygnematophyceae). *Plant J* **109**, 568-584 (2022). https://doi.org:10.1111/tpj.15577
106 Ma, J. *et al.* Major episodes of horizontal gene transfer drove the evolution of land plants. *Mol Plant* **15**, 857-871 (2022). https://doi.org:10.1016/j.molp.2022.02.001
107 Lampugnani, E. R. *et al.* Cellulose Synthesis - Central Components and Their Evolutionary Relationships. *Trends Plant Sci* **24**, 402-412 (2019). https://doi.org:10.1016/j.tplants.2019.02.011
108 Tsekos, I. THE SITES OF CELLULOSE SYNTHESIS IN ALGAE: DIVERSITY AND EVOLUTION OF CELLULOSE-SYNTHESIZING ENZYME COMPLEXES. *Journal of Phycology* **35**, 635-655 (1999). https://doi.org:https://doi.org/10.1046/j.1529-8817.1999.3540635.x
109 Yang, J. *et al.* Biochemical and Genetic Analysis Identify CSLD3 as a beta-1,4-Glucan Synthase That Functions during Plant Cell Wall Synthesis. *Plant Cell* **32**, 1749-1767 (2020). https://doi.org:10.1105/tpc.19.00637
110 Urbanowicz, B. R. *et al.* Structural organization and a standardized nomenclature for plant endo-1,4-beta-glucanases (cellulases) of glycosyl hydrolase family 9. *Plant Physiol* **144**, 1693-1696 (2007). https://doi.org:10.1104/pp.107.102574
111 Popper, Z. A. & Tuohy, M. G. Beyond the green: understanding the evolutionary puzzle of plant and algal cell walls. *Plant Physiol* **153**, 373-383 (2010). https://doi.org:10.1104/pp.110.158055

112 Voiniciuc, C. Modern mannan: a hemicellulose's journey. *New Phytol* **234**, 1175-1184 (2022). https://doi.org:10.1111/nph.18091
113 Yin, Y., Huang, J. & Xu, Y. The cellulose synthase superfamily in fully sequenced plants and algae. *BMC Plant Biol* **9**, 99 (2009). https://doi.org:10.1186/1471-2229-9-99
114 Yin, Y., Johns, M. A., Cao, H. & Rupani, M. A survey of plant and algal genomes and transcriptomes reveals new insights into the evolution and function of the cellulose synthase superfamily. *BMC Genomics* **15**, 260 (2014). https://doi.org:10.1186/1471-2164-15-260
115 Rodriguez-Gacio Mdel, C., Iglesias-Fernandez, R., Carbonero, P. & Matilla, A. J. Softening-up mannan-rich cell walls. *J Exp Bot* **63**, 3976-3988 (2012). https://doi.org:10.1093/jxb/ers096
116 Kim, S. J. & Brandizzi, F. Advances in Cell Wall Matrix Research with a Focus on Mixed-Linkage Glucan. *Plant Cell Physiol* **62**, 1839-1846 (2021). https://doi.org:10.1093/pcp/pcab106
117 Chang, S. C., Saldivar, R. K., Liang, P. H. & Hsieh, Y. S. Y. Structures, Biosynthesis, and Physiological Functions of (1,3;1,4)-beta-D-Glucans. *Cells* **10** (2021). https://doi.org:10.3390/cells10030510
118 Samar, D., Kieler, J. B. & Klutts, J. S. Identification and deletion of Tft1, a predicted glycosyltransferase necessary for cell wall beta-1,3;1,4-glucan synthesis in Aspergillus fumigatus. *PLoS One* **10**, e0117336 (2015). https://doi.org:10.1371/journal.pone.0117336
119 Perez-Mendoza, D. *et al.* Novel mixed-linkage beta-glucan activated by c-di-GMP in Sinorhizobium meliloti. *Proc Natl Acad Sci U S A* **112**, E757-765 (2015). https://doi.org:10.1073/pnas.1421748112
120 Roberts, A. W. *et al.* Functional Characterization of a Glycosyltransferase from the Moss Physcomitrella patens Involved in the Biosynthesis of a Novel Cell Wall Arabinoglucan. *Plant Cell* **30**, 1293-1308 (2018). https://doi.org:10.1105/tpc.18.00082
121 Blum, M., Waldner, M. & Gisi, U. A single point mutation in the novel PvCesA3 gene confers resistance to the carboxylic acid amide fungicide mandipropamid in Plasmopara viticola. *Fungal Genet Biol* **47**, 499-510 (2010). https://doi.org:10.1016/j.fgb.2010.02.009
122 Blanton, R. L., Fuller, D., Iranfar, N., Grimson, M. J. & Loomis, W. F. The cellulose synthase gene of Dictyostelium. *Proc Natl Acad Sci U S A* **97**, 2391-2396 (2000). https://doi.org:10.1073/pnas.040565697
123 Matthews, P. R., Schindler, M., Howles, P., Arioli, T. & Williamson, R. E. A CESA from Griffithsia monilis (Rhodophyta, Florideophyceae) has a family 48 carbohydrate-binding module. *J Exp Bot* **61**, 4461-4468 (2010). https://doi.org:10.1093/jxb/erq254
124 Matthysse, A. G. *et al.* A functional cellulose synthase from ascidian epidermis. *Proc Natl Acad Sci U S A* **101**, 986-991 (2004). https://doi.org:10.1073/pnas.0303623101
125 Nobles, D. R., Romanovicz, D. K. & Brown, R. M., Jr. Cellulose in cyanobacteria. Origin of vascular plant cellulose synthase? *Plant Physiol* **127**, 529-542 (2001).
126 Zhao, C. *et al.* High-yield production of extracellular type-I cellulose by the cyanobacterium Synechococcus sp. PCC 7002. *Cell Discov* **1**, 15004 (2015). https://doi.org:10.1038/celldisc.2015.4

127 Romling, U. & Galperin, M. Y. Bacterial cellulose biosynthesis: diversity of operons, subunits, products, and functions. *Trends Microbiol* **23**, 545-557 (2015). https://doi.org:10.1016/j.tim.2015.05.005
128 Jenal, U., Reinders, A. & Lori, C. Cyclic di-GMP: second messenger extraordinaire. *Nat Rev Microbiol* **15**, 271-284 (2017). https://doi.org:10.1038/nrmicro.2016.190
129 Abidi, W., Torres-Sanchez, L., Siroy, A. & Krasteva, P. V. Weaving of bacterial cellulose by the Bcs secretion systems. *FEMS Microbiol Rev* **46** (2022). https://doi.org:10.1093/femsre/fuab051
130 Galloway, A. F. *et al.* Xyloglucan is released by plants and promotes soil particle aggregation. *New Phytol* **217**, 1128-1136 (2018). https://doi.org:10.1111/nph.14897
131 Telagathoti, A., Probst, M. & Peintner, U. Habitat, Snow-Cover and Soil pH, Affect the Distribution and Diversity of Mortierellaceae Species and Their Associations to Bacteria. *Front Microbiol* **12**, 669784 (2021). https://doi.org:10.3389/fmicb.2021.669784
132 Shinohara, N. & Nishitani, K. Cryogenian Origin and Subsequent Diversification of the Plant Cell-Wall Enzyme XTH Family. *Plant Cell Physiol* **62**, 1874-1889 (2021). https://doi.org:10.1093/pcp/pcab093
133 Gunl, M. *et al.* AXY8 encodes an alpha-fucosidase, underscoring the importance of apoplastic metabolism on the fine structure of Arabidopsis cell wall polysaccharides. *Plant Cell* **23**, 4025-4040 (2011). https://doi.org:10.1105/tpc.111.089193
134 Wu, Y. *et al.* Functional identification of two nonredundant Arabidopsis alpha(1,2)fucosyltransferases specific to arabinogalactan proteins. *J Biol Chem* **285**, 13638-13645 (2010). https://doi.org:10.1074/jbc.M110.102715
135 Del-Bem, L. E. Xyloglucan evolution and the terrestrialization of green plants. *New Phytol* **219**, 1150-1153 (2018). https://doi.org:10.1111/nph.15191
136 Piršelová, B. & Matušíková, I. Callose: the plant cell wall polysaccharide with multiple biological functions. *Acta Physiologiae Plantarum* **35**, 635-644 (2013). https://doi.org:10.1007/s11738-012-1103-y
137 Davis, D. J. *et al.* Callose deposition is essential for the completion of cytokinesis in the unicellular alga Penium margaritaceum. *J Cell Sci* **133** (2020). https://doi.org:10.1242/jcs.249599
138 Scherp, P., Grotha, R. & Kutschera, U. Occurrence and phylogenetic significance of cytokinesis-related callose in green algae, bryophytes, ferns and seed plants. *Plant Cell Reports* **20**, 143-149 (2001). https://doi.org:10.1007/s002990000301
139 Gaudioso-Pedraza, R. & Benitez-Alfonso, Y. A phylogenetic approach to study the origin and evolution of plasmodesmata-localized glycosyl hydrolases family 17. *Front Plant Sci* **5**, 212 (2014). https://doi.org:10.3389/fpls.2014.00212
140 Aspeborg, H., Coutinho, P. M., Wang, Y., Brumer, H., 3rd & Henrissat, B. Evolution, substrate specificity and subfamily classification of glycoside hydrolase family 5 (GH5). *BMC Evol Biol* **12**, 186 (2012). https://doi.org:10.1186/1471-2148-12-186
141 Liang, D., Andersen, C. B., Vetukuri, R. R., Dou, D. & Grenville-Briggs, L. J. Horizontal Gene Transfer and Tandem Duplication Shape the Unique CAZyme Complement of the Mycoparasitic Oomycetes Pythium oligandrum and Pythium periplocum. *Front Microbiol* **11**, 581698 (2020). https://doi.org:10.3389/fmicb.2020.581698

142 Yin, Y. *et al.* dbCAN: a web resource for automated carbohydrate-active enzyme annotation. *Nucleic Acids Res* **40**, W445-451 (2012). https://doi.org:10.1093/nar/gks479
143 Zhong, R., Cui, D. & Ye, Z. H. Secondary cell wall biosynthesis. *New Phytol* **221**, 1703-1723 (2019). https://doi.org:10.1111/nph.15537
144 Jensen, J. K. *et al.* Identification of an algal xylan synthase indicates that there is functional orthology between algal and plant cell wall biosynthesis. *New Phytol* **218**, 1049-1060 (2018). https://doi.org:10.1111/nph.15050
145 Taujale, R. & Yin, Y. Glycosyltransferase family 43 is also found in early eukaryotes and has three subfamilies in Charophycean green algae. *PLoS One* **10**, e0128409 (2015). https://doi.org:10.1371/journal.pone.0128409
146 Anders, N. *et al.* Glycosyl transferases in family 61 mediate arabinofuranosyl transfer onto xylan in grasses. *Proc Natl Acad Sci U S A* **109**, 989-993 (2012). https://doi.org:10.1073/pnas.1115858109
147 Seifert, G. J. & Roberts, K. The biology of arabinogalactan proteins. *Annu Rev Plant Biol* **58**, 137-161 (2007). https://doi.org:10.1146/annurev.arplant.58.032806.103801
148 Happ, K. & Classen, B. Arabinogalactan-Proteins from the Liverwort Marchantia polymorpha L., a Member of a Basal Land Plant Lineage, Are Structurally Different to Those of Angiosperms. *Plants (Basel)* **8** (2019). https://doi.org:10.3390/plants8110460
149 Silva, J., Ferraz, R., Dupree, P., Showalter, A. M. & Coimbra, S. Three Decades of Advances in Arabinogalactan-Protein Biosynthesis. *Front Plant Sci* **11**, 610377 (2020). https://doi.org:10.3389/fpls.2020.610377
150 Knoch, E., Dilokpimol, A. & Geshi, N. Arabinogalactan proteins: focus on carbohydrate active enzymes. *Front Plant Sci* **5**, 198 (2014). https://doi.org:10.3389/fpls.2014.00198
151 Atmodjo, M. A., Hao, Z. & Mohnen, D. Evolving views of pectin biosynthesis. *Annu Rev Plant Biol* **64**, 747-779 (2013). https://doi.org:10.1146/annurev-arplant-042811-105534
152 Amos, R. A. *et al.* Polymerization of the backbone of the pectic polysaccharide rhamnogalacturonan I. *Nat Plants* **8**, 1289-1303 (2022). https://doi.org:10.1038/s41477-022-01270-3
153 Takenaka, Y. *et al.* Pectin RG-I rhamnosyltransferases represent a novel plant-specific glycosyltransferase family. *Nat Plants* **4**, 669-676 (2018). https://doi.org:10.1038/s41477-018-0217-7
154 Ebert, B. *et al.* The Three Members of the Arabidopsis Glycosyltransferase Family 92 are Functional beta-1,4-Galactan Synthases. *Plant Cell Physiol* **59**, 2624-2636 (2018). https://doi.org:10.1093/pcp/pcy180
155 Ishimaru, M., Smith, D. L., Mort, A. J. & Gross, K. C. Enzymatic activity and substrate specificity of recombinant tomato beta-galactosidases 4 and 5. *Planta* **229**, 447-456 (2009). https://doi.org:10.1007/s00425-008-0842-x
156 Dumont, M. *et al.* The cell wall pectic polymer rhamnogalacturonan-II is required for proper pollen tube elongation: implications of a putative sialyltransferase-like protein. *Ann Bot* **114**, 1177-1188 (2014). https://doi.org:10.1093/aob/mcu093
157 Park, K.-C., Kwon, S.-J. & Kim, N.-S. Intron loss mediated structural dynamics and functional differentiation of the polygalacturonase gene family in land plants. *Genes & Genomics* **32**, 570-577 (2010). https://doi.org:10.1007/s13258-010-0076-8

158 Yang, Y., Yu, Y., Liang, Y., Anderson, C. T. & Cao, J. A Profusion of Molecular Scissors for Pectins: Classification, Expression, and Functions of Plant Polygalacturonases. *Front Plant Sci* **9**, 1208 (2018). https://doi.org:10.3389/fpls.2018.01208
159 Domingo, C. *et al.* A pectate lyase from Zinnia elegans is auxin inducible. *Plant J* **13**, 17-28 (1998). https://doi.org:10.1046/j.1365-313x.1998.00002.x
160 Marin-Rodriguez, M. C., Smith, D. L., Manning, K., Orchard, J. & Seymour, G. B. Pectate lyase gene expression and enzyme activity in ripening banana fruit. *Plant Mol Biol* **51**, 851-857 (2003). https://doi.org:10.1023/a:1023057202847
161 Xie, F. *et al.* Legume pectate lyase required for root infection by rhizobia. *Proc Natl Acad Sci U S A* **109**, 633-638 (2012). https://doi.org:10.1073/pnas.1113992109
162 Han, J. W. & Kim, G. H. An ELIP-like gene in the freshwater green alga, Spirogyra varians (Zygnematales), is regulated by cold stress and CO2 influx. *Journal of applied phycology* **25**, 1297-1307 (2013).
163 Crozet, P. *et al.* Mechanisms of regulation of SNF1/AMPK/SnRK1 protein kinases. *Frontiers in plant science* **5**, 190 (2014).
164 Feiguelman, G., Fu, Y. & Yalovsky, S. ROP GTPases structure-function and signaling pathways. *Plant physiology* **176**, 57-79 (2018).
165 Yamanaka, T. *et al.* Complete inhibition of tobamovirus multiplication by simultaneous mutations in two homologous host genes. *Journal of virology* **76**, 2491-2497 (2002).
166 Van de Poel, B., Cooper, E. D., Van Der Straeten, D., Chang, C. & Delwiche, C. F. Transcriptome profiling of the green alga Spirogyra pratensis (Charophyta) suggests an ancestral role for ethylene in cell wall metabolism, photosynthesis, and abiotic stress responses. *Plant Physiology* **172**, 533-545 (2016).
167 Williams, M. E. *et al.* Mutations in the Arabidopsis phosphoinositide phosphatase gene SAC9 lead to overaccumulation of PtdIns (4, 5) P2 and constitutive expression of the stress-response pathway. *Plant physiology* **138**, 686-700 (2005).
168 Morant, A. V. *et al.* β-Glucosidases as detonators of plant chemical defense. *Phytochemistry* **69**, 1795-1813 (2008).
169 Ma, B. *et al.* Membrane protein MHZ3 stabilizes OsEIN2 in rice by interacting with its Nramp-like domain. *Proceedings of the National Academy of Sciences* **115**, 2520-2525 (2018).
170 Wunder, T. *et al.* The major thylakoid protein kinases STN7 and STN8 revisited: effects of altered STN8 levels and regulatory specificities of the STN kinases. *Frontiers in plant science* **4**, 417 (2013).
171 Bracher, A., Sharma, A., Starling-Windhof, A., Hartl, F. U. & Hayer-Hartl, M. Degradation of potent Rubisco inhibitor by selective sugar phosphatase. *Nature plants* **1**, 1-7 (2015).
172 Xiao, P., Feng, J.-W., Zhu, X.-T. & Gao, J. Evolution analyses of CAMTA transcription factor in plants and its enhancing effect on cold-Tolerance. *Frontiers in Plant Science* **12**, 758187 (2021).
173 de Vries, J. *et al.* Heat stress response in the closest algal relatives of land plants reveals conserved stress signaling circuits. *The Plant Journal* **103**, 1025-1048 (2020).
174 Brenner, W. G. & Schmülling, T. Summarizing and exploring data of a decade of cytokinin-related transcriptomics. *Frontiers in Plant Science* **6**, 29 (2015).

175 Bowman, J. L., Briginshaw, L. N., Fisher, T. J. & Flores-Sandoval, E. Something ancient and something neofunctionalized—evolution of land plant hormone signaling pathways. *Current Opinion in Plant Biology* **47**, 64-72 (2019).
176 Ju, C. *et al.* Conservation of ethylene as a plant hormone over 450 million years of evolution. *Nature plants* **1**, 14004 (2015).
177 Cuming, A. C., Cho, S. H., Kamisugi, Y., Graham, H. & Quatrano, R. S. Microarray analysis of transcriptional responses to abscisic acid and osmotic, salt, and drought stress in the moss, Physcomitrella patens. *New Phytologist* **176**, 275-287 (2007).
178 Umezawa, T. *et al.* Molecular basis of the core regulatory network in ABA responses: sensing, signaling and transport. *Plant and cell physiology* **51**, 1821-1839 (2010).
179 Eklund, D. M. *et al.* An evolutionarily conserved abscisic acid signaling pathway regulates dormancy in the liverwort Marchantia polymorpha. *Current Biology* **28**, 3691-3699. e3693 (2018).
180 Sun, Y. *et al.* A ligand-independent origin of abscisic acid perception. *Proceedings of the National Academy of Sciences* **116**, 24892-24899 (2019).
181 Jia, K.-P. *et al.* An alternative, zeaxanthin epoxidase-independent abscisic acid biosynthetic pathway in plants. *Molecular Plant* **15**, 151-166 (2022).
182 Adamowski, M. & Friml, J. PIN-dependent auxin transport: action, regulation, and evolution. *The Plant Cell* **27**, 20-32 (2015).
183 Friml, J. *et al.* Efflux-dependent auxin gradients establish the apical–basal axis of Arabidopsis. *Nature* **426**, 147-153 (2003).
184 Weijers, D. & Wagner, D. Transcriptional responses to the auxin hormone. *Annual review of plant biology* **67**, 539-574 (2016).
185 Flores-Sandoval, E., Romani, F. & Bowman, J. L. Co-expression and transcriptome analysis of Marchantia polymorpha transcription factors supports class C ARFs as independent actors of an ancient auxin regulatory module. *Frontiers in Plant Science* **9**, 1345 (2018).
186 Mutte, S. K. *et al.* Origin and evolution of the nuclear auxin response system. *Elife* **7**, e33399 (2018).
187 Martin-Arevalillo, R. *et al.* Evolution of the auxin response factors from charophyte ancestors. *PLoS Genetics* **15**, e1008400 (2019).
188 Żabka, A. *et al.* PIN2-like proteins may contribute to the regulation of morphogenetic processes during spermatogenesis in Chara vulgaris. *Plant cell reports* **35**, 1655-1669 (2016).
189 Skokan, R. *et al.* PIN-driven auxin transport emerged early in streptophyte evolution. *Nature Plants* **5**, 1114-1119 (2019).
190 Vosolsobě, S., Skokan, R. & Petrášek, J. The evolutionary origins of auxin transport: what we know and what we need to know. *Journal of experimental botany* **71**, 3287-3295 (2020).
191 Yue, J., Hu, X. & Huang, J. Origin of plant auxin biosynthesis. *Trends in plant science* **19**, 764-770 (2014).
192 Proust, H. *et al.* Strigolactones regulate protonema branching and act as a quorum sensing-like signal in the moss Physcomitrella patens. *Development* **138**, 1531-1539 (2011).

193 Delaux, P.-M. *et al.* Origin of strigolactones in the green lineage. *New Phytologist* **195**, 857-871 (2012).
194 Waters, M. T., Gutjahr, C., Bennett, T. & Nelson, D. C. Strigolactone signaling and evolution. *Annual review of plant biology* **68**, 291-322 (2017).
195 Kodama, K. *et al.* An ancestral function of strigolactones as symbiotic rhizosphere signals. *Nature communications* **13**, 3974 (2022).
196 Smýkalová, I. *et al.* Green microalga Trebouxia sp. produces strigolactone-related compounds. *bioRxiv*, 195883 (2017).

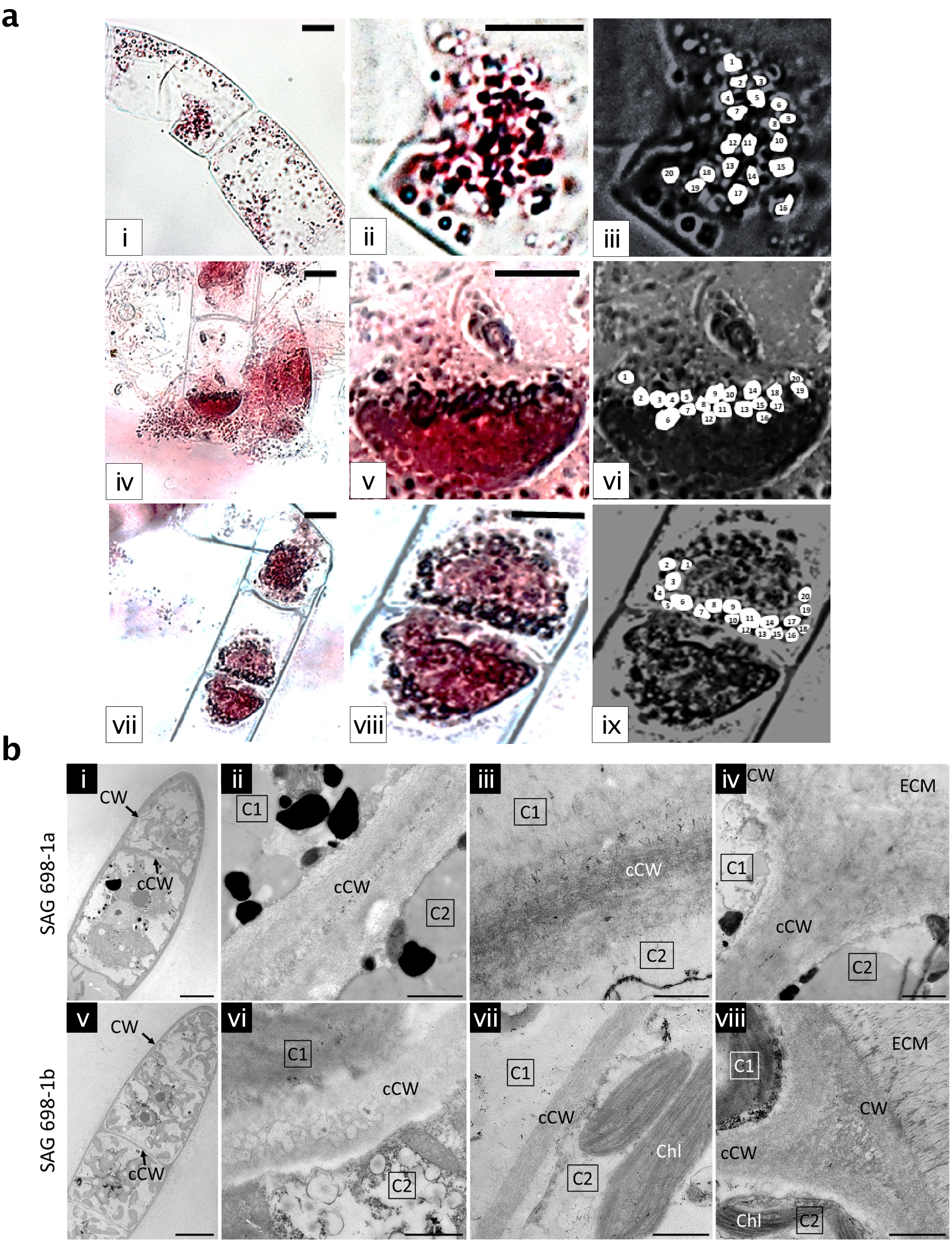

Supplementary Figure 1. (**a**) Light micrographs of *Zygnema circumcarinatum* SAG 698-1b fixed and stained with acetocarmine. (i-iii) are slides without crush preparation, (iv-ix) are crush prepared slides. (i) overview of prophase, (ii) detail from A with chromosomes visible, (iii) counted chromosomes from ii represented as sketches, (iv) overview of metaphase, (v) detail from iv with chromosomes visible, (vi) counted chromosomes from v represented as sketches, (vii) overview of telophase, (viii) detail from vii with chromosomes visible, (ix) counted chromosomes from viii represented as sketches. Scale bars: 10 µm. (**b**) Transmission electron micrographs of Zygnema cylindricum (SAG 698-1a i-iv) and Zygnema cricumcarinatum (SAG 698-1b, v-viii). The images show connected cells within a filament surrounded by a cell wall (CW) and a thin cross cell wall (cCW) connecting the cells within a filament (i, v). In panels B-D details of this cCW are illustrated, the two separated cells within a filament are marked by C1 and C2. At the connection site both cell walls are clearly visible, separated by a distinct middle part (ii, iii), and on the corners this structure is broadened (iv). In strains 698-1b the cross walls are very thin (vi, vii) containing loose material to connect them (vi) or only one cCW is visible (vii), in the corner parts the surrounding cell wall and the extracellular matrix (ECM) are clearly visible. Scale bars i, v: 10 µm, ii-iv, vi-viii 1 µm. A minimum of 15 algal filaments each for two biological replicates (cell cultures) were analyzed.

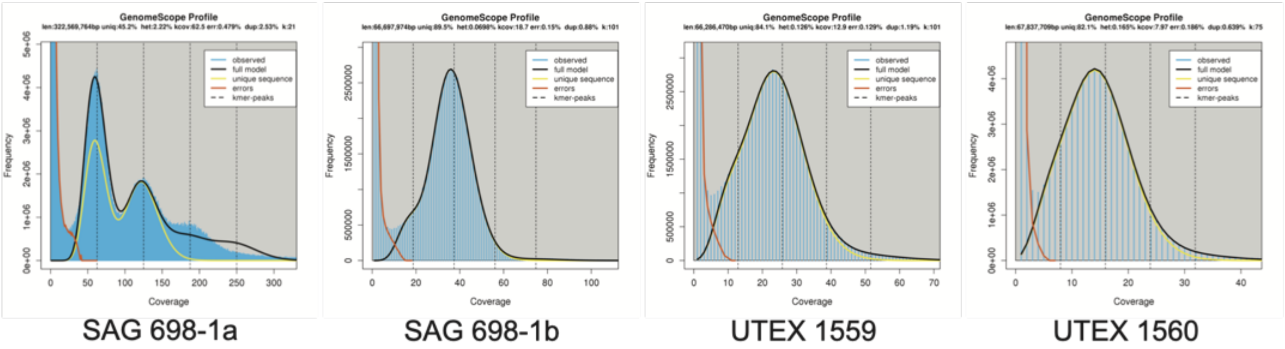

Supplementary Figure 2. Genome size estimation of genomes of *Zygnema* cf. *cylindricum* SAG 698-1a_XF, *Z. circumcarinatum* SAG 698-1b, UTEX 1559 and UTEX 1560 with k-mer analysis. Analysis details are described in the Methods. The input data is given in Table S1B.

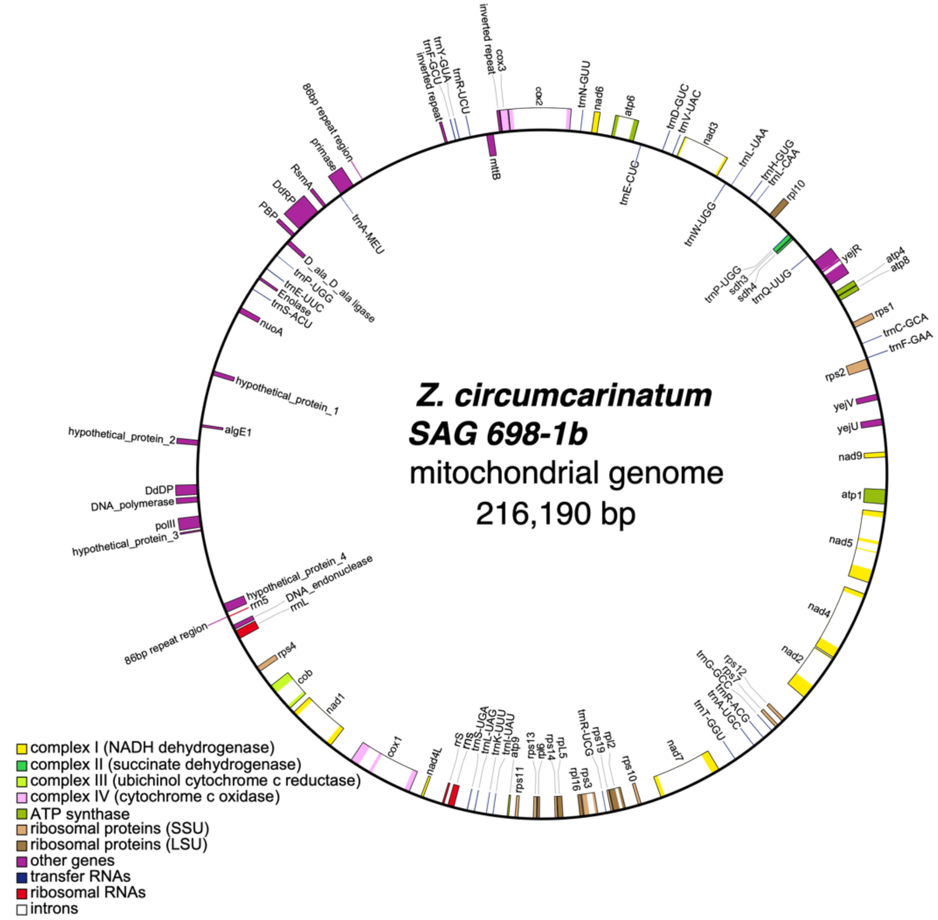

Supplementary Figure 3. Gene annotation of *Zygnema circumcarinatum* SAG 698-1b mitogenome. Analysis details are described in the Methods. The UTEX 1560 mitogenome is identical to that of SAG 698-1b, and slightly different from that of UTEX 1559 (MT040698, 215,954 bp).

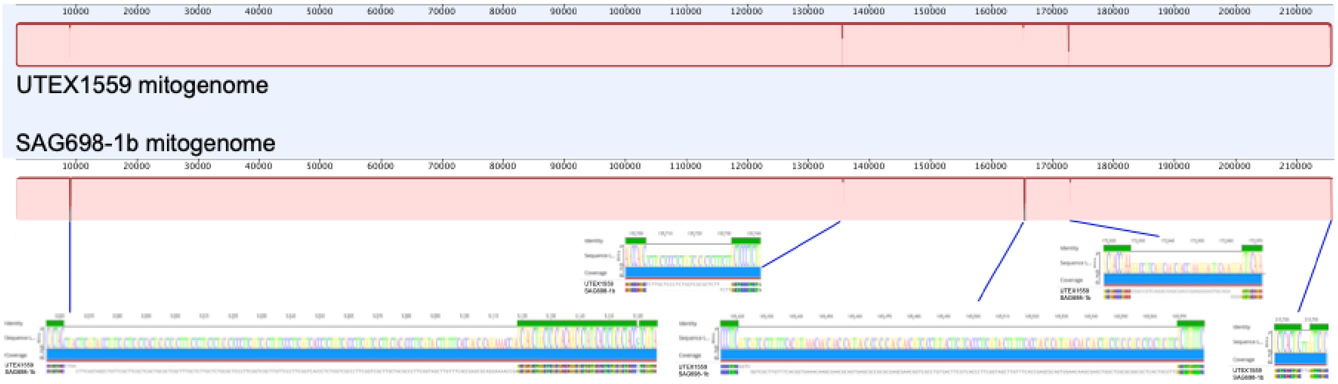

Supplementary Figure 4. Mauve alignment of mitogenomes of UTEX 1559 (top) and SAG 698-1b (bottom). The five different regions are shown with enlarged views.

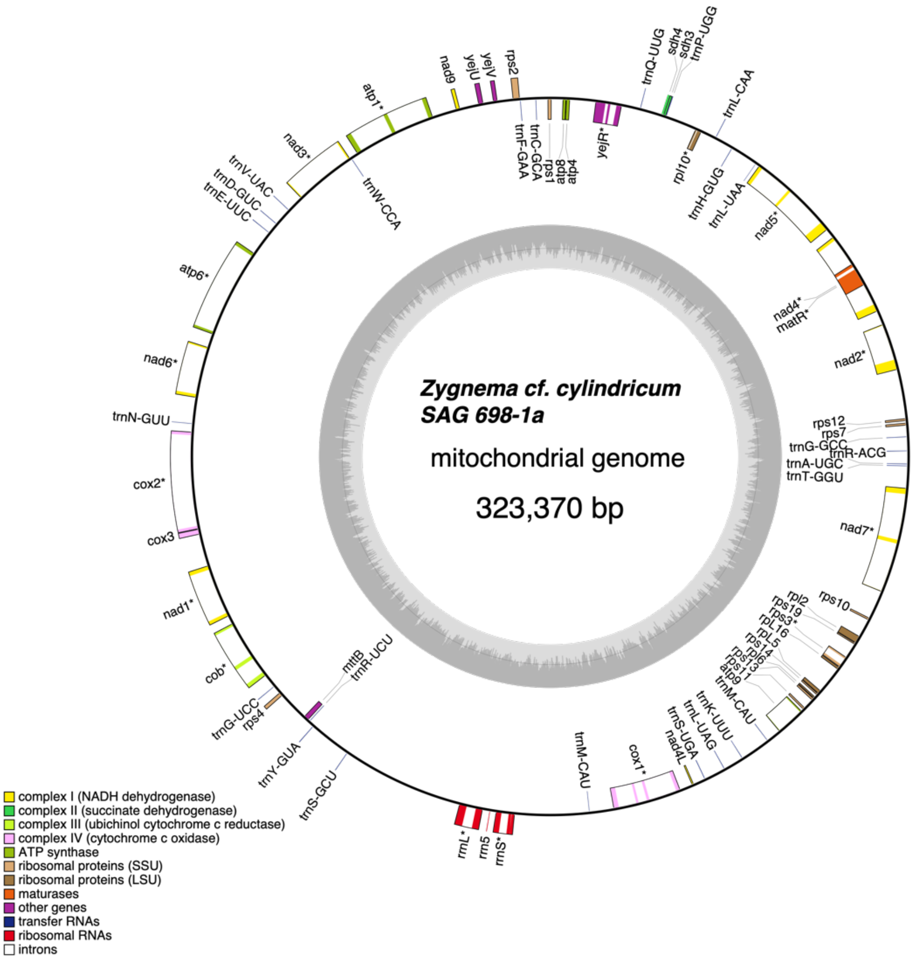

Supplementary Figure 5. Gene annotation of *Zygnema* cf. *cylindricum* SAG 698-1a_XF mitogenome. Analysis details are described in the Methods. The mitogenome comparison between SAG 698-1a_XF and SAG 698-1b is given in Table S1G,H.

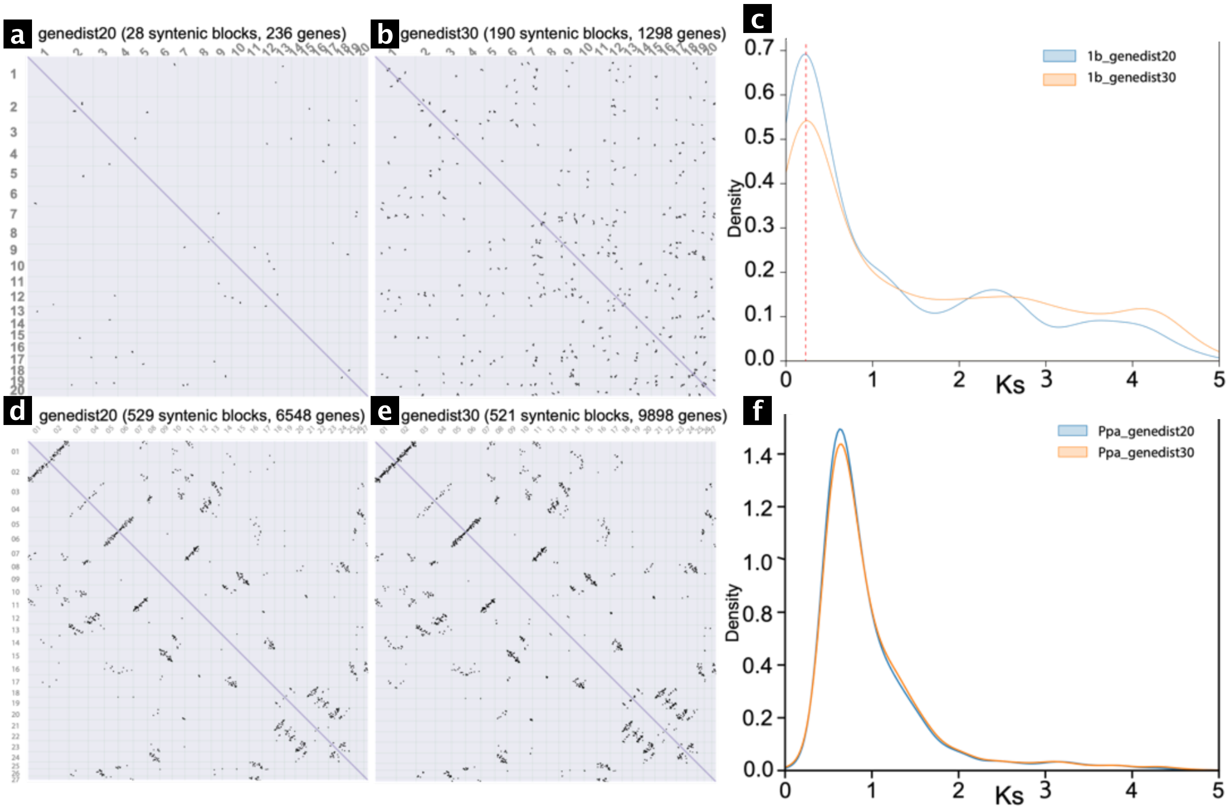

Supplementary Figure 6. Polyploidy analysis of SAG 698-1b chromosome-level genome and comparison with *Physcomitrium patens*. (a) Dot plot of syntenic blocks in SAG 698-1b genome. The syntenic block regions were identified by MCscan with the parameter that the distance between two colinear genes within a syntenic block < 20 genes. All the paralogous genes were identified using protein RBBH (reciprocal best BLASTP hits) with E-value < 10-6. (b) Dot plot of syntenic blocks in SAG 698-1b genome. The syntenic block regions were identified by MCscan with the parameter that the distance between two colinear genes within a syntenic block < 30 genes. (c) The Ks distribution of all paralog RBBH pairs in SAG 698-1b genome. (d) Dot plot of syntenic blocks in *P. patens* genome with distance threshold < 20 genes. (e) Dot plot of syntenic blocks in P. patens genome with distance threshold < 30 genes. (f) The Ks distribution of all paralog RBBH (Reciprocal Best Blast Hit) pairs in the *P. patens* genome.

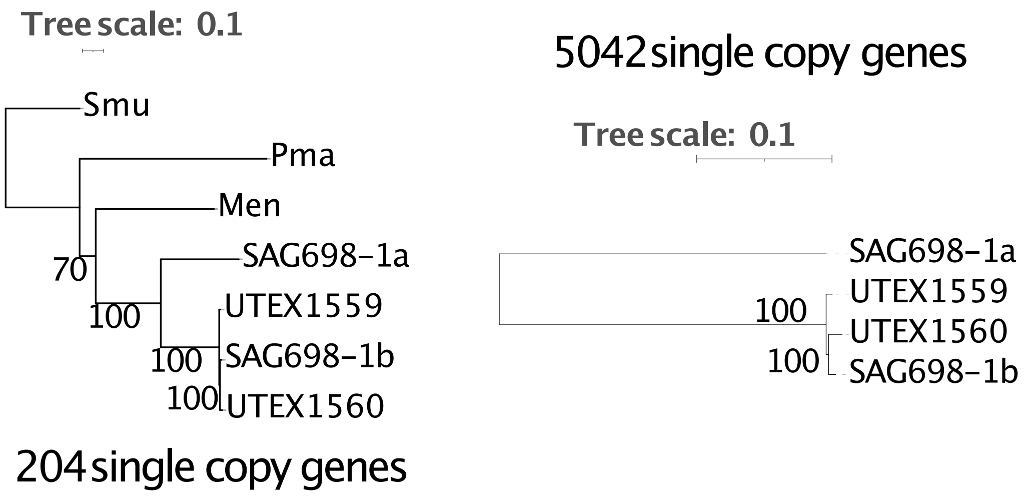

Supplementary Figure 7. Maximum likelihood trees inferred from orthogroups obtained from Zygnematophyceae (left) and *Zygnema* (right) genomes. The numbers of single copy orthogroups used in the phylogenetic reconstructions are indicated. Analysis details are described in the Methods.

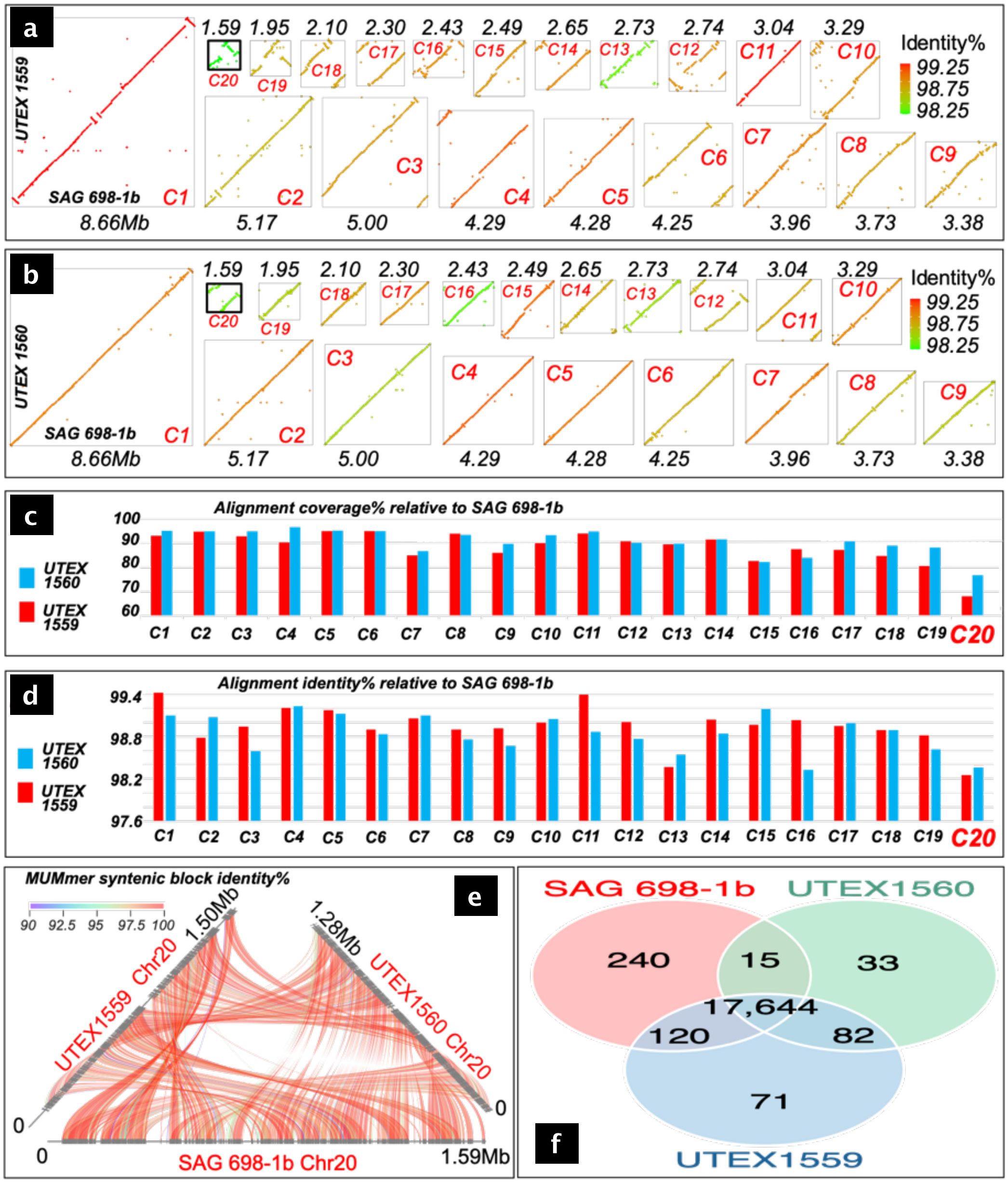

Supplementary Figure 8. Comparisons of the three *Z. circumcarinatum* chromosome-level genomes. (**a**) Dot plots of NUCmer (MUMMER) identified colinear DNA blocks (size > 1000bp) between SAG 698-1b (x-axis) and UTEX 1559 (y-axis) for all the 20 chromosomes. The dots are colored based on the average NUCmer calculated nucleotide identity of all blocks in the chromosomes (Chr). Chromosome lengths are shown for SAG 698-1b (Mb). Chromosomes are drawn in proportion to their lengths. (**b**) Same as A but for between SAG 698-1b (x-axis) and UTEX 1560 (y-axis) comparisons. (**c**) Alignment coverage (the summed length of colinear DNA blocks divided by the total length of the chromosome). (**d**) The average alignment identity of all NUCmer aligned colinear DNA blocks of the chromosome. (**e**) The 3-way colinear plot of all NUCmer colinear DNA blocks of the chromosome 20 among the three genomes. (**f**) Venn diagram of shared genes.

the counts of core genes, shell genes, and unique genes from the gene content comparisons among the three genomes. Details are provided in the Methods.

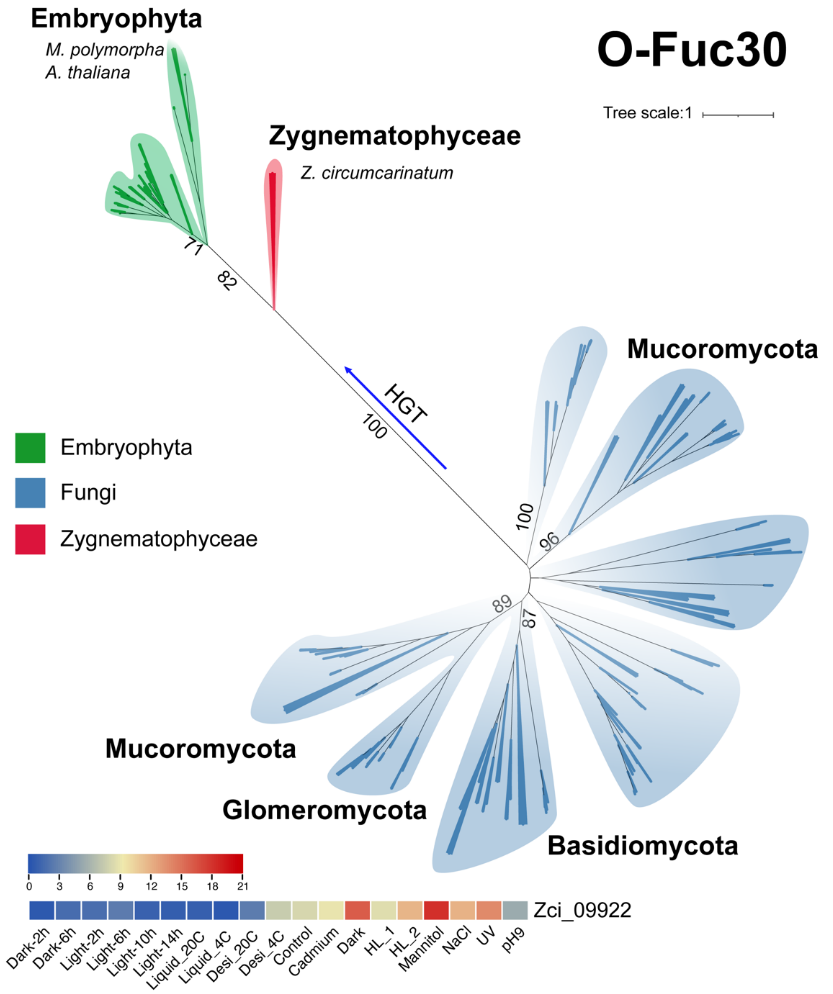

Supplementary Figure 9. Phylogeny of the O-FucT family. The Pfam O-FucT domain (PF10250) was used to search against NCBI-nr database (E-value < 1e-10) to gather homologs, which were combined with homologs in the 16 genomes to build the tree. Zci_09922 contains a conserved O-FucT (GDP-fucose protein O-fucosyltransferase) domain (Pfam: PF10250), and was identified as a HGT candidate from fungi (Fig. 4A). The Pfam search of O-FucT found in total Interestingly, under drought and cold conditions, the expression levels of this gene (Zci_09922) were upregulated, indicating that it was involved in response to stresses. Furthermore, our previous results showed that the fucose metabolism genes were enriched in Zygnema (Fitzek et al., 2019). In *Arabidopsis*, the O-FucT involved in cell wall integrity and cell adhesion (Verger et al., 2016). This acquired gene from fungi might facilitate the algae in adaption in extreme environments, such as drought, UV radiation.

a

b

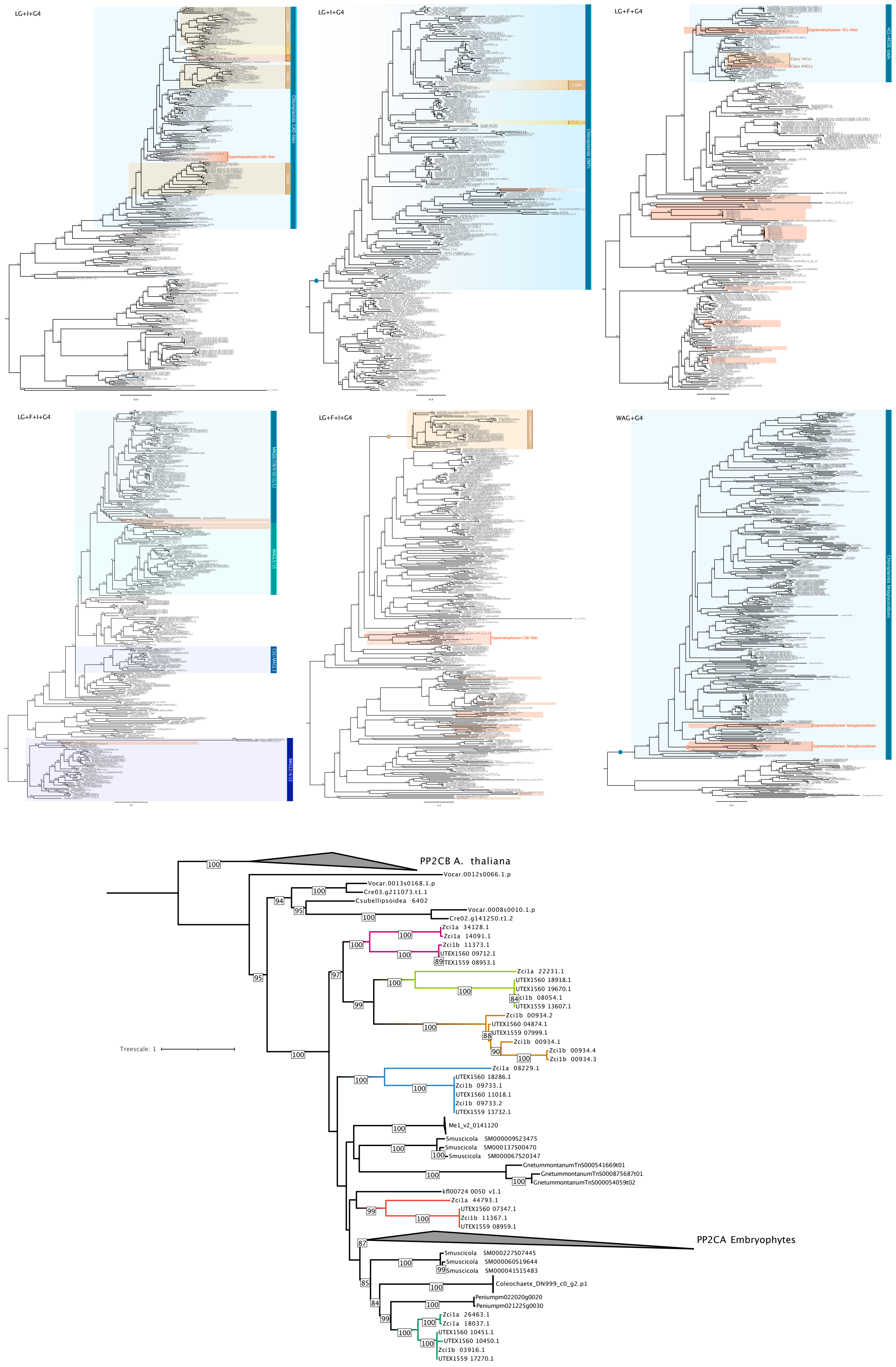

Supplementary Figure 10. Maximum likelihood phylogenies of (**a**) genes salient to the production of phenylpropanoid-derived specialized metabolites. (**b**) PP2C homologs. Best-fit models of protein evolution are noted in the top left corner.

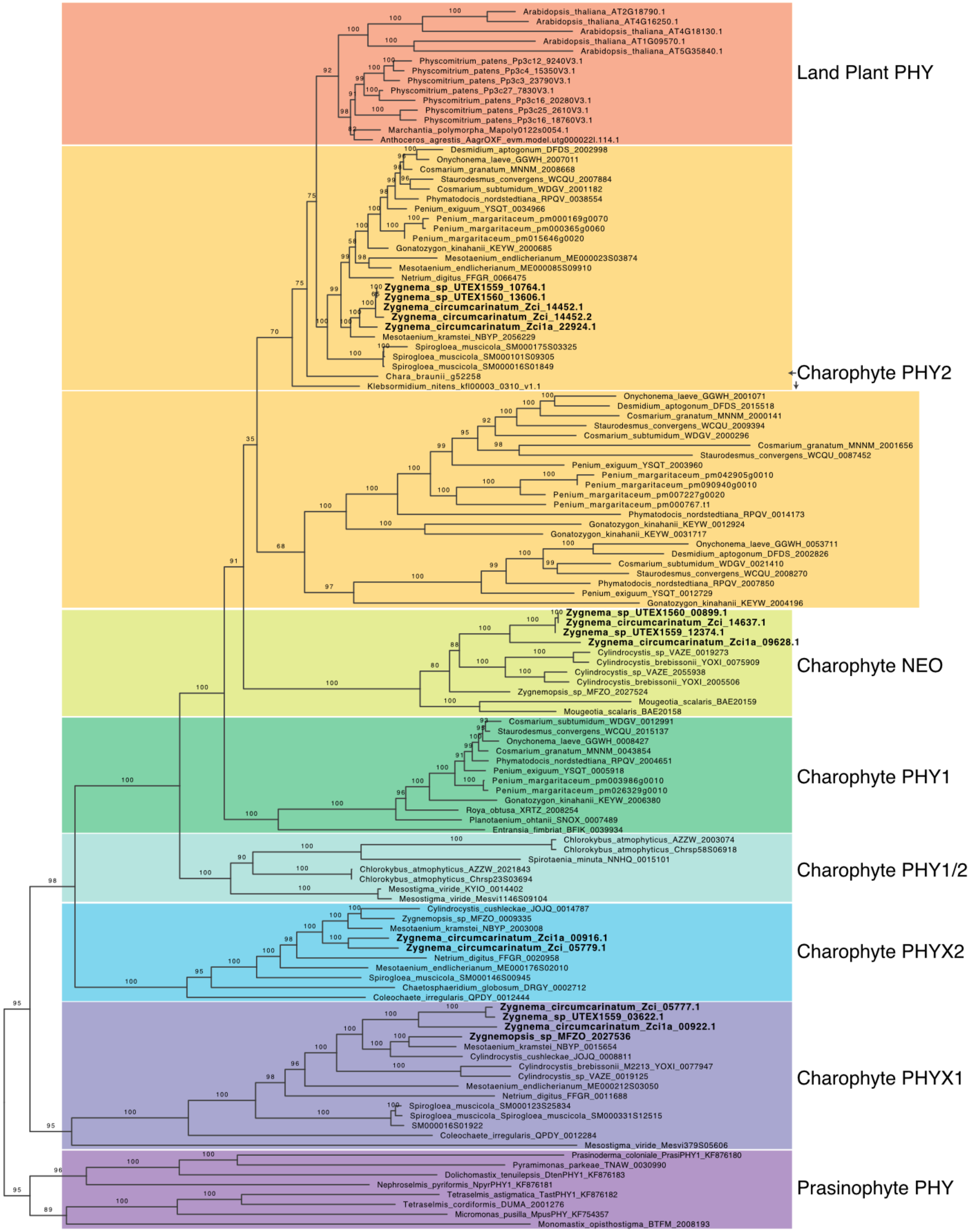

Supplementary Figure 11. Phylogeny of phytochromes. The *Zygnema* sequences from this study were bolded. The dataset and clade nomenclature were derived from Li et al., 2015

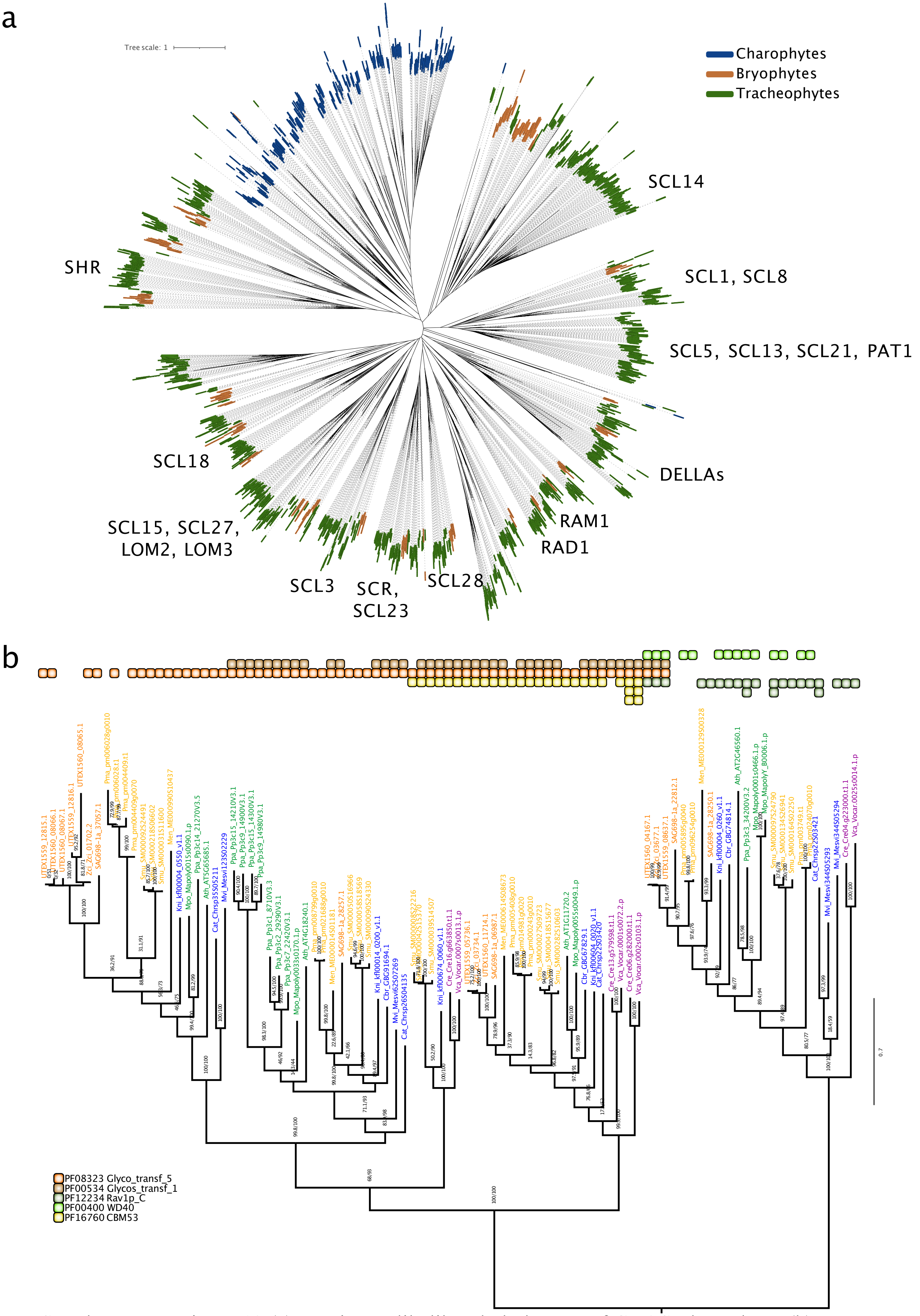

Supplementary Figure 12.(a) Maximum likelihood phylogeny of GRAS homologs. (b) Phylogenetic tree of the transducin / WD-40 repeat family protein orthogroup and the corresponding protein domain architecture for each sequence

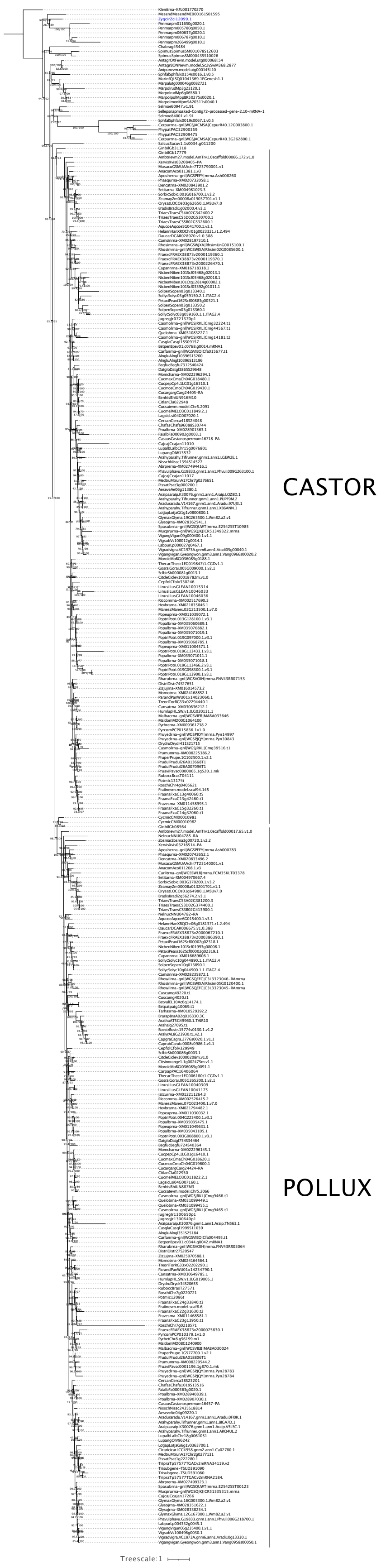

Supplementary Figure 13. Maximum likelihood phylogeny of CASTOR and POLLUX homologs.

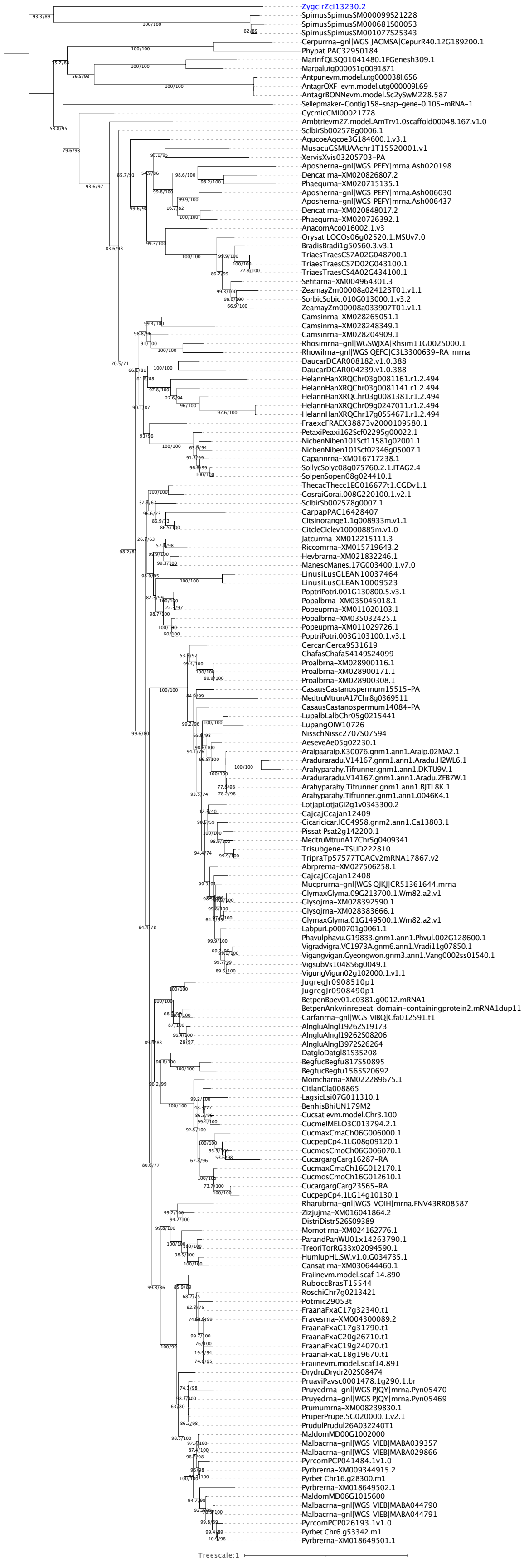

Supplementary Figure 14. Maximum likelihood phylogeny of CYCLOPS homologs

Supplementary Figure 15. Maximum likelihood phylogenies CCAMK homologs

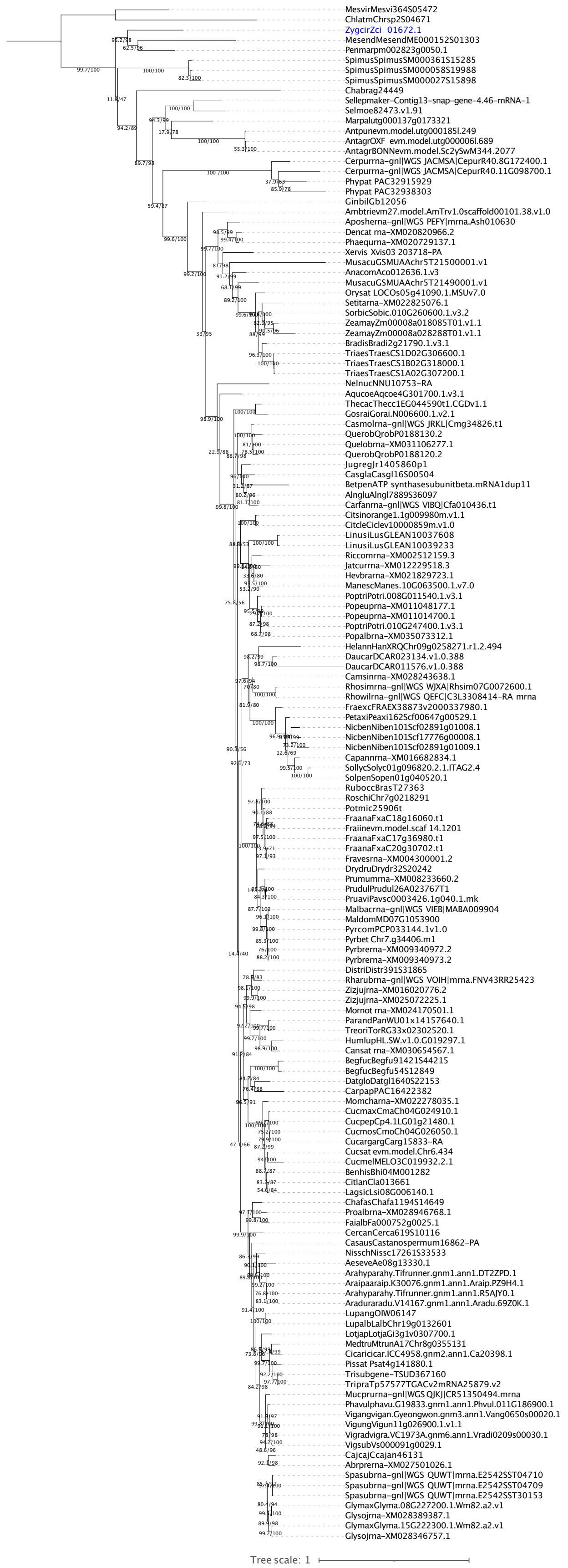

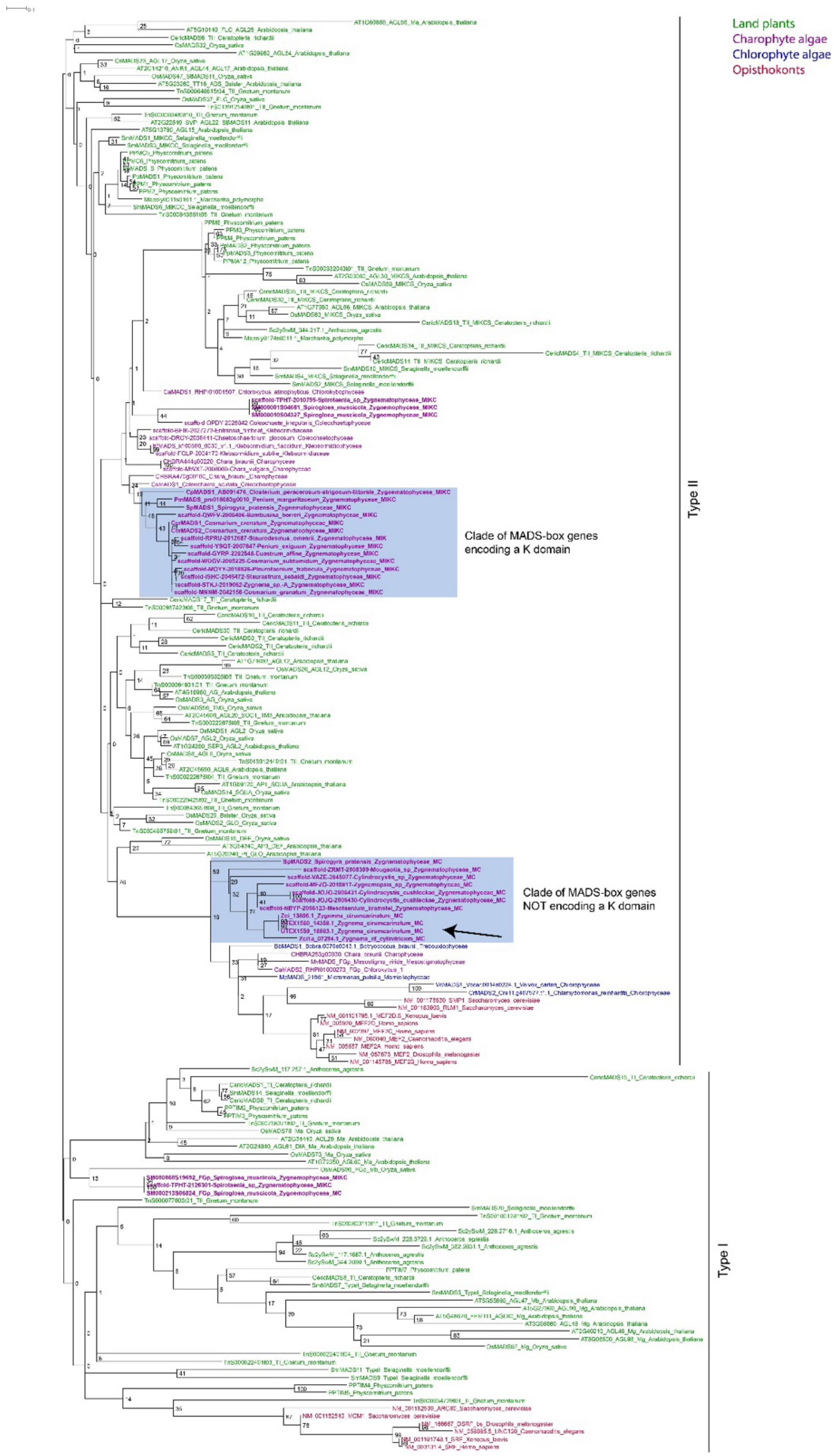

Supplementary Figure 16. Maximum Likelihood phylogeny of MADS-domain proteins. The phylogeny was reconstructed using RAxML. Names of proteins are colored as follows: green, proteins of land plants, purple, proteins of charophytes, blue, proteins of chlorophytes, red, proteins of opisthokonts. The two major clades of Type II MADS-domain proteins in Zygnematophyceae, one comprised of proteins containing the K domain and the other including proteins without a K domain, are highlighted by shading. The positions of the MADS-domain proteins of the *Zygnema* genomes sequenced here is indicated by an arrow. The clades of Type I and Type II MADS-domain proteins is indicated on the right. Bootstrap values are given on the nodes.

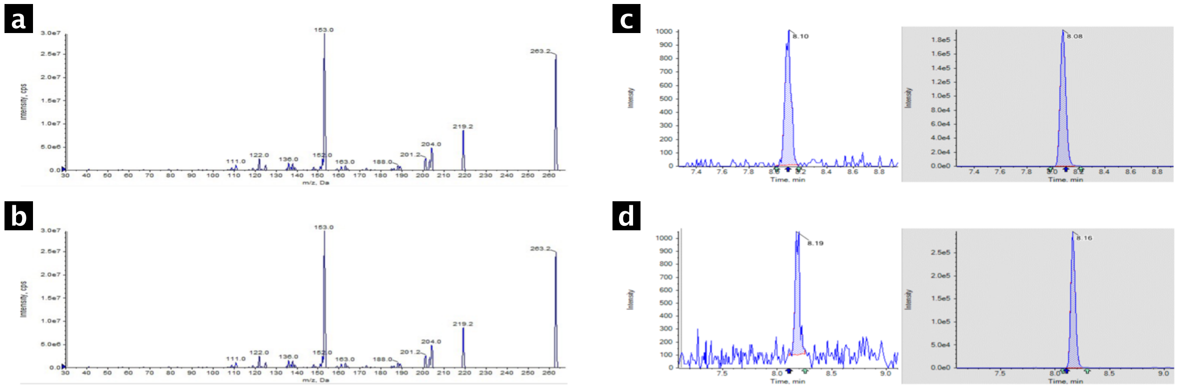

Supplementary Figure 17. Identification of ABA in SAG 698-1b using an internal standard. Negative ESI MS/MS spectrum of abscisic acid (**a**) or abscisic acid-D6 (**b**) fragment with m/z 263.2 (**c,d** left) and 269.2 (**c,d**) respectively were used for quantification ABA and the deuterated standards. LC-MS/MS (MRM) chromatograms of 0.1 ng/ml abscisic acid (**c**, left) calibration standard and the internal standard (**c**,. right). Detection of ABA using LC-MS/MS (MRM) chromatograms of the abscisic acid (**d**, left) internal standard (**d**, right) detected in the sample.

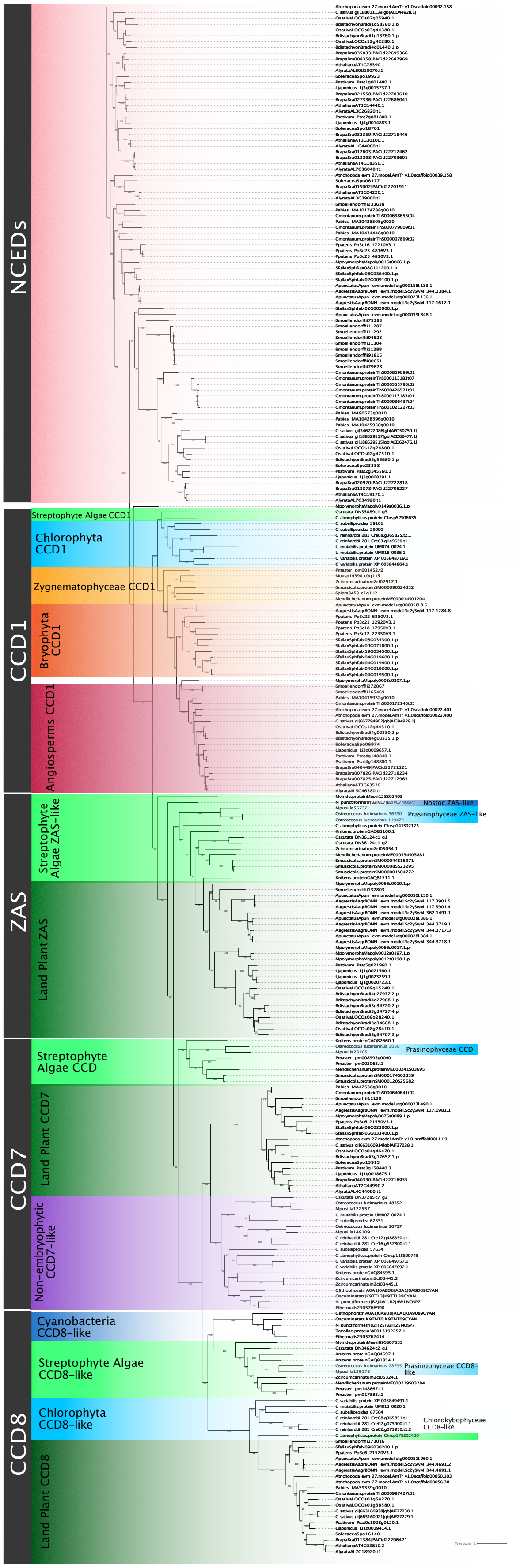

Supplementary Figure 18. Phylogenetic tree for CCD proteins. The tree was constructed by aligning 267 CCD proteins from 41 species including *Crocus* using the MAFFT program and subjected to phylogenetic analysis by the maximum likelihood method using the IQ-TREE software. The numbers on the nodes indicate the bootstrap values after 100 replicates.

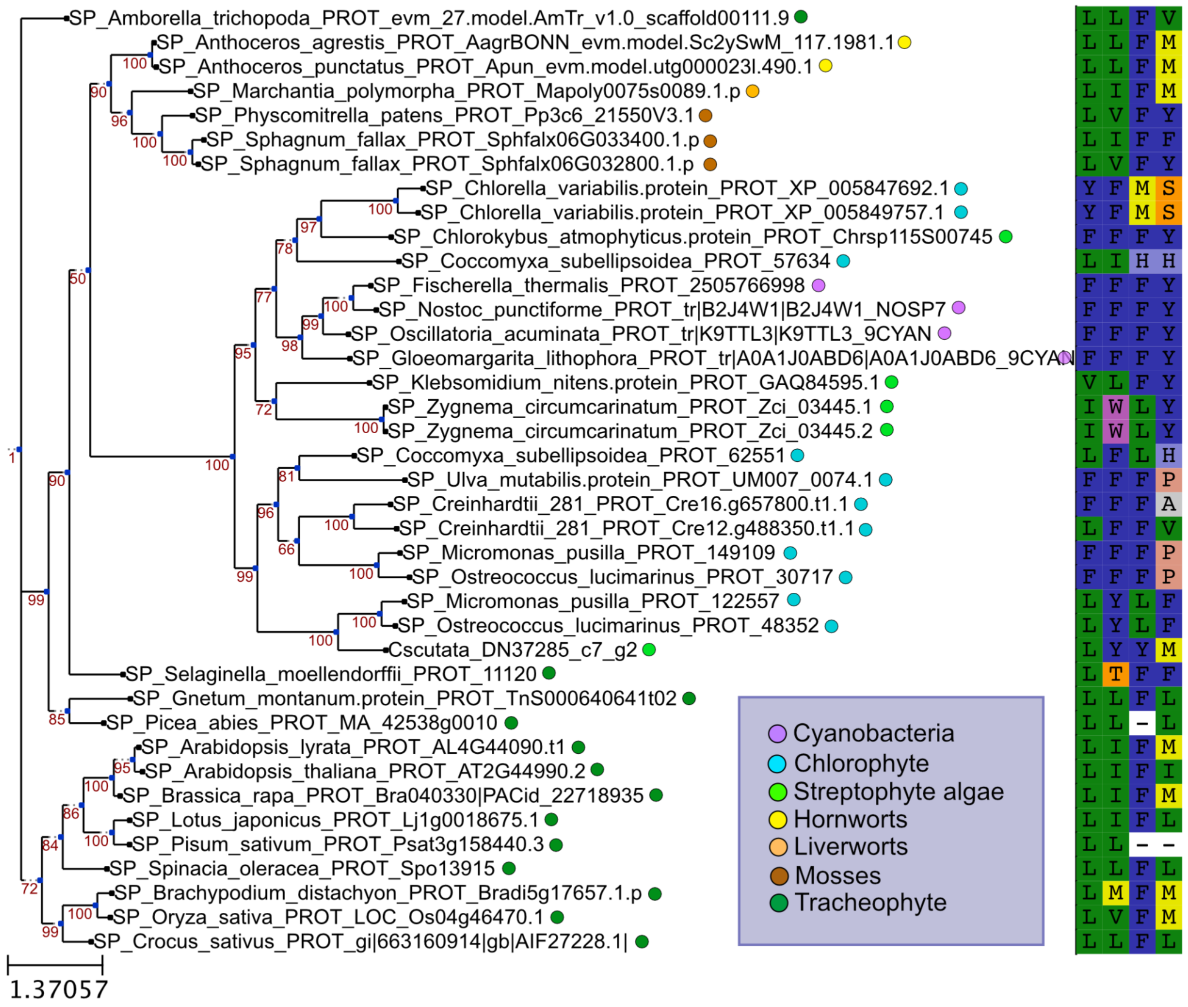

Supplementary Figure 19. Phylogeny of CCD7 homologs. On the left, amino acids correspond to the positions of Phe-171, Phe-411, Val-478 and Phe-589 of ZmVP14. These amino acids were proposed to be crucial for substrate specificity of all CCDs (Messing et al., 2010). Dashes are gaps.

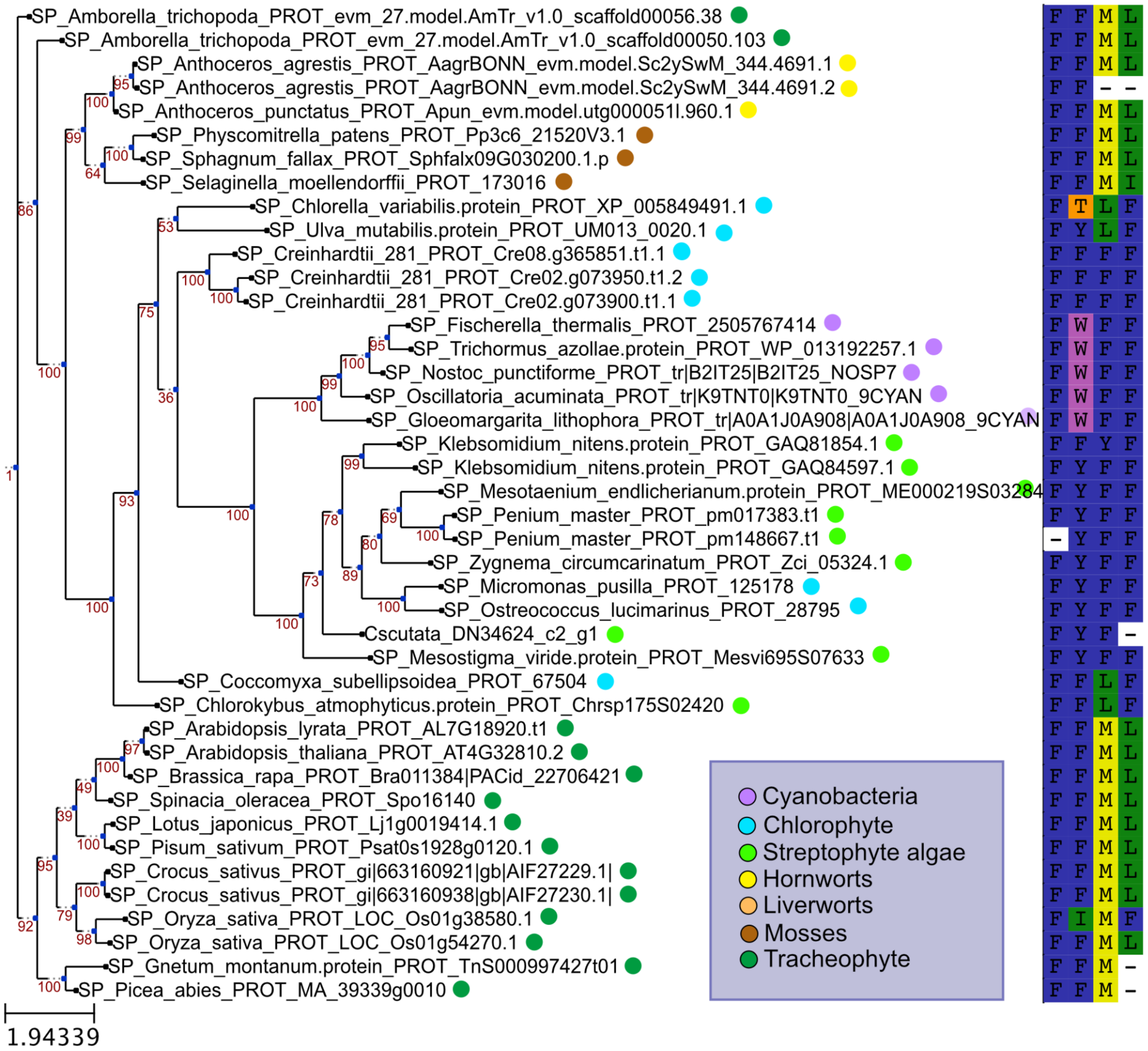

Supplementary Figure 20. Phylogeny of CCD8 homologs. On the left, amino acids correspond to the positions of Phe-171, Phe-411, Val-478 and Phe-589 of ZmVP14. These amino acids were proposed to be crucial for substrate specificity of all CCDs (Messing et al., 2010). Dashes are gaps.

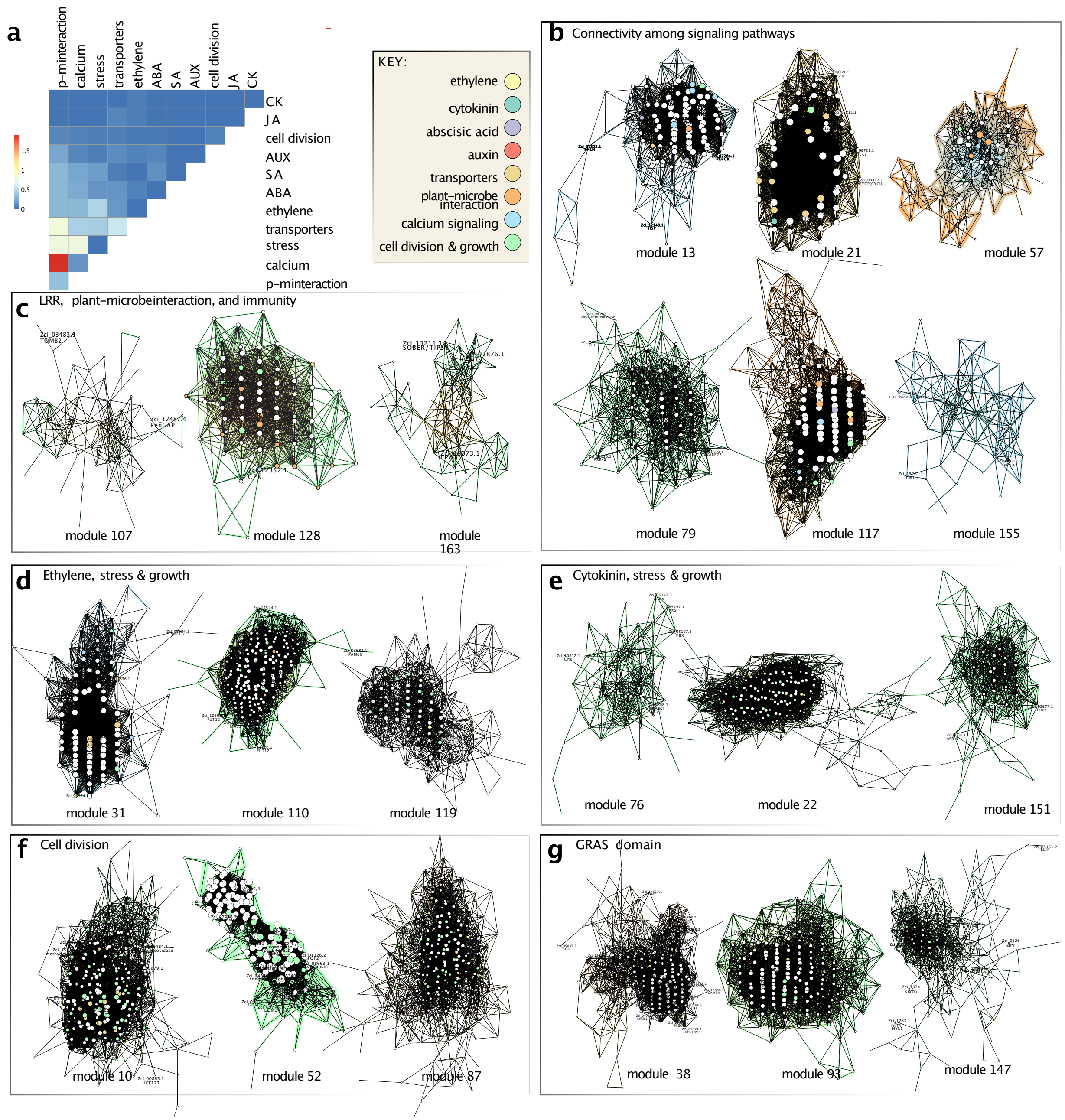

Supplementary Figure 21. Selected gene co-expression modules. (**a**) Heatmap of per-module co-occurrence frequencies among genes associated with plant-microbe (p-m) interaction, calcium signaling, phytohormone, stress, transporters, cell division, and diverse phytohormones (see abbreviations below); based on 150 out of 406 total gene co-expression modules showing co-occurrence of at least two functional categories. Selected gene modules are shown reflecting (**b**) Connectivity among signaling pathways, (**c**) leucine-rich repeat (LRR) proteins, plant-microbe interaction, and immunity, (**d**) ethylene, stress, and growth, (**e**) cytokinin, stress, and growth, (**f**) cell division, and (**g**) GRAS domain-containing proteins. A detailed description of gene modules is provided in Supplementary Text 4. Node (gene) sizes are proportional to number of neighbors and edge (co-expression) widths are proportional to Pearson's correlation coefficient whereas colors are those of interconnected genes; edge gradient colors highlight the two dominant gene categories as indicated in the KEY. The full gene co-expression results can be accessed in our online portal (https://zygnema.sbs.ntu.edu.sg/).
