# Supplementary material for: Genomes of multicellular algal sisters to land plants illuminate signaling network evolution
Source: Nat Genet. 2024 May 1;56(5):1018–31. doi: 10.1038/s41588-024-01737-3 (PMC11096116; doi:10.1038/s41588-024-01737-3)
Supplement: Supplementary file 2 — Reporting Summary [file 41588_2024_1737_MOESM2_ESM.pdf]

nature portfolio

Corresponding author(s): Jan de Vries, Yanbin Yin

Last updated by author(s): Feb 28, 2024

Reporting Summary

Nature Portfolio wishes to improve the reproducibility of the work that we publish. This form provides structure for consistency and transparency in reporting. For further information on Nature Portfolio policies, see our Editorial Policies and the Editorial Policy Checklist.

Statistics

For all statistical analyses, confirm that the following items are present in the figure legend, table legend, main text, or Methods section.

n/a	Confirmed	
☐	☒	The exact sample size (*n*) for each experimental group/condition, given as a discrete number and unit of measurement
☐	☒	A statement on whether measurements were taken from distinct samples or whether the same sample was measured repeatedly
☒	☐	The statistical test(s) used AND whether they are one- or two-sided<br>*Only common tests should be described solely by name; describe more complex techniques in the Methods section.*
☐	☒	A description of all covariates tested
☐	☒	A description of any assumptions or corrections, such as tests of normality and adjustment for multiple comparisons
☒	☐	A full description of the statistical parameters including central tendency (e.g. means) or other basic estimates (e.g. regression coefficient) AND variation (e.g. standard deviation) or associated estimates of uncertainty (e.g. confidence intervals)
☒	☐	For null hypothesis testing, the test statistic (e.g. *F*, *t*, *r*) with confidence intervals, effect sizes, degrees of freedom and *P* value noted<br>*Give P values as exact values whenever suitable.*
☒	☐	For Bayesian analysis, information on the choice of priors and Markov chain Monte Carlo settings
☒	☐	For hierarchical and complex designs, identification of the appropriate level for tests and full reporting of outcomes
☐	☒	Estimates of effect sizes (e.g. Cohen's *d*, Pearson's *r*), indicating how they were calculated

Our web collection on statistics for biologists contains articles on many of the points above.

Software and code

Policy information about availability of computer code

Data collection	Transmission electron micrographs were taken on a Zeiss Libra 120 transmission electron microscope (Carl Zeiss AG, Oberkochen, Germany) at 80 kV, which was equipped with a TRS 2k SSCCD camera and operated by ImageSP software (Albert Tröndle Restlichtverstärker Systeme, Moorenweis, Germany).<br><br>DNA extraction: Quality and quantity of purified DNA was evaluated by using 1% agarose gel electrophoresis, NanoDrop 2000/2000c Spectrophotometers, and Qubit 3.0 Fluorometer (Thermo Fisher Scientific).<br><br>Library preparation and sequencing: DNA samples were sequenced at Roy J. Carver Biotechnology Center at University of Illinois at Urbana-Champaign, using Oxford Nanopore and Illumina technologies (Table S1A). Oxford Nanopore DNA libraries were prepared with 1D library kit SQK-LSK109 and sequenced with SpotON R9.4.1 FLO-MIN106 flowcells for 48h on a GridIONx5 sequencer. Base calling was performed with Guppy v1.5 (https://community.nanoporetech.com). Illumina shotgun genomic libraries were prepared with the Hyper Library construction kit (Kapa Biosystems, Roche). Libraries had an average fragment size of 450 bp, from 250 to 500 bp, and sequenced with 2x250 bp paired-end on HiSeq 2500. Additional DNA samples were sequenced at the Genome Research Core in University of Illinois at Chicago and JGI. The Illumina shotgun genomic libraries were prepared with Nextera DNA Flex Library Prep Kit. The libraries had an average fragment size of 403 bp and sequenced with 2x150 bp paired-end on HiSeq 4000 (Table S1A). RNA samples were sequenced at the Genome Research Core in University of Illinois at Chicago. The libraries were prepared by rRNA depletion with Illumina Stranded Total RNA kit plus Ribo-Zero Plant (https://www.illumina.com/products/by-type/sequencing-kits/library-prep-kits/truseq-stranded-total-rna-plant.html), and 2x150 bp paired-end sequencing was performed on HiSeq 4000. RNA from the third batch of stress experiments were sequenced at the NGS-Integrative Genomics Core Unit of the University Medical Center Göttingen, Germany. Stranded mRNA libraries were prepared with the Illumina stranded mRNA kit and paired-end sequencing of 2x150 bp reads was carried out on an Illumina HiSeq 4000 platform.

LC-MS/MS analysis of abscisic acid
Abscisic acid was determined in samples using an LC-MS/MS system which consisted of Nexera X2 UPLC (Shimadzu) coupled QTRAP 6500+ mass spectrometer (Sciex). Chromatographic separations were carried out using the Acclaim RSLC C18 column (150×2.1 mm, 2.2µm, Thermo Scientific) employing acetonitrile/water+0.1% acetic acid linear gradient. The mass spectrometer was operated in negative ESI mode. Data was acquired in MRM mode using following transitions: 1) ABA 263.2->153.1 (-14 eV), 263.2->219.1 (-18 eV); 2) ABA -D6 (IS) 269.2->159.1 (-14 eV), 269.2->225.1 (-18 eV); declustering potential was -45 V. Freeze-dried moss samples were ground using the metal beads in homogenizer (Bioprep-24) to a fine powder. Accurately weighted (about 20 mg) samples were spiked with isotopically labeled ABA -D6 (total added amount was 2 ng) and extracted with 1.5 ml acetonitrile/water (1:1) solution acidified with 0.1% formic acid. Extraction was assisted by sonication (Elma S 40 H, 15 min, two cycles) and solution was left overnight for completion of extraction. Liquid was filtered through 0.2 µm regenerated cellulose membrane filters, evaporated to dryness upon a stream of dry nitrogen and redissolved in 100 µl extraction solution.

Data analysis

Transcriptome assembly
Raw RNA-seq reads (Table S1A) were quality checked with FastQC v.0.11.9 (http://www.bioinformatics.babraham.ac.uk/projects/fastqc/) (Andrews 2010), trimmed with TrimGalore (https://github.com/FelixKrueger/TrimGalore), and were inspected again with FastQC. All reads were combined, and de novo assembled with Trinity version 2.9.0 (Grabherr et al. 2011; Haas et al. 2013).

K-mer frequency analysis
The trimmed DNA Illumina reads were filtered out with BLASTP version 2.13.0+ using plastomes and mitogenomes from Zygnema as references. Remaining (putatively nuclear) were used to predict the best k-mer size by kmergenie v1.7048 (http://kmergenie.bx.psu.edu/) (Chikhi and Medvedev 2014). The histogram of the best k-mer was then uploaded to GenomeScope for viewing the genome plot (http://qb.cshl.edu/genomescope/) (Vurture et al. 2017) (Table S1B and Figure S2).

Genome assembly and scaffolding
To assemble the genome of SAG 698-1b, a total of 5.4 Gb (82x) of Oxford Nanopore nuclei DNA reads were assembled with wtdbg2 (Ruan and Li 2020) (https://github.com/ruanjue/wtdbg). Assembled contigs were polished by Racon v1.4 (Vaser et al. 2017) and three iterations of pilon version 1.2 (Walker et al. 2014) with Illumina paired-end reads. The polished genome was scaffolded by Dovetail Genomics HiRise software with Hi-C sequencing data (https://dovetailgenomics.com/). Genome contamination was examined by BLASTX against NCBI's NR database and contaminated scaffolds were removed.
To assemble the UTEX 1559 genome, an initial assembly was done with SPAdes v3 (Antipov et al. 2016) using Illumina paired-end reads (2x150 bp), three mate-pair libraries (insert size: 3-5 kb; 5-7 kb and 8-10 kb) and Oxford Nanopore reads (Table S1A). Assembled contigs were further scaffolded by two rounds of Platanus-allee (Kajitani et al. 2019) with Illumina paired-end reads (2x 250 bp), three mate-pair libraries (insert size 3-5 kb; 5-7 kb and 8-10 kb) and Oxford Nanopore reads. For the UTEX 1560 genome, Illumina paired-end (2x150 bp) and PacBio HiFi reads were used for assembly with SPAdes and further scaffolded with Platanus-allee. Scaffolds with contaminations were identified by BLASTX against NR and removed. The genomes of UTEX1559 and UTEX1560 were scaffolded by Dovetail Genomics HiRise software with Hi-C sequencing data from SAG 698-1b.
The genome of SAG 698-1a_XF was sequenced with PacBio HiFi long reads (40 Gb), Nanopore long reads (4 Gb), and Illumina short reads (>100 Gb). The k-mer analysis using Illumina reads revealed two peaks in the k-mer distribution, suggesting that SAG 698-1a_XF exists as a diploid organism with an estimated heterozygosity rate of 2.22% (Figure S2). All Illumina short reads and the Nanopore reads were first assembled into contigs using SPAdes. Then, WENGAN (Di Genova et al. 2021) was used to assemble HiFi long reads and Illumina paired-end reads (2x150 bp) using the SPAdes contigs as the reference. Lastly, the resulting WENGAN v0.2 contigs were scaffolded and gaps were closed with Platanus-allee using all the Nanopore, HiFi, and Illumina reads to derive a consensus pseudo-haploid genome.
To evaluate the quality of assembled genomes (Table S1D), raw RNA-seq reads, Oxford Nanopore and Illumina DNA reads were mapped to the assembly with hisat v2 (Kim et al. 2019), minimap v2 (Li 2018), and bowtie v2 (Langmead and Salzberg 2012), respectively. To assess genome completeness, a BUSCO v.5.2.2 (Seppey et al. 2019) analysis was performed with the 'Eukaryota odb10' and 'Viridiplantae odb10' reference sets.

Repeat annotation
Repetitive DNA was annotated using the homology strategy with repeat libraries generated with RepeatModeler (v.2.0.1). RepeatModeler integrates RepeatScout, RECON, LTRharvest and LTR_retriever tools (version 2.0.1; http://www.repeatmasker.org/RepeatModeler/) (Flynn et al. 2020). The MITE (Miniature inverted-repeat transposable elements) library was identified with MITE-tracker (2018 release) (Crescente et al. 2018) software. These two identified libraries were combined and incorporated into RepeatMasker (version 4.0.9; http://www.repeatmasker.org/) for repeat annotation.

Genome annotation
In all four genomes, protein coding genes were predicted by the MAKER-P pipeline (v3.01.03) (Campbell et al. 2014) which integrates multiple gene prediction resources, including ab initio prediction, protein homology-based gene prediction and transcripts-based evidence. First, repetitive elements were masked by RepeatMasker with a custom repeat library generated by RepeatModeler. Rfam with infernal and tRNA-Scan2 were used to analyze non-coding RNA and tRNA. For the transcripts evidence, total of 103,967 transcripts were assembled by Trinity v2.9.0 (reference-free) and StringTie v2.1 (reference-based) with RNA-seq reads. Transcriptome assembly was used for generating a complete protein-coding gene models using PASA. Proteins from M. endlicherianum, S. muscicola and A. thaliana (TAIR10) were used as homology-based evidence. Then, the resulting protein-coding gene models from the first iteration of MAKER-P pipeline were used as training date set for SNAP and Augustus models, which were fed into MAKER for the second iteration of annotation. After three rounds of gene prediction, MAKER-P combined all the protein-coding genes as the final annotated gene sets.

Plastome and mitogenome assembly and annotation
NOVOPlasty 3.8.2 (https://github.com/ndierckx/NOVOPlasty) (Dierckxsens et al. 2017) was used to assemble plastomes. The contiguity of assembled plastomes was examined in Geneious software (https://www.geneious.com/) (Kearse et al. 2012) with read mapping. For SAG 698-1b mitogenome assembly, Oxford Nanopore reads were assembled with Canu (Koren et al. 2017), where one long mitogenome contig of 238,378 bp was assembled. This contig was circularized and polished with three rounds of pilon (Walker et al. 2014), that was further corrected with Illumina raw reads and compared with mitogenome of UTEX 1559 (MT040698; (Orton et al. 2020)) in Geneious. For SAG 698-1a_XF, PacBio HiFi reads were used for the assembly of its mitogenome.
Plastome and mitogenome annotation was performed with GeSeq (Tillich et al. 2017; v2021) (https://chlorobox.mpimp-golm.mpg.de/geseq.html). For plastome annotation, BLAT search and HMMER profile search (Embryophyta chloroplast) were used for coding sequence, rRNA and tRNA prediction; ARAGORN v1.2.38, ARWEN v1.2.3 and tRNAscan-SE v2.0.5 were used for tRNA annotation. For mitogenome annotation, Viridiplantae was use for BLAT Reference Sequences. The annotated gff files were uploaded for drawing circular organelle

genome maps on OGDRAW (https://chlorobox.mpimp-golm.mpg.de/OGDraw.html) (Greiner et al. 2019).

Comparison of Z. circumcarinatum genomes (SAG 698-1b, UTEX 1559, UTEX 1560)
Two approaches were used to compare the three genomes (Figure S8). The first approach was based on the whole genome alignment (WGA) by using MUMMER v4.0.0. The parameters "--maxmatch -c 90 -l 40" were set to align the three genomes and then "-i 90 -l 1000" were set to filter out the smaller fragments. The second approach focused on the gene content comparisons. Orthofinder was used to obtain ortholog groups (orthogroups) from genomes' annotated proteins. Orthofinder results led to a Venn diagram with unique genes (orthogroups with genes from only 1 genome), cloud genes (orthogroups with genes from only 2 genomes), and core genes (orthogroups with genes from all 3 genomes), which collectively form the pan-genome. Orthofinder could have failed to detect homology between very rapidly evolved orthologous genes, which leads to an under-estimation of core genes. Also, gene prediction may have missed genes in one genome but found them in other genomes. To address these issues, the raw DNA reads of each genome were mapped to the unique genes and cloud genes using BWA. This step was able to push more unique genes and cloud genes to core genes or push some unique genes to cloud genes. The following criteria were used to determine if an orthogroup needed to be re-assigned: (i) the number of reads and coverage calculated by bedtools are >10 and >0.8 for a gene, respectively, and (ii) >60% coverage of genes in the orthogroup find sequencing reads from the other genomes. After this step the final Venn diagram was made (Figure S8F), showing the counts of the final core genes, cloud genes, and unique genes.

Whole genome duplication (WGD) analysis
To identify possible WGDs, Ks and 4dtv values were calculated for each genome. First, all paralog pairs were identified using RBBH (Reciprocal Best BLAST Hit) method using protein sequences (E-value < 1e-6), following the method described by Bowman et al. (Bowman et al. 2017). RBBH paralog pairs were aligned with MAFFT v7.3.10 (Katoh and Standley 2013) and the corresponding nucleotide alignments were generated. Using RBBH alignments of paralog pairs, KaKs_Calculator2.0 (Wang et al. 2010) with the YN model and the calculate_4DTV_correction.pl script were run to calculate Ks and 4dtv values for each alignment, respectively. Ks = 0 and 4dtv = 0 values were filtered. The Ks and 4dtv distributions were fitted with a gaussian kernel density model using the seaborn package. For the SAG 698-1b chromosome-level genome, MCscan (Wang et al. 2012) was run to identify syntenic block regions with default parameters.

Species phylogeny and divergence time analysis
Sixteen representative genomes were selected, including two chlorophytes (Volvox carteri, Chlamydomonas reinhardtii), seven Zygnematophyceae (Zygnema circumcarinatum SAG 698-1b, UTEX 1559, UTEX 1560, Z. cf. cylindricum SAG 698-1a_XF, Mesotaenium endlicherianum, Penium margaritaceum, Spirogloea muscicola), four additional streptophyte algae (Chara braunii, Klebsormidium nitens, Chlorokybus melkonianii, Mesostigma viride), two bryophytes (Marchantia polymorpha, Physcomitrium patens) and a vascular plant (Arabidopsis thaliana). Orthogroups were generated by OrthoFinder version 2.5.2 (Emms and Kelly 2019) and 493 low-copy orthogroups containing ≤ 3 gene copies per genome were aligned with MAFFT v7.310 (Katoh and Standley 2013). Gene alignments were concatenated and gaps were removed by Gblocks version 0.91b (Castresana 2000). Phylogenetic tree was built using RAxML v.8 (Stamatakis 2014) with the "-f a" method and the PROTGAMMAJTT model, and support with 100 pseudoreplicates of non-parametric bootstrap. The tree was rooted on Chlorophyta.
Using the above methodology, additional phylogenetic analyses were performed with (i) the four Zygnema strains and (ii) the seven Zygnematophyceae genomes, in order to obtain a higher number of single-copy loci, 5,042 and 204, respectively (Figure S7).
Divergence time estimation was carried out with MCMCTree implemented in the PAML package version 4.10.0j (Yang 2007). The 493 low-copy orthogroup protein sequence alignment was converted to the corresponding nucleotide alignment for MCMCTree, in which 10 MCMC (Markov Chain Monte Carlo) chains were run, each for 1,000,000 generations (Table S1F). Three calibration were set in the reference tree according to Morris et al., (Morris et al. 2018) on the nodes Viridiplantae (972.4~669.9 Ma), Streptophyta (890.9~629.1 Ma) and Embrophyta (514.8~473.5 Ma).

Comparative genomics analysis
Sixteen representative genomes were selected, including two chlorophytes (Volvox carteri, Chlamydomonas reinhardtii), seven Zygnematophyceae (Zygnema circumcarinatum SAG 698-1b, UTEX 1559, UTEX 1560, Z. cf. cylindricum SAG 698-1a_XF, Mesotaenium endlicherianum, Penium margaritaceum, Spirogloea muscicola), four additional streptophyte algae (Chara braunii, Klebsormidium nitens, Chlorokybus melkonianii, Mesostigma viride), two bryophytes (Marchantia polymorpha, Physcomitrium patens) and a vascular plant (Arabidopsis thaliana).
Orthogroups were inferred with Orthofinder. Time-calibrated species phylogeny was built with low-copy orthogroups (≤ 3 gene copies). Divergence time estimation was carried out with MCMCTree (version from 2017). Expanded and contracted gene families were identified with CAFE and the species phylogeny. For microexon analyses, MEPmodeler(Yu et al. 2022) was used (https://github.com/yuhuihui2011/MEPmodeler).
For comparative genomics studies of multicellularity, the sixteen genomes were classified into two groups, the unicellular group (C. reinhardtii, C. melkonianii, M. viride, S. muscicola, M. endlicherianum, P. margaritaceum) and the multicellular group (V. carteri, K. nitens, C. braunii, SAG 698-1a_XF, SAG 698-1b, UTEX 1559, UTEX 1560, and M. polymorpha, P. patens, A. thaliana). Proteins in the 16 genomes were annotated by Pfam to find functional domains. Domain occurrences (presense/absence) and abundances in each genome were recorded, and were compared between the two groups of genomes to infer domain gain, loss, and combination.

Gene family phylogenetic analysis
CAZyme families were identified with dbCAN v2 (Zhang et al. 2018) with default parameters (E-value < 1e-10 and coverage > 0.35). Whenever needed, dbCAN2 was rerun by using more relaxed parameters. The experimentally characterized cell wall enzymes were manually curated from the literature (Data S1 and Table S1L). Reference genes were included into the phylogenies to infer the presence of orthologs across the 16 genomes and guide the split of large families into subfamilies. Phylogenetic trees were built by using FastTree initially, and for some selected families, RAxML (Stamatakis 2014) and IQ-Tree v1.5.5 (Nguyen et al. 2015) were used to rebuild phylogenies to verify topologies.

Orthogroup expansion and contraction analysis
We inferred expanded and contracted gene families with CAFE v.5 using orthogroups inferred with Orthofinder v.2.4.0 and the previously inferred time-calibrated species phylogeny. CAFE v.5 was run with default settings (base) using the inferred orthogroups and a calibrated species phylogeny. Two independent runs arrived to the same final likelihood and lambda values. The first eight orthogroups (OG0-7) were excluded from the analysis due to too drastic size changes between branches that hampered likelihood calculation; excluded orthogroups were mostly exclusive to a single Zygnema or Chara genome and likely represented transposable elements, as judged by results of BLASTP against NR.

Phytohormones

Proteins involved in phytohormone biosynthesis and signaling were identified by BLASTP against annotated proteomes (e-value<1e-6) using Arabidopsis genes as queries. For genes with ubiquitous domains (e.g. CIPK, CPK3, SNRK2, CDG1, BAK1), hits were filtered by requiring BLASTP coverage ≥50% of the query. Significant hits were then aligned (MAFFT auto) and maximum likelihood gene trees were inferred in IQ-Tree using best-fit models and 1000 replicates of SH-like aLRT branch support ('-m TEST -msub nuclear -alrt 1000'). The final sets of homologs were identified by visually inspecting gene trees and identifying the most taxonomically diverse clade (with high support of SH-aLRT>0.85) that included the characterized Arabidopsis proteins. Bubble plot was generated with ggplot2 in R.

Constructing the co-expression network and establishing the Zygnema database.
The Highest Reciprocal Rank (HRR) co-expression network for Z. circumcarinatum SAG 698-1b was built from all samples (19 growth conditions) and the Zygnema database was established using the CoNekT framework (Proost and Mutwil 2018). The gene co-expression clusters were identified using the Heuristic Cluster Chiseling Algorithm (HCCA) with standard settings (Mutwil et al. 2010).

Screening for symbiotic genes and phylogenetic analysis
Symbiotic genes were screened against a database of 211 plant species across Viridiplantae lineage using proteins of the model plant Medicago truncatula as queries in BLASTP v2.11.0+ (Camacho et al. 2009) searches with default parameters and an e-value < 1e-10. Initial alignments of all identified homologs was performed using the DECIPHER package (Wright 2015) in R v4.1.2 (R Core Team). Positions with >60% gaps were removed with trimAl v1.4 (Capella-Gutiérrez et al. 2009) and a phylogenetic analysis was performed with FastTree v2.1.11 (Price et al. 2009). Clades corresponding to M. truncatula orthologs queries were extracted and a second phylogeny was performed. Proteins were aligned using MUSCLE v3.8.1551 (Edgar 2004) with default parameters and alignments cleaned as described above. Tree reconstruction was performed using IQ-Tree v2.1.2 (Minh et al. 2020) based on BIC-selected model determined by ModelFinder (Kalyaanamoorthy et al. 2017). Branch supports was estimated with 10,000 replicates each of both SH-aLRT (Guindon et al. 2010) and UltraFast Bootstraps (Hoang et al. 2018). Trees were visualized and annotated with iTOL v6 (Letunic and Bork 2021). For the GRAS family, a subset of 42 species representing the main lineages of Viridiplantae was selected and GRAS putative proteins screened using the HMMSEARCH program with default parameters and the PFAM domain PF03514 from HMMER3.3 (Johnson et al. 2010) package. Phylogenetic analysis was then conducted as described above.

Screening for CCD homologs and phylogenetic analysis
Annotated proteins from 21 land plant genomes (Amborella trichopoda, Anthoceros agrestis, Anthoceros punctatus, Arabidopsis lyrata, Arabidopsis thaliana, Azolla filiculoides, Brachypodium distachyon, Brassica rapa, Lotus japonicus, Marchantia polymorpha, Medicago truncatula, Oryza sativa, Physcomitrium patens, Picea abies, Pisum sativum, Salvinia cucullata, Selaginella moellendorffii, Sphagnum fallax, Spinacia oleracea, Gnetum montanum, Crocus sativus), 7 streptophyte algal genomes (Spirogloea muscicola, Penium_margaritaceum, Mesotaenium endlicherianum, Mesostigma viride, Klebsormidium nitens, Chlorokybus melkonianii, Chara braunii, Zygnema circumcarinatum), 6 chlorophyte genomes (Ulva mutabilis, Ostreococcus lucimarinus, Micromonas pusilla, Micromonas sp., Chlamydomonas reinhardtii, Coccomyxa subellipsoidea, Chlorella_variabilis), 5 cyanobacterial genomes (Trichormus azollae, Oscillatoria acuminata, Nostoc punctiforme, Gloeomargarita lithophora, Fischerella thermalis), as well as the transcriptome of Coleochaete scutata (de Vries et al. 2018). The representative A. thaliana protein was used as query for BLASTP searches against the above annotated proteins (E-value < 1e-5). Homologs were aligned with MAFFTv7.453 L-INS-I approach (Katoh and Standley 2013) and maximum likelihood phylogenies computed with IQ-Tree v.1.5.5 (Nguyen et al. 2015), with 100 bootstrap replicates and BIC-selected model (WAG+R9) with ModelFinder (Kalyaanamoorthy et al. 2017). Functional residue analyses were done based on published structural analysis (Messing et al. 2010), and the alignments were plotted with ETE3 (Huerta-Cepas et al. 2016).

Phylogeny of MADS-box genes
MADS-domain proteins were identified by Hidden Markov Model (HMM) searches (Eddy 1998) on annotated protein collections. MADS-domain sequences of land plants and opisthokonts were taken from previous publications (Marchant et al. 2022; Gramzow et al. 2010). MADS domain proteins of other streptophyte algae were obtained from the corresponding genome annotations and transcriptomic data (One Thousand Plant Transcriptomes Initiative, 2019) (One thousand plant transcriptomes and the phylogenomics of green plants 2019). Additional MADS-domain proteins of Zygnematophyceae were identified by BLAST against transcriptome data available at NCBI's sequence read archive (SRA) (Sayers et al. 2021). MADS-domain-protein sequences were aligned using MAFFTv7.310 (Katoh and Standley 2013) with default options. Sequences with bad fit to the MADS domain were excluded and the remaining sequences realigned, and trimmed using trimAl (Capella-Gutiérrez et al. 2009) with options "-gt .9 -st .0001". A maximum likelihood phylogeny was reconstructed using RAxMLv8.2.12 (Stamatakis 2014) on the CIPRES Science Gateway (Miller et al. 2011).

For manuscripts utilizing custom algorithms or software that are central to the research but not yet described in published literature, software must be made available to editors and reviewers. We strongly encourage code deposition in a community repository (e.g. GitHub). See the Nature Portfolio guidelines for submitting code & software for further information.

Data

Policy information about availability of data

All manuscripts must include a data availability statement. This statement should provide the following information, where applicable:

- Accession codes, unique identifiers, or web links for publicly available datasets
- A description of any restrictions on data availability
- For clinical datasets or third party data, please ensure that the statement adheres to our policy

Data and code availability
The four Zygnema genomes, raw DNA reads, and rRNA-depleted RNA-seq of SAG 698-1b can be accessed through NCBI BioProject PRJNA917633. The raw DNA read data of UTEX1559 and UTEX1560 sequenced by the Joint Genome Institute can be accessed through BioProjects PRJNA566554 and PRJNA519006, respectively. RNA-seq data of UTEX1559 can be accessed through BioProject PRJNA524229. Poly-A enriched RNA-seq data of SAG 698-1b can be accessed through BioProject PRJNA890248 and the Sequence Read Archive (SRA) under the accession SRR21891679 to SRR21891705. Zygnema genomes are also available through the PhycoCosm portal129 (https://phycocosm.jgi.doe.gov/SAG698-1a; https://phycocosm.jgi.doe.gov/SAG698-1b; https://phycocosm.jgi.doe.gov/UTEX1559; https://phycocosm.jgi.doe.gov/UTEX1560). Data files are available at Figshare https://doi.org/10.6084/m9.figshare.22568197 and Mendeley under doi: 10.17632/gk965cbjp9.1
No original code was used; all computational analyses were performed with published tools and are cited in the Methods section.

Research involving human participants, their data, or biological material

Policy information about studies with human participants or human data. See also policy information about sex, gender (identity/presentation), and sexual orientation and race, ethnicity and racism.

Reporting on sex and gender	n/a
Reporting on race, ethnicity, or other socially relevant groupings	n/a
Population characteristics	n/a
Recruitment	n/a
Ethics oversight	n/a

Note that full information on the approval of the study protocol must also be provided in the manuscript.

Field-specific reporting

Please select the one below that is the best fit for your research. If you are not sure, read the appropriate sections before making your selection.

☒ Life sciences ☐ Behavioural & social sciences ☐ Ecological, evolutionary & environmental sciences

For a reference copy of the document with all sections, see nature.com/documents/nr-reporting-summary-flat.pdf

Life sciences study design

All studies must disclose on these points even when the disclosure is negative.

Sample size	Analyses were done on millions of pooled filaments of Zygnema (cultures were grown to a density on plate during which healthy growth still occurred but the whole plate was covered to yield appropriate biomass for extraction of nucleic acids). Sequencing was performed to a depth that was choes based on approaching saturation level (based on obtaining global gene expression patterns given the number of genes in the genomes of Zygnema). For chromosome counting, a minimum of 10 cells each from three independent cell cultures were analyzed. TEM was performed using two independent cell cultures and each time ≥15 algal filaments.
Data exclusions	No data were excluded
Replication	RNAseq under different conditions was done on at least three independent biological replicates per condition, all of which were used for this study. All attempts at replication were successful. For chromosome counting, three independent cell cultures were analyzed.
Randomization	Apart from taking a random selection of millions of filaments from a liquid culture, samples were not additionally randomized selected for any experiment. Treatments were designed so that no batch effect due to setups occurred (defined media, exact concentrations of challenges, monitoring of growth light and temperature etc.)
Blinding	Blinding was not relevant for this study. It is irrelevant for the genome and transcriptome analyses because we always worked with all data, using all versus all comparisons, unsupervised methods, and fully transparent pipelines. All phenotypic evaluation (e.g. counts of chromosomes) are quantifyable and unambigous.

Reporting for specific materials, systems and methods

We require information from authors about some types of materials, experimental systems and methods used in many studies. Here, indicate whether each material, system or method listed is relevant to your study. If you are not sure if a list item applies to your research, read the appropriate section before selecting a response.

Materials & experimental systems

n/a	Involved in the study	
☒	☐	Antibodies
☐	☒	Eukaryotic cell lines
☒	☐	Palaeontology and archaeology
☒	☐	Animals and other organisms
☒	☐	Clinical data
☒	☐	Dual use research of concern
☐	☒	Plants

Methods

n/a	Involved in the study	
☒	☐	ChIP-seq
☒	☐	Flow cytometry
☒	☐	MRI-based neuroimaging

Eukaryotic cell lines

Policy information about cell lines and Sex and Gender in Research

Cell line source(s)	Z. circumcarinatum SAG 698-1b and Z. cf. cylindricum SAG 698-1a were obtained from the Culture Collection of Algae at Göttingen University (SAG) (https://sagdb.uni-goettingen.de). Z. circumcarinatum UTEX 1559 and UTEX 1560 were obtained from the UTEX Culture Collection of Algae at UT-Austin (https://utex.org/). From Z. cf. cylindricum SAG 698-1a, a single filament was picked and a new culture was established, coined Z. cf. cylindricum SAG 698-1a_XF; Z. cf. cylindricum SAG 698-1a_XF was used for genome sequencing.
Authentication	Authentication was carried out by the Experimental Phycology and Culture Collection of Algae in Göttingen, Germany, via microscopy and genetic markers.
Mycoplasma contamination	n/a
Commonly misidentified lines (See ICLAC register)	n/a

Dual use research of concern

Policy information about dual use research of concern

Hazards

Could the accidental, deliberate or reckless misuse of agents or technologies generated in the work, or the application of information presented in the manuscript, pose a threat to:

No	Yes	
☒	☐	Public health
☒	☐	National security
☒	☐	Crops and/or livestock
☒	☐	Ecosystems
☒	☐	Any other significant area

Experiments of concern

Does the work involve any of these experiments of concern:

No	Yes	
☒	☐	Demonstrate how to render a vaccine ineffective
☒	☐	Confer resistance to therapeutically useful antibiotics or antiviral agents
☒	☐	Enhance the virulence of a pathogen or render a nonpathogen virulent
☒	☐	Increase transmissibility of a pathogen
☒	☐	Alter the host range of a pathogen
☒	☐	Enable evasion of diagnostic/detection modalities
☒	☐	Enable the weaponization of a biological agent or toxin
☒	☐	Any other potentially harmful combination of experiments and agents
