# Supplementary material for: Genomes of multicellular algal sisters to land plants illuminate signaling network evolution
Source: Nat Genet. 2024 May 1;56(5):1018–31. doi: 10.1038/s41588-024-01737-3 (PMC11096116; doi:10.1038/s41588-024-01737-3)
Supplement: Supplementary file 5 — Supplementary Data [file 41588_2024_1737_MOESM5_ESM.pdf]

Data S1-1

GT2

https://itol.embl.de/tree/979822487247861669516257

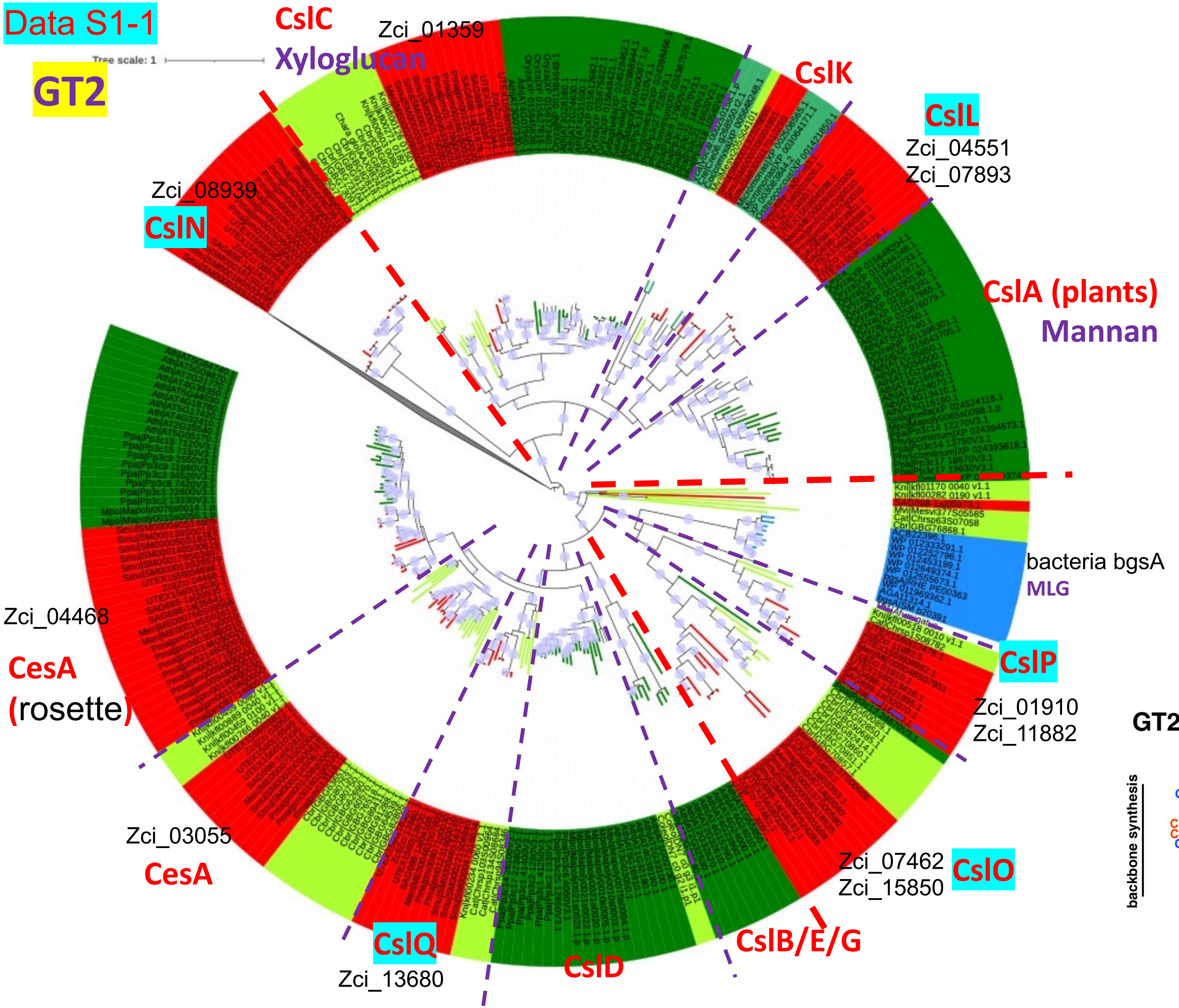

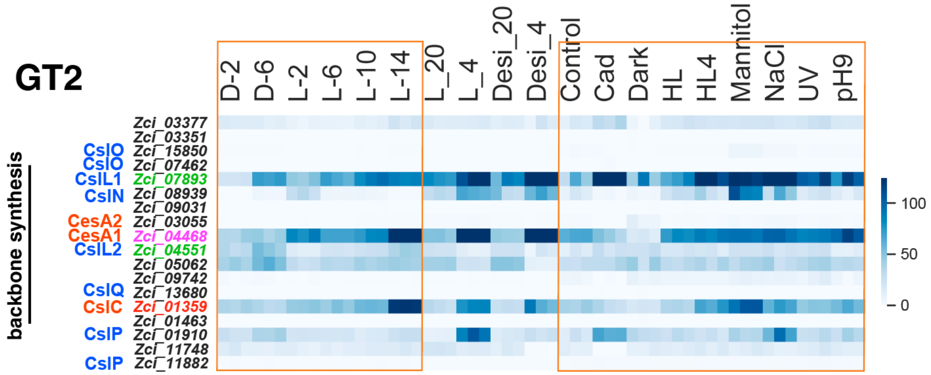

Data S1-2

GH9: cellulase

(Urbanowicz et al., 2007)

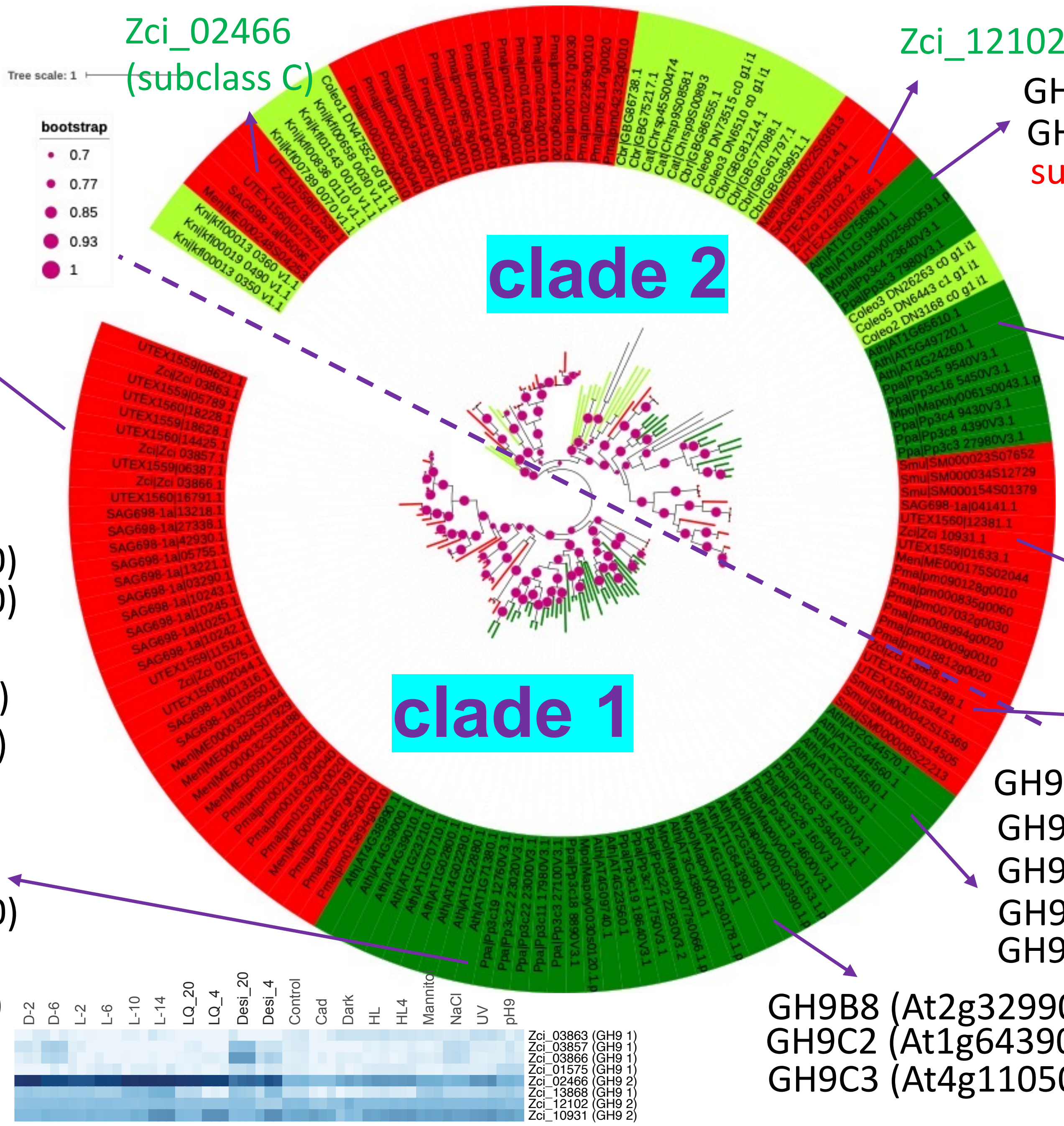

Zci_02466 (subclass C)

Zci_12102 (subclass A)

GH9B7 (At1g75680)
GH9B5 (At1g19940)
subclass B

Zci_03863
Zci_03857
Zci_03866
Zci_01575
(subclass C)

KOR3/GH9A2 (At1g65610)
KOR1/GH9A1 (AT5G49720)
KOR2/GH9A3 (AT4G24260)
subclass A

Zci_10931 (subclass A)

Zci_13868 (subclass C)

GH9B14 (At4g09740)
GH9B15 (At4g23560)
GH916 (At4g38990)
GH9B17 (At4g39000)
GH9B18 (At4g39010)
GH9B6 (At1g23210)
GH9B1 (At1g70710)
GH9B2 (At1g02800)
GH9B13 (At1g02290)
GH9B4 (At1g22880)
GH9B3 (At1g71380)
GH9 (At3g43860)
subclass B

GH9B12 (At2g44570)
GH9B11 (At2g44560)
GH9B9 (At2g44540)
GH9C1 (At1g48930)
GH9B10 (At2g44550)

GH9B8 (At2g32990)
GH9C2 (At1g64390)
GH9C3 (At4g11050)
subclass B&C

https://itol.embl.de/tree/208127240222127741667356629

Data S1-3a

Mannan

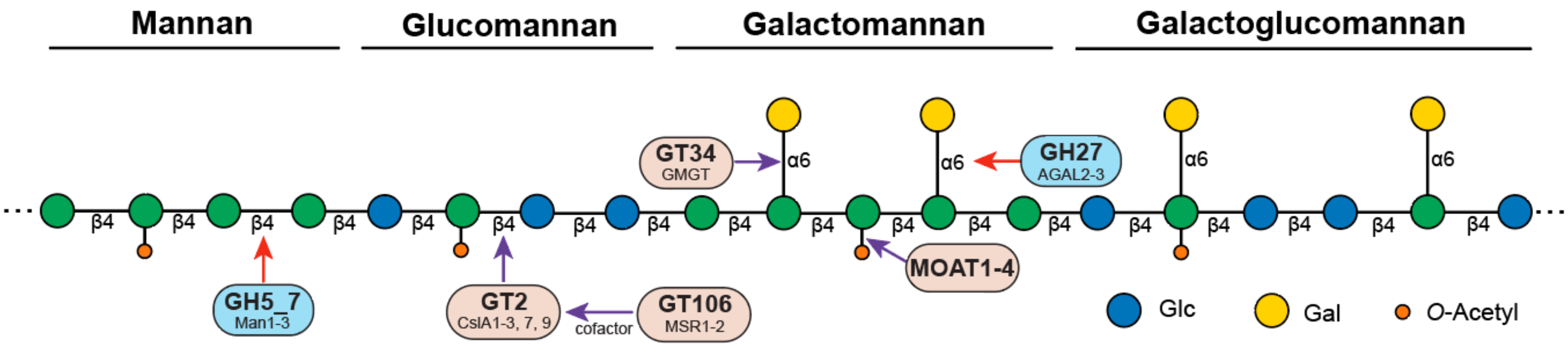

Data S1-3b

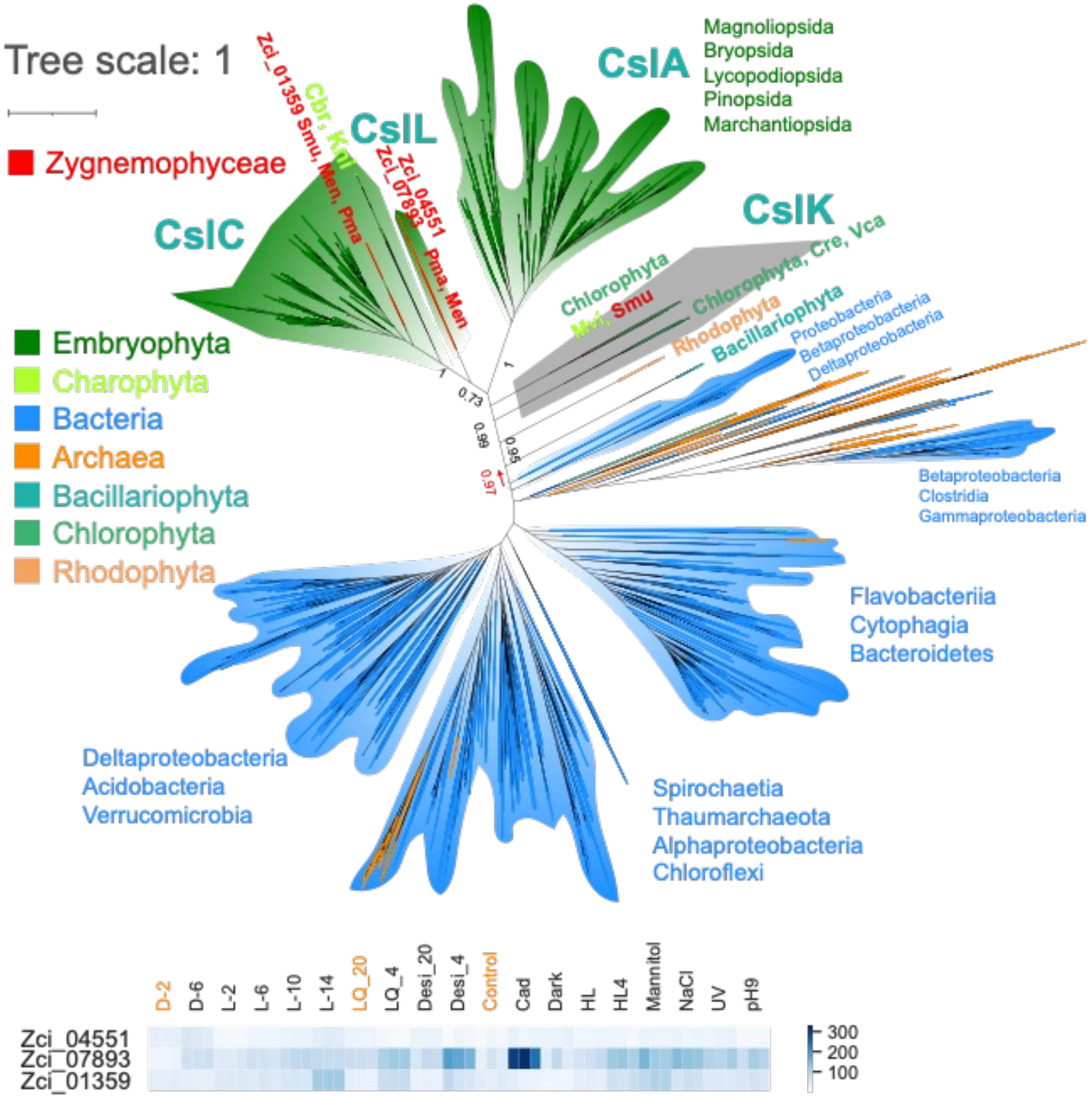

Tree scale: 1
Zygnemophyceae
Embryophyta
Charophyta
Bacteria
Archaea
Bacillariophyta
Chlorophyta
Rhodophyta
CslC
CslL
CslA
CslK
Zci_01359 Smu, Men, Pma
Cbr, Kni
Zci_04551
Zci_07893
Pma, Men
Magnoliopsida
Bryopsida
Lycopodiopsida
Pinopsida
Marchantiopsida
Chlorophyta
Mvi, Smu
Chlorophyta, Cre, Vca
Rhodophyta
Bacillariophyta
Proteobacteria
Betaproteobacteria
Deltaproteobacteria
Betaproteobacteria
Clostridia
Gammaproteobacteria
Flavobacteriia
Cytophagia
Bacteroidetes
Deltaproteobacteria
Acidobacteria
Verrucomicrobia
Spirochaetia
Thaumarchaeota
Alphaproteobacteria
Chloroflexi
D-2
D-6
L-2
L-6
L-10
L-14
LQ_20
LQ_4
Desi_20
Desi_4
Control
Cad
Dark
HL
HL4
Mannitol
NaCl
UV
pH9
Zci_04551
Zci_07893
Zci_01359
300
200
100

Data S1-4

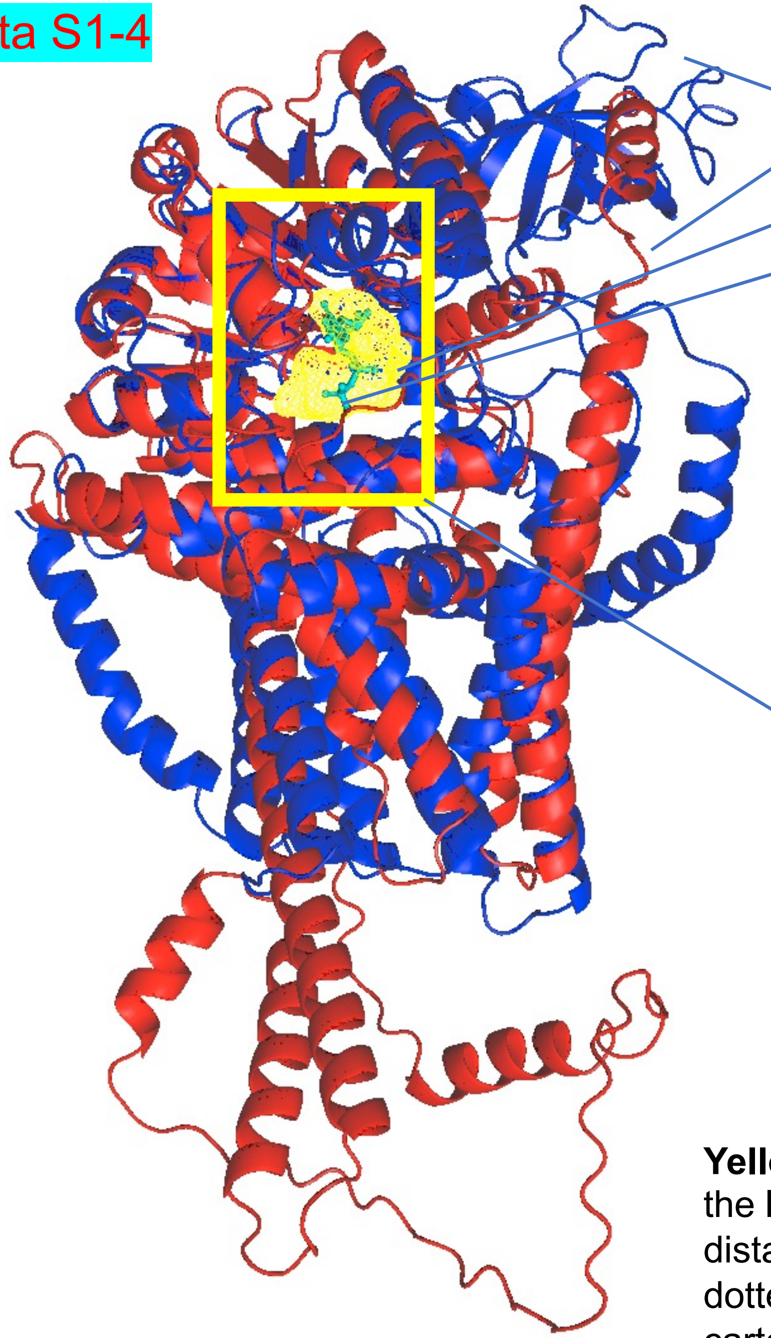

Superimposed 3D structures with ligands

blue: 4HG6 (bcsA, solved PDB structure, PubMed: 23222542)

red: Zci_07893 (predicted ZcCslL1 3D structure from AlphaFold)

yellow mesh: GDP-mannose docked into ZcCslL1 structure

cyan sticks: UDP-glucose in bcsA structure

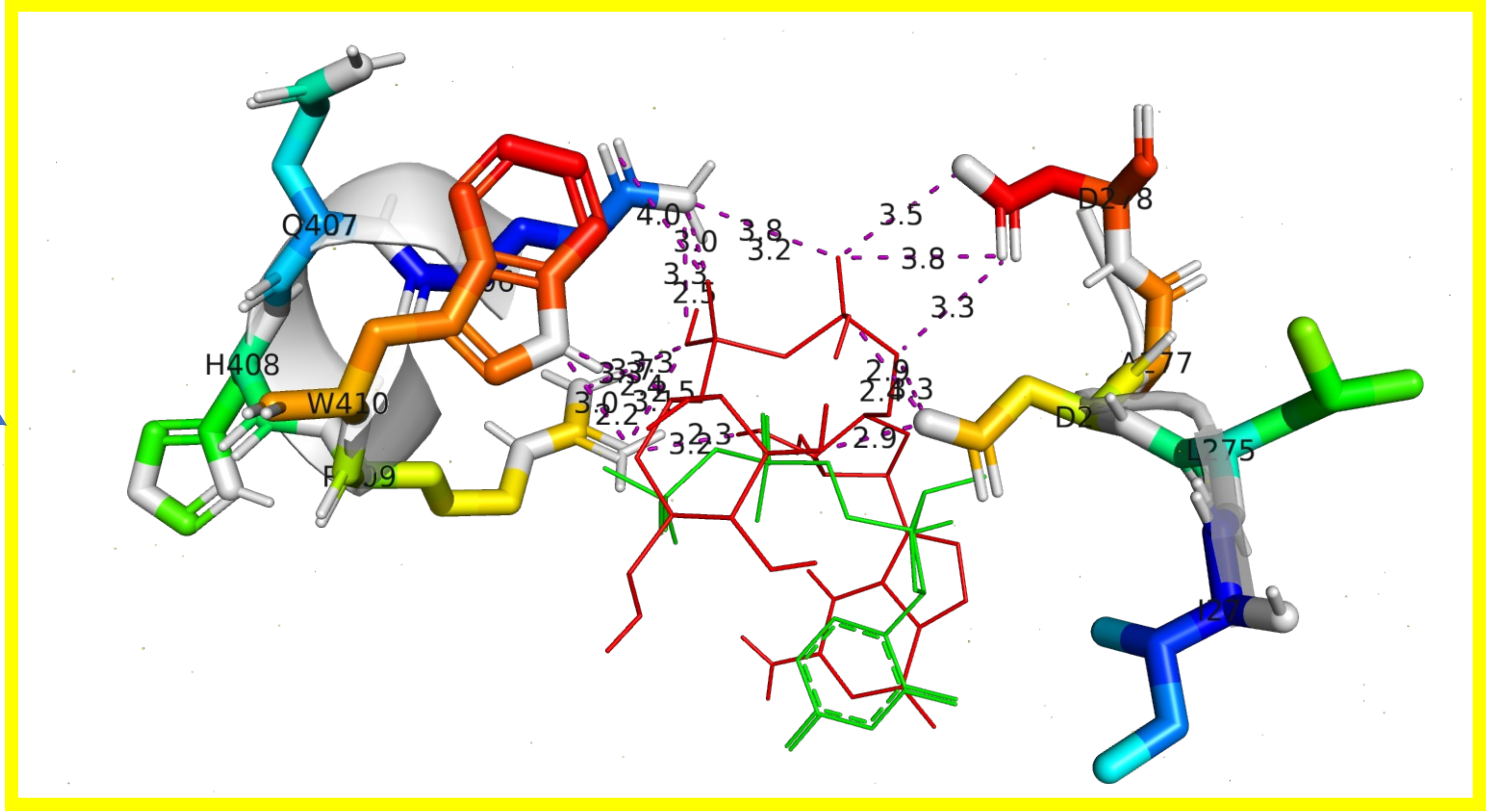

Yellow rectangle zoomed-in view of the binding sites of GDP-mannose (red lines) in ZcCslL1: sticks on the left are the QQHRW motif (406-410), on the right are the ILDADD motif (274-278). The RMSD distances between the closest atoms in GDP-mannose and in the two motifs are show indicated with dotted lines. The superimposed binding motifs of UDP-glucose (green lines) in 4HG6 are also shown as cartoon view: on the left are the QRGRW motif (379-383), on the right are the VFDAD motif (244-248).

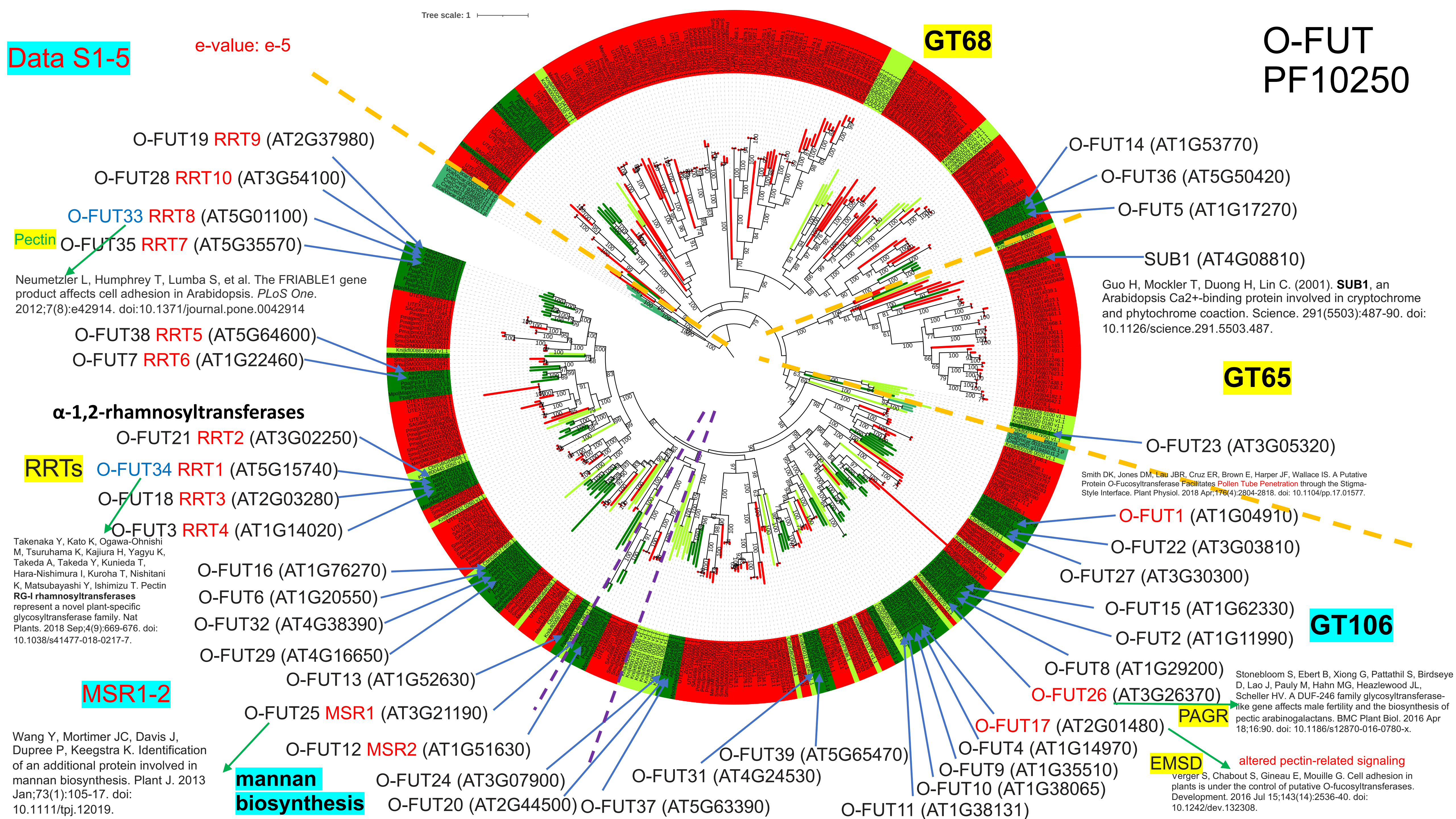
Tree scale: 1
Data S1-5
e-value: e-5
GT68
O-FUT
PF10250
O-FUT19 RRT9 (AT2G37980)
O-FUT28 RRT10 (AT3G54100)
O-FUT33 RRT8 (AT5G01100)
Pectin
O-FUT35 RRT7 (AT5G35570)
Neumetzler L, Humphrey T, Lumba S, et al. The FRIABLE1 gene product affects cell adhesion in Arabidopsis. PLoS One. 2012;7(8):e42914. doi:10.1371/journal.pone.0042914
O-FUT38 RRT5 (AT5G64600)
O-FUT7 RRT6 (AT1G22460)
α-1,2-rhamnosyltransferases
O-FUT21 RRT2 (AT3G02250)
RRTs
O-FUT34 RRT1 (AT5G15740)
O-FUT18 RRT3 (AT2G03280)
O-FUT3 RRT4 (AT1G14020)
Takenaka Y, Kato K, Ogawa-Ohnishi M, Tsuruhama K, Kajiura H, Yagyu K, Takeda A, Takeda Y, Kunieda T, Hara-Nishimura I, Kuroha T, Nishitani K, Matsubayashi Y, Ishimizu T. Pectin RG-I rhamnosyltransferases represent a novel plant-specific glycosyltransferase family. Nat Plants. 2018 Sep;4(9):669-676. doi: 10.1038/s41477-018-0217-7.
O-FUT16 (AT1G76270)
O-FUT6 (AT1G20550)
O-FUT32 (AT4G38390)
O-FUT29 (AT4G16650)
O-FUT13 (AT1G52630)
MSR1-2
O-FUT25 MSR1 (AT3G21190)
O-FUT12 MSR2 (AT1G51630)
Wang Y, Mortimer JC, Davis J, Dupree P, Keegstra K. Identification of an additional protein involved in mannan biosynthesis. Plant J. 2013 Jan;73(1):105-17. doi: 10.1111/tpj.12019.
mannan biosynthesis
O-FUT24 (AT3G07900)
O-FUT20 (AT2G44500)
O-FUT37 (AT5G63390)
O-FUT31 (AT4G24530)
O-FUT39 (AT5G65470)
O-FUT11 (AT1G38131)
O-FUT10 (AT1G38065)
O-FUT9 (AT1G35510)
O-FUT4 (AT1G14970)
O-FUT17 (AT2G01480)
EMSD
altered pectin-related signaling
Verger S, Chabout S, Gineau E, Mouille G. Cell adhesion in plants is under the control of putative O-fucosyltransferases. Development. 2016 Jul 15;143(14):2536-40. doi: 10.1242/dev.132308.
O-FUT26 (AT3G26370)
PAGR
Stonebloom S, Ebert B, Xiong G, Pattathil S, Birdseye D, Lao J, Pauly M, Hahn MG, Heazlewood JL, Scheller HV. A DUF-246 family glycosyltransferase-like gene affects male fertility and the biosynthesis of pectic arabinogalactans. BMC Plant Biol. 2016 Apr 18;16:90. doi: 10.1186/s12870-016-0780-x.
O-FUT8 (AT1G29200)
O-FUT2 (AT1G11990)
GT106
O-FUT15 (AT1G62330)
O-FUT27 (AT3G30300)
O-FUT22 (AT3G03810)
O-FUT1 (AT1G04910)
Smith DK, Jones DM, Lau JBR, Cruz ER, Brown E, Harper JF, Wallace IS. A Putative Protein O-Fucosyltransferase Facilitates Pollen Tube Penetration through the Stigma-Style Interface. Plant Physiol. 2018 Apr;176(4):2804-2818. doi: 10.1104/pp.17.01577.
O-FUT23 (AT3G05320)
GT65
SUB1 (AT4G08810)
Guo H, Mockler T, Duong H, Lin C. (2001). SUB1, an Arabidopsis Ca2+-binding protein involved in cryptochrome and phytochrome coaction. Science. 291(5503):487-90. doi: 10.1126/science.291.5503.487.
O-FUT5 (AT1G17270)
O-FUT36 (AT5G50420)
O-FUT14 (AT1G53770)

Data S1-6

GT34

Xyloglucan

XXT5 (AT1G74380)

XXT4 (AT1G18690)

XXT3 (AT5G07720)

XXT2 (AT4G02500)

XXT1 (AT3G62720)

Faik A, Price NJ, Raikhel NV, Keegstra K. An Arabidopsis gene encoding an alpha-xylosyltransferase involved in xyloglucan biosynthesis. Proc Natl Acad Sci U S A. 2002 May 28;99(11):7797-802. doi: 10.1073/pnas.102644799.

Cavalier DM, Keegstra K. Two xyloglucan xylosyltransferases catalyze the addition of multiple xylosyl residues to cellohexaose. J Biol Chem. 2006 Nov 10;281(45):34197-207. doi: 10.1074/jbc.M606379200.

Vuttipongchaikij S, Brocklehurst D, Steele-King C, Ashford DA, Gomez LD, McQueen-Mason SJ. Arabidopsis GT34 family contains five xyloglucan α-1,6-xylosyltransferases. New Phytol. 2012 Aug;195(3):585-595. doi: 10.1111/j.1469-8137.2012.04196.x.

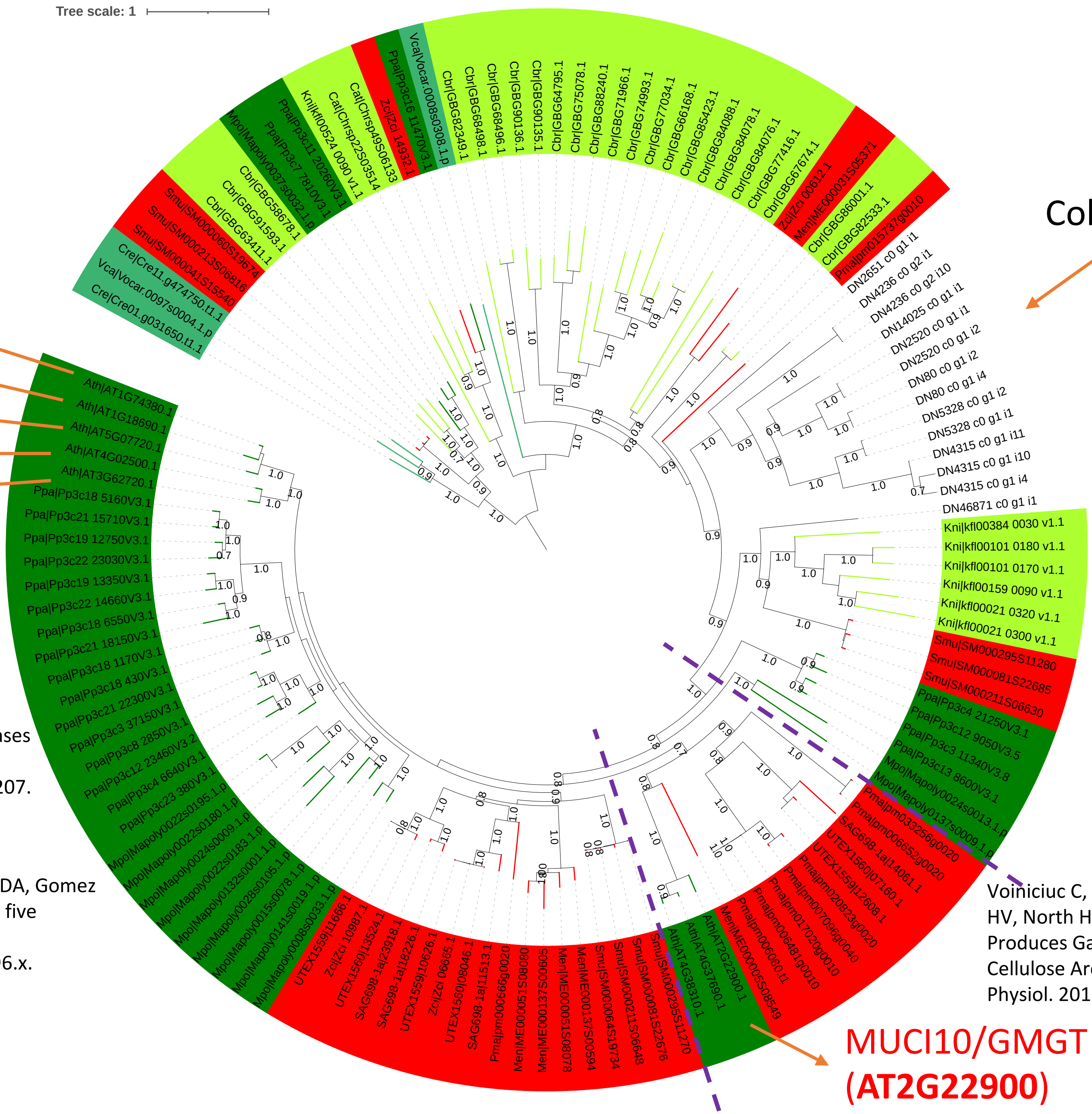

Coleochaete scutate

Mannan

galactomannan α-1,6-galactosyltransferase activity

Voiniciuc C, Schmidt MH, Berger A, Yang B, Ebert B, Scheller HV, North HM, Usadel B, Günl M. MUCILAGE-RELATED10 Produces Galactoglucomannan That Maintains Pectin and Cellulose Architecture in Arabidopsis Seed Mucilage. Plant Physiol. 2015 Sep;169(1):403-20. doi: 10.1104/pp.15.00851.

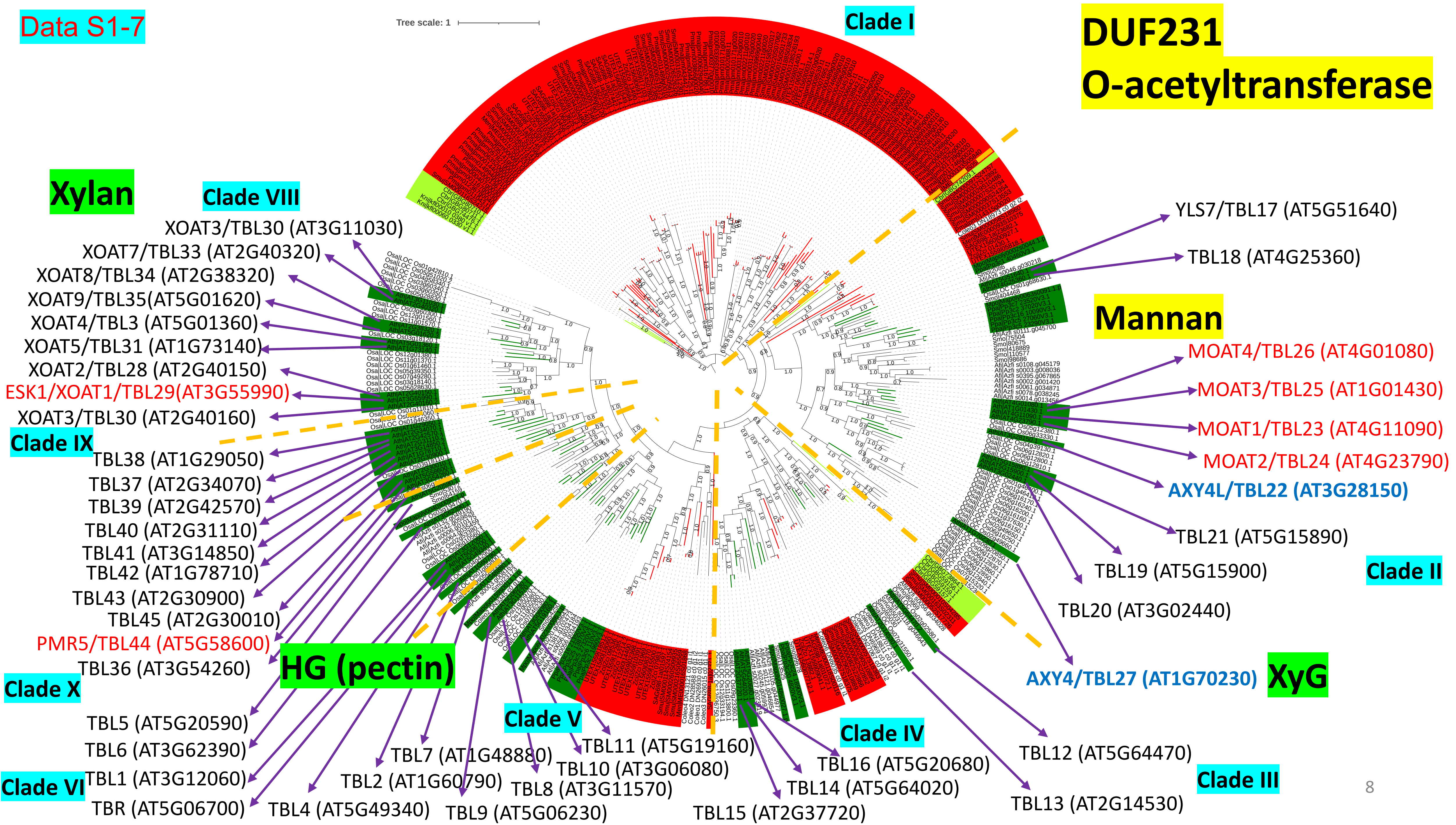
Data S1-7
Tree scale: 1
Clade I
DUF231
O-acetyltransferase
Xylan
Clade VIII
XOAT3/TBL30 (AT3G11030)
XOAT7/TBL33 (AT2G40320)
XOAT8/TBL34 (AT2G38320)
XOAT9/TBL35(AT5G01620)
XOAT4/TBL3 (AT5G01360)
XOAT5/TBL31 (AT1G73140)
XOAT2/TBL28 (AT2G40150)
ESK1/XOAT1/TBL29(AT3G55990)
XOAT3/TBL30 (AT2G40160)
Clade IX
TBL38 (AT1G29050)
TBL37 (AT2G34070)
TBL39 (AT2G42570)
TBL40 (AT2G31110)
TBL41 (AT3G14850)
TBL42 (AT1G78710)
TBL43 (AT2G30900)
TBL45 (AT2G30010)
PMR5/TBL44 (AT5G58600)
TBL36 (AT3G54260)
HG (pectin)
Clade X
TBL5 (AT5G20590)
TBL6 (AT3G62390)
TBL1 (AT3G12060)
Clade VI
TBR (AT5G06700)
TBL4 (AT5G49340)
TBL2 (AT1G60790)
TBL7 (AT1G48880)
TBL9 (AT5G06230)
TBL8 (AT3G11570)
Clade V
TBL10 (AT3G06080)
TBL11 (AT5G19160)
TBL15 (AT2G37720)
TBL14 (AT5G64020)
TBL16 (AT5G20680)
Clade IV
TBL13 (AT2G14530)
TBL12 (AT5G64470)
Clade III
AXY4/TBL27 (AT1G70230)
XyG
TBL20 (AT3G02440)
TBL19 (AT5G15900)
Clade II
TBL21 (AT5G15890)
AXY4L/TBL22 (AT3G28150)
MOAT2/TBL24 (AT4G23790)
MOAT1/TBL23 (AT4G11090)
MOAT3/TBL25 (AT1G01430)
MOAT4/TBL26 (AT4G01080)
Mannan
TBL18 (AT4G25360)
YLS7/TBL17 (AT5G51640)

Data S1-8

GH5_7

endo-β-1,4-mannanase (EC 3.2.1.78)

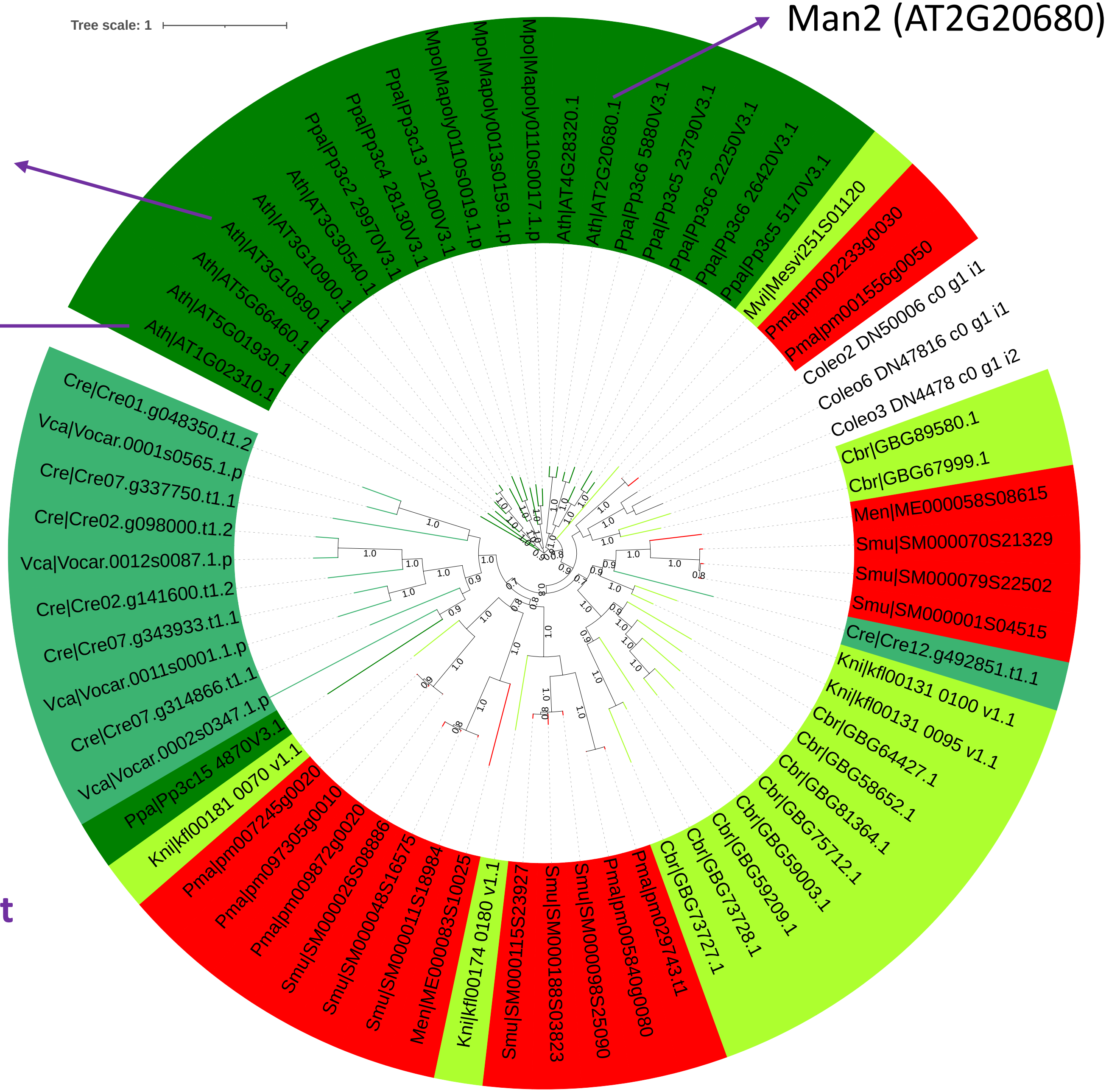

Mannan biosynthesis and degradation first appeared in the LCA of Chloroplastida

Data S1-9

GH5_7 (nr)

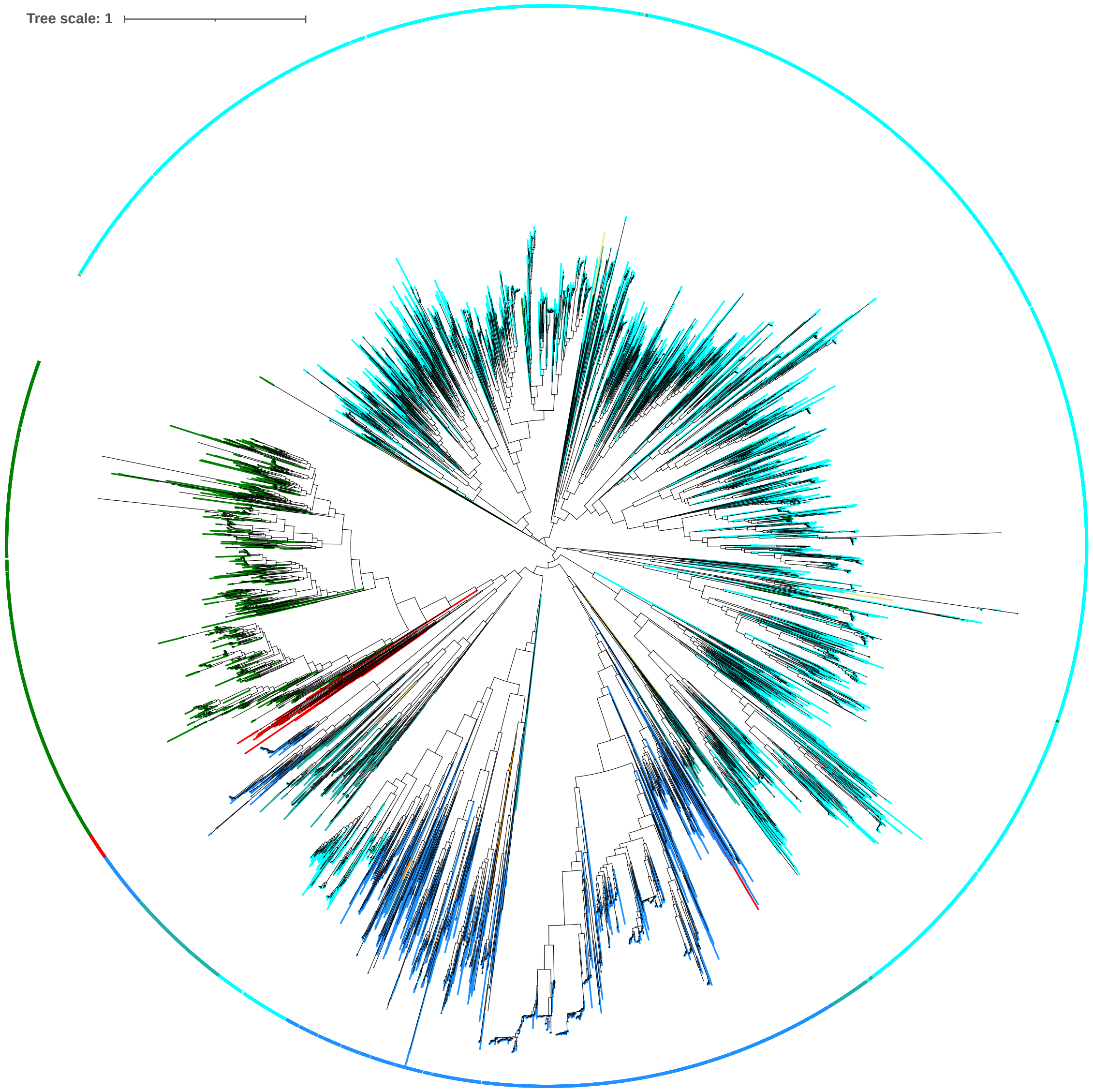

Possible HGT from bacteria

https://itol.embl.de/tree/134238164171357031667587420

Data S1-10

GH27 nr

Imaizumi et al. Heterologous expression and characterization of an Arabidopsis β-l-arabinopyranosidase and α-d-galactosidases acting on β-l-arabinopyranosyl residues. J Exp Bot. 2017 Jul 20;68(16):4651-4661. doi: 10.1093/jxb/erx279.

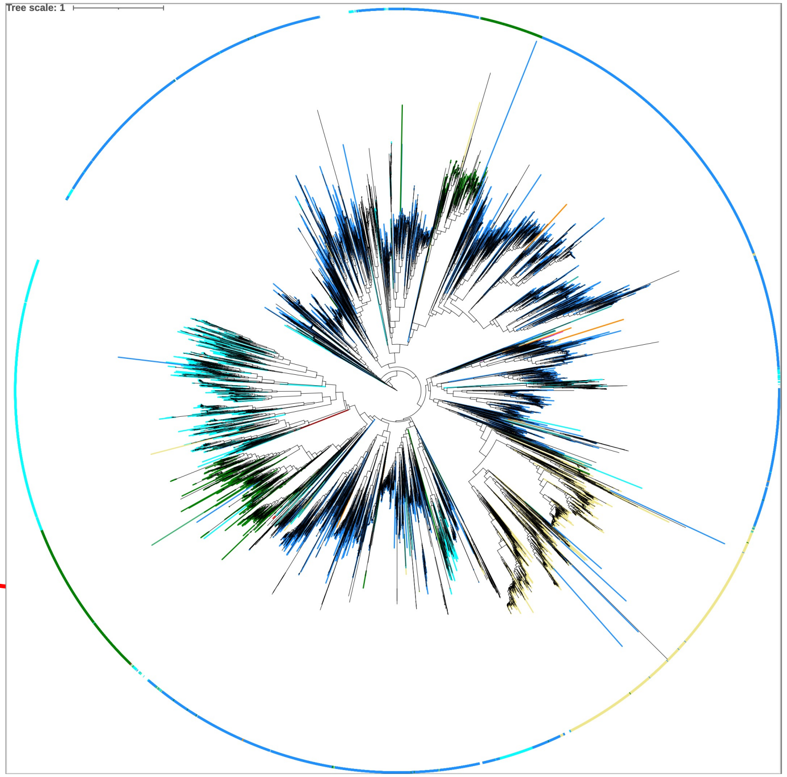

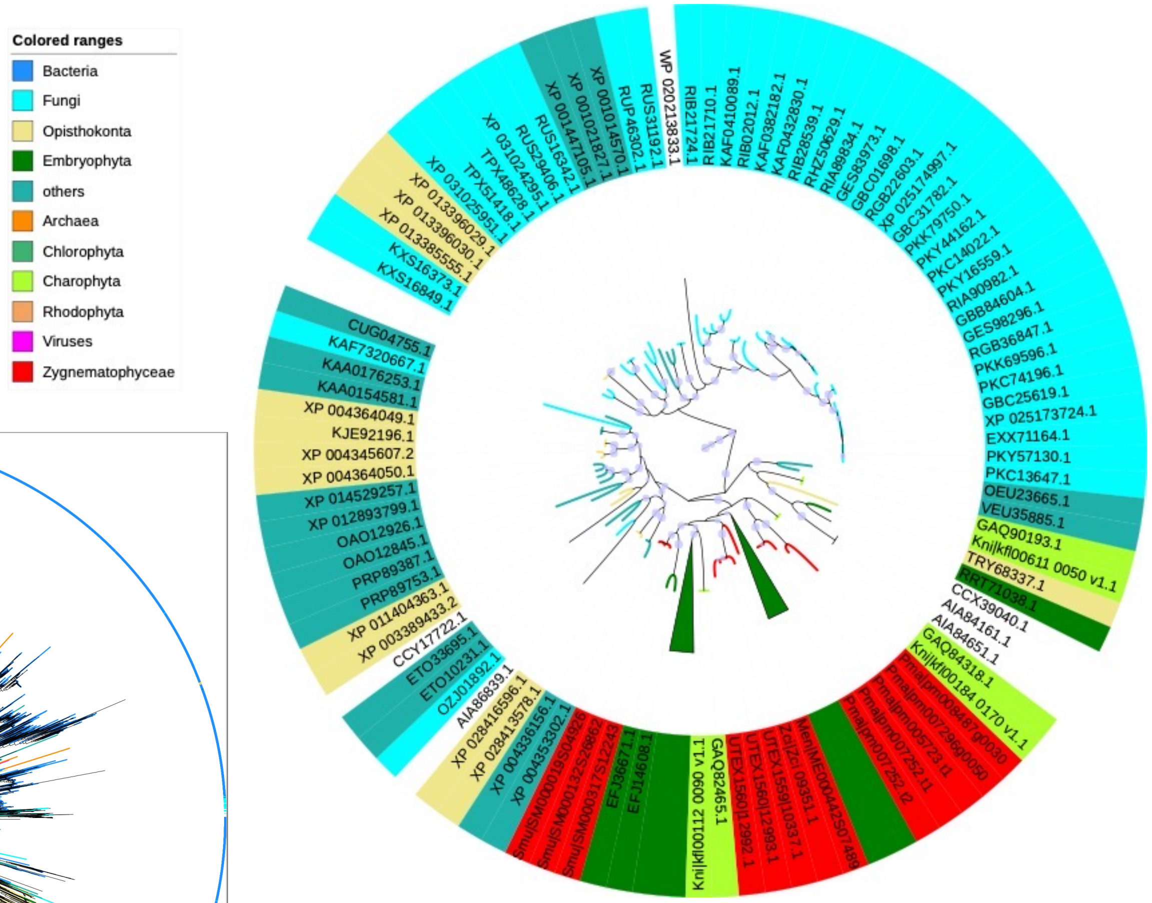

AGAL1 (AT5G08380)
AGAL2 (AT5G08370)
AGAL3 (AT3G56310)

might have been gained via HGT between fungi and Klebsormidium

https://itol.embl.de/tree/20812724022256331665203458

Data S1-11

https://itol.embl.de/tree/134238164171203571671560784

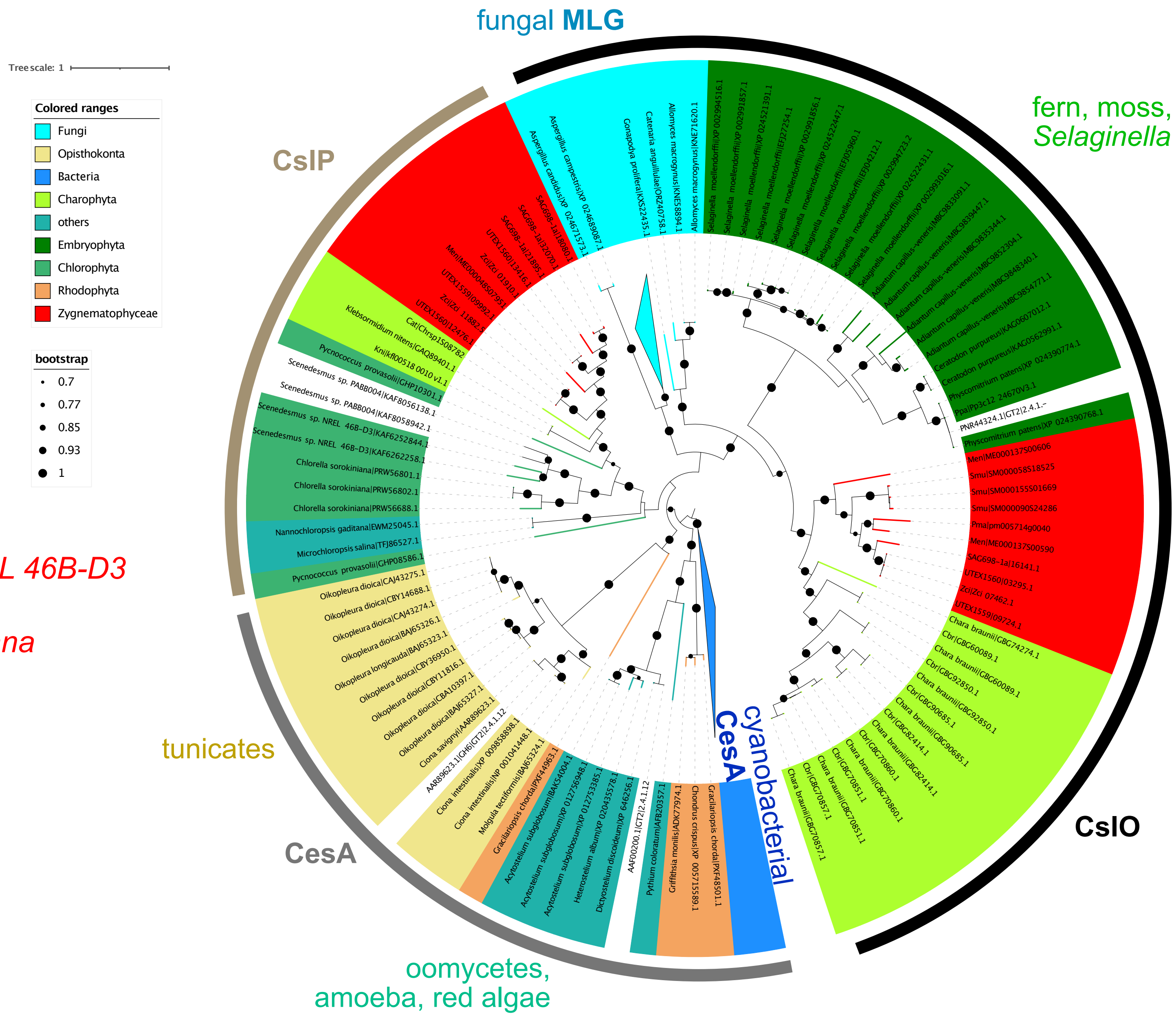

Embryophyta:
Adiantum capillus-veneris (fern)
Selaginella moellendorffii (spike moss)
Ceratodon purpureus (moss)
Physcomitrium patens (moss)

Chlorophyta:
Scenedesmus sp. NREL 46B-D3
Stramenopiles:
Nannochloropsis gaditana

Data S1-12

https://itol.embl.de/tree/208127240222143781672028870

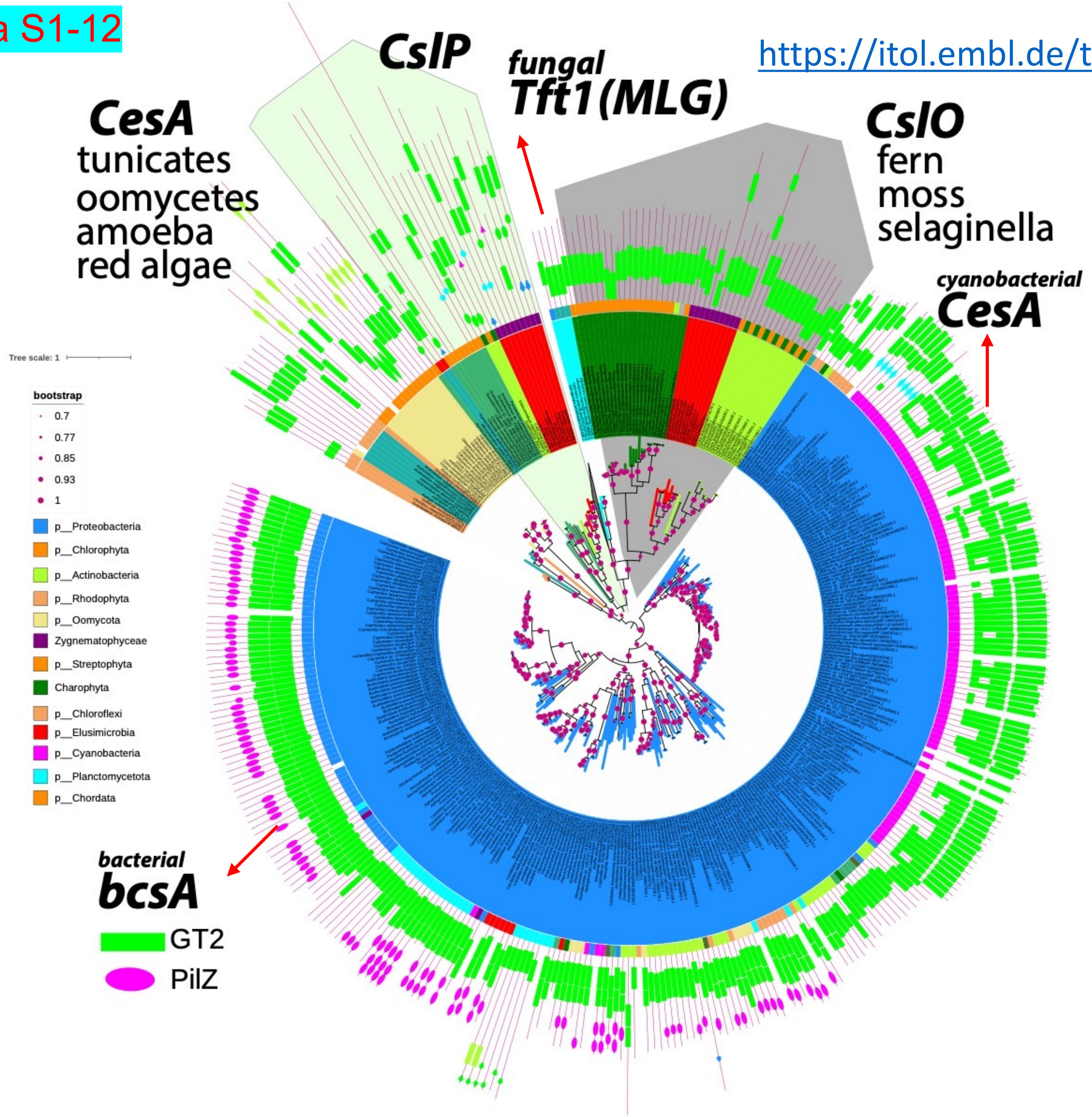

Data S1-13

https://itol.embl.de/tree/2081272402224417016711255554

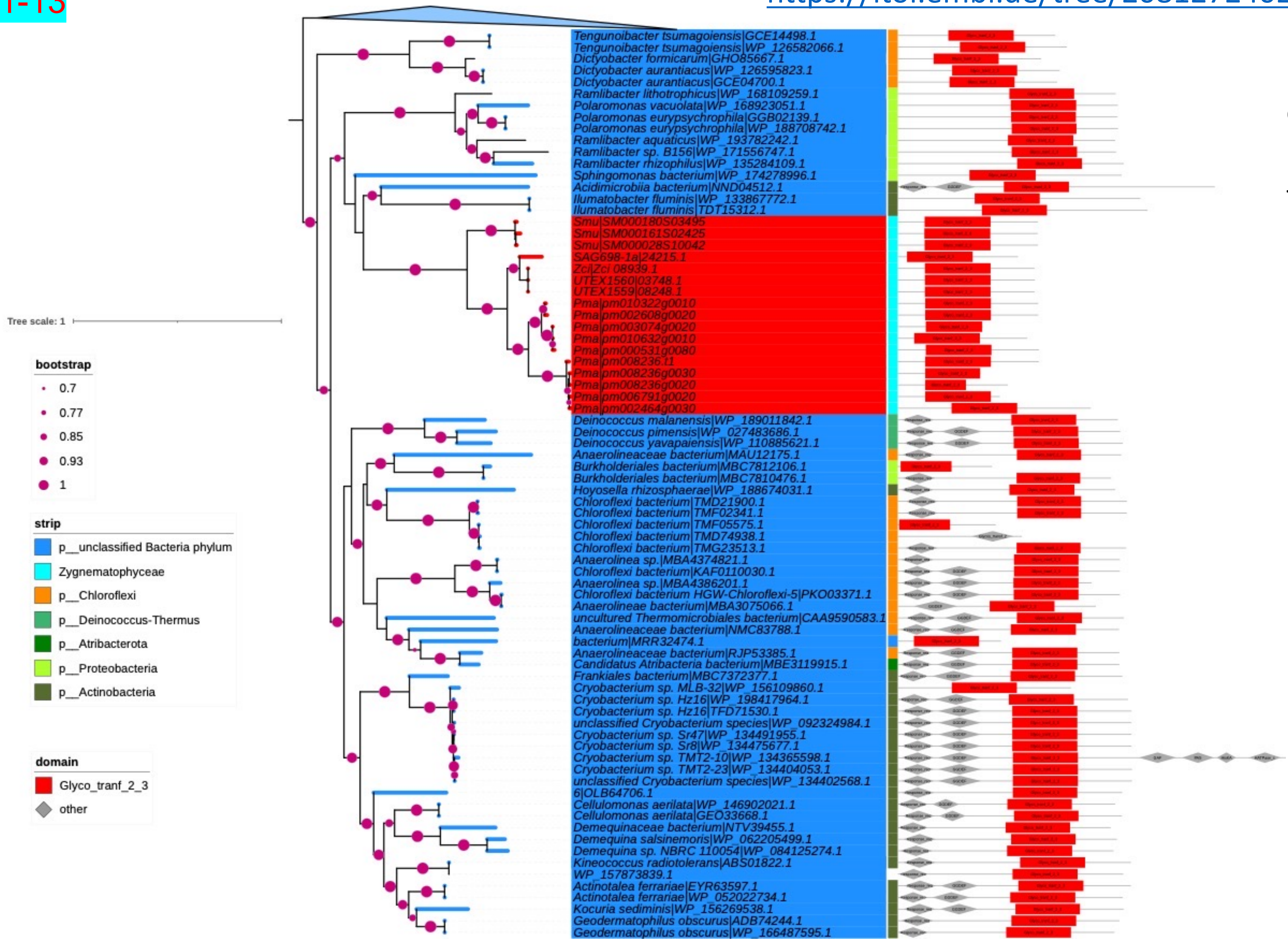

CslN might have been gained via HGT from bacteria (bcsA)

Data S1-14

Xyloglucan

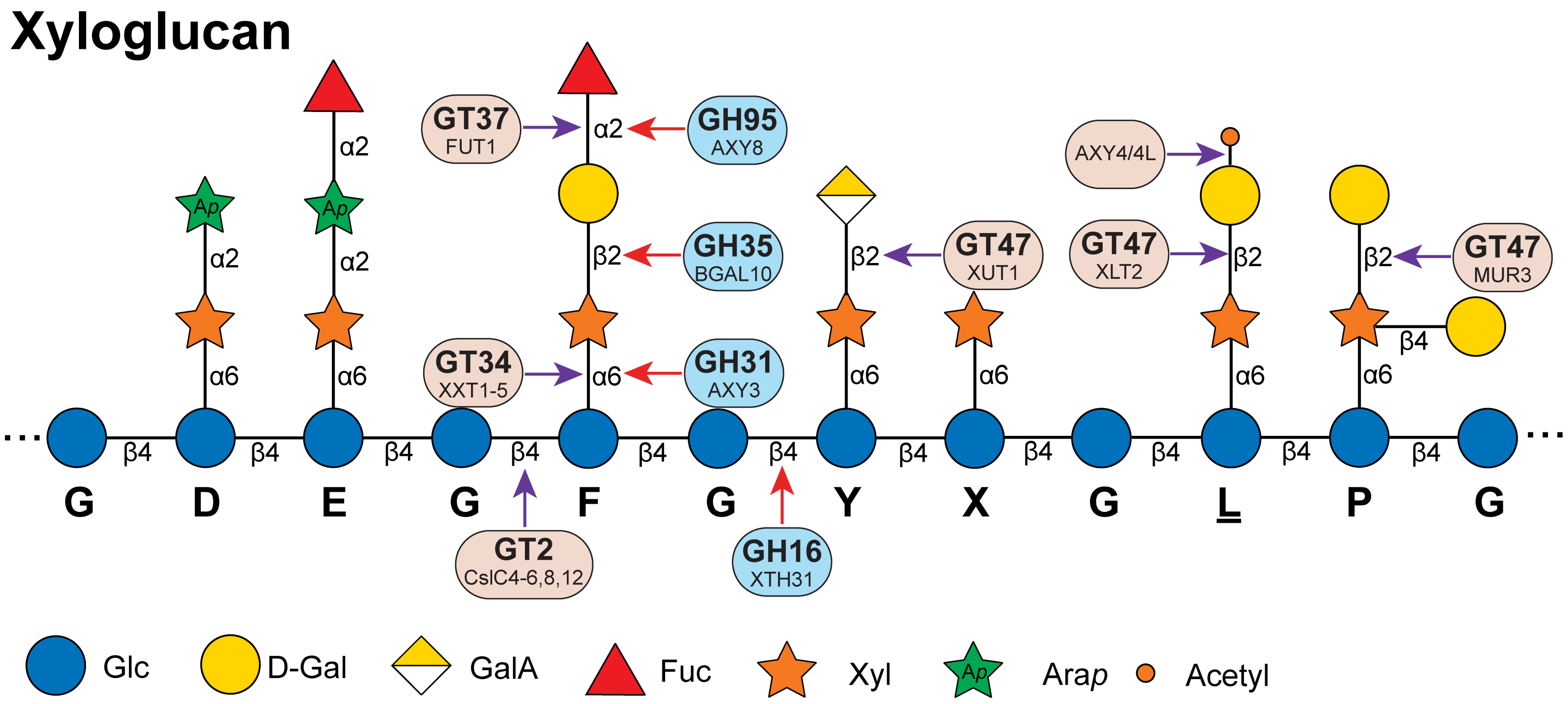

Data S1-15

GT34

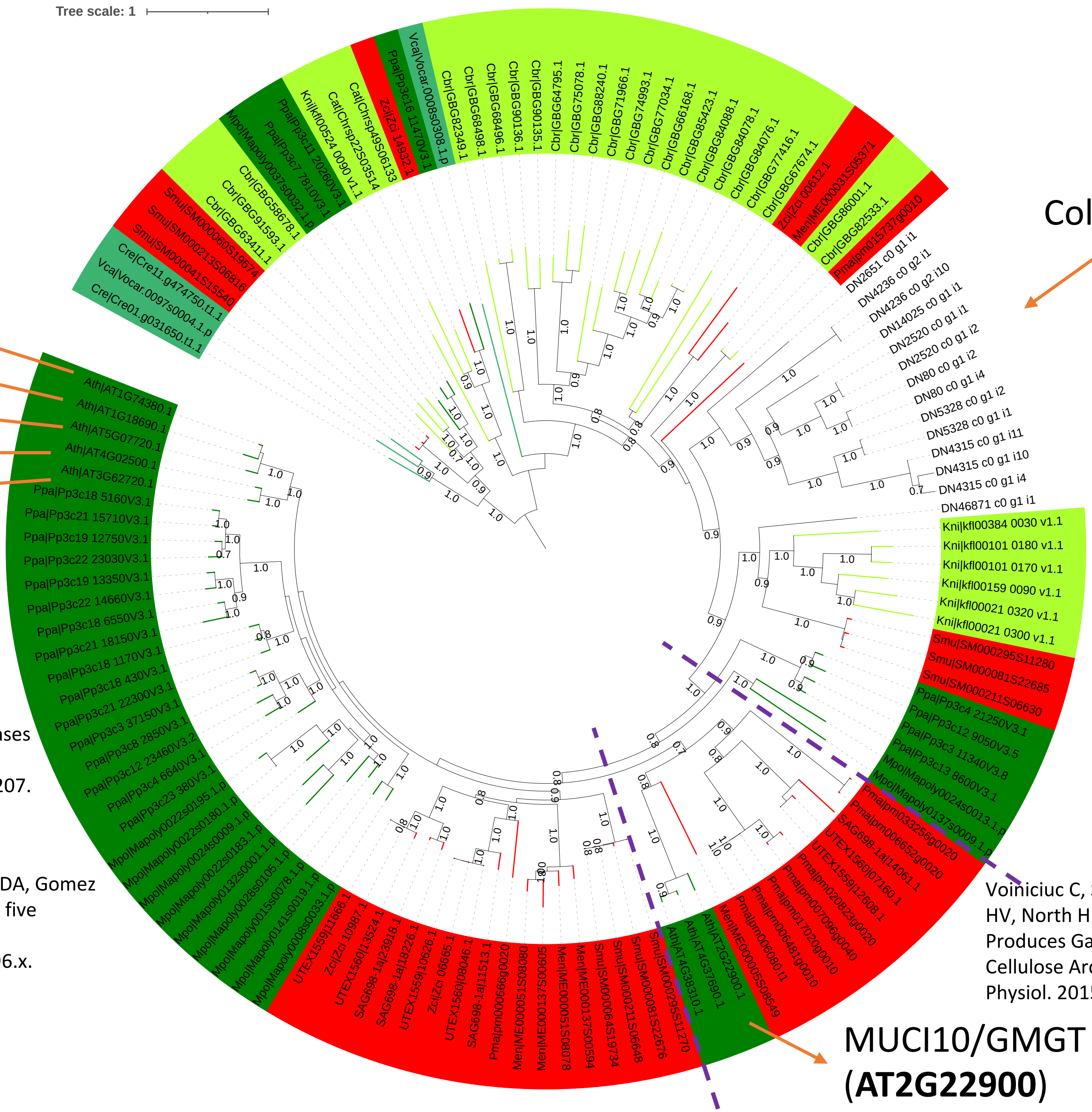

Xyloglucan

- XXT5 (AT1G74380)
- XXT4 (AT1G18690)
- XXT3 (AT5G07720)
- XXT2 (AT4G02500)
- XXT1 (AT3G62720)

Faik A, Price NJ, Raikhel NV, Keegstra K. An Arabidopsis gene encoding an alpha-xylosyltransferase involved in xyloglucan biosynthesis. Proc Natl Acad Sci U S A. 2002 May 28;99(11):7797-802. doi: 10.1073/pnas.102644799.

Cavalier DM, Keegstra K. Two xyloglucan xylosyltransferases catalyze the addition of multiple xylosyl residues to cellohexaose. J Biol Chem. 2006 Nov 10;281(45):34197-207. doi: 10.1074/jbc.M606379200.

Vuttipongchaikij S, Brocklehurst D, Steele-King C, Ashford DA, Gomez LD, McQueen-Mason SJ. Arabidopsis GT34 family contains five xyloglucan α-1,6-xylosyltransferases. New Phytol. 2012 Aug;195(3):585-595. doi: 10.1111/j.1469-8137.2012.04196.x.

Coleochaete scutate

Mannan

galactomannan α-1,6-galactosyltransferase activity

Voiniciuc C, Schmidt MH, Berger A, Yang B, Ebert B, Scheller HV, North HM, Usadel B, Günl M. MUCILAGE-RELATED10 Produces Galactoglucomannan That Maintains Pectin and Cellulose Architecture in Arabidopsis Seed Mucilage. Plant Physiol. 2015 Sep;169(1):403-20. doi: 10.1104/pp.15.00851.

MUCI10/GMGT (**AT2G22900**)

GT47

Data S1-16

Madson M, Dunand C, Li X, Verma R, Vanzin GF, Caplan J, Shoue DA, Carpita NC, Reiter WD. The MUR3 gene of Arabidopsis encodes a xyloglucan galactosyltransferase that is evolutionarily related to animal exostosins. Plant Cell. 2003 Jul;15(7):1662-70. doi: 10.1105/tpc.009837.

XyG

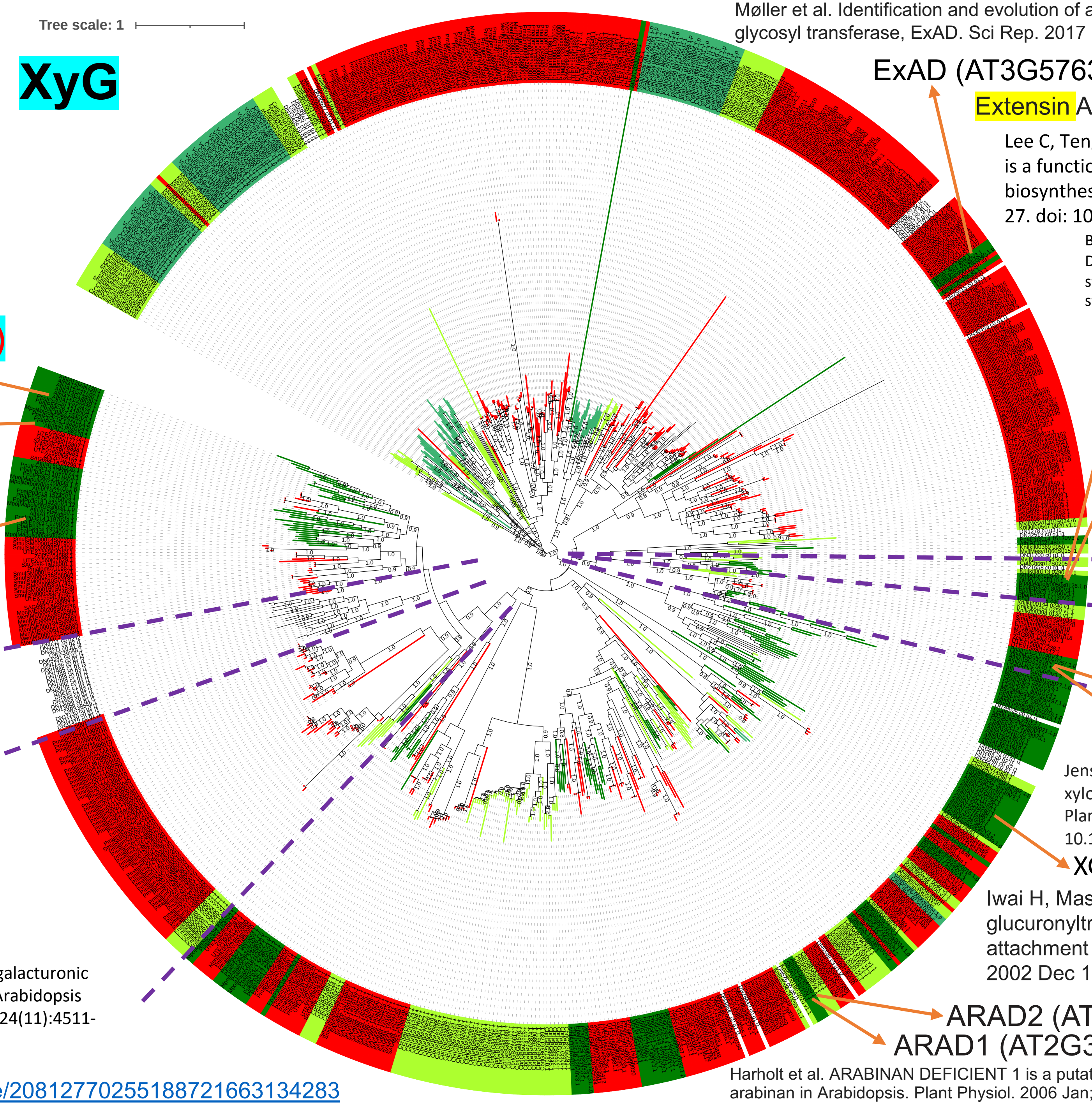

MUR3 (AT2G20370)

XLT2 (AT5G62220)

XUT1 (AT1G63450)

Coleochaete scutate
Coleochaete orbicularis

Jensen JK, Schultink A, Keegstra K, Wilkerson CG, Pauly M. RNA-Seq analysis of developing nasturtium seeds (Tropaeolum majus): identification and characterization of an additional galactosyltransferase involved in xyloglucan biosynthesis. Mol Plant. 2012 Sep;5(5):984-92. doi: 10.1093/mp/sss032.

Peña MJ, Kong Y, York WS, O'Neill MA. A galacturonic acid-containing xyloglucan is involved in Arabidopsis root hair tip growth. Plant Cell. 2012 Nov;24(11):4511-24. doi: 10.1105/tpc.112.103390.

Møller et al. Identification and evolution of a plant cell wall specific glycoprotein glycosyl transferase, ExAD. Sci Rep. 2017 Mar 30;7:45341. doi: 10.1038/srep45341

ExAD (AT3G57630)

Extensin Arabinose Deficient transferase

Lee C, Teng Q, Huang W, Zhong R, Ye ZH. The F8H glycosyltransferase is a functional paralog of FRA8 involved in glucuronoxylan biosynthesis in Arabidopsis. Plant Cell Physiol. 2009 Apr;50(4):812-27. doi: 10.1093/pcp/pcp025. Epub 2009 Feb 18. PMID: 19224953.

Brown, D.M., Goubet, F., Wong, V.W., Goodacre, R., Stephens, E., Dupree, P., and Turner, S.R. (2007). Comparison of five xylan synthesis mutants reveals new insight into the mechanisms of xylan synthesis. Plant J. 52: 1154–1168.

F8H (AT5G22940)

IRX7/FRA8 (AT2G28110)

NpGUT1: pectin b-glucuronyltransferase transferring glucuronic acid to RG-II

Urbanowicz BR, Peña MJ, Moniz HA, Moremen KW, York WS. 2014. Two Arabidopsis proteins synthesize acetylated xylan in vitro. Plant Journal 80: 197–206.

Jensen JK, Busse-Wicher M, Poulsen CP, Fangel JU, Smith PJ, Yang JY, Peña MJ, Dinesen MH, Martens HJ, Melkonian M et al. 2018. Identification of an algal xylan synthase indicates that there is functional orthology between algal and plant cell wall biosynthesis. New Phytologist 218: 1049–1060.

XYS1/IRX10-L (AT5G61840)

IRX10 (AT1G27440)

Xylan

Jensen et al. Identification of a xylogalacturonan xylosyltransferase involved in pectin biosynthesis in Arabidopsis. Plant Cell. 2008 May;20(5):1289-302. doi: 10.1105/tpc.107.050906

XGD1 (AT5G33290)

XGA

Iwai H, Masaoka N, Ishii T, Satoh S. A pectin glucuronyltransferase gene is essential for intercellular attachment in the plant meristem. Proc Natl Acad Sci U S A. 2002 Dec 10;99(25):16319-24. doi: 10.1073/pnas.252530499.

ARAD2 (AT5G44930)

ARAD1 (AT2G35100)

RG-I

Harholt et al. ARABINAN DEFICIENT 1 is a putative arabinosyltransferase involved in biosynthesis of pectic arabinan in Arabidopsis. Plant Physiol. 2006 Jan;140(1):49-58. doi: 10.1104/pp.105.072744.

https://itol.embl.de/tree/20812770255188721663134283

Data S1-17

GT47

https://itol.embl.de/tree/2081278868477251663179754

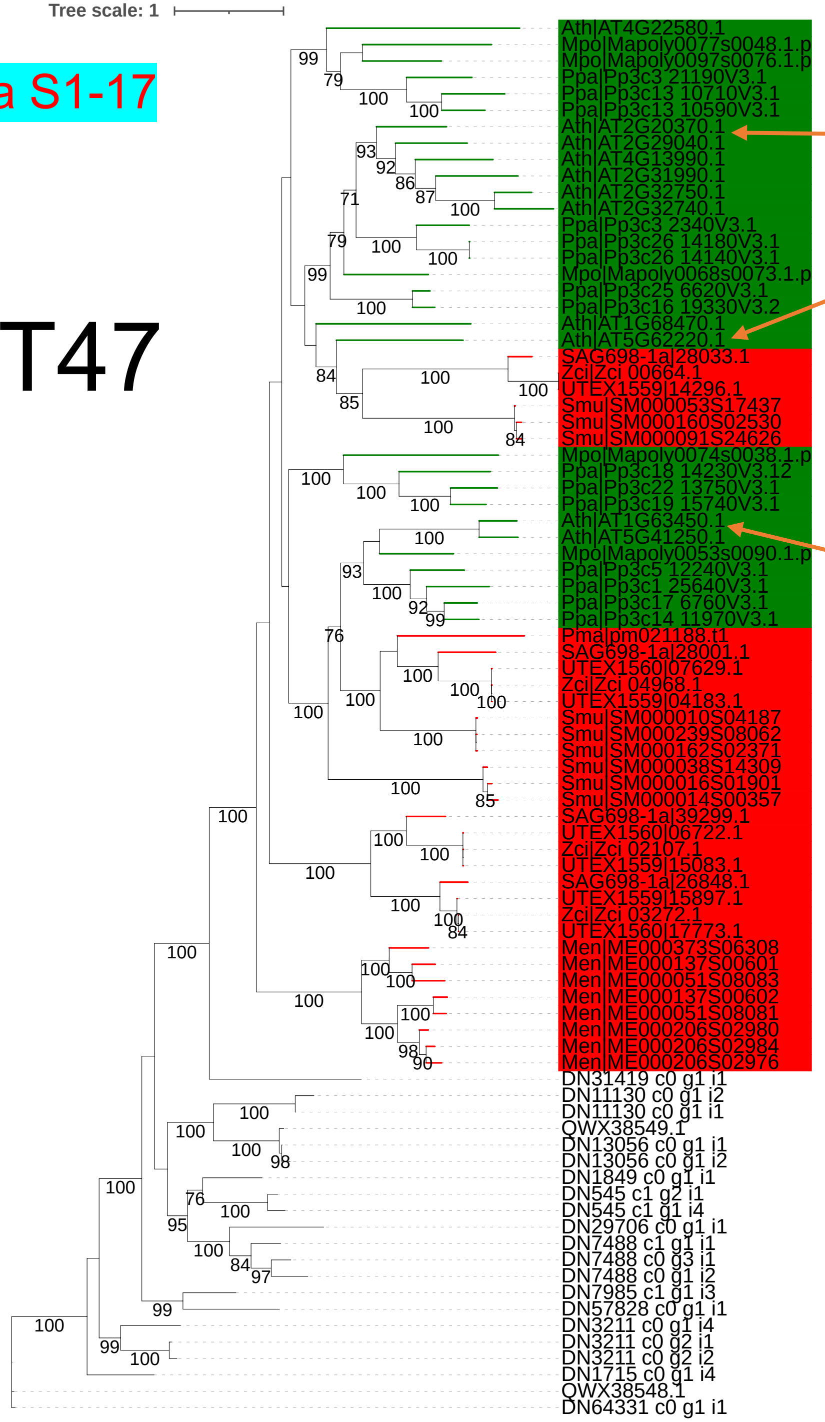

MUR3 (AT2G20370)

XLT2 (AT5G62220)

XUT1 (AT1G63450)

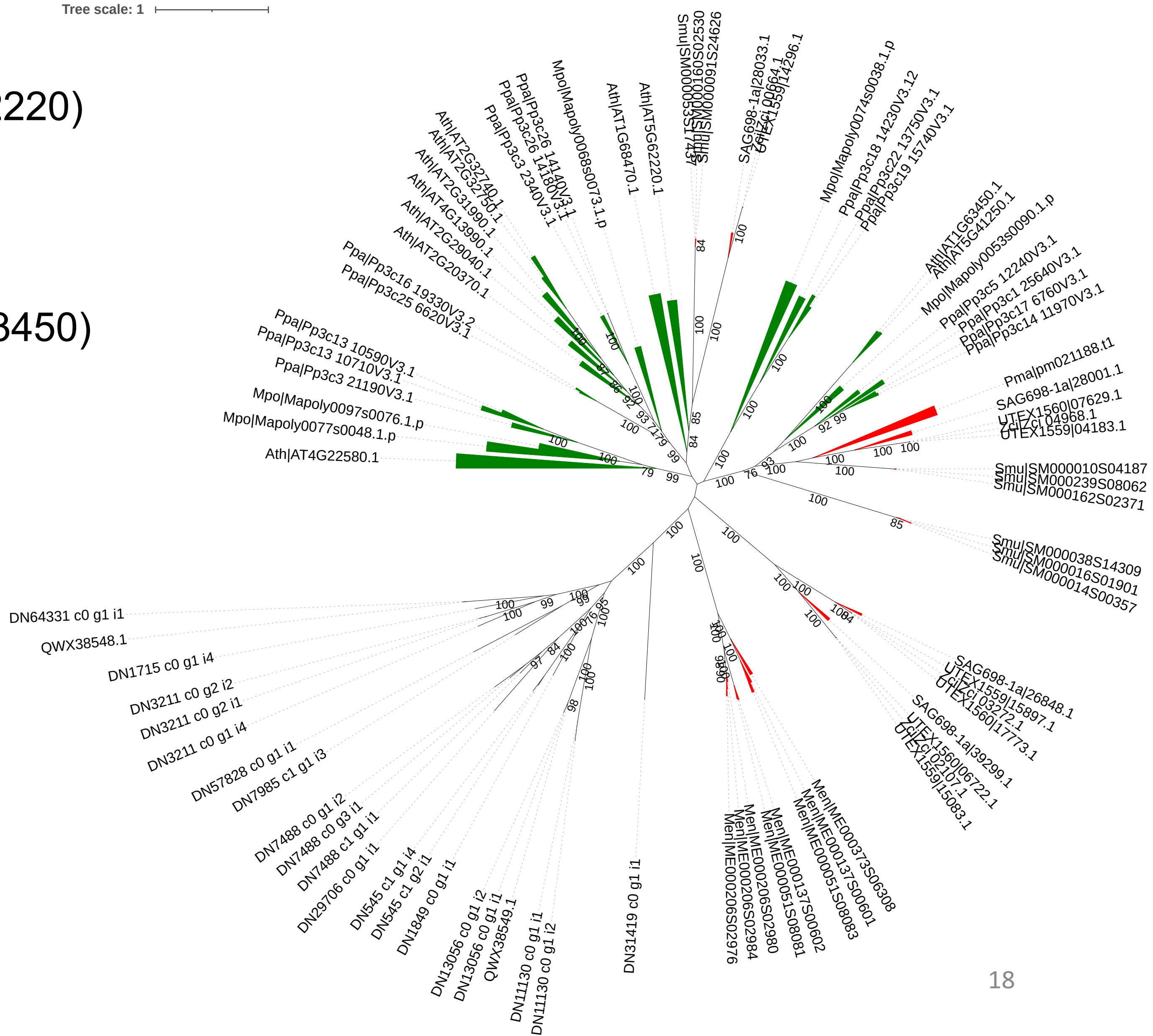

Data S1-18

GT37

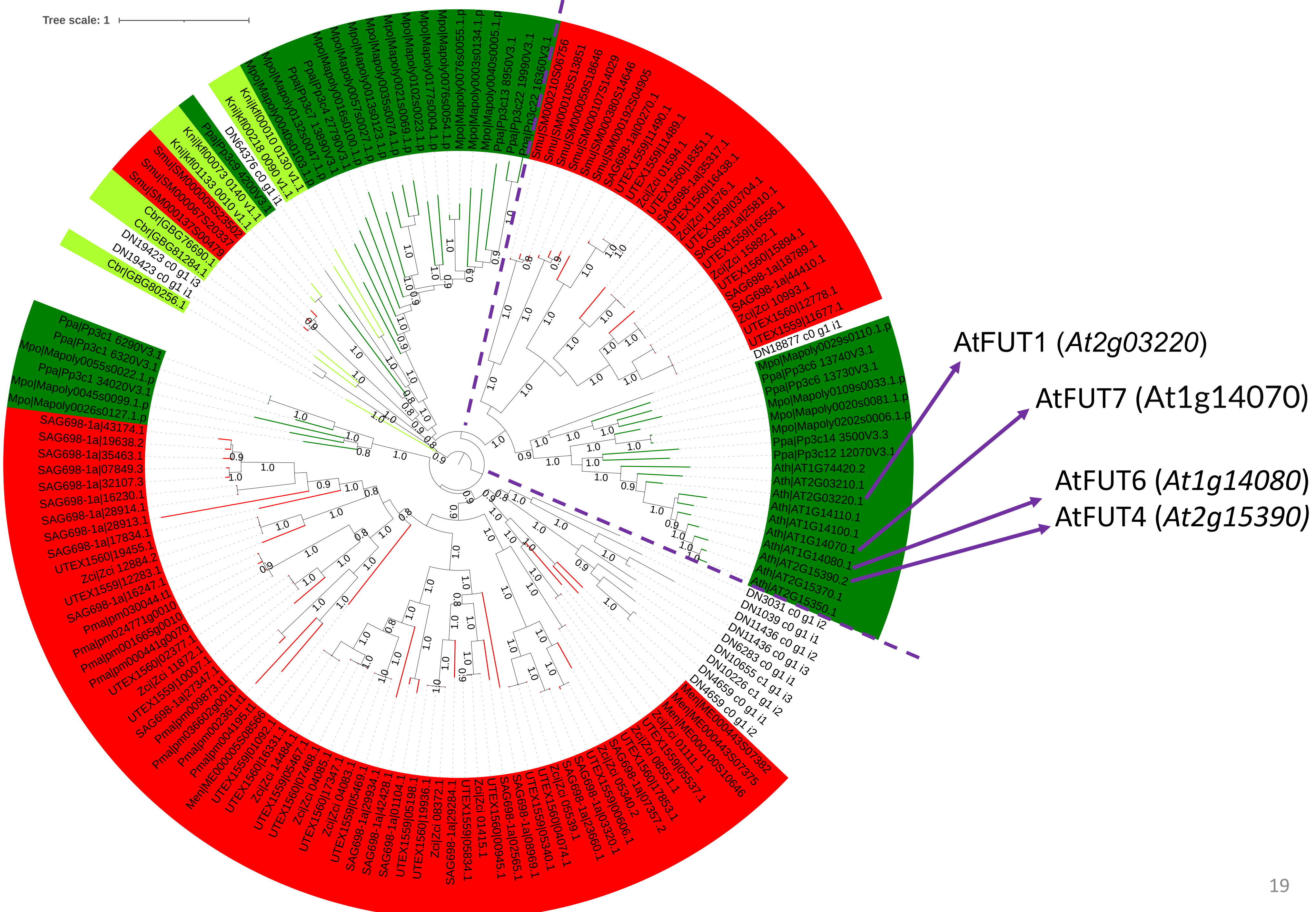

Data S1-19

https://itol.embl.de/tree/1342381641712575816691445811

GT37 nr

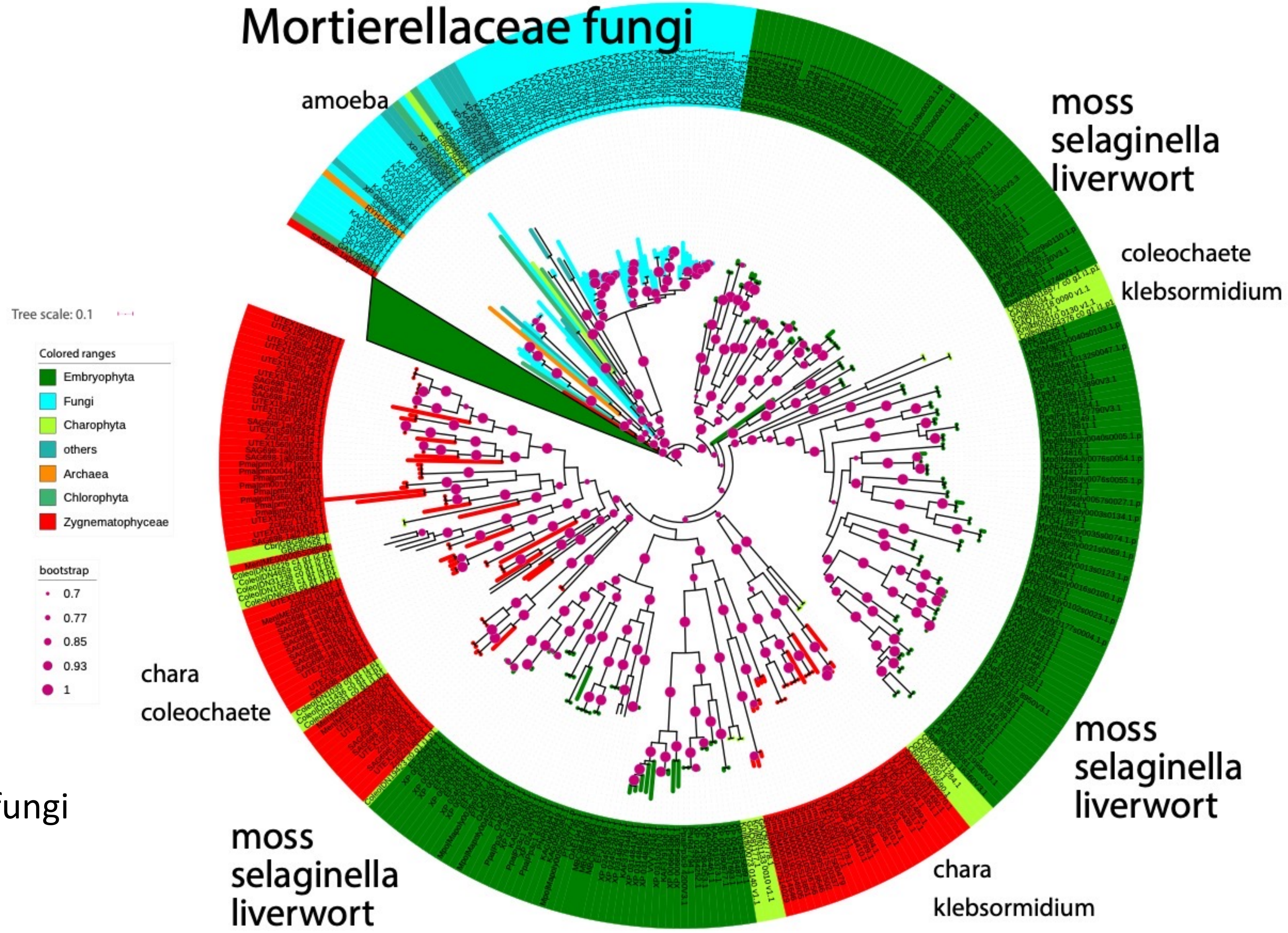

might have been
gained via HGT from fungi

Data S1-20

GH16_20

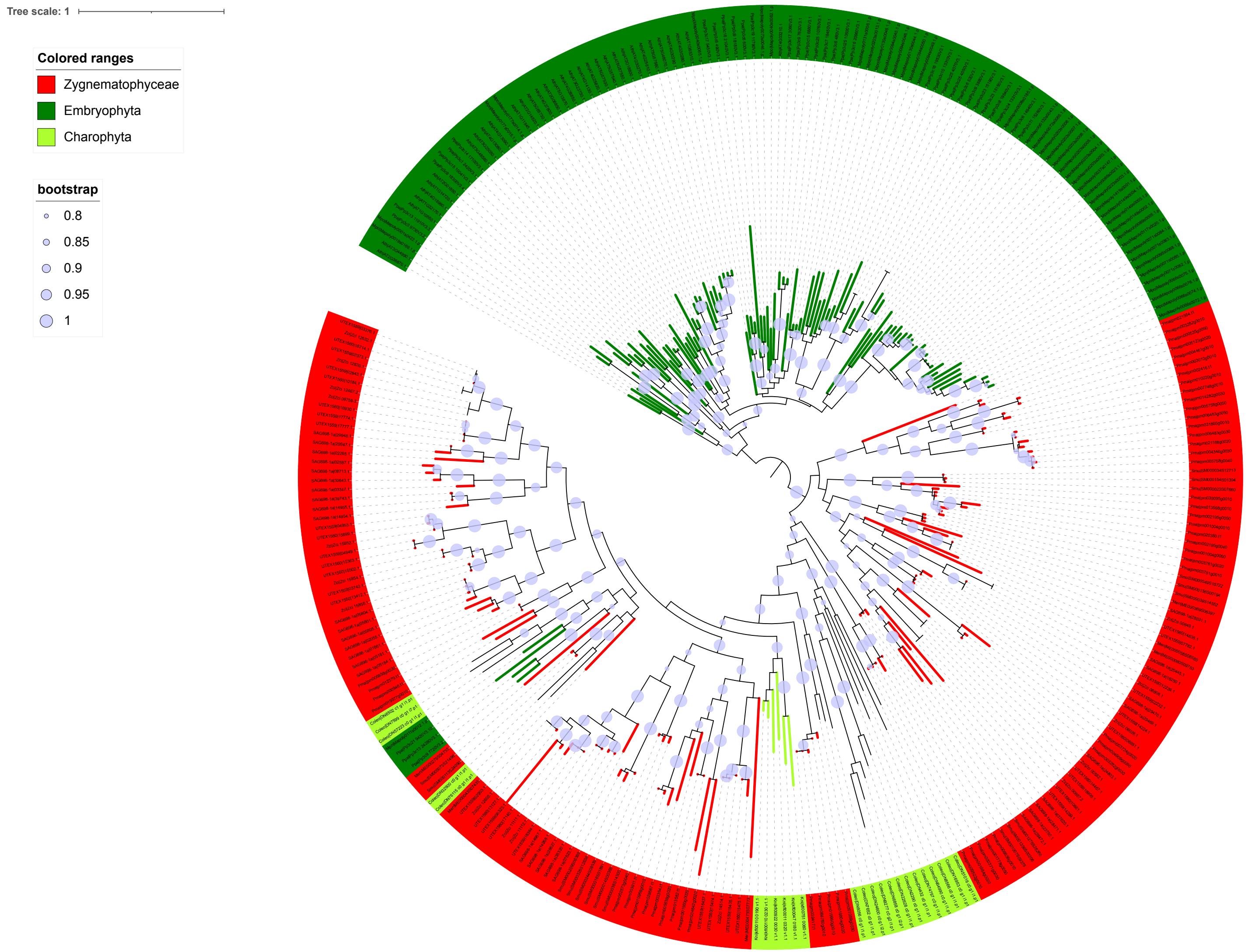

xyloglucan endotransferase

XTH4 (AT2G06850)
XTH15 (AT4G14130)
XTH14 (AT4G25820)
XTH24 (AT4G30270)
XTH22 (AT5G57560)
XTH32 (AT2G36870)
XTH31 (AT3G44990)

xyloglucanase

https://itol.embl.de/tree/20812724022342461668379872

https://itol.embl.de/tree/134238164171126381664207231

GH16_20

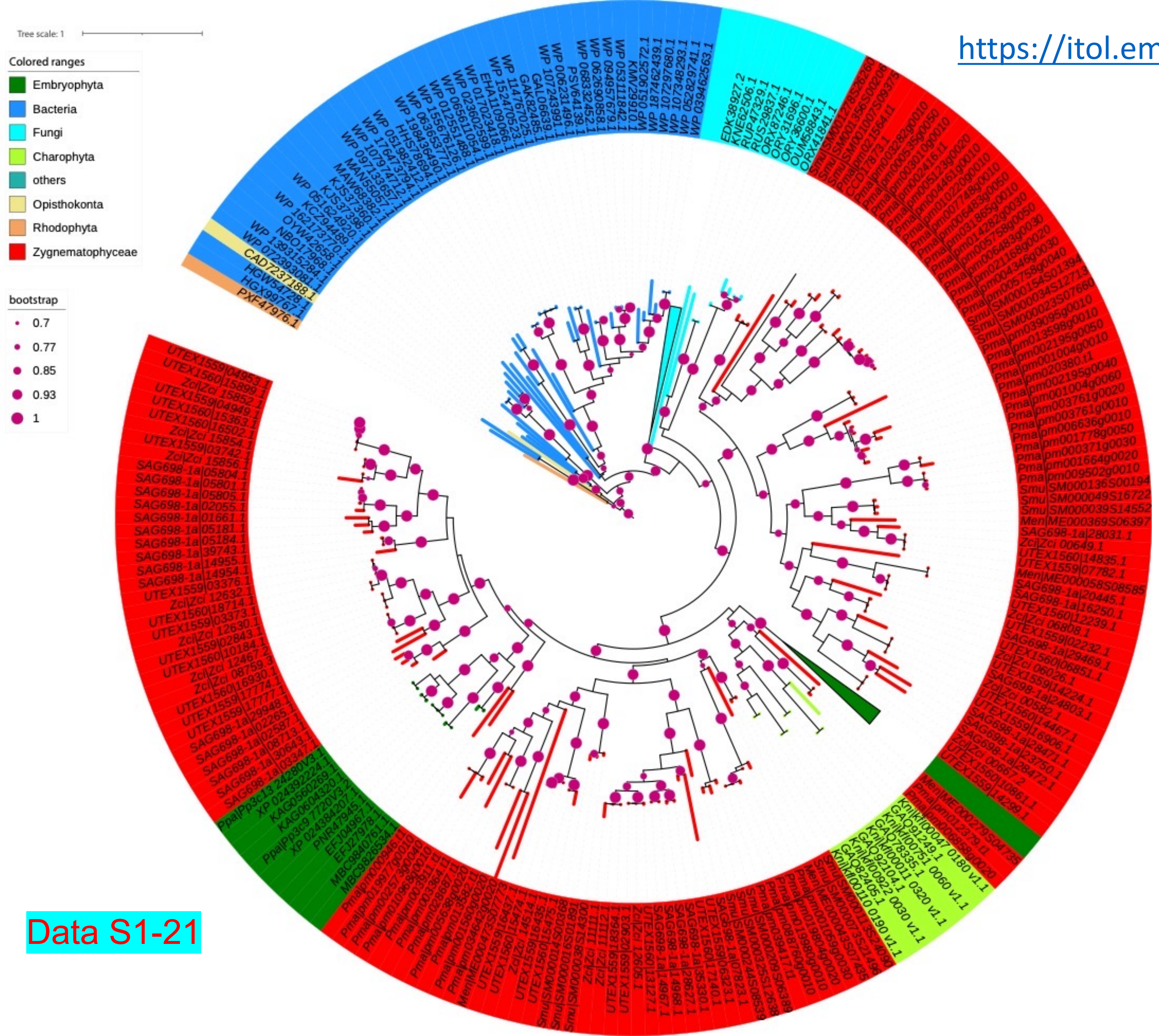

Data S1-21

Putative HGT of XTH between fungi and streptophyte algae (Shinohara and Nishitani, 2021).

XTH genes are expanded in Zygnemophyceae (1b has 16 genes)

Naoki Shinohara, Kazuhiko Nishitani, Cryogenian Origin and Subsequent Diversification of the Plant Cell-Wall Enzyme XTH Family, Plant and Cell Physiology, Volume 62, Issue 12, December 2021, Pages 1874–1889, https://doi.org/10.1093/pcp/pcab093

Data S1-22

GH95: might have been gained via HGT from bacteria

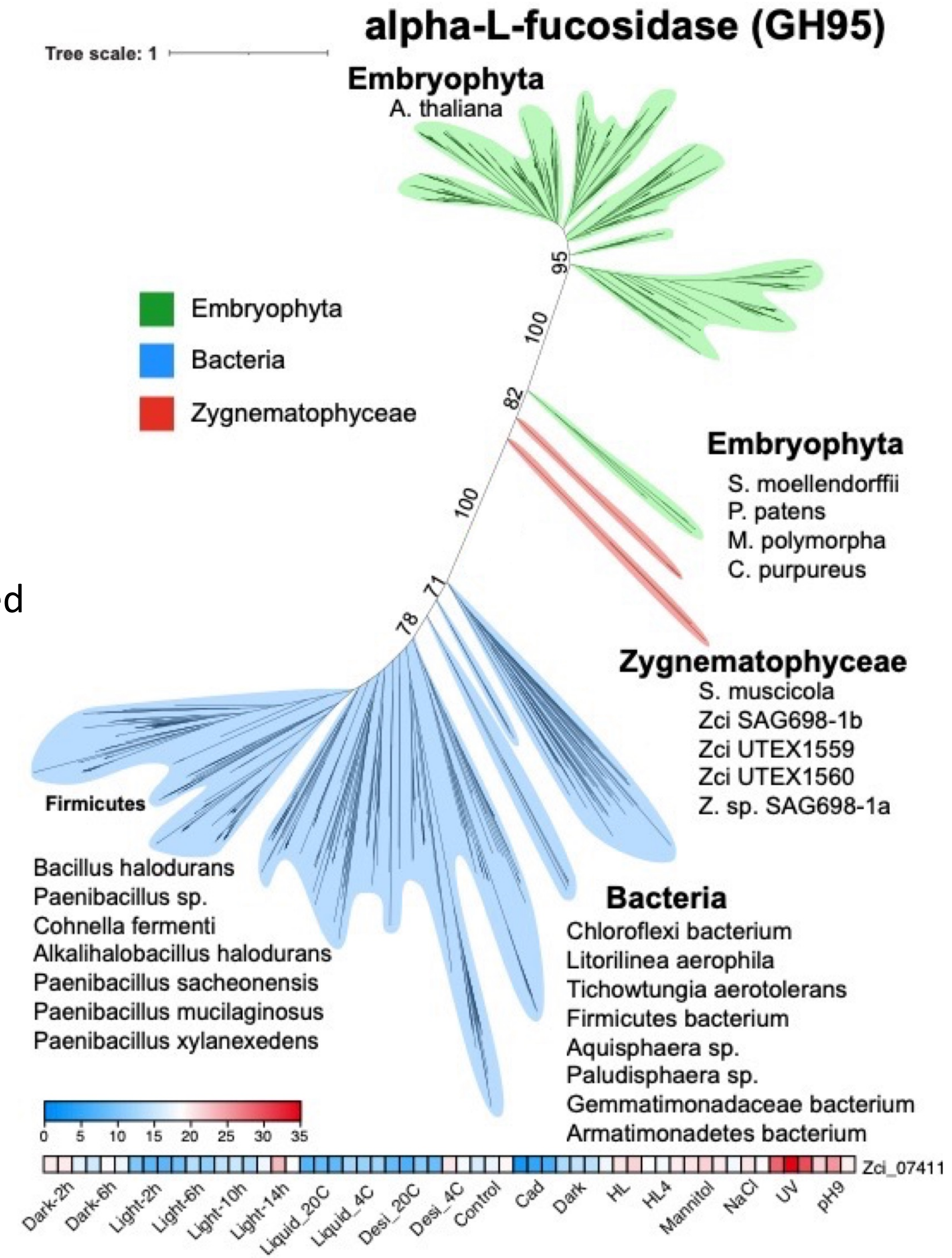

Data S1-23

BGAL8 (AT3G28470)

Kotake T, Dina S, Konishi T, Kaneko S, Igarashi K, Samejima M, Watanabe Y, Kimura K, Tsumuraya Y. Molecular cloning of a {beta}-galactosidase from radish that specifically hydrolyzes {beta}-(1->3)- and {beta}-(1->6)-galactosyl residues of Arabinogalactan protein. Plant Physiol. 2005 Jul;138(3):1563-76. doi: 10.1104/pp.105.062562.

Sampedro et al. AtBGAL10 is the main xyloglucan β-galactosidase in Arabidopsis, and its absence results in unusual xyloglucan subunits and growth defects. Plant Physiol. 2012 Mar;158(3):1146-57. doi: 10.1104/pp.111.192195.

https://itol.embl.de/tree/20812724022273791665197706
https://itol.embl.de/tree/13423816417179911667574537

AtBGAL10 (AT5g63810)

Ishimaru M, Smith DL, Mort AJ, Gross KC. Enzymatic activity and substrate specificity of recombinant tomato beta-galactosidases 4 and 5. Planta. 2009 Jan;229(2):447-56. doi: 10.1007/s00425-008-0842-x.

Eda M, Ishimaru M, Tada T, Sakamoto T, Kotake T, Tsumuraya Y, Mort AJ, Gross KC. Enzymatic activity and substrate specificity of the recombinant tomato β-galactosidase 1. J Plant Physiol. 2014 Oct 15;171(16):1454-60. doi: 10.1016/j.jplph.2014.06.010.

BGAL12 (AT4G26140)

GH35

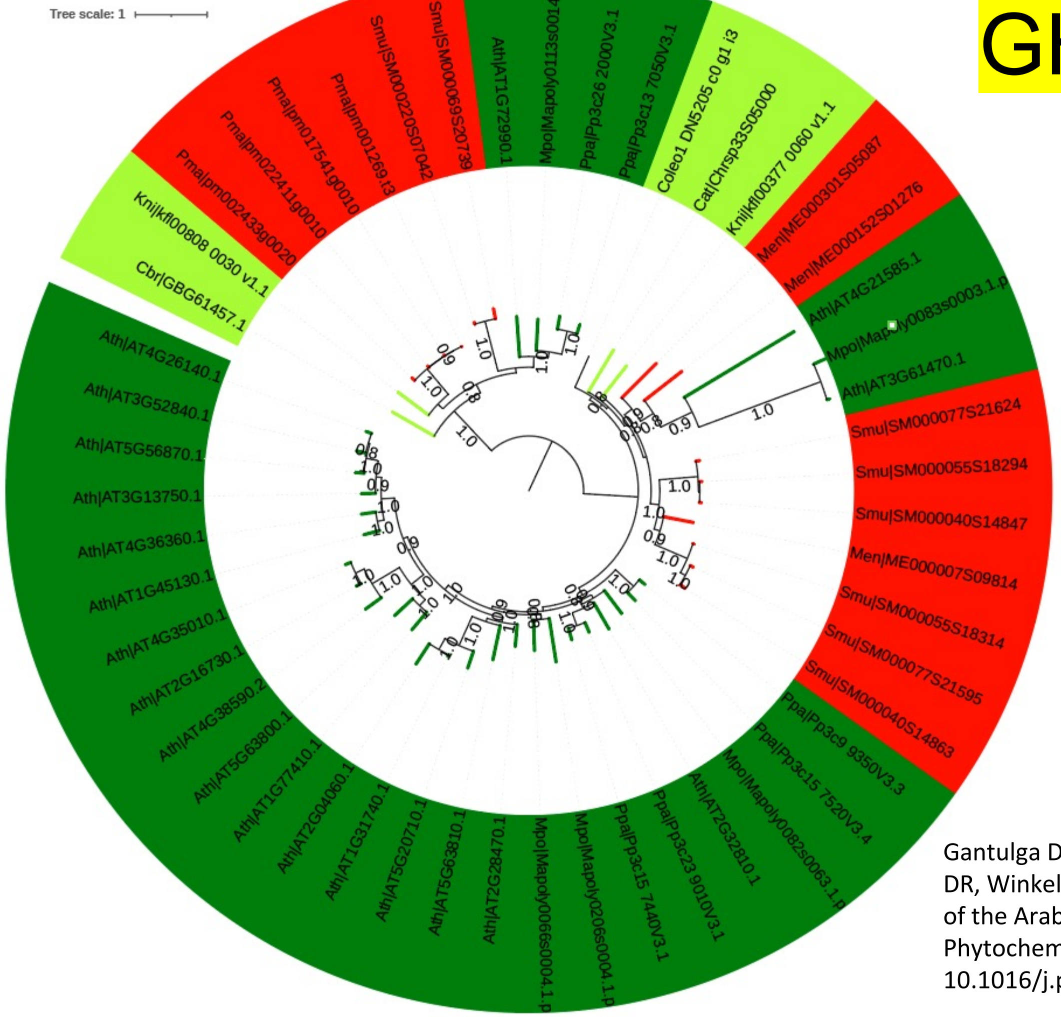

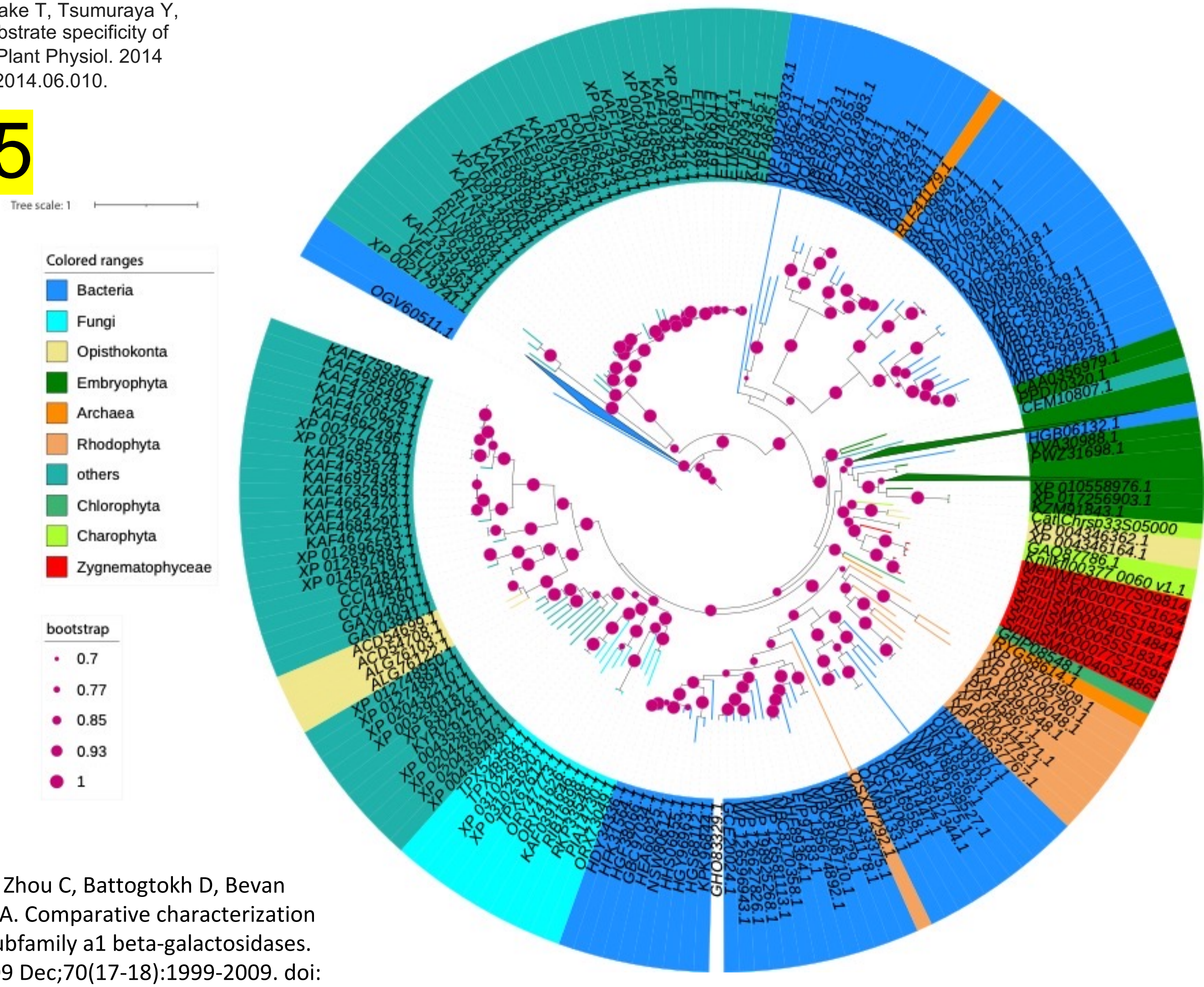

Gantulga D, Ahn YO, Zhou C, Battogtokh D, Bevan DR, Winkel BS, Esen A. Comparative characterization of the Arabidopsis subfamily a1 beta-galactosidases. Phytochemistry. 2009 Dec;70(17-18):1999-2009. doi: 10.1016/j.phytochem.2009.08.008.

Data S1-24

GH31

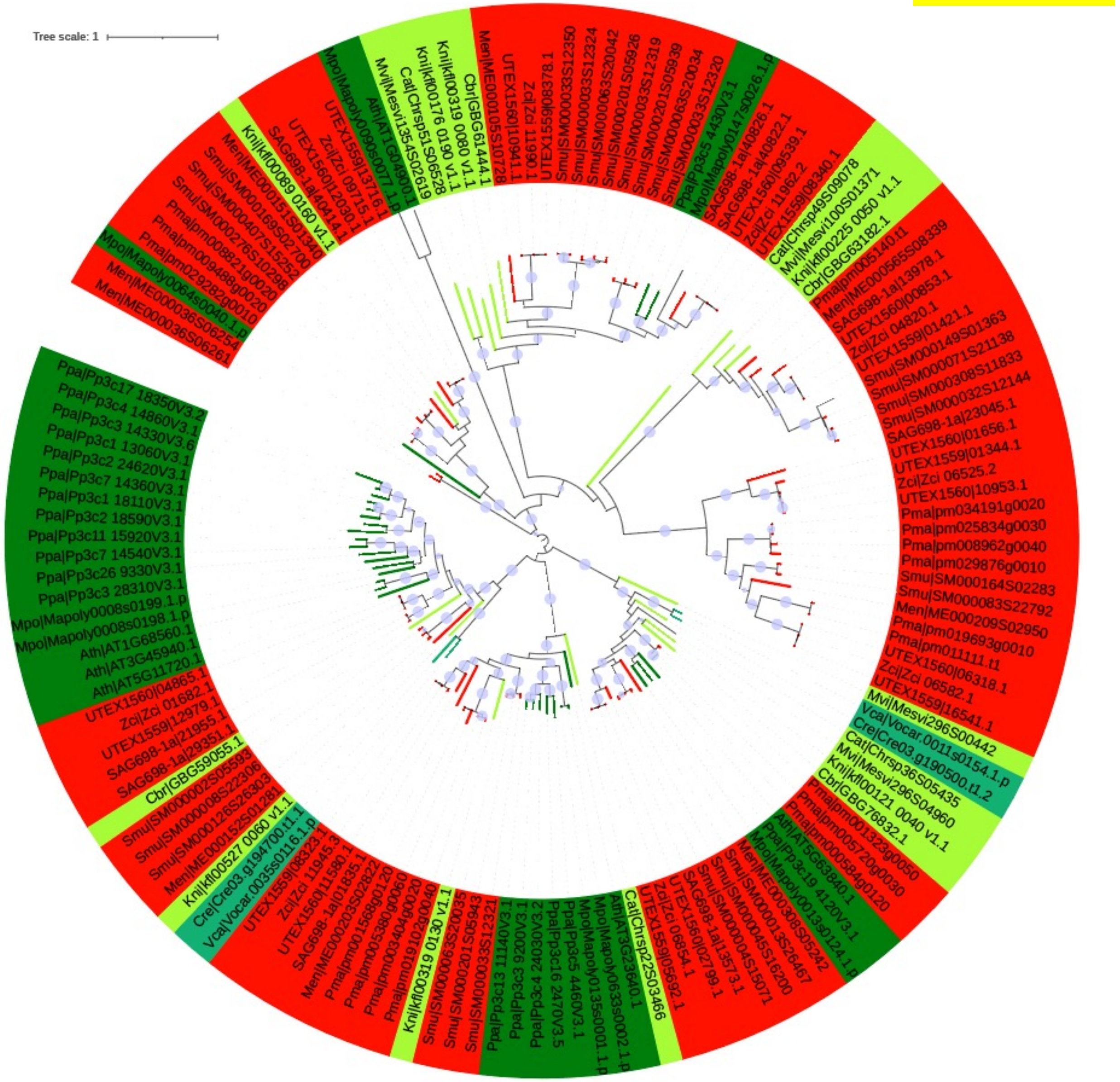

(AXY3)AT1G68560

Javier Sampedro , Brenda Pardo, Cristina Gianzo, Esteban Guitián, Gloria Revilla, Ignacio Zarra. Lack of α-xylosidase activity in Arabidopsis alters xyloglucan composition and results in growth defects
Plant Physiol . 2010 Nov;154(3):1105-15. doi: 10.1104/pp.110.163212. Epub 2010 Aug 26.
PMID: 20801759 PMCID: PMC2971592 DOI: 10.1104/pp.110.163212

Data S1-25

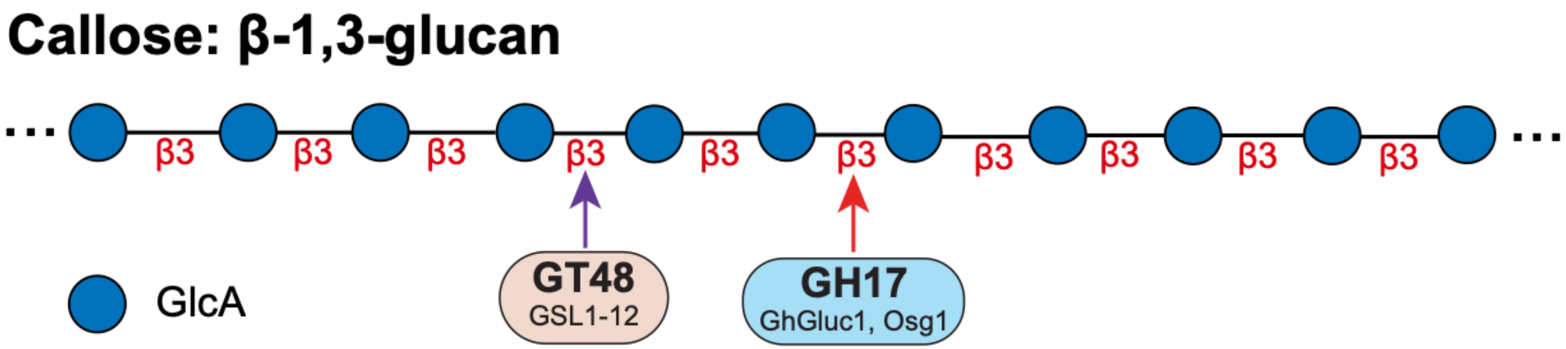

Scherp, P., Grotha, R., and Kutschera, U. (2001). Occurrence and phylogenetic significance of cytokinesis-related callose in green algae, bryophytes, ferns and seed plants. Plant Cell Reports *20*, 143-149.

Davis, D.J., Wang, M., Sorensen, I., Rose, J.K.C., Domozych, D.S., and Drakakaki, G. (2020). Callose deposition is essential for the completion of cytokinesis in the unicellular alga Penium margaritaceum. J Cell Sci *133*.

Data S1-26

GT48

1,3-β-glucan synthase

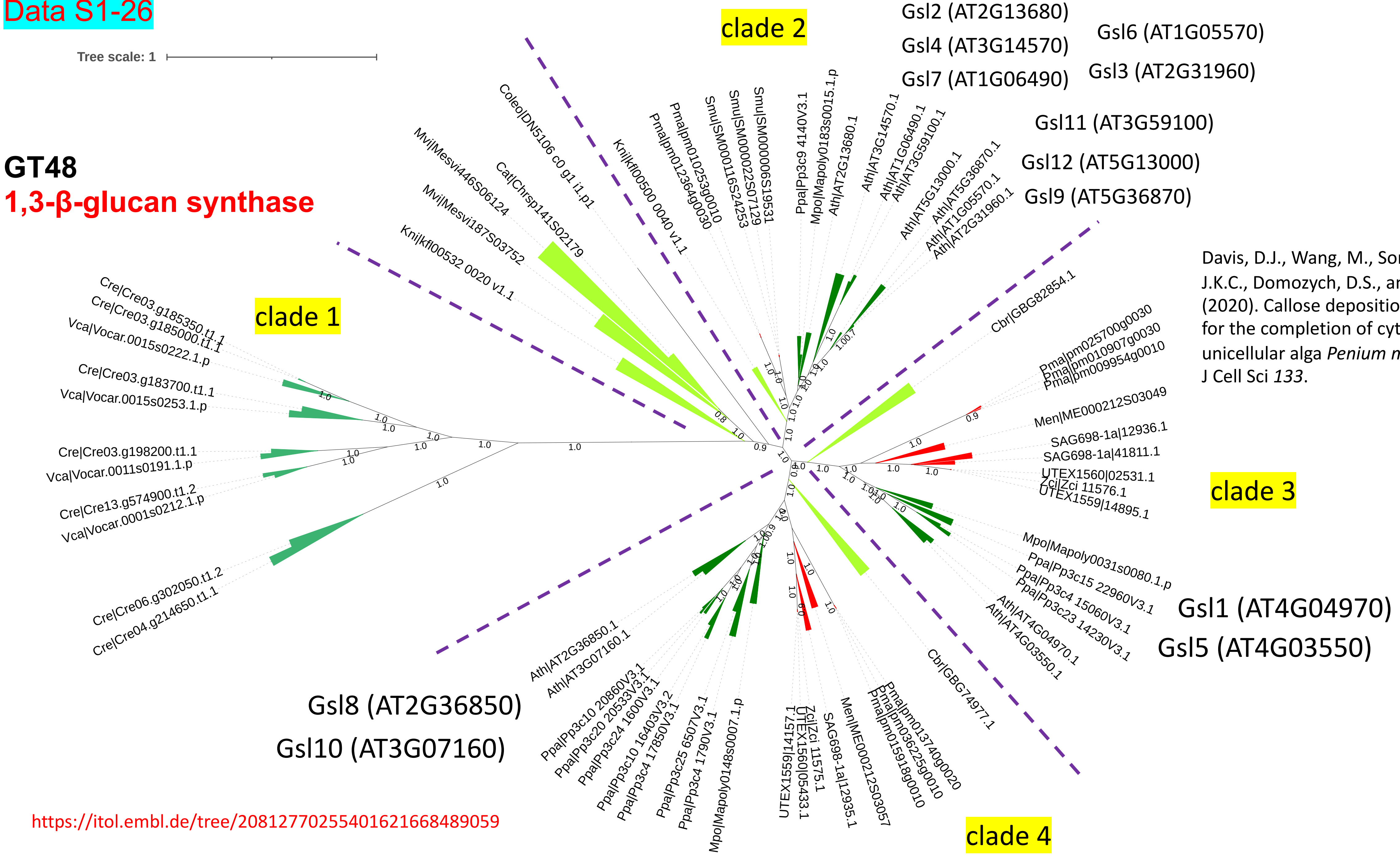

Davis, D.J., Wang, M., Sorensen, I., Rose, J.K.C., Domozych, D.S., and Drakakaki, G. (2020). Callose deposition is essential for the completion of cytokinesis in the unicellular alga *Penium margaritaceum*. J Cell Sci *133*.

https://itol.embl.de/tree/20812770255401621668489059

Data S1-27

GT48 nr hits

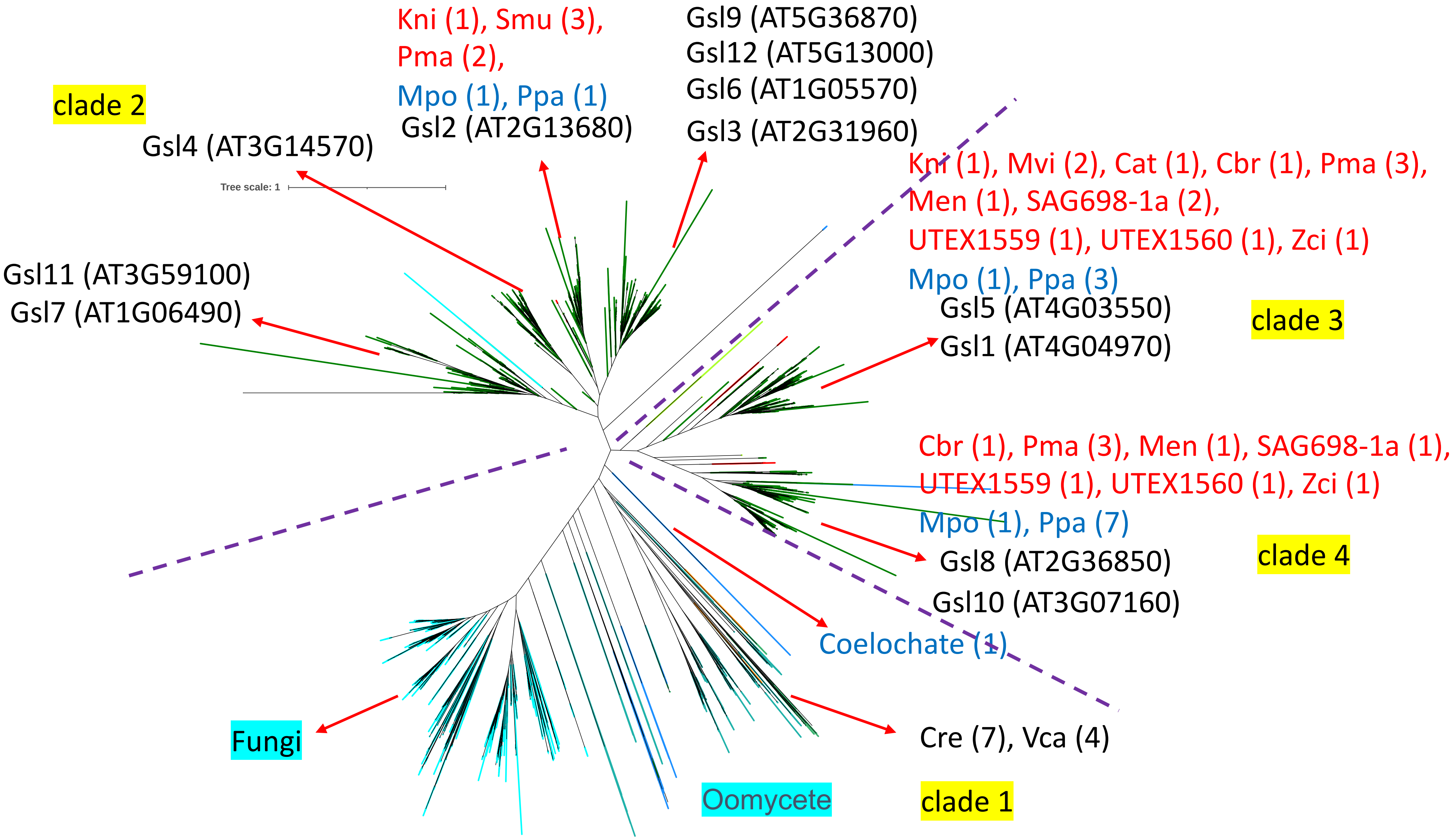

https://itol.embl.de/tree/20812793105136771668526914

Data S1-28

GH17

gamma

BG2
(AT3G57260)

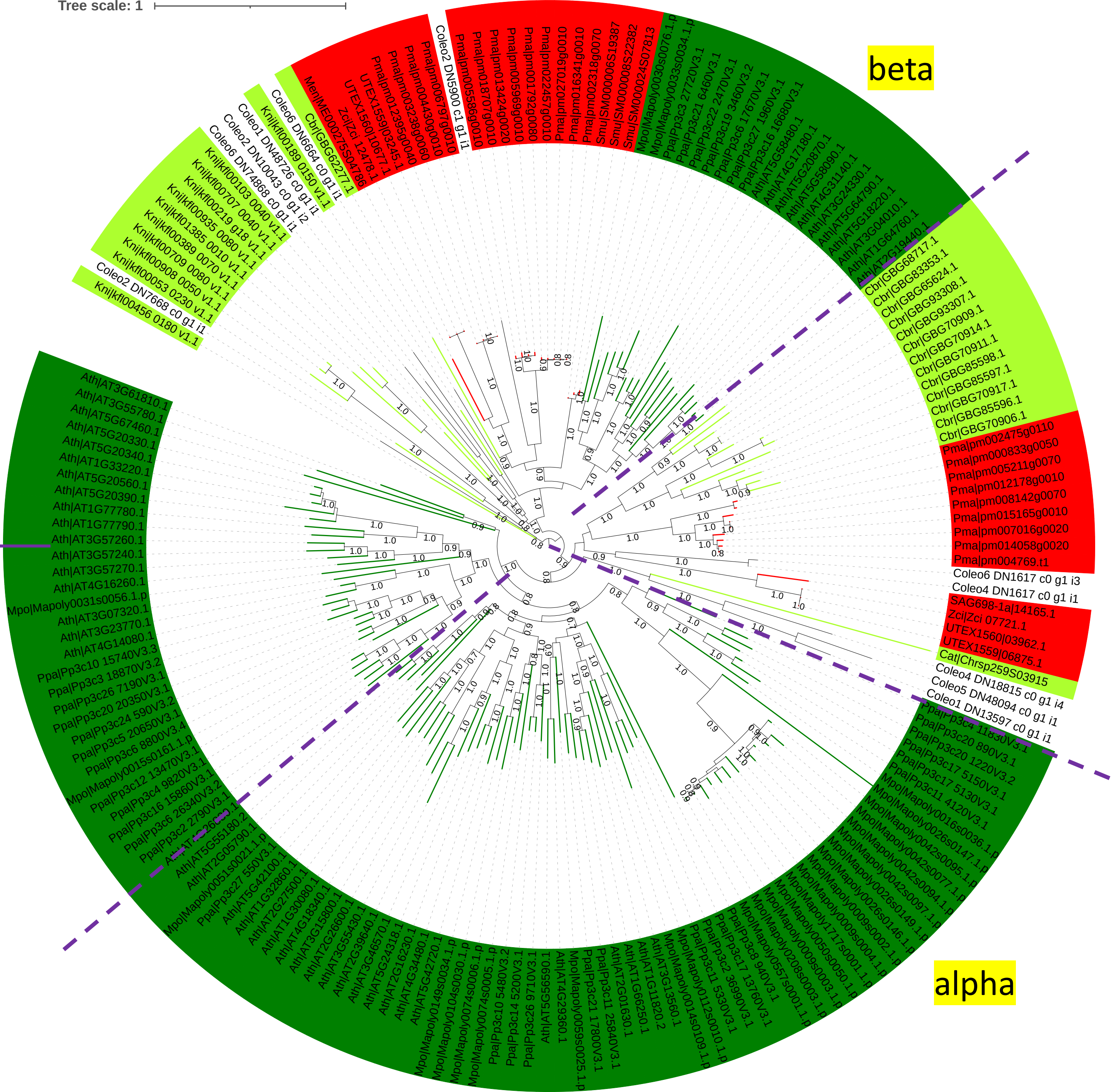

PMID: 24904609

class	Cre	Vca	Mvi	Cat	Kni	Cbr	Smu	Pma	Men	1a	UTEX1559	UTEX1560	1b	Mpo	Ppa	Ath
GH5_14	0	0	0	0	0	0	11	18	14	11	8	9	9	3	2	0

Data S1-29

exo-b-1,3-glucanase (EC 3.2.1.58)

https://itol.embl.de/tree/13423816417184651661223495

AT3G26130: e-value is low 9.1e-06

GH5_14 hmm

Zhou Y, Zeng L, Gui J, Liao Y, Li J, Tang J, Meng Q, Dong F, Yang Z. Functional characterizations of β-glucosidases involved in aroma compound formation in tea (Camellia sinensis). Food Res Int. 2017 Jun;96:206-214. doi: 10.1016/j.foodres.2017.03.049.

Opassiri R, Pomthong B, Akiyama T, Nakphaichit M, Onkoksoong T, Ketudat Cairns M, Ketudat Cairns JR. A stress-induced rice (Oryza sativa L.) beta-glucosidase represents a new subfamily of glycosyl hydrolase family 5 containing a fascin-like domain. Biochem J. 2007 Dec 1;408(2):241-9. doi: 10.1042/BJ20070734.

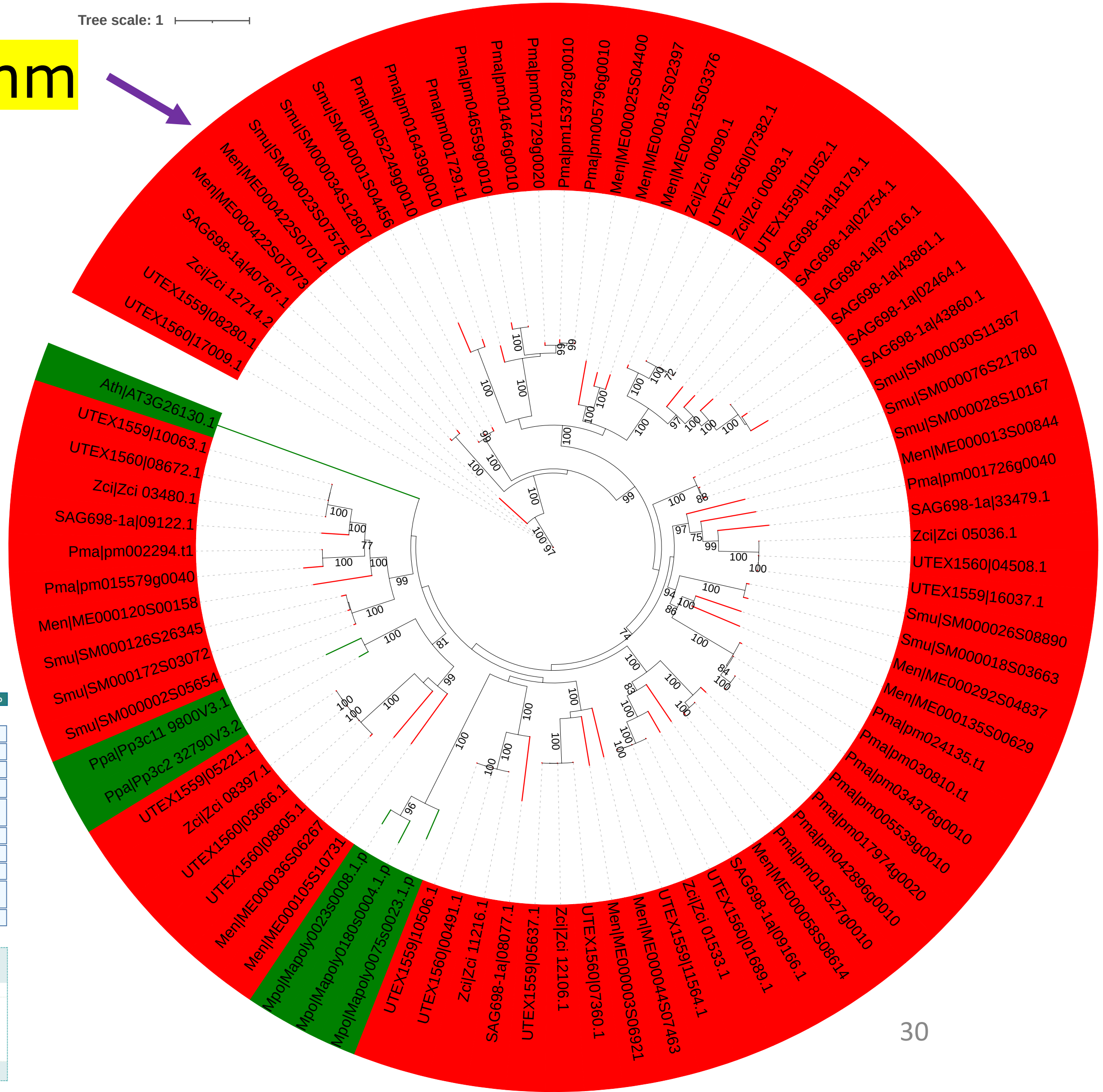

Glycoside Hydrolase Family 5 / Subf 14

GH5 Go GH5_14 Go Family Go

Activities in Sub Family	b-glucosidase (EC 3.2.1.21);exo-b-1,3-glucanase (EC 3.2.1.58)
Mechanism	Retaining
Clan	GH-A
3D Structure Status	(β / α) $_8$ barrel
Catalytic Nucleophile/Base	Glu (experimental)
Catalytic Proton Donor	Glu (experimental)
Note	Once known as cellulase family A; many members have been assigned to subfamilies as described by Aspeborg et al. (2012) BMC Evol Biol. 12(1):186 (PMID: 22992189).
External resources	CAZypedia; HOMSTRAD; PROSITE;
Commercial Enzyme Provider(s)	MEGAZYME; NZYTech; PROZOMIX;
Statistics	GenBank accession (103); Uniprot accession (3);

Summary | Download GH5_14 (83) | Taxonomic display | Characterized (2)

Eukaryota

Protein Name	EC#	Reference	Organism	GenBank	Uniprot	PDB/3D	Subf
β-glucosidase 1 GH5 family (GH5BG1;CsGH5BG1)	3.2.1.21	pubmed	Camellia sinensis	ARU79086.1	A0A1Y0K2D4		14
exo-β-1,3-glucosidase (GH5BG;Os10g0370500)	3.2.1.58	pubmed	Oryza sativa Japonica Group	AAM08614.1<br>AAM08821.1<br>AAP53379.1<br>BAF26372.1<br>BAG89316.1<br>BAT10560.1	Q0IY43<br>Q8RU06		14

Top

Data S1-30

GH5_14 nr
e-15

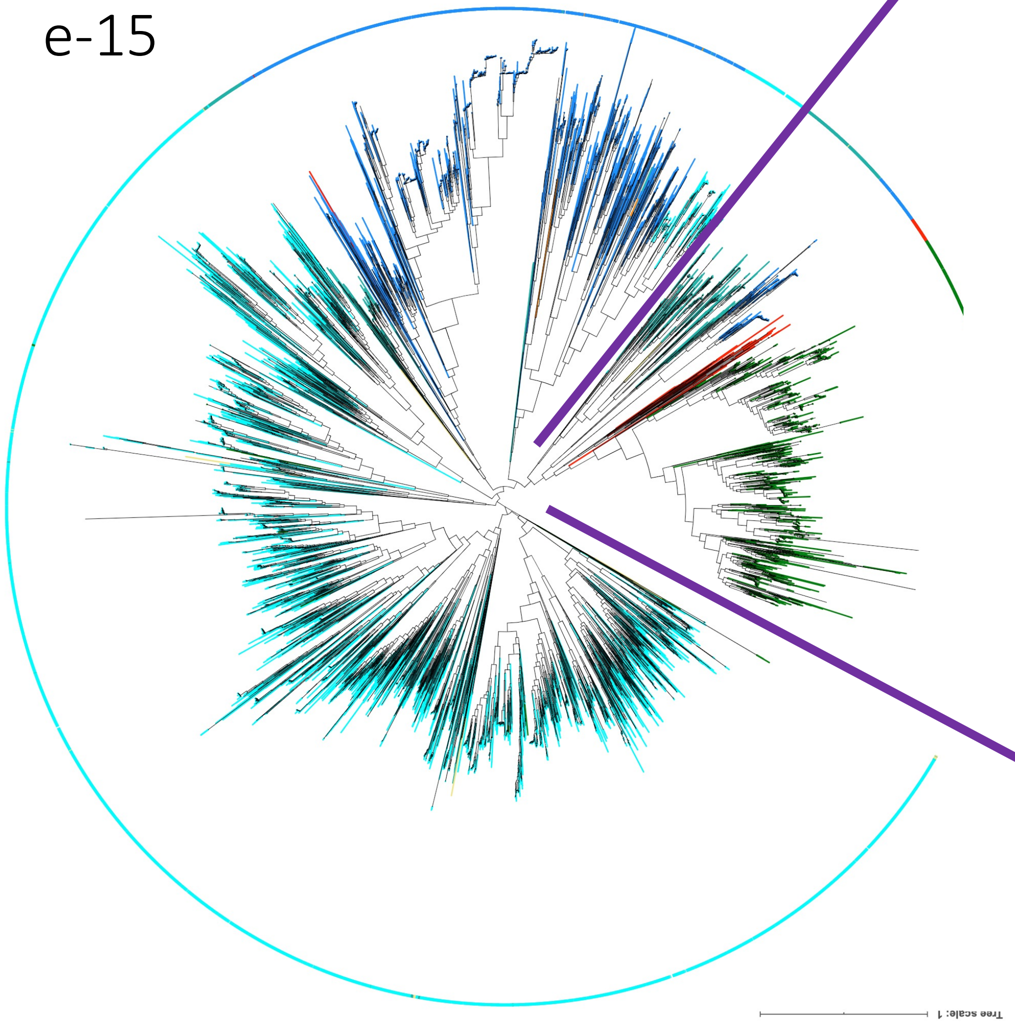

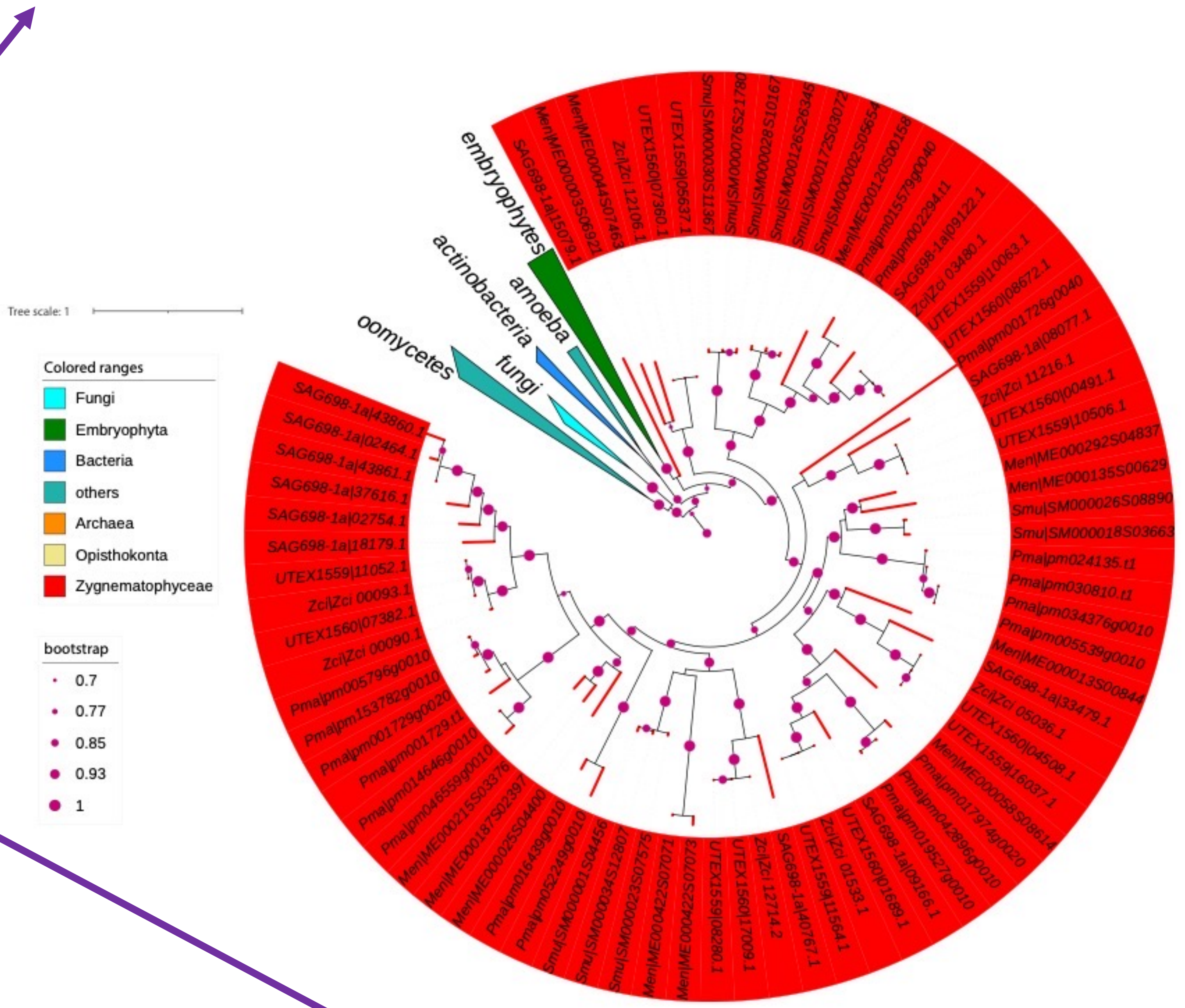

Possible gain via HGT from bacteria

Data S1-31

The combination of Fascin domain and GH5_14 is first present in Zygnematophyceae

Conserved domains on [gi|19920182|gb|AAM08614|]

Putative Glucan 1,3-beta-glucosidase precursor [Oryza sativa Japonica Group]

View Standard Results

Graphical summary Zoom to residue level show extra options »

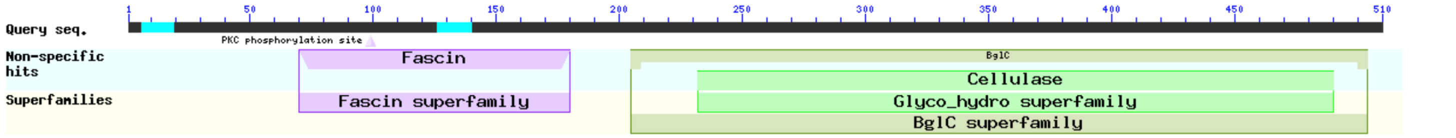

Search for similar domain architectures | Refine search

List of domain hits

Name	Accession	Description	Interval	E-value
[+] BglC	COG2730	Aryl-phospho-beta-D-glucosidase BglC, GH1 family [Carbohydrate transport and metabolism];	205-504	2.41e-29
[+] Cellulase	pfam00150	Cellulase (glycosyl hydrolase family 5);	232-490	8.40e-14
[-] Fascin	cd00257	Fascin-like domain; members include actin-bundling/crosslinking proteins fascin, ...	70-180	3.74e-03

Fascin-like domain; members include actin-bundling/crosslinking proteins fascin, histoactophilin and singed; identified in sea urchin, Drosophila, Xenopus, rodents, and humans; The fascin-like domain adopts a beta-trefoil topology and contains an internal threefold repeat; the fascin subgroup contains four copies of the domain; Structurally similar to fibroblast growth factor (FGF)

Pssm-ID: 238160 **Cd Length:** 119 **Bit Score:** 37.25 **E-value:** 3.74e-03

```
                        10        20        30        40        50        60        70        80
                ....*....|....*....|....*....|....*....|....*....|....*....|....*....|....*....|
gi 19920182  70 TQLQFKSVTQNmYLCAEQGGGTiLVANRTSASGWETFKLWRIDEDTFDLRVFDNLFVTVAGDGVTVVATvaSPGPGEAFQ 149
Cdd:cd00257   2 PQVVLRSVNGR-YLSAEAGGDK-VDANRDSLKGDETFTLEFDNTGKYALRSHDGKYLSADSDGGVQLEG--HPNADCRFT 77

                        90       100       110
                ....*....|....*....|....*....|.
gi 19920182 150 IVRNGDkTRARIRAPNGMFLQAKTSDSVTAD 180
Cdd:cd00257  78 LEFHGD-GKWALRAENGRYLGGDGSGTLKAS 107
```

rice GH5BG (Fascin domain)

Conserved domains on [lcl|Query_8185]

Local query sequence

View Standard Results

Graphical summary Zoom to residue level show extra options »

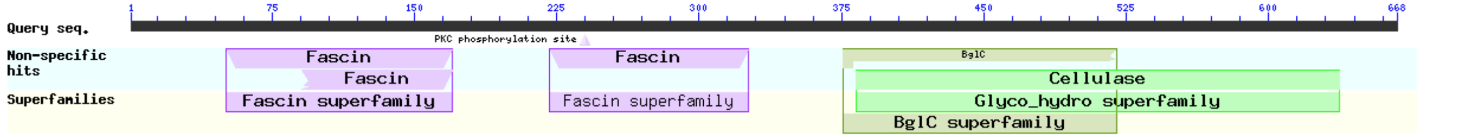

Search for similar domain architectures | Refine search

List of domain hits

Name	Accession	Description	Interval	E-value
[+] BglC	COG2730	Aryl-phospho-beta-D-glucosidase BglC, GH1 family [Carbohydrate transport and metabolism];	376-520	7.10e-17
[+] Cellulase	pfam00150	Cellulase (glycosyl hydrolase family 5);	383-637	2.23e-09
[+] Fascin	cd00257	Fascin-like domain; members include actin-bundling/crosslinking proteins fascin, ...	221-326	2.79e-09
[+] Fascin	pfam06268	Fascin domain; This family consists of several eukaryotic fascin or singed proteins. The ...	51-170	1.19e-06
[-] Fascin	cd00257	Fascin-like domain; members include actin-bundling/crosslinking proteins fascin, ...	91-170	4.00e-06

Fascin-like domain; members include actin-bundling/crosslinking proteins fascin, histoactophilin and singed; identified in sea urchin, Drosophila, Xenopus, rodents, and humans; The fascin-like domain adopts a beta-trefoil topology and contains an internal threefold repeat; the fascin subgroup contains four copies of the domain; Structurally similar to fibroblast growth factor (FGF)

Pssm-ID: 238160 **Cd Length:** 119 **Bit Score:** 46.11 **E-value:** 4.00e-06

```
                        10        20        30        40        50        60        70        80
                ....*....|....*....|....*....|....*....|....*....|....*....|....*....|....*....|
Query_8185   91 VAFRGPNGKLLAAHADATVTASGEASgtdipTECMFRVEKKPNKKFTIWSVsNSYYLRAWDNGTLRLHMKAPTSWELFSI 170
Cdd:cd00257  46 YALRSHDGKYLSADSDGGVQLEGHPN-----ADCRFTLEFHGDGKWALRAE-NGRYLGGDGSGTLKASSETVGPDELFEL 119
```

Zci|Zci_03480.1 (Fascin domain)

Data S1-32

Xylan / AX / GX / GAX

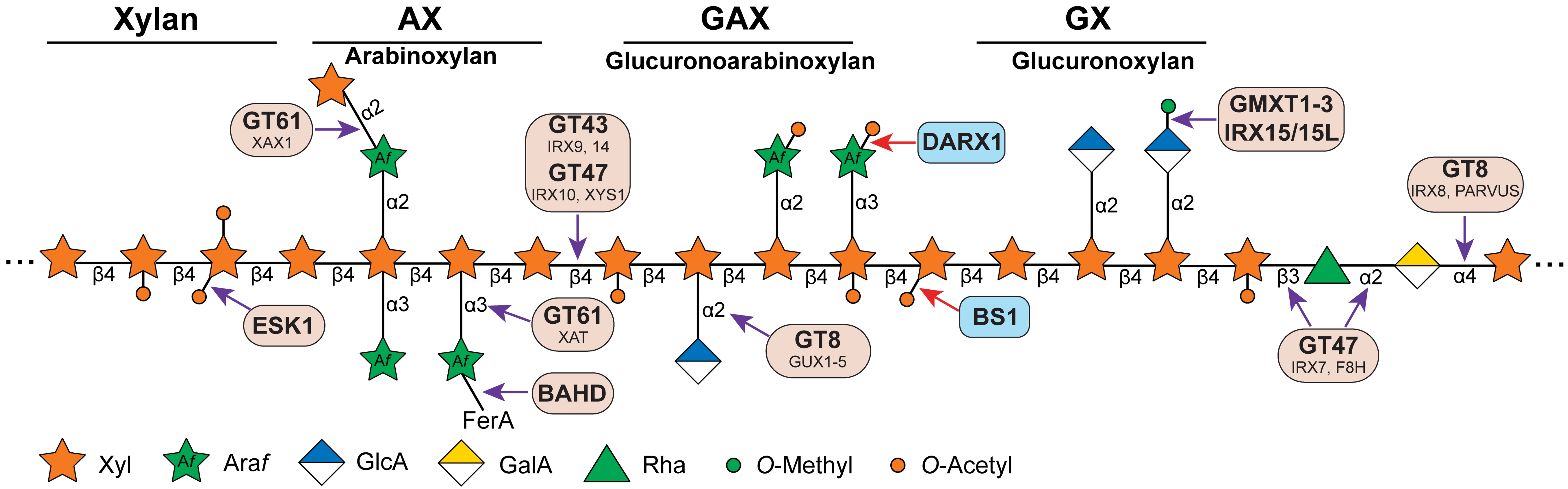

GT47

Data S1-33

Madson M, Dunand C, Li X, Verma R, Vanzin GF, Caplan J, Shoue DA, Carpita NC, Reiter WD. The MUR3 gene of Arabidopsis encodes a xyloglucan galactosyltransferase that is evolutionarily related to animal exostosins. Plant Cell. 2003 Jul;15(7):1662-70. doi: 10.1105/tpc.009837.

XyG

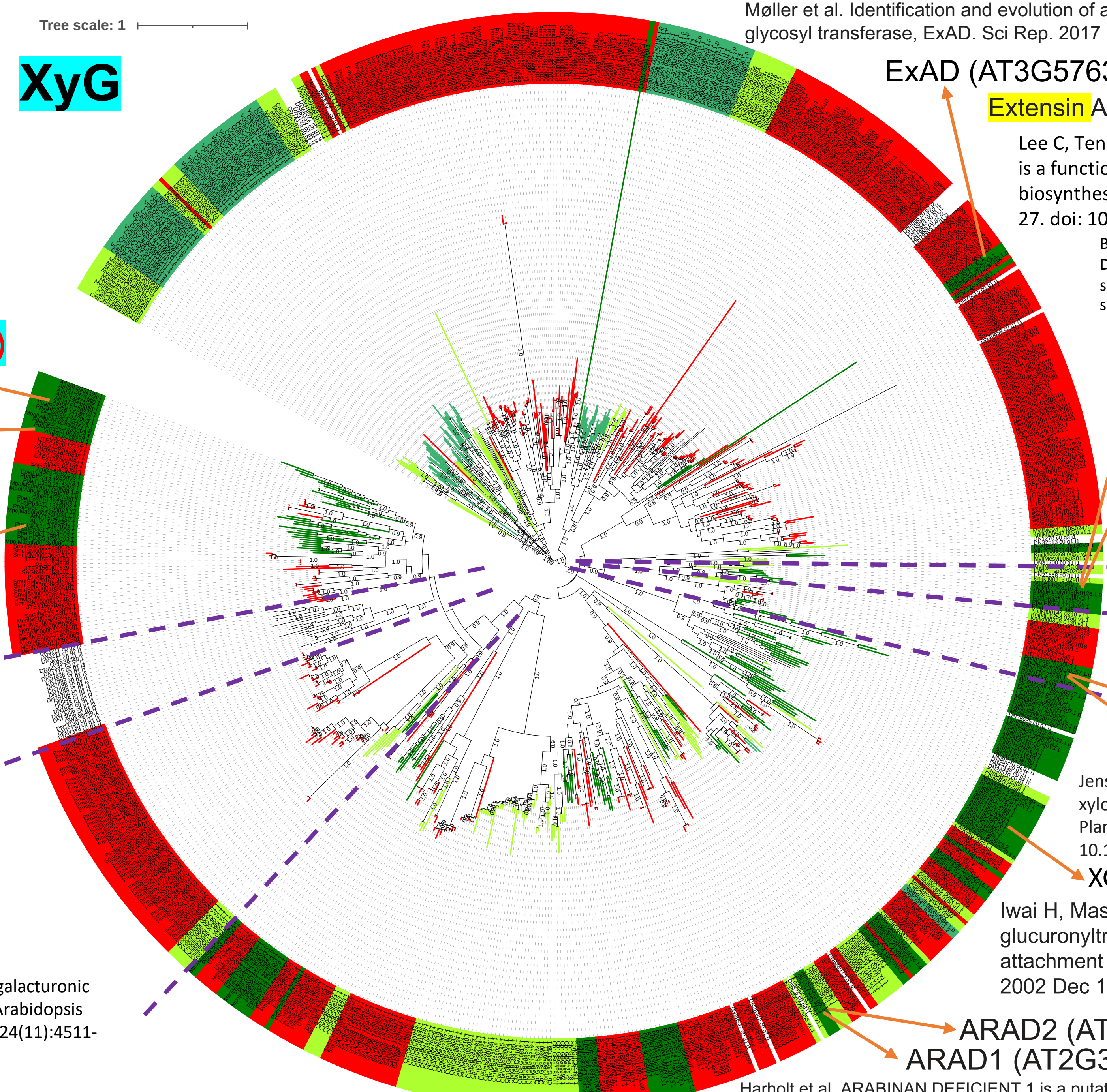

MUR3 (AT2G20370)

XLT2 (AT5G62220)

XUT1 (AT1G63450)

Coleochaete scutate
Coleochaete orbicularis

Jensen JK, Schultink A, Keegstra K, Wilkerson CG, Pauly M. RNA-Seq analysis of developing nasturtium seeds (Tropaeolum majus): identification and characterization of an additional galactosyltransferase involved in xyloglucan biosynthesis. Mol Plant. 2012 Sep;5(5):984-92. doi: 10.1093/mp/sss032.

Peña MJ, Kong Y, York WS, O'Neill MA. A galacturonic acid-containing xyloglucan is involved in Arabidopsis root hair tip growth. Plant Cell. 2012 Nov;24(11):4511-24. doi: 10.1105/tpc.112.103390.

https://itol.embl.de/tree/2081277025518872166313428 3

Møller et al. Identification and evolution of a plant cell wall specific glycoprotein glycosyl transferase, ExAD. Sci Rep. 2017 Mar 30;7:45341. doi: 10.1038/srep45341

ExAD (AT3G57630)

Extensin Arabinose Deficient transferase

Lee C, Teng Q, Huang W, Zhong R, Ye ZH. The F8H glycosyltransferase is a functional paralog of FRA8 involved in glucuronoxylan biosynthesis in Arabidopsis. Plant Cell Physiol. 2009 Apr;50(4):812-27. doi: 10.1093/pcp/pcp025. Epub 2009 Feb 18. PMID: 19224953.

Brown, D.M., Goubet, F., Wong, V.W., Goodacre, R., Stephens, E., Dupree, P., and Turner, S.R. (2007). Comparison of five xylan synthesis mutants reveals new insight into the mechanisms of xylan synthesis. Plant J. 52: 1154–1168.

F8H (AT5G22940)

IRX7/FRA8 (AT2G28110)

NpGUT1: pectin b-glucuronyltransferase transferring glucuronic acid to RG-II

Urbanowicz BR, Peña MJ, Moniz HA, Moremen KW, York WS. 2014. Two Arabidopsis proteins synthesize acetylated xylan in vitro. Plant Journal 80: 197–206.

Jensen JK, Busse-Wicher M, Poulsen CP, Fangel JU, Smith PJ, Yang JY, Peña MJ, Dinesen MH, Martens HJ, Melkonian M et al. 2018. Identification of an algal xylan synthase indicates that there is functional orthology between algal and plant cell wall biosynthesis. New Phytologist 218: 1049–1060.

XYS1/IRX10-L (AT5G61840)

IRX10 (AT1G27440)

Xylan

Jensen et al. Identification of a xylogalacturonan xylosyltransferase involved in pectin biosynthesis in Arabidopsis. Plant Cell. 2008 May;20(5):1289-302. doi: 10.1105/tpc.107.050906

XGD1 (AT5G33290)

XGA

Iwai H, Masaoka N, Ishii T, Satoh S. A pectin glucuronyltransferase gene is essential for intercellular attachment in the plant meristem. Proc Natl Acad Sci U S A. 2002 Dec 10;99(25):16319-24. doi: 10.1073/pnas.252530499.

ARAD2 (AT5G44930)

ARAD1 (AT2G35100)

RG-I

Harholt et al. ARABINAN DEFICIENT 1 is a putative arabinosyltransferase involved in biosynthesis of pectic arabinan in Arabidopsis. Plant Physiol. 2006 Jan;140(1):49-58. doi: 10.1104/pp.105.072744.

Data S1-34

https://itol.embl.de/tree/208127702551887216631342838

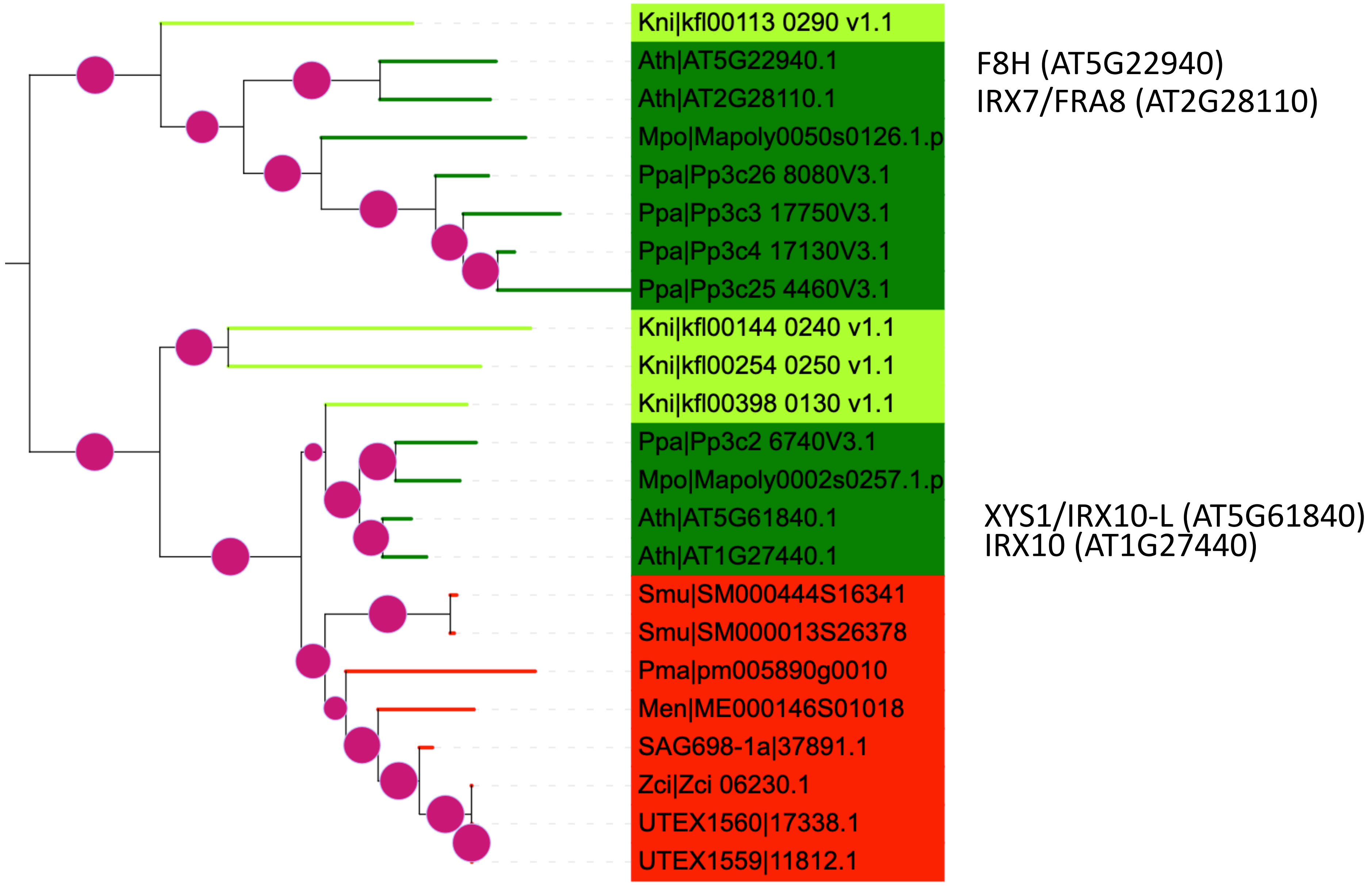

GT47

Data S1-35

https://itol.embl.de/tree/208127240222430161661878342

Expanded GT43 (7 1b genes)

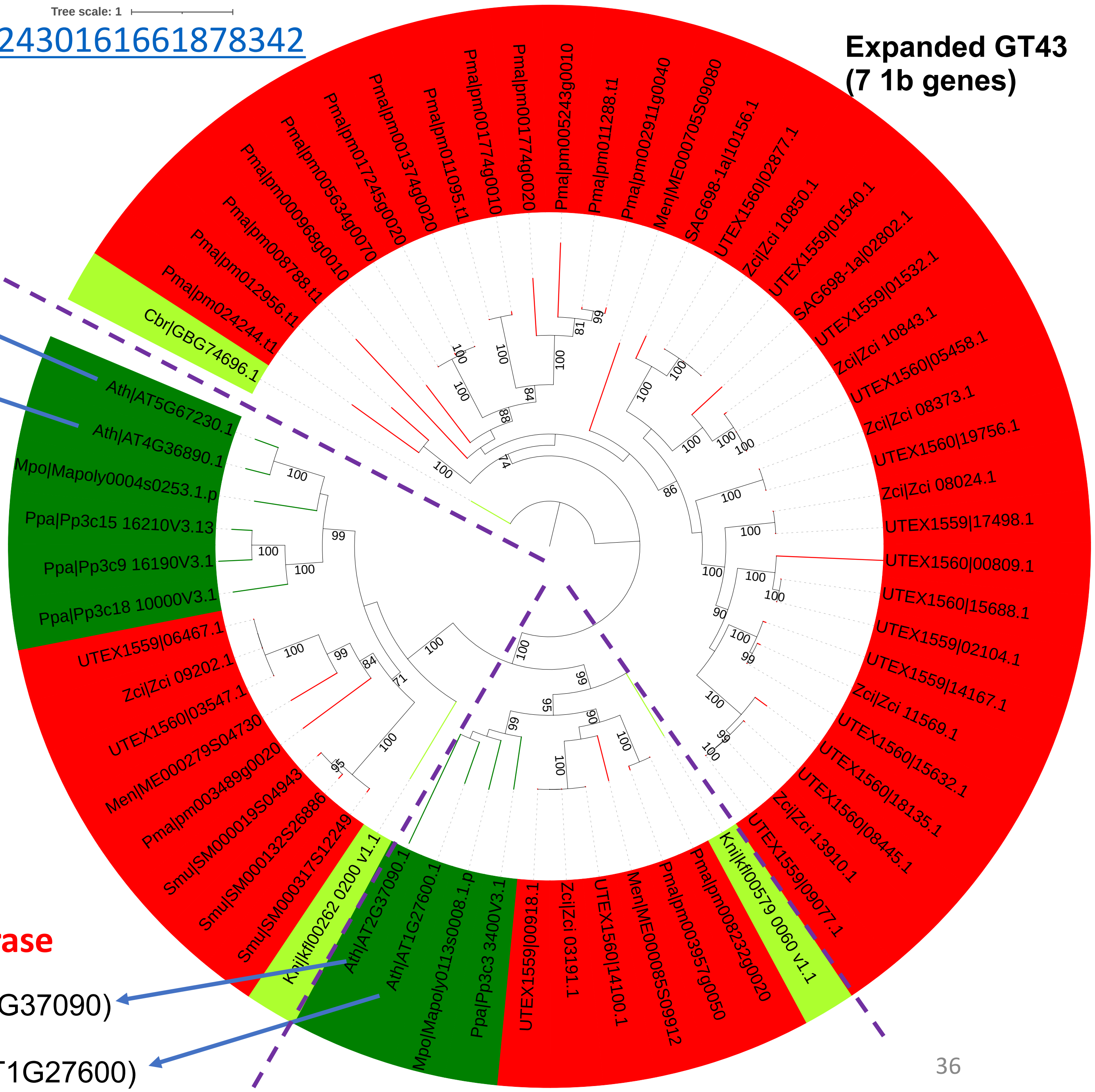

glucuronoxylan glycosyltransferase

IRX14-L (AT5G67230)

IRX14 (AT4G36890)

secondary wall thickening in fibers

Brown, D.M., Goubet, F., Wong, V.W., Goodacre, R., Stephens, E., Dupree, P., and Turner, S.R. (2007). Comparison of five xylan synthesis mutants reveals new insight into the mechanisms of xylan synthesis. Plant J. 52: 1154–1168.

Lee C, Teng Q, Huang W, Zhong R, Ye ZH. The Arabidopsis family GT43 glycosyltransferases form two functionally nonredundant groups essential for the elongation of glucuronoxylan backbone. Plant Physiol. 2010 Jun;153(2):526-41. doi: 10.1104/pp.110.155309.

Ren Y, Hansen SF, Ebert B, Lau J, Scheller HV. Site-directed mutagenesis of IRX9, IRX9L and IRX14 proteins involved in xylan biosynthesis: glycosyltransferase activity is not required for IRX9 function in Arabidopsis. PLoS One. 2014 Aug 13;9(8):e105014. doi: 10.1371/journal.pone.0105014.

GT43

xylan β-1,4-xylosyltransferase

IRX9 (AT4G37090)

IRX9-L (AT1G27600)

Data S1-36

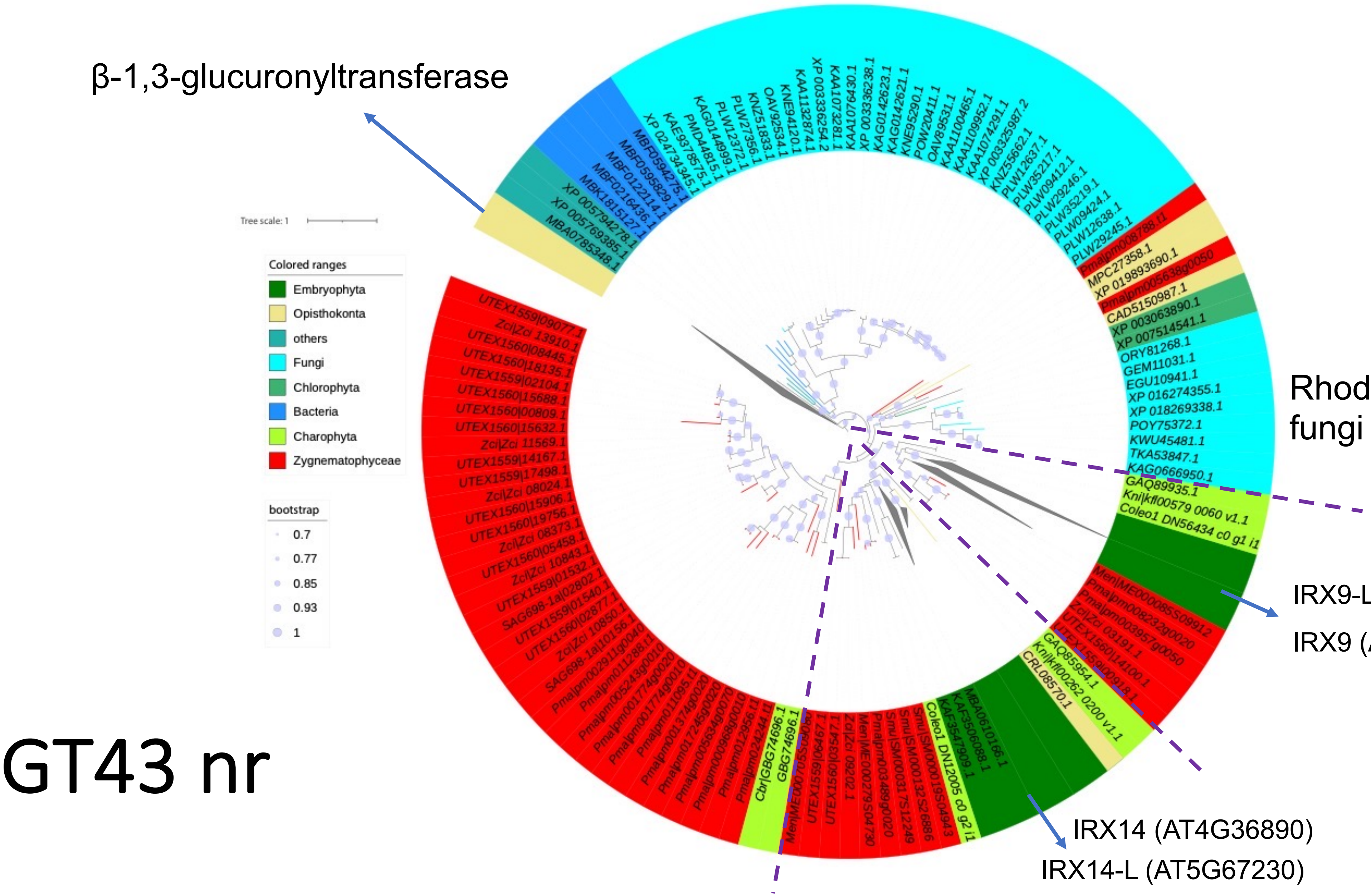

β-1,3-glucuronyltransferase

Rhodotorula fungi

IRX9-L (AT1G27600)

IRX9 (AT4G37090)

IRX14 (AT4G36890)

IRX14-L (AT5G67230)

GT43 nr

HGT with fungi

Data S1-37

GT8 (dbcan) + Coleochaete

Glucuronyltransferases of Xylan (first present in LCA Z+E)

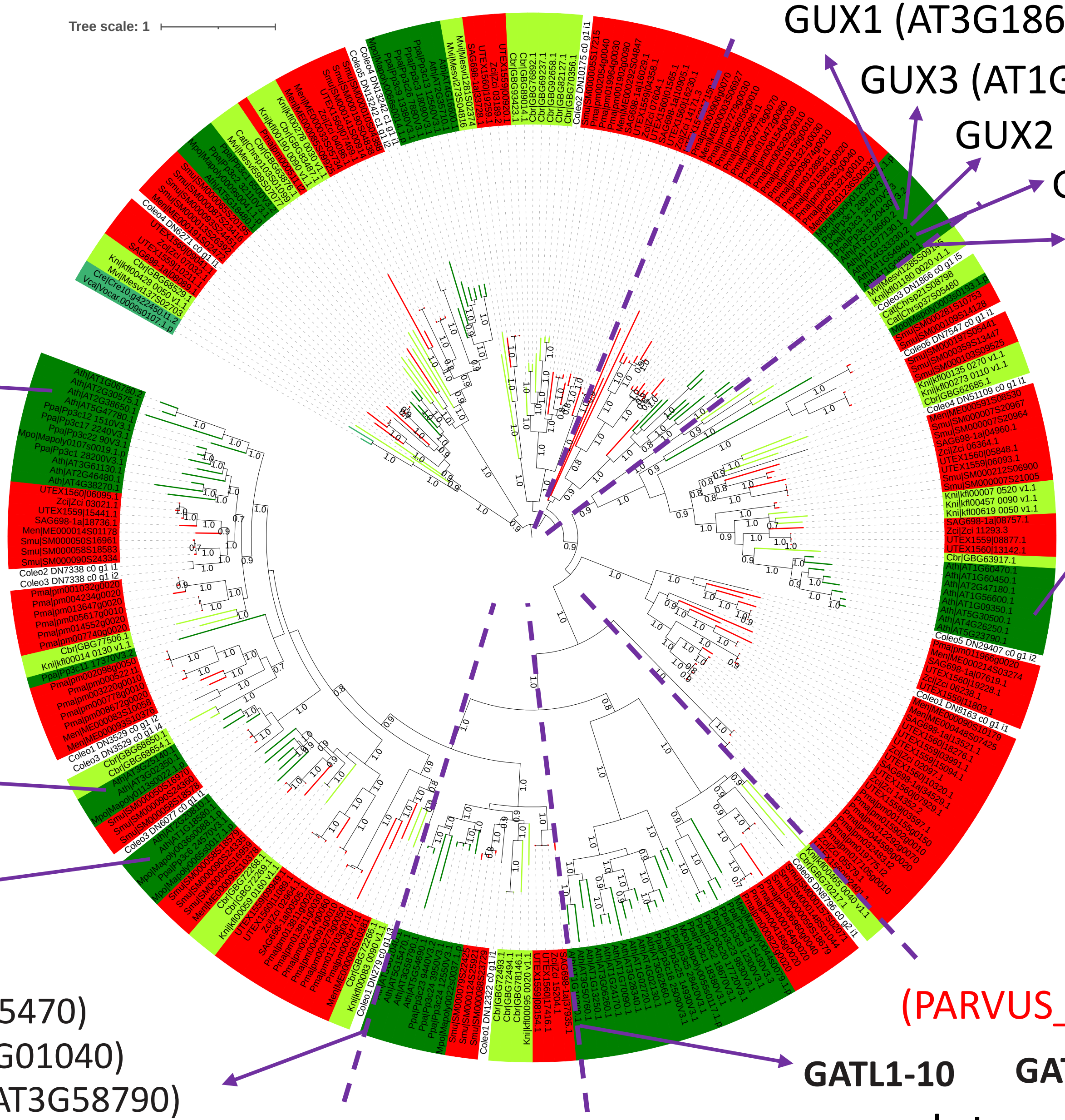

GUX1 (AT3G18660)
GUX3 (AT1G77130)
GUX2 (AT4G33330)
GUX4 (AT1G54940)
GUX5 (AT1G08990)

Rennie EA, Hansen SF, Baidoo EEK, Hadi MZ, Keasling JD, Scheller HV. 2012. Three members of the Arabidopsis glycosyltransferase family 8 are xylan glucuronosyltransferases. Plant Physiology 159: 1408–1417.

GOLS

GAUT6 (AT1G06780)
GAUT5 (AT2G30575)
GAUT7 (AT2G38650)
GAUT4 (AT5G47780)
GAUT1 (AT3G61130)
GAUT2 (AT2G46480)
GAUT3 (AT4G38270)

GAUT8 (AT3G25140)
GAUT9 (AT3G02350)

GAUT10 (AT2G20810)
GAUT11 (AT1G18580)

GAUT14 (AT5G15470)
GAUT13 (AT3G01040)
GAUT15 (AT3G58790)
GAUT12 (AT5G54690) (IRX8: Xylan)

(PARVUS_GATL: Xylan)

GATL1-10 **GATL1** (AT1G19300)

galacturonosyltransferase-like

Data S1-38

https://itol.embl.de/tree/208127240222263521665352415

(PARVUS_GATL: Xylan)

GAUT12 (AT5G54690) (IRX8: Xylan)

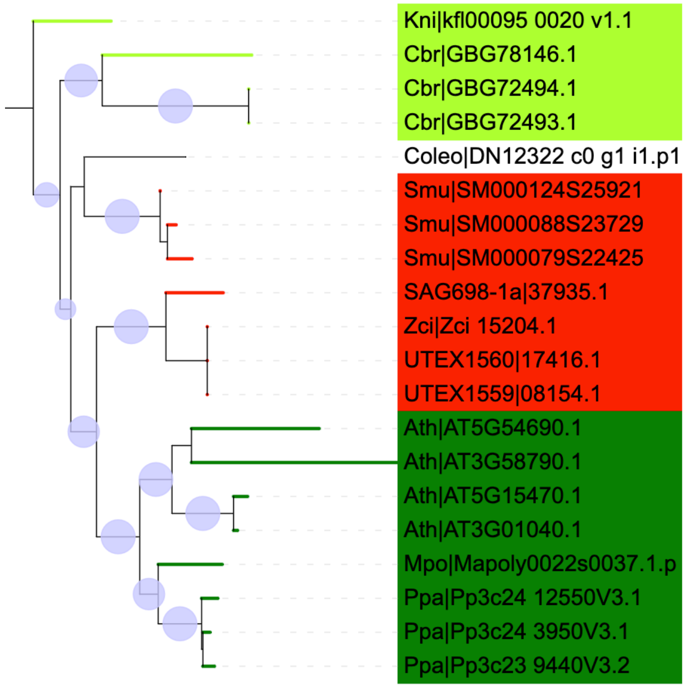

GATL1 (AT1G19300)

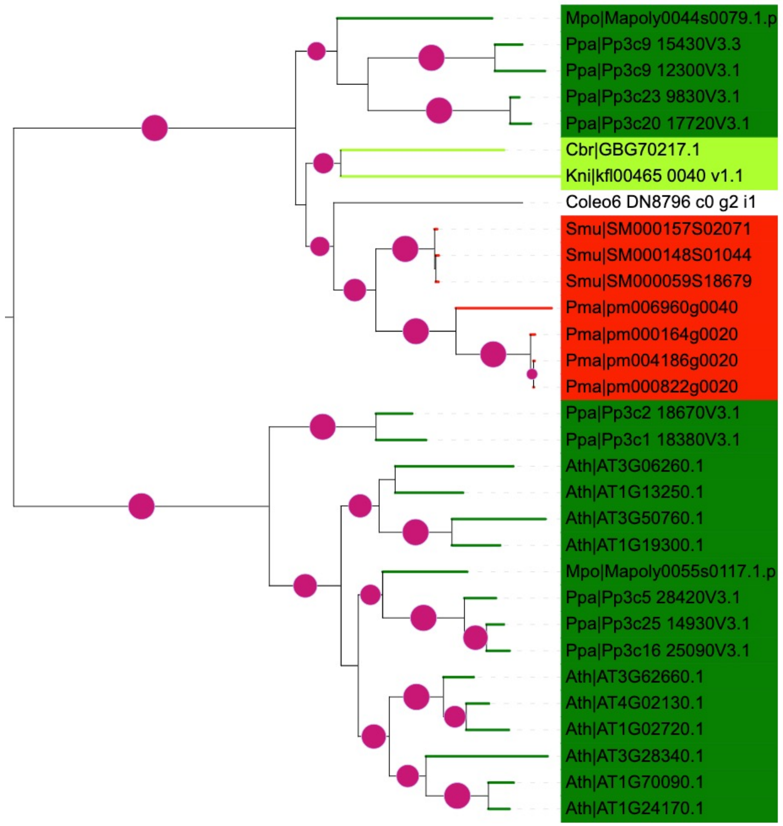

GUX1 (AT3G18660)

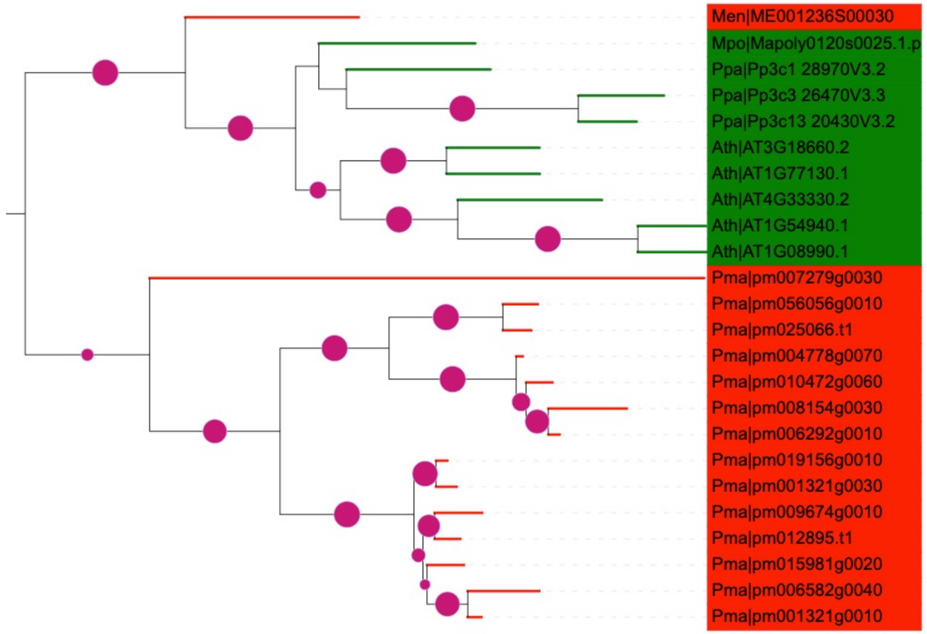

Data S1-39b

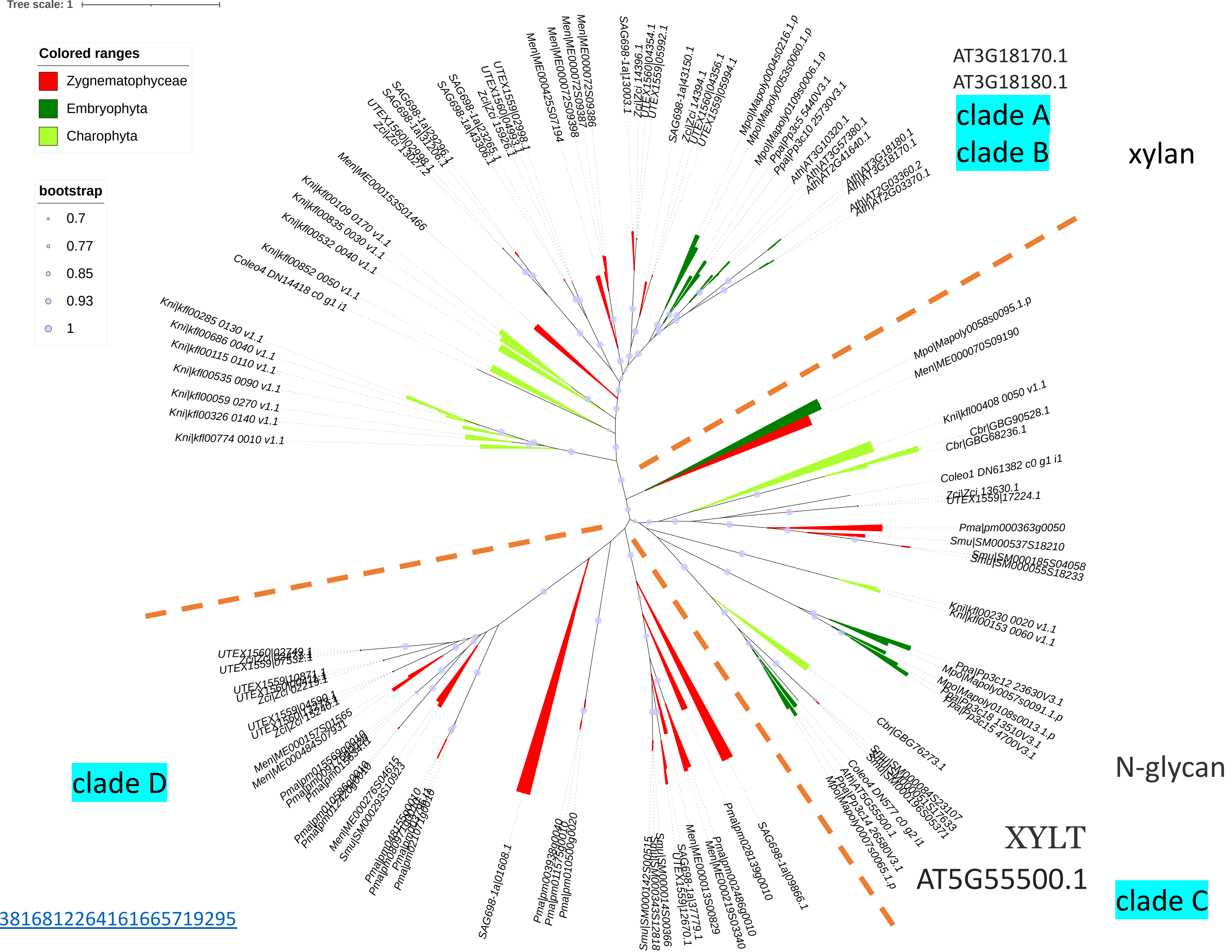

https://itol.embl.de/tree/13423816812264161665719295

Data S1-39c

https://itol.embl.de/tree/13423816812471401665758298

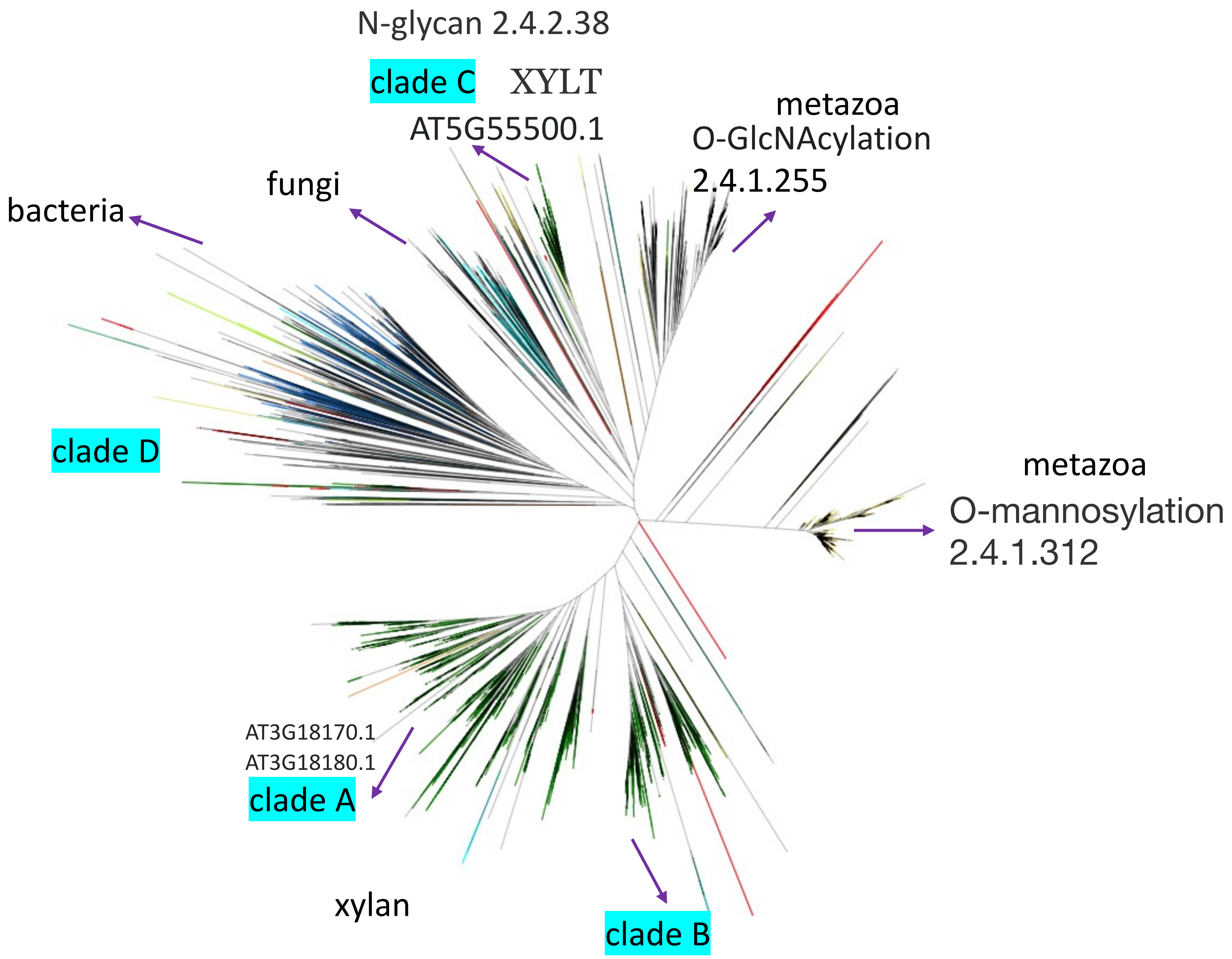

Data S1-40

GXMT1 + nr

DUF579
PF04669

https://itol.embl.de/tree/2081277025533432166122408

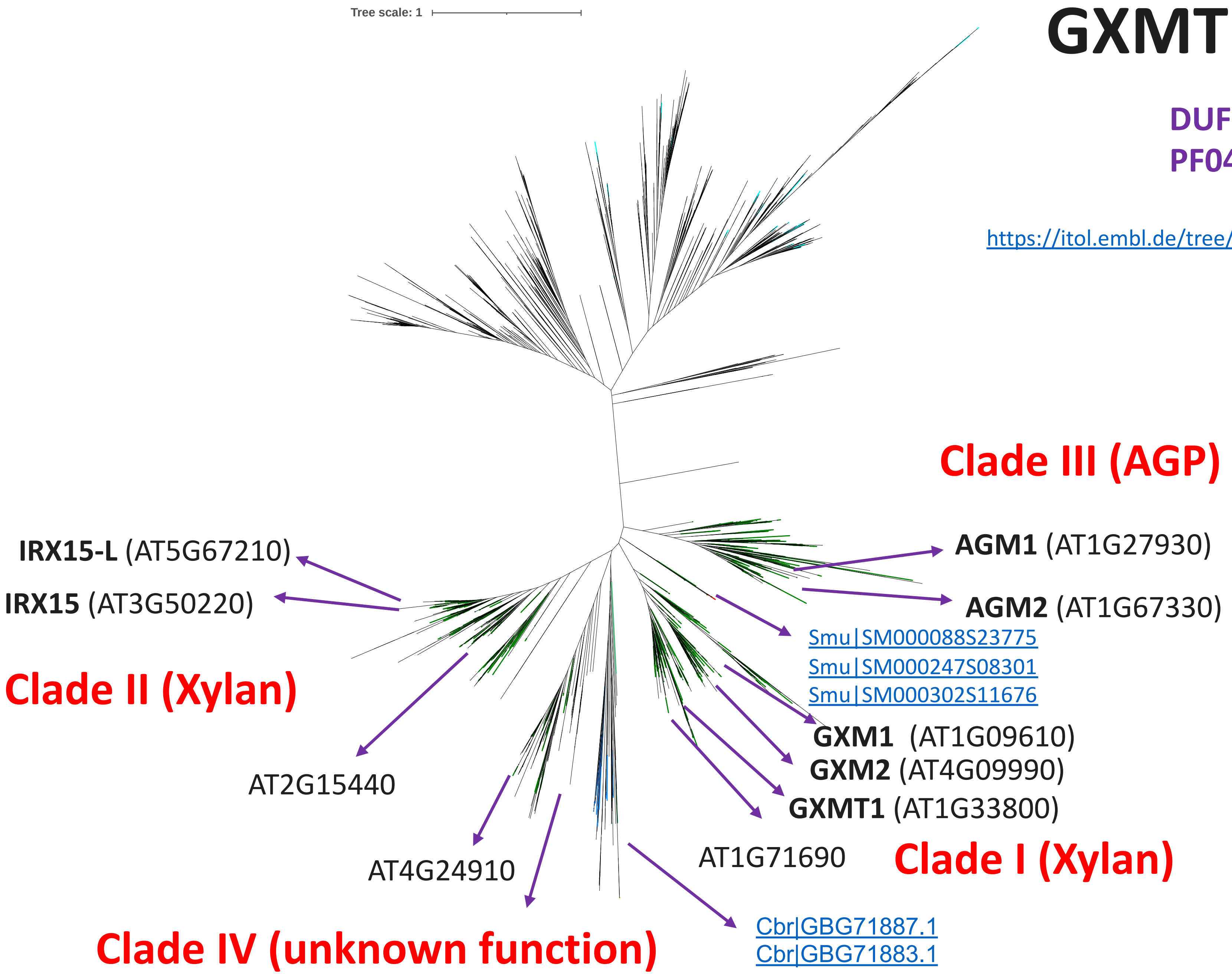

Data S1-41

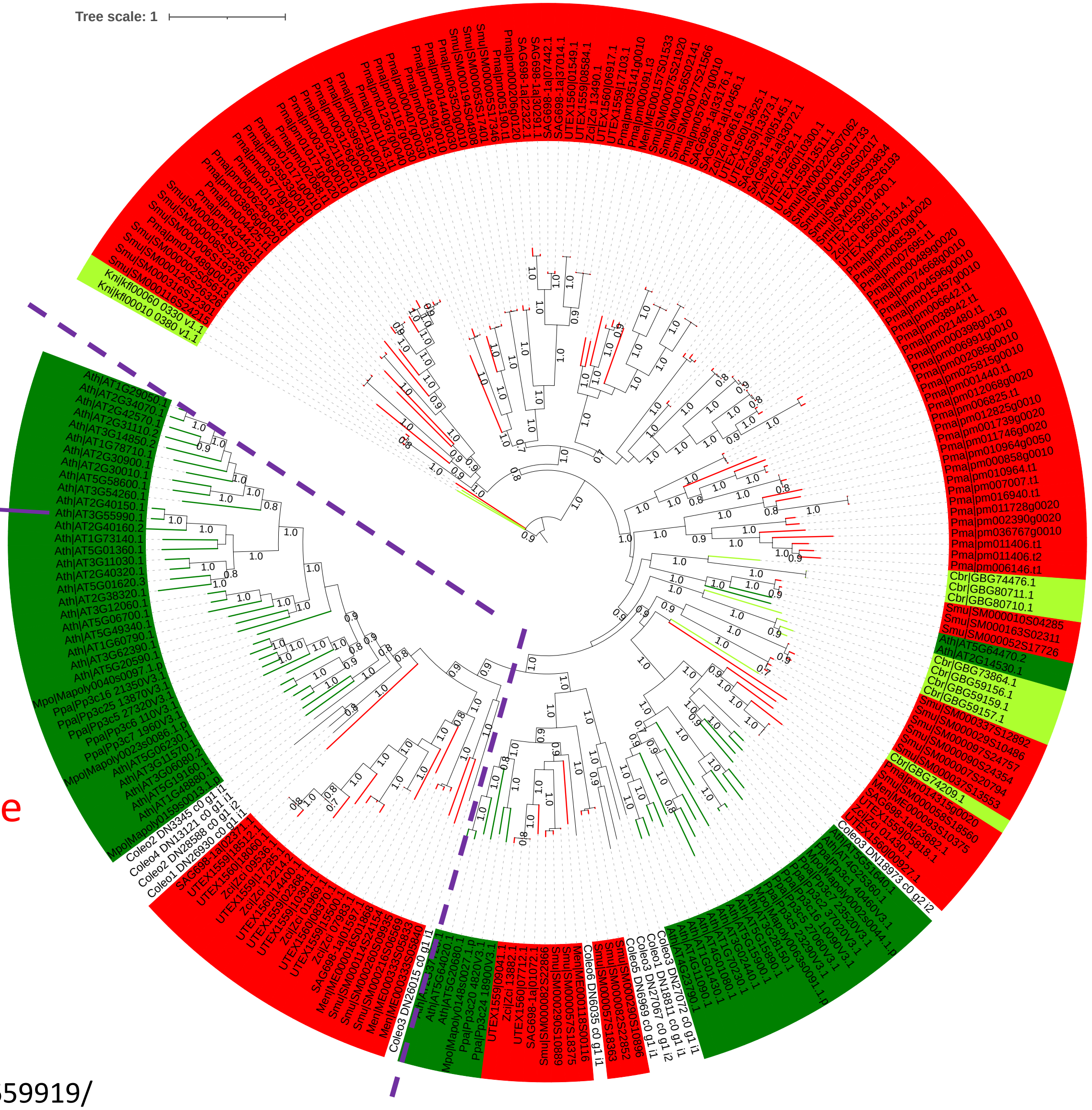

ESK1 (AT3G55990)

LCA of Zygnematophyceae and land plants likely already contained Xylan O-acetyltransferases

References
https://pubmed.ncbi.nlm.nih.gov/23659919/
https://pubmed.ncbi.nlm.nih.gov/25141999/

Data S1-42

BS1 + DARX1

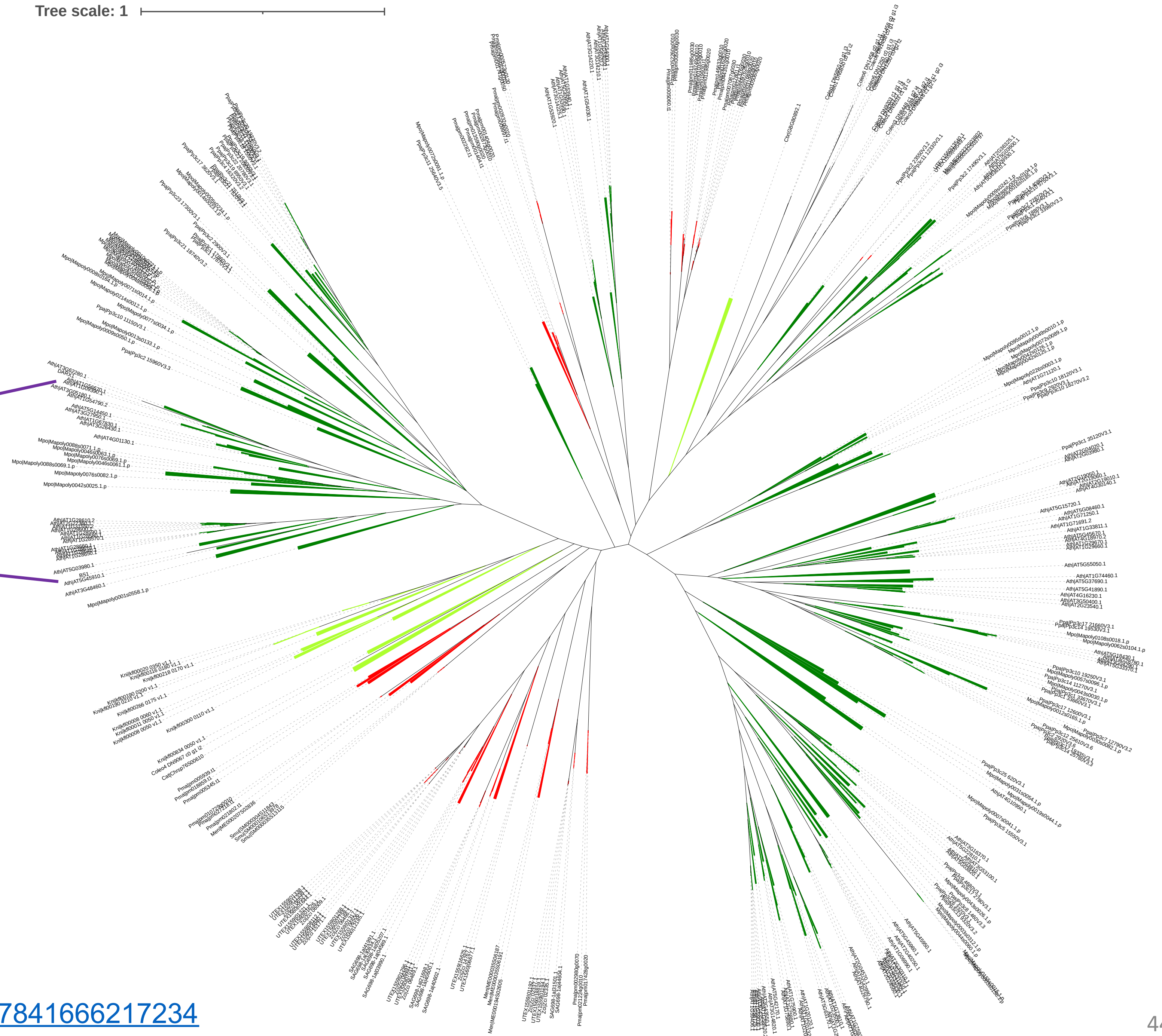

DARX1 (AT1G09390)

BS1 (AT5G45910)

https://itol.embl.de/tree/1342381681226784166621723

Data S1-43

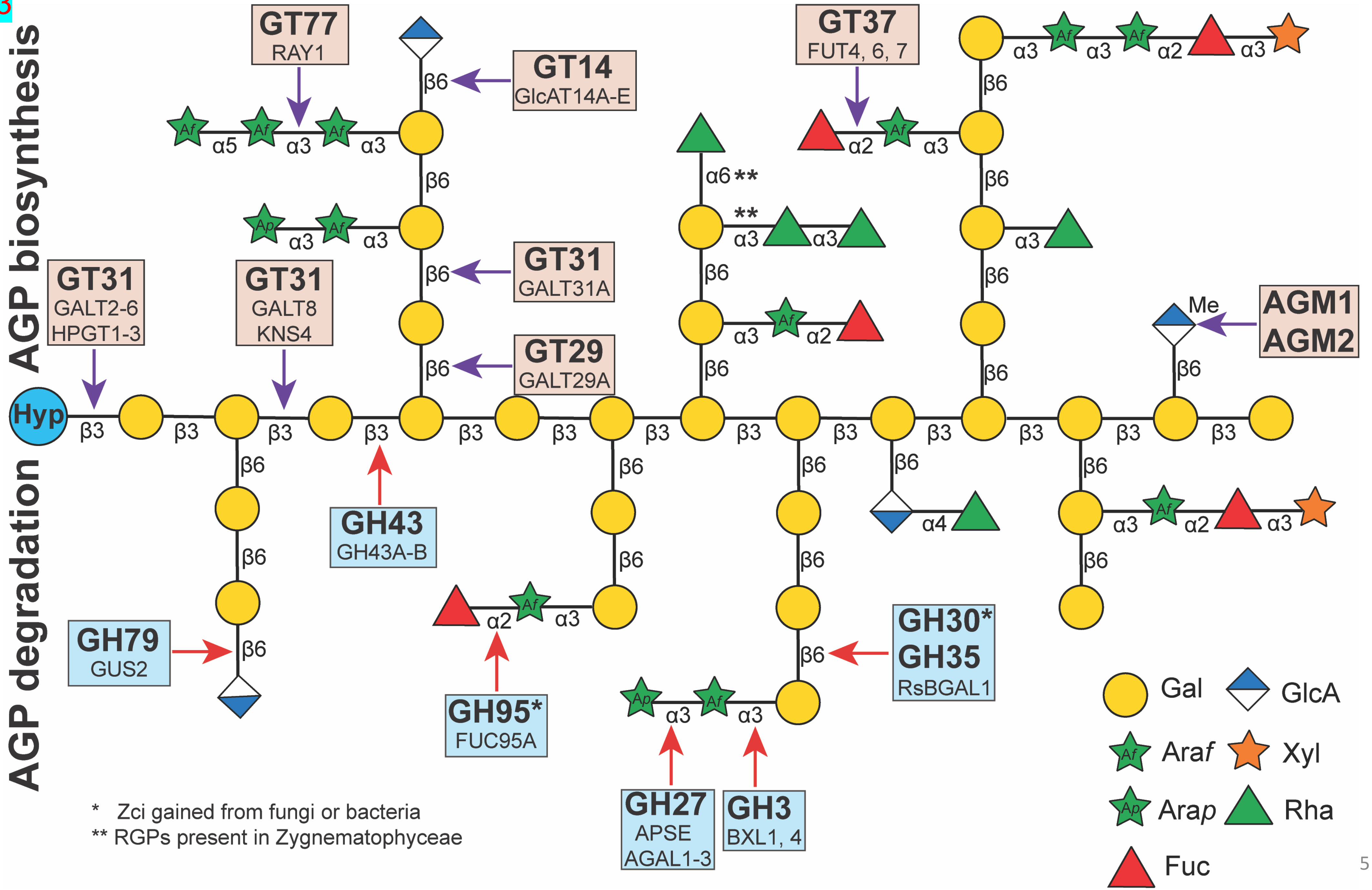
AGP biosynthesis
AGP degradation
GT77
RAY1
GT14
GlcAT14A-E
GT31
GALT31A
GT29
GALT29A
GT31
GALT2-6
HPGT1-3
GT31
GALT8
KNS4
GT37
FUT4, 6, 7
AGM1
AGM2
Me
Hyp
GH43
GH43A-B
GH79
GUS2
GH95*
FUC95A
GH27
APSE
AGAL1-3
GH3
BXL1, 4
GH30*
GH35
RsBGAL1
Gal
GlcA
Araf
Xyl
Arap
Rha
Fuc
* Zci gained from fungi or bacteria
** RGPs present in Zygnematophyceae

AGP

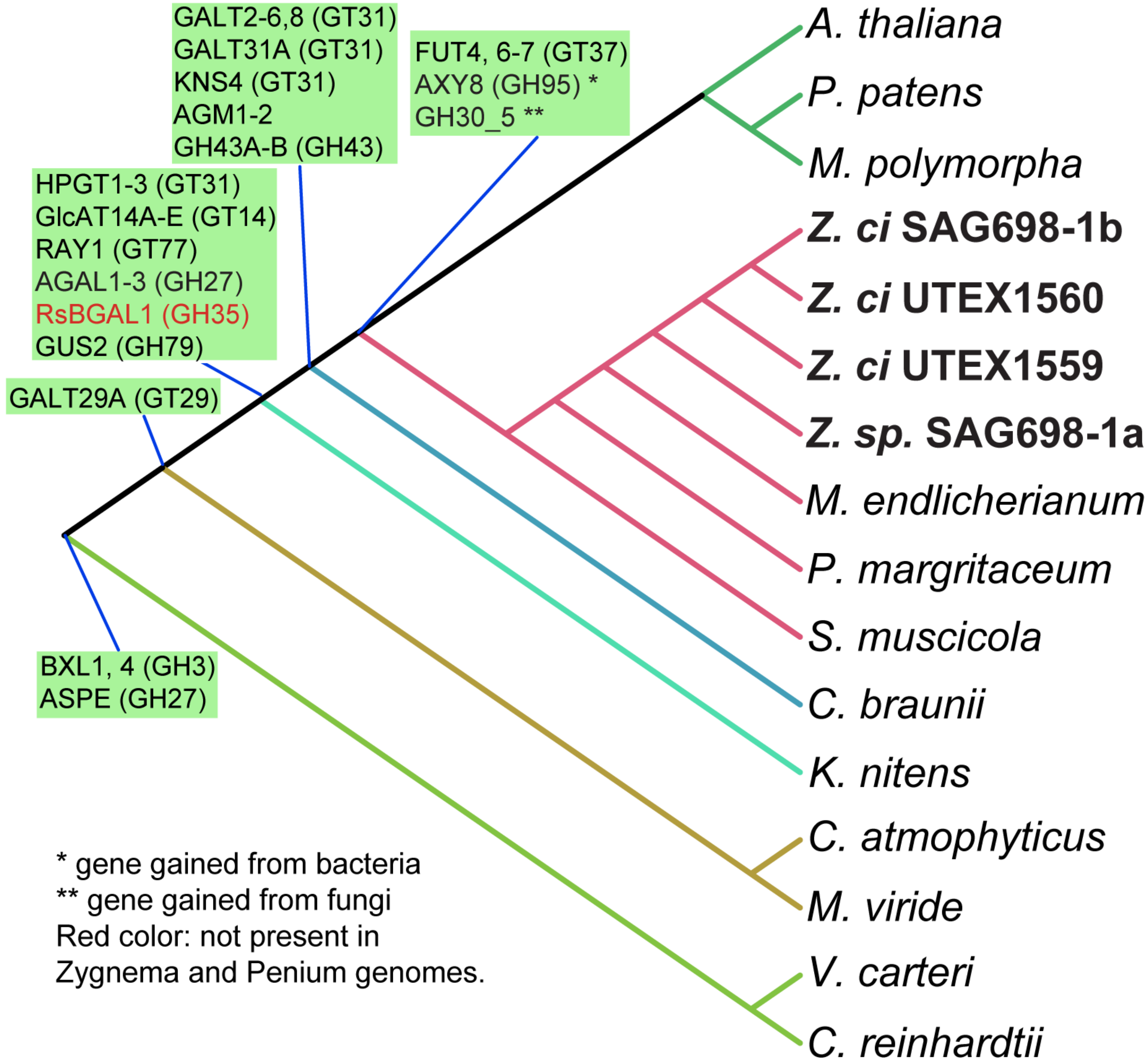

* gene gained from bacteria
** gene gained from fungi
Red color: not present in Zygnema and Penium genomes.

Data S1-44

GT31

AT5G53340 (HPGT1)

AT2G25300 (HPGT3)

AT4G32120 (HPGT2)

Arabidopsis Hyp O-galactosyltransferases: HPGT1, HPGT2 and HPGT3

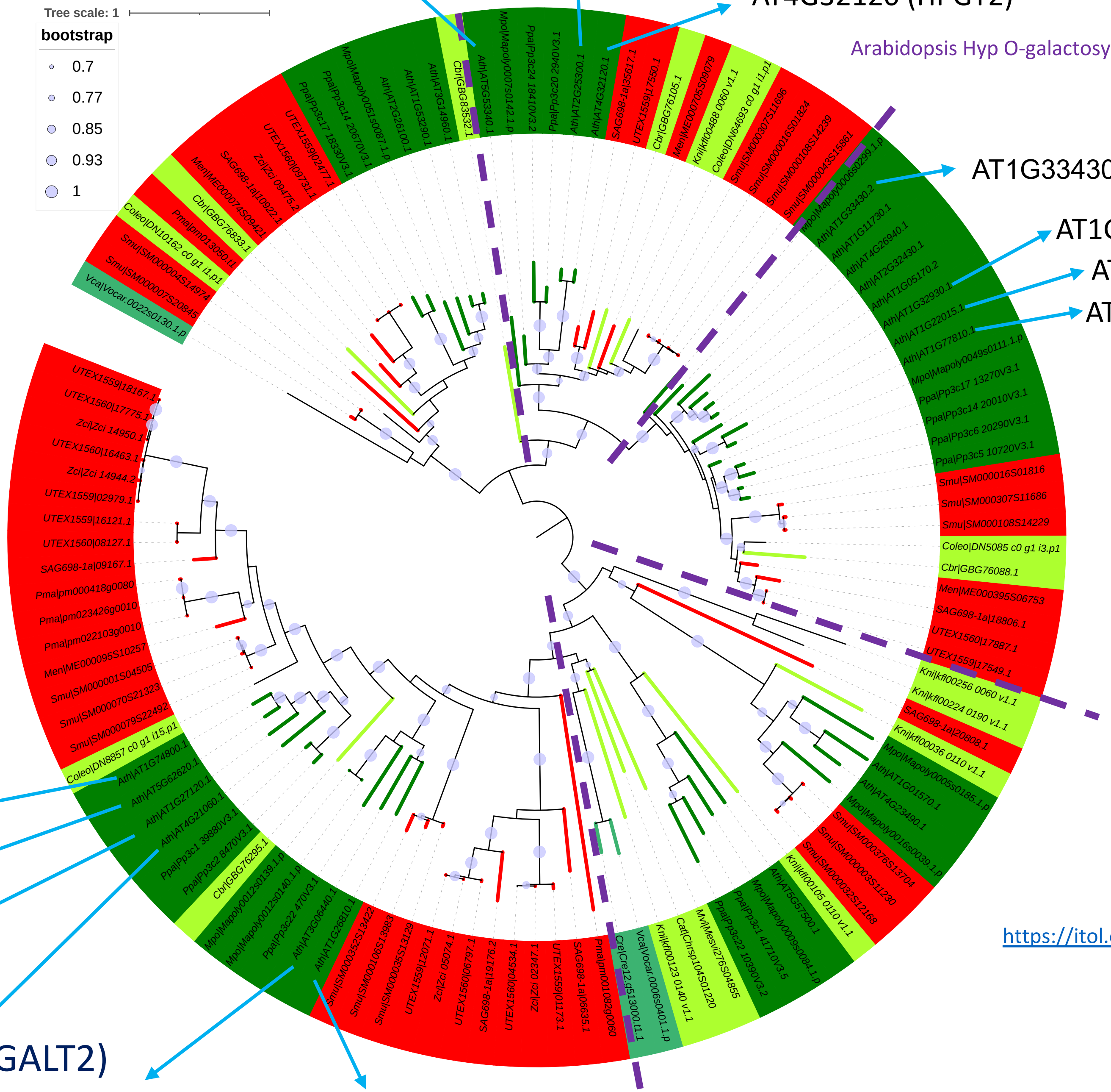

Basu, D., Liang, Y., Liu, X., Himmeldirk, K., Faik, A., Kieliszewski, M., et al. (2013). Functional identification of a hydroxyproline-o-galactosyltransferase specific for arabinogalactan protein biosynthesis in Arabidopsis. J. Biol. Chem. 288, 10132–10143. doi: 10.1074/jbc.M112.432609

Basu D, Wang W, Ma S, DeBrosse T, Poirier E, Emch K, Soukup E, Tian L, Showalter AM. Two Hydroxyproline Galactosyltransferases, GALT5 and GALT2, Function in Arabinogalactan-Protein Glycosylation, Growth and Development in Arabidopsis. PLoS One. 2015 May 14;10(5):e0125624. doi: 10.1371/journal.pone.0125624.

Basu D, Tian L, Wang W, Bobbs S, Herock H, Travers A, Showalter AM. A small multigene hydroxyproline-O-galactosyltransferase family functions in arabinogalactan-protein glycosylation, growth and development in Arabidopsis. BMC Plant Biol. 2015 Dec 21;15:295. doi: 10.1186/s12870-015-0670-7.

Strasser R, Bondili JS, Vavra U, Schoberer J, Svoboda B, Glössl J, Léonard R, Stadlmann J, Altmann F, Steinkellner H, Mach L. A unique beta1,3-galactosyltransferase is indispensable for the biosynthesis of N-glycans containing Lewis a structures in Arabidopsis thaliana. Plant Cell. 2007 Jul;19(7):2278-92. doi: 10.1105/tpc.107.052985.

AT1G33430 (KNS4/UPEX1)

AT1G32930 (*AtGALT31A*)

AT1G22015 (*AtGALT8*)

AT1G77810 (β-1,3-GalT)

Ogawa-Ohnishi M, Matsubayashi Y. Identification of three potent hydroxyproline O-galactosyltransferases in Arabidopsis. Plant J. 2015 Mar;81(5):736-46. doi: 10.1111/tpj.12764.

Geshi, N., Johansen, J. N., Dilokpimol, A., Rolland, A., Belcram, K., Verger, S., et al. (2013). A galactosyltransferase acting on arabinogalactan protein glycans is essential for embryo development in Arabidopsis. Plant J. 76, 128–137. doi: 10.1111/tpj.12281

Narciso JO, Zeng W, Ford K, Lampugnani ER, Humphries J, Austarheim I, van de Meene A, Bacic A, Doblin MS. Biochemical and Functional Characterization of GALT8, an Arabidopsis GT31 β-(1,3)-Galactosyltransferase That Influences Seedling Development. Front Plant Sci. 2021 May 25;12:678564. doi: 10.3389/fpls.2021.678564.

https://itol.embl.de/tree/208127240222157721668394053

AT1G74800 (AtGALT5)

AT1G27120 (AtGALT4)

AT5G62620 (AtGALT6)

AT4G21060 (AtGALT2)

AT3G06440 (AtGALT3)

AT1G26810 (AtGALT1)

Data S1-45

https://itol.embl.de/tree/208127240222157721668394053

At4G32120
(HPGT2)

At2G25300
(HPGT3)

At5G53340
(HPGT1)

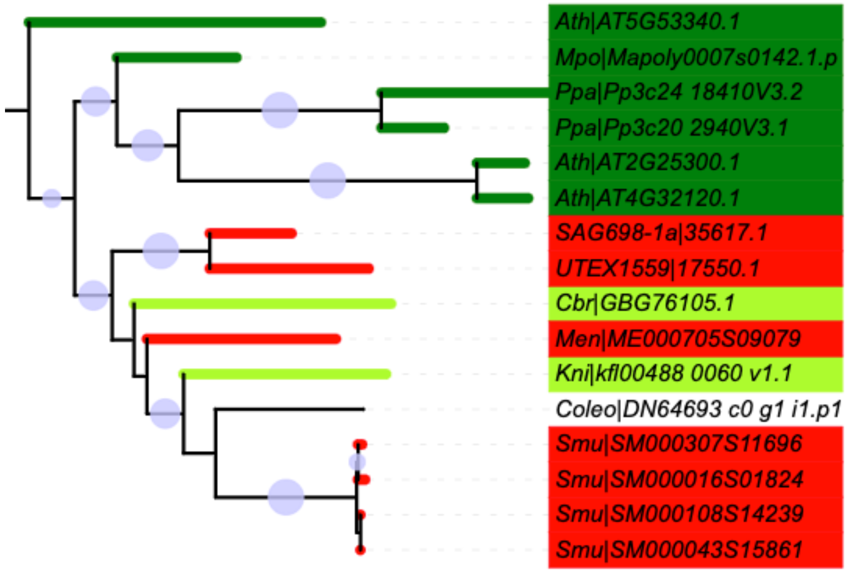

β-1,3-galactan (backbone)
initiate

GALT2-6 At4g21060

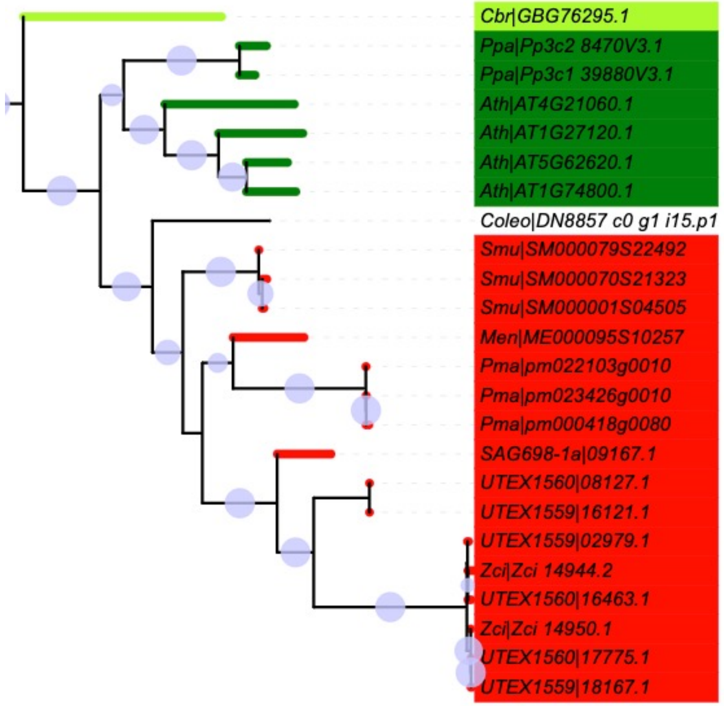

β-1,3-galactan
initiate

At1g33430
(KNS4/UPEX1)

At1g22015
(*AtGALT8*)

At1G32930
(*AtGALT31A*)

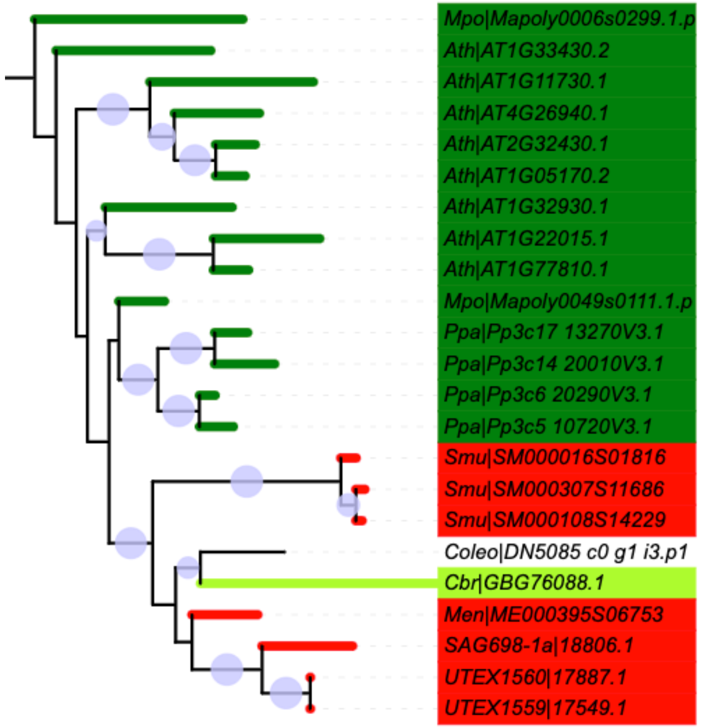

β-1,3-galactan
β-1,6-galactan (sidechain)
elongate

Data S1-46

GT29 + Coleochaete

https://itol.embl.de/tree/2081278323533483166511609 2

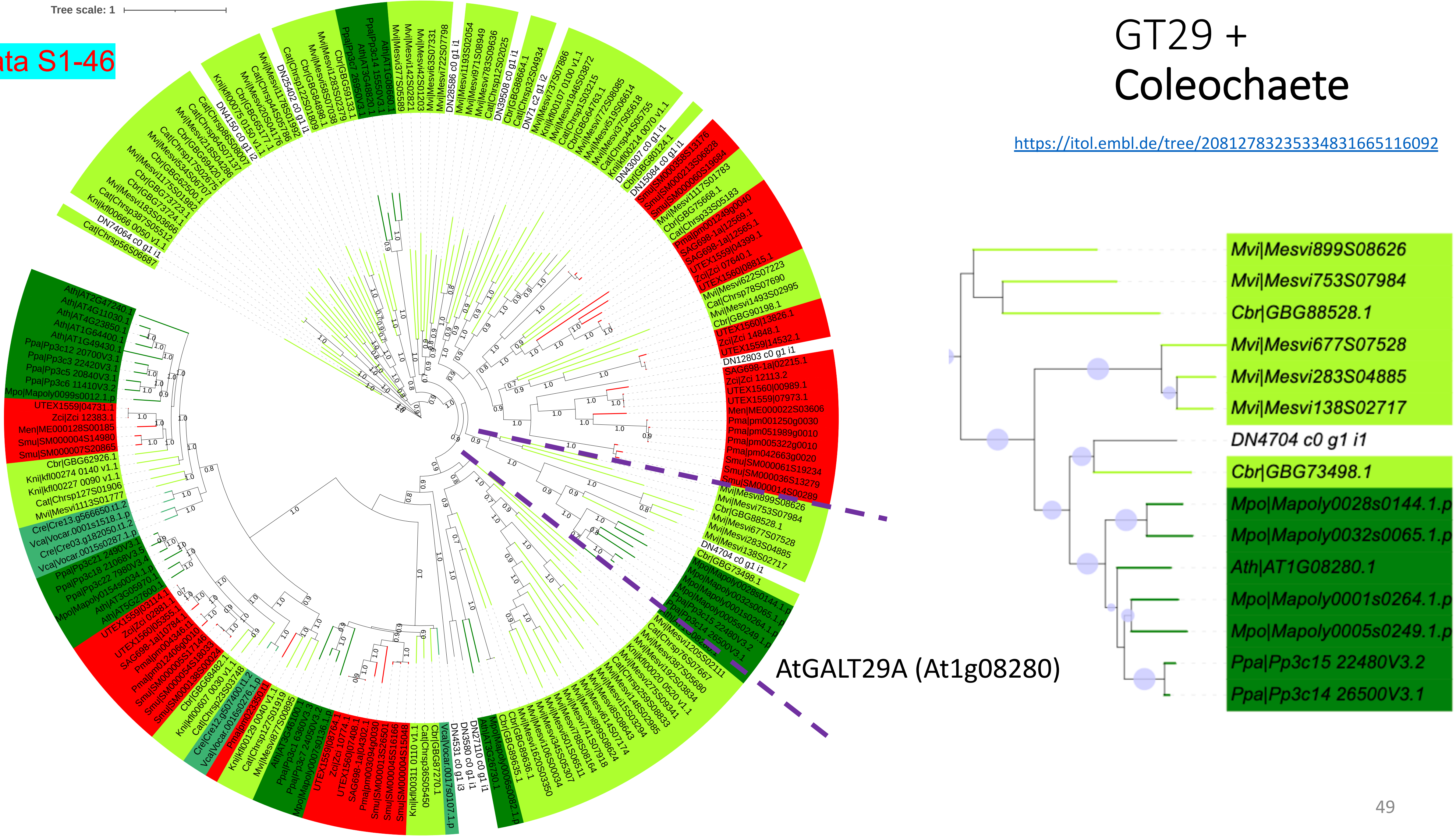

Data S1-47

https://itol.embl.de/tree/1342381641712360716651770614

AGP

At1g70630
(REDUCED
ARABINOSE
YARIV1, **RAY1**)

a putative arabinofuranosyltransferase

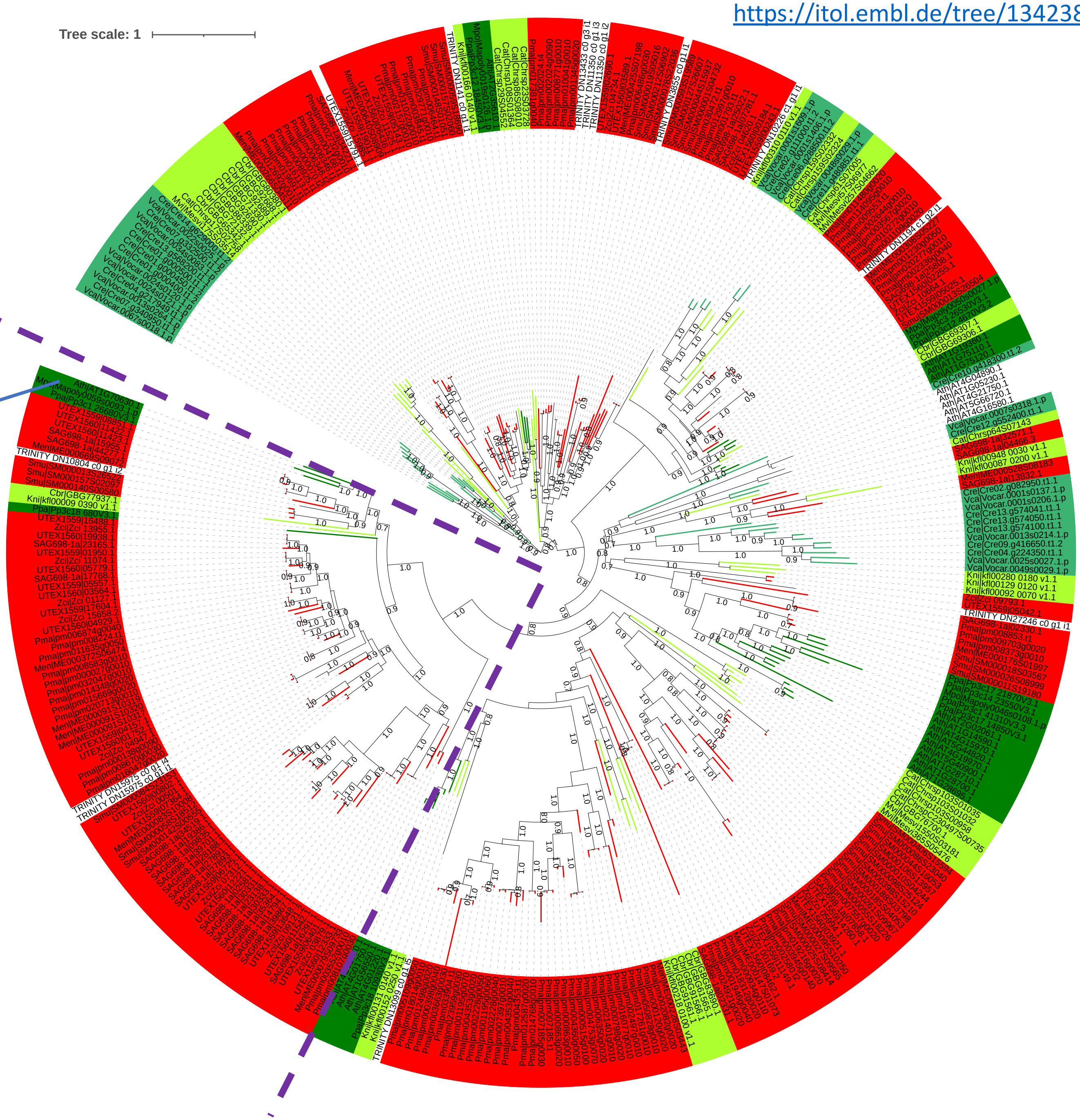

Gille, S., Sharma, V., Baidoo, E. E. K., Keasling, J. D., Scheller, H. V., and Pauly, M. (2013). Arabinosylation of a yariv-precipitable cell wall polymer impacts plant growth as exemplified by the arabidopsis glycosyltransferase mutant ray1. Mol. Plant 6, 1369–1372. doi: 10.1093/mp/sst029

GT77 + Coleochaete

Data S1-48

GT14

AtGlcAT14D (AT3G24040)

AtGlcAT14C (At2g37585)

AtGlcAT14E (AT3G15350)

AtGlcAT14A (At5g39990)

AtGlcAT14B (At5g15050)

biosynthesis of AGP glycans, β-GlcAT activity

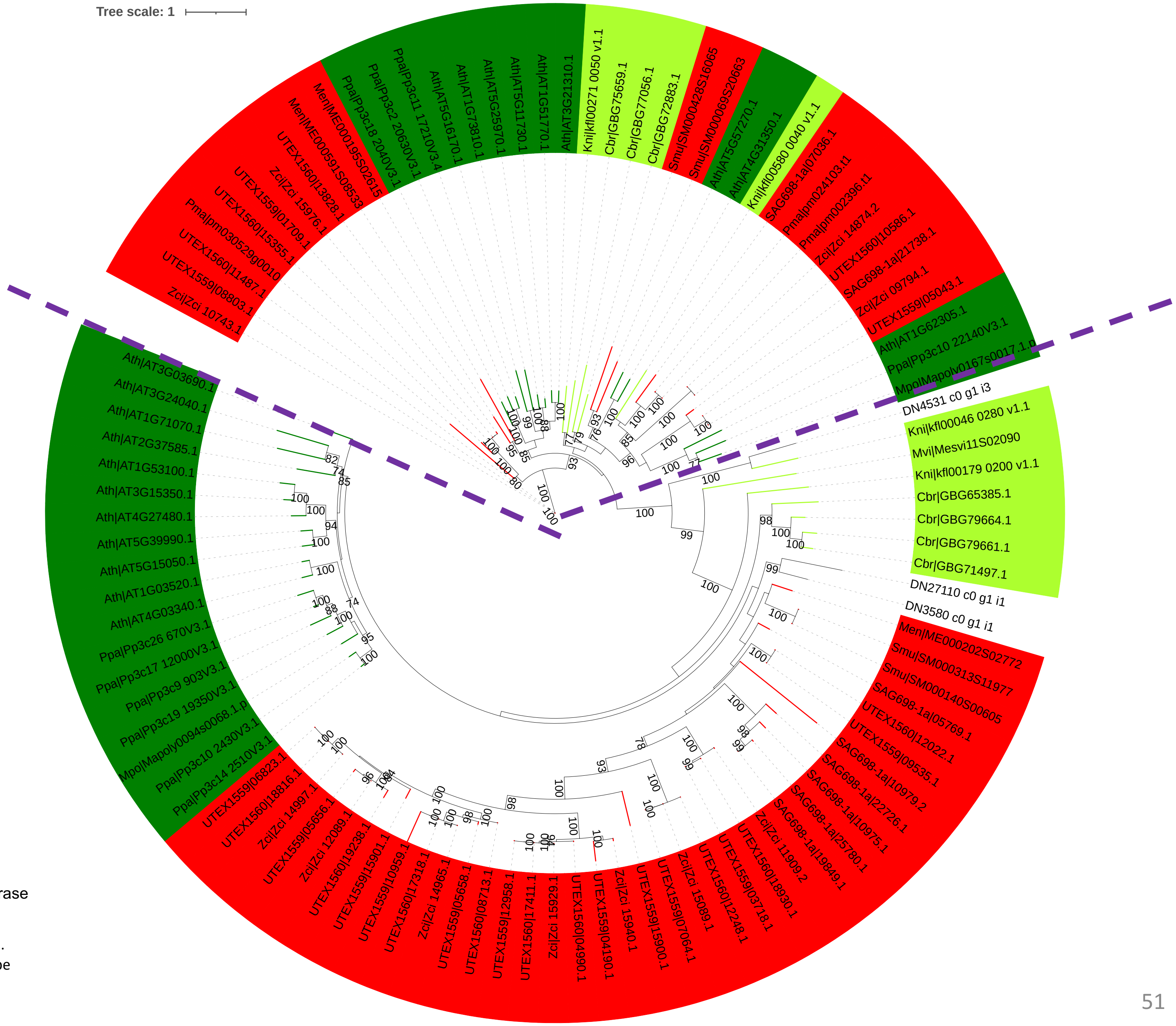

Dilokpimol, A., and Geshi, N. (2014). Arabidopsis thaliana glucuronosyltransferase in family GT14. Plant Signal. Behav. 9:e28891. doi: 10.4161/psb.28891

Knoch, E., Dilokpimol, A., Tryfona, T., Poulsen, C. P., Xiong, G., Harholt, J., et al. (2013). A β-glucuronosyltransferase from Arabidopsis thaliana involved in biosynthesis of type II arabinogalactan has a role in cell elongation during seedling growth. Plant J. 76, 1016–1029. doi: 10.1111/tpj.12353

Data S1-49

GT37

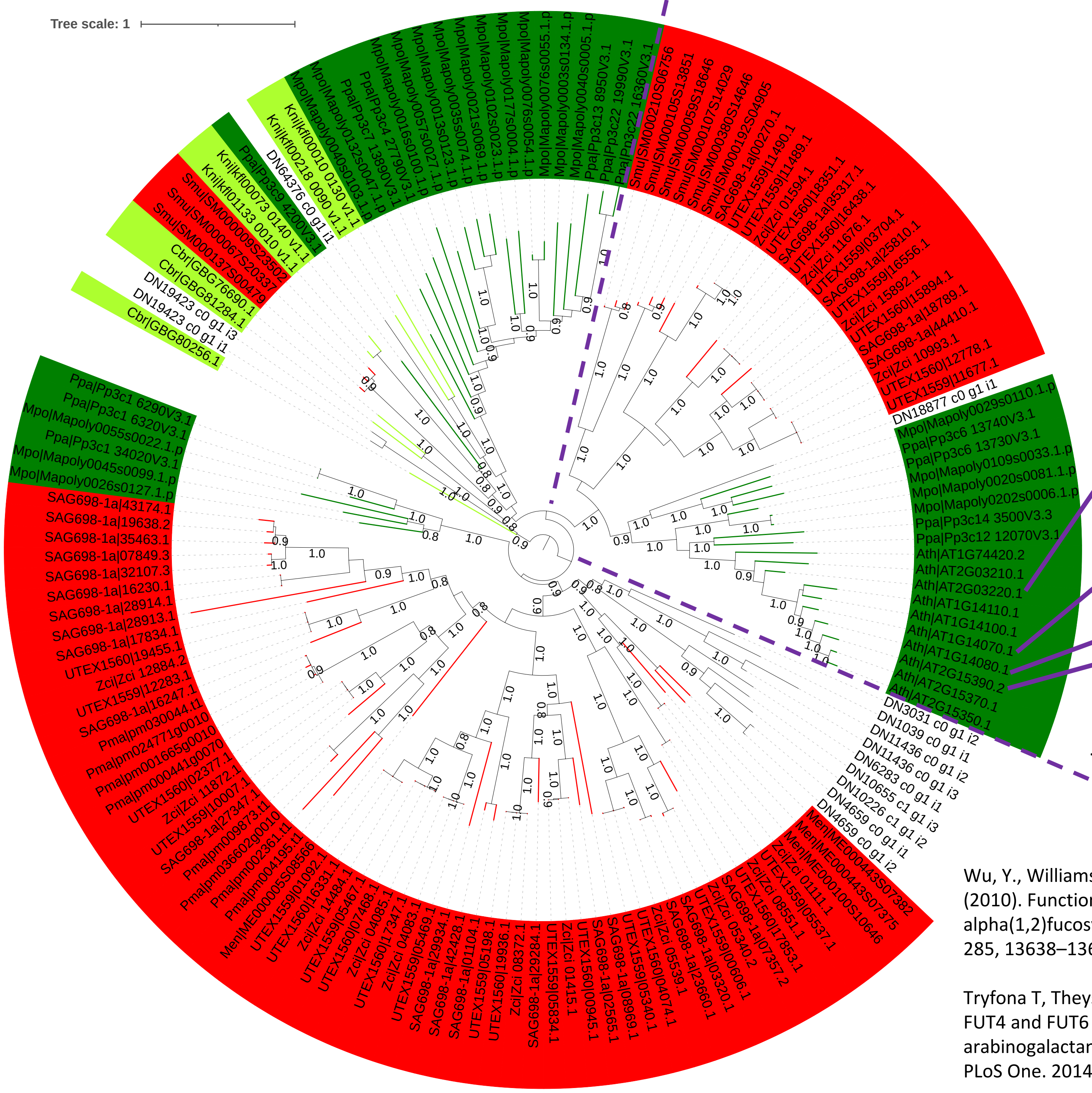

Vanzin GF, Madson M, Carpita NC, Raikhel NV, Keegstra K, Reiter WD. The mur2 mutant of Arabidopsis thaliana lacks fucosylated xyloglucan because of a lesion in fucosyltransferase AtFUT1. Proc Natl Acad Sci U S A. 2002 Mar 5;99(5):3340-5. doi: 10.1073/pnas.052450699.

AtFUT1 (*At2g03220*)

AtFUT7 (At1g14070)

AtFUT6 (*At1g14080*)

AtFUT4 (*At2g15390*)

Two AtGT37 are α-1,2-fucosyltransferases

Link to biosynthesis of AGP glycans

Wu, Y., Williams, M., Bernard, S., Driouich, A., Showalter, A. M., and Faik, A. (2010). Functional identification of two nonredundant Arabidopsis alpha(1,2)fucosyltransferases specific to arabinogalactan proteins. J. Biol. Chem. 285, 13638–13645. doi: 10.1074/jbc.M110.102715

Tryfona T, Theys TE, Wagner T, Stott K, Keegstra K, Dupree P. Characterisation of FUT4 and FUT6 α-(1 → 2)-fucosyltransferases reveals that absence of root arabinogalactan fucosylation increases Arabidopsis root growth salt sensitivity. PLoS One. 2014 Mar 25;9(3):e93291. doi: 10.1371/journal.pone.0093291.

Data S1-50

hmmsearch PF04669

Polysacc_synt_4	190	Ath|AT1G09610.1	282	3.8e-70	1	190	79	263	0.994736842105263
Polysacc_synt_4	190	Ath|AT1G27930.1	289	1.8e-74	1	190	83	270	0.994736842105263
Polysacc_synt_4	190	Ath|AT1G33800.1	297	1.5e-71	2	190	93	275	0.989473684210526
Polysacc_synt_4	190	Ath|AT1G67330.1	291	2.5e-75	1	190	86	274	0.994736842105263
Polysacc_synt_4	190	Ath|AT1G71690.1	295	2.4e-77	2	190	92	278	0.989473684210526
Polysacc_synt_4	190	Ath|AT2G15440.1	329	3e-71	2	190	99	287	0.989473684210526
Polysacc_synt_4	190	Ath|AT3G50220.1	322	8.9e-74	2	189	109	296	0.984210526315789
Polysacc_synt_4	190	Ath|AT4G09990.1	290	7.5e-70	2	190	85	268	0.989473684210526
Polysacc_synt_4	190	Ath|AT4G24910.1	315	7.2e-65	2	190	105	295	0.989473684210526
Polysacc_synt_4	190	Ath|AT5G67210.1	317	1.2e-73	2	189	102	289	0.984210526315789
Polysacc_synt_4	190	Cbr|GBG71883.1	344	2.2e-16	17	176	165	316	0.836842105263158
Polysacc_synt_4	190	Cbr|GBG71887.1	339	9.2e-14	17	175	152	302	0.831578947368421
Polysacc_synt_4	190	Smu|SM000088S23775	194	8.5e-21	2	106	84	193	0.547368421052632
Polysacc_synt_4	190	Smu|SM000247S08301	194	5.5e-21	2	106	84	193	0.547368421052632
Polysacc_synt_4	190	Smu|SM000302S11676	194	3.4e-21	2	106	84	193	0.547368421052632

AGM1 & AGM2

blastp

Ath|AT2G15440.1	AGM2	36.604	265	150	8	51	303	33	291	5.73e-55	169
Ath|AT2G15440.1	AGM1	38.034	234	133	7	76	303	62	289	1.30e-54	168
Ath|AT1G67330.1	AGM2	100.000	291	0	0	1	291	1	291	0.0	611
Ath|AT1G67330.1	AGM1	68.905	283	81	5	15	291	8	289	2.32e-142	390
Ath|AT1G71690.1	AGM1	51.174	213	102	2	70	281	62	273	2.63e-80	233
Ath|AT1G71690.1	AGM2	45.385	260	136	5	38	294	35	291	1.42e-78	229
Ath|AT1G33800.1	AGM2	49.784	231	107	4	67	289	62	291	9.91e-81	234
Ath|AT1G33800.1	AGM1	52.511	219	99	4	70	284	62	279	5.86e-78	227
Ath|AT1G09610.1	AGM2	51.786	224	103	5	62	281	69	291	7.58e-81	234
Ath|AT1G09610.1	AGM1	52.133	211	92	3	62	266	66	273	5.14e-80	232
Ath|AT4G24910.1	AGM2	29.831	295	187	5	26	307	4	291	1.80e-42	136
Ath|AT4G24910.1	AGM1	35.238	210	125	5	105	307	84	289	1.04e-40	132
Ath|AT3G50220.1	AGM2	34.768	302	165	12	21	310	10	291	6.03e-48	151
Ath|AT3G50220.1	AGM1	37.229	231	130	7	89	310	65	289	7.55e-48	150
Ath|AT1G27930.1	AGM1	100.000	289	0	0	1	289	1	289	0.0	600
Ath|AT1G27930.1	AGM2	69.258	283	80	5	8	289	15	291	1.44e-145	399
Ath|AT4G09990.1	AGM2	44.706	255	129	2	22	272	32	278	3.71e-79	230
Ath|AT4G09990.1	AGM1	51.887	212	99	3	68	276	67	278	1.19e-75	221
Ath|AT5G67210.1	AGM2	34.932	292	172	9	20	303	10	291	8.08e-52	161
Ath|AT5G67210.1	AGM1	36.199	221	134	5	82	300	65	280	3.68e-48	151
Cbr|GBG71883.1	AGM2	31.395	172	82	5	166	315	102	259	4.28e-23	85.1
Cbr|GBG71883.1	AGM1	30.994	171	83	5	166	315	99	255	6.38e-21	79.0
Cbr|GBG71887.1	AGM1	29.825	171	85	4	153	302	99	255	4.98e-21	79.0
Cbr|GBG71887.1	AGM2	30.058	173	83	6	153	302	102	259	3.13e-19	73.9
Smu|SM000088S23775	AGM2	38.830	188	98	5	10	193	19	193	1.07e-35	115
Smu|SM000088S23775	AGM1	43.846	130	65	3	66	193	66	189	2.05e-30	101
Smu|SM000247S08301	AGM2	38.830	188	98	5	10	193	19	193	1.61e-35	114
Smu|SM000247S08301	AGM1	42.308	130	67	3	66	193	66	189	3.04e-29	98.2
Smu|SM000302S11676	AGM2	38.298	188	99	5	10	193	19	193	6.69e-35	112
Smu|SM000302S11676	AGM1	43.077	130	66	3	66	193	66	189	1.43e-29	99.0

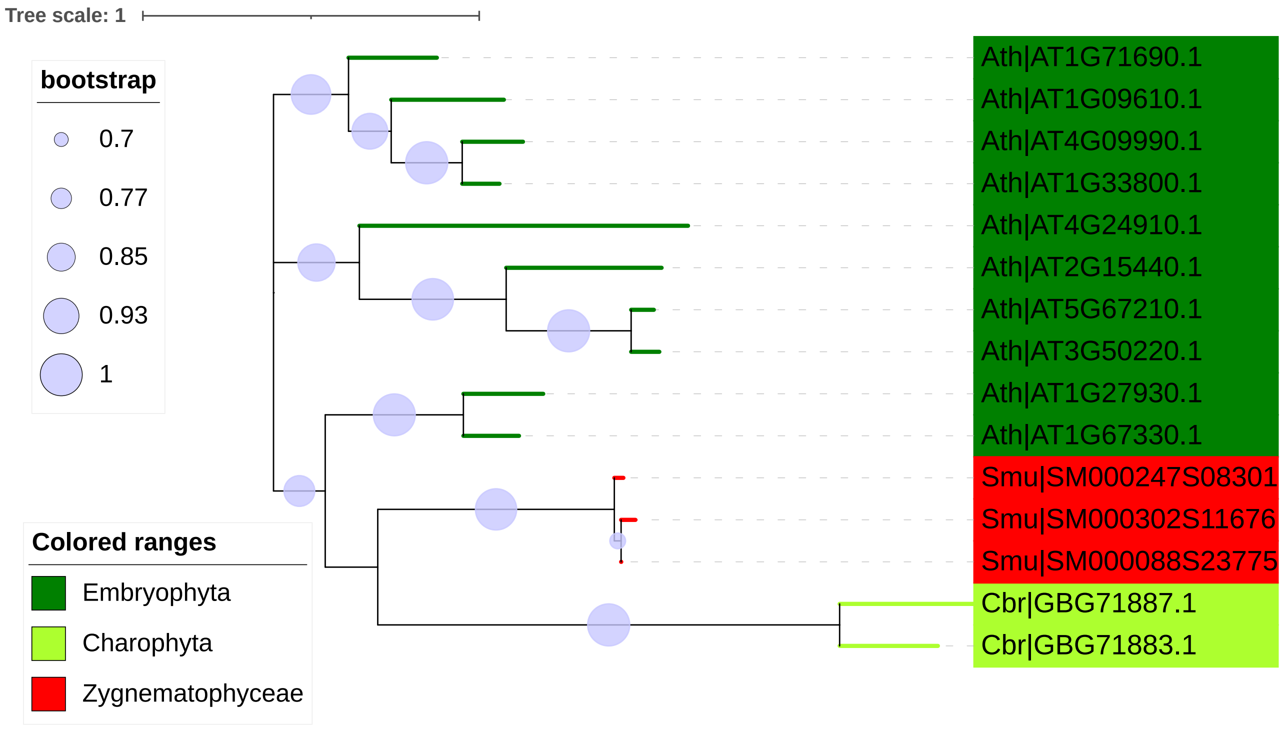

https://itol.embl.de/tree/134238191141230141690854677

See the NR phylogeny in Data S1-40

Data S1-51

https://itol.embl.de/tree/208127240222403281665195572

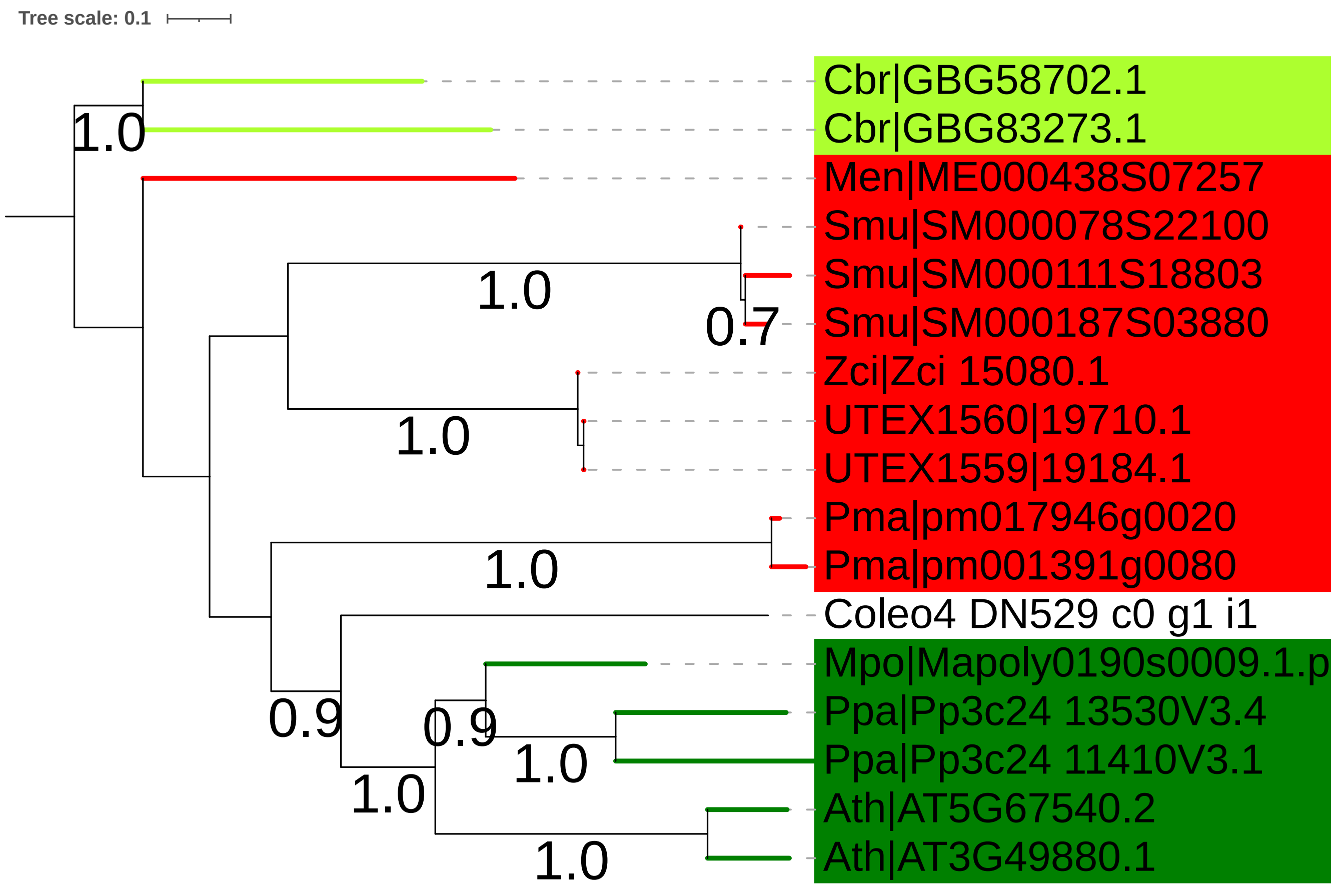

GH43A (AT5G67540)

GH43B (AT3G49880)

GH43_24 + Coleochaete

exo-β-1,3-galactosidase activity of GH43 enzymes: hampered by β-1,6 branches on β-1,3-galactans.

Nibbering P, Petersen BL, Motawia MS, Jørgensen B, Ulvskov P, Niittylä T. Golgi-localized exo-β1,3-galactosidases involved in cell expansion and root growth in Arabidopsis. J Biol Chem. 2020 Jul 31;295(31):10581-10592. doi: 10.1074/jbc.RA120.013878.

Data S1-52

GH43_24 nr

Arabidopsis GH43_24: involved in AGP backbone degradation

GH43_24: exo-b-1,3-galactanase (EC 3.2.1.145), might be HGT from bacteria.

https://itol.embl.de/tree/20812770255164771664771351

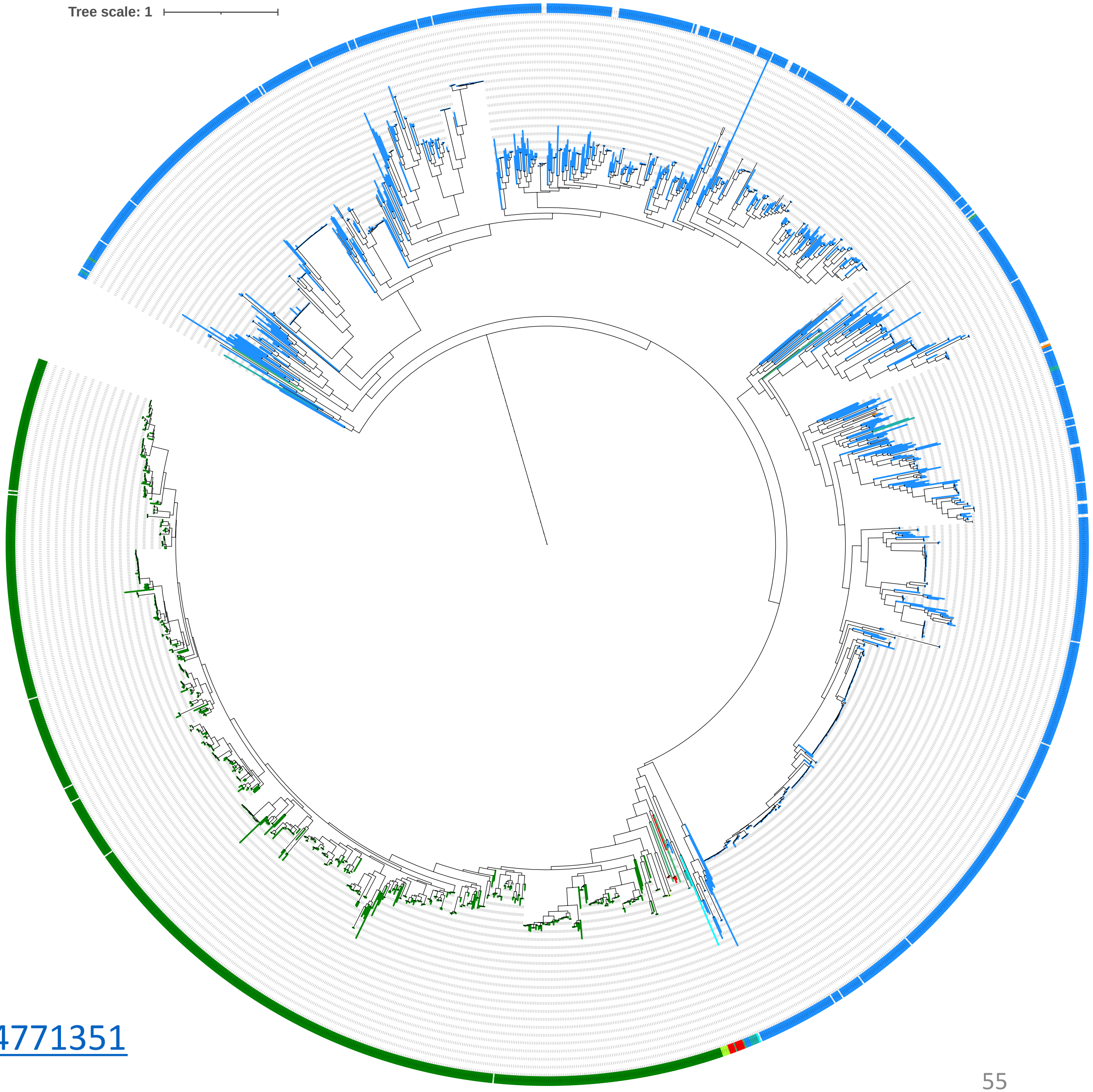

Data S1-53

GH30_5

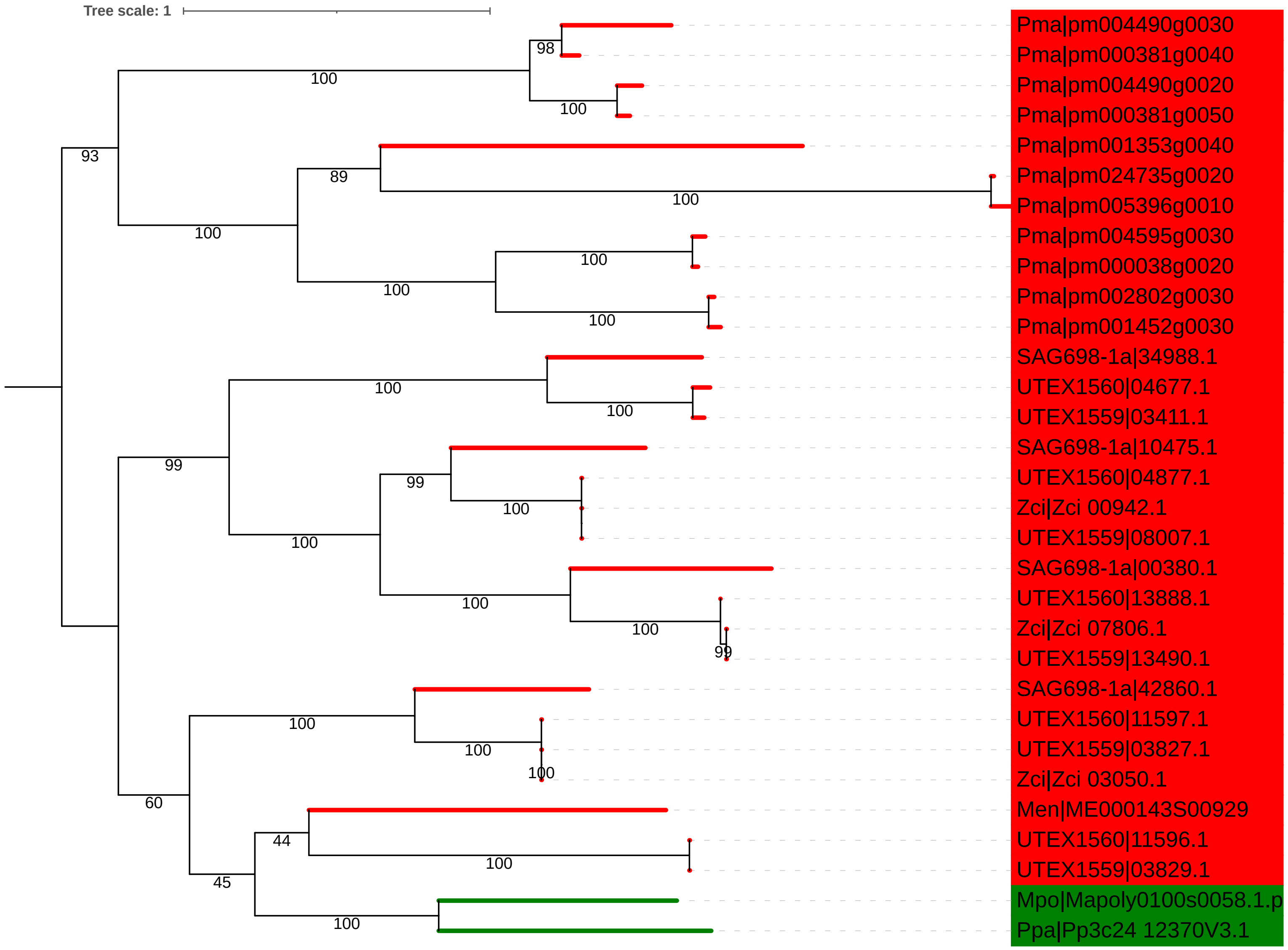

endo-b-1,6-galactanase (EC 3.2.1.164); galactan exo-1,6-b-galactobiohydrolase (non-reducing end) (EC 3.2.1.213)

No Coleochaete

Data S1-54

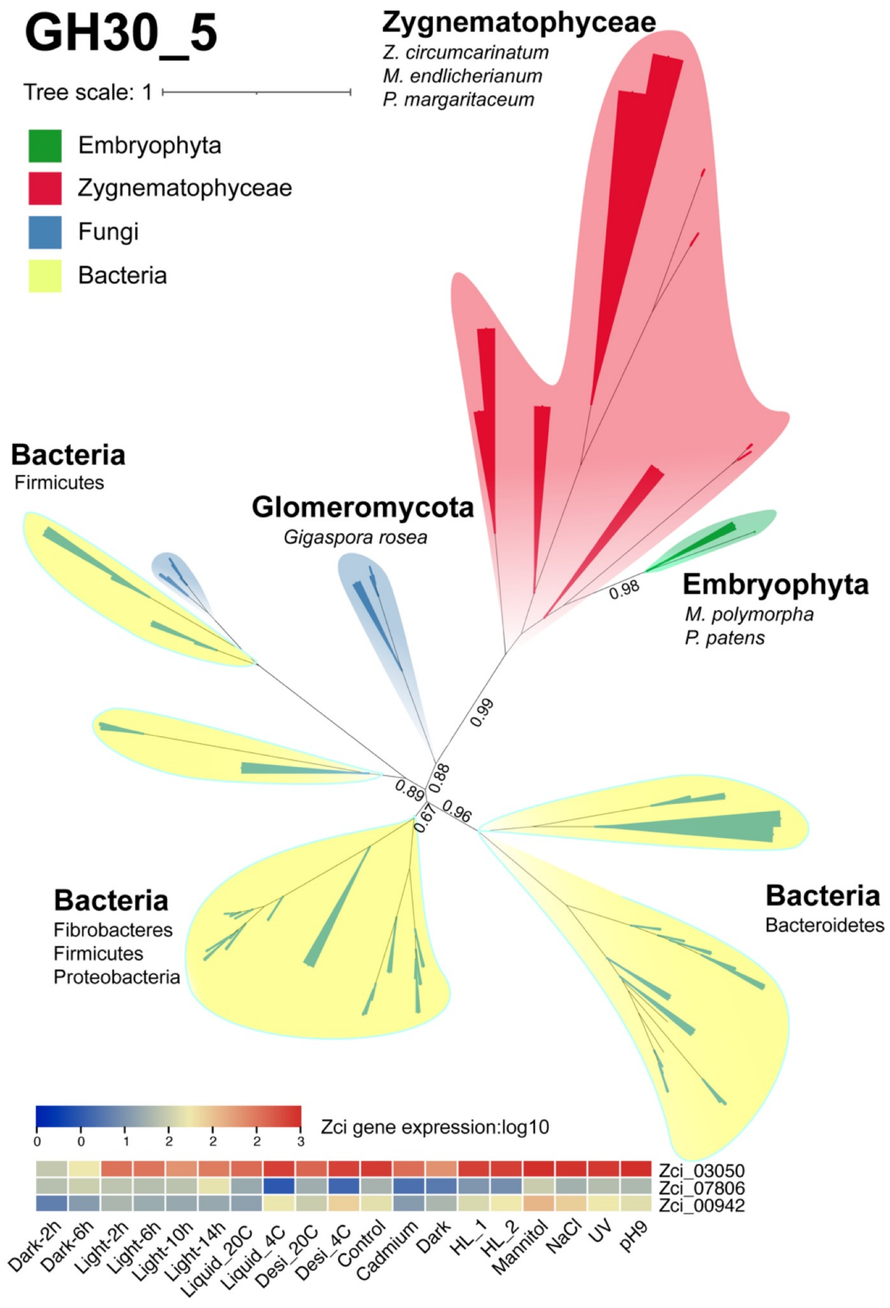

GH30_5
Tree scale: 1
Embryophyta
Zygnematophyceae
Fungi
Bacteria
Zygnematophyceae
Z. circumcarinatum
M. endlicherianum
P. margaritaceum
Bacteria
Firmicutes
Glomeromycota
Gigaspora rosea
Embryophyta
M. polymorpha
P. patens
Bacteria
Fibrobacteres
Firmicutes
Proteobacteria
Bacteria
Bacteroidetes
0.98
0.99
0.88
0.89
0.67
0.96
Zci gene expression:log10
Zci_03050
Zci_07806
Zci_00942
Dark-2h
Dark-6h
Light-2h
Light-6h
Light-10h
Light-14h
Liquid_20C
Liquid_4C
Desi_20C
Desi_4C
Control
Cadmium
Dark
HL_1
HL_2
Mannitol
NaCl
UV
pH9

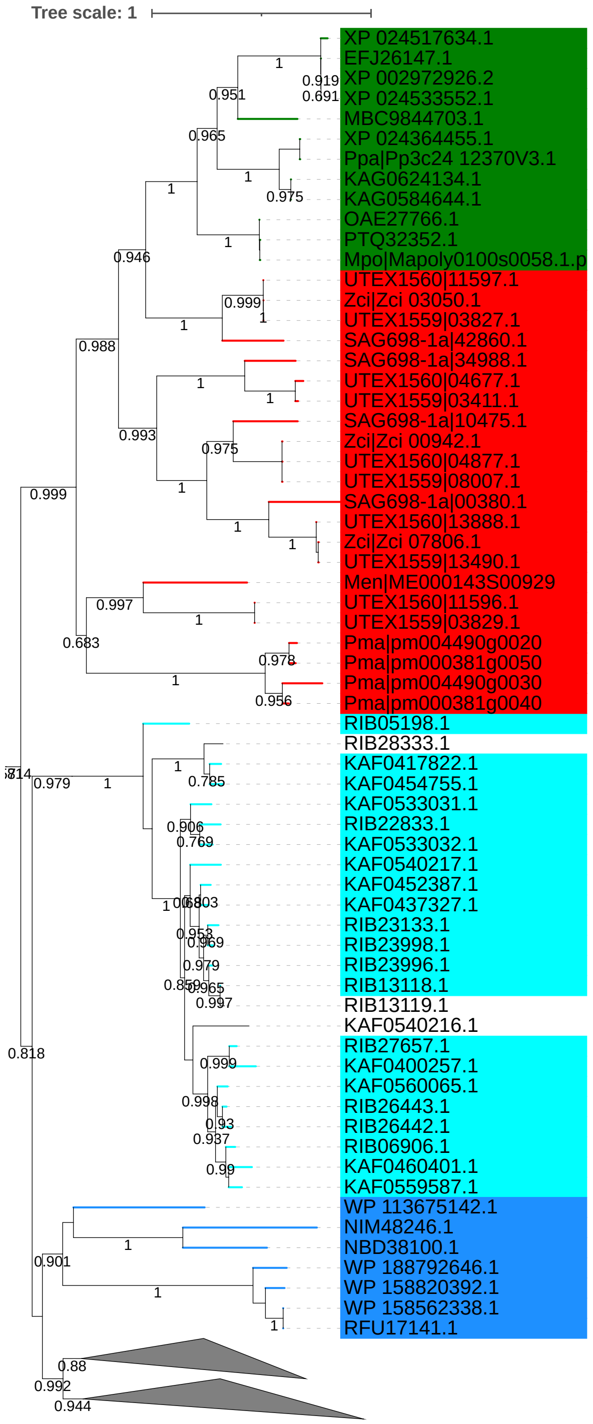

Tree scale: 1
XP 024517634.1
EFJ26147.1
XP 002972926.2
XP 024533552.1
MBC9844703.1
XP 024364455.1
Ppa|Pp3c24 12370V3.1
KAG0624134.1
KAG0584644.1
OAE27766.1
PTQ32352.1
Mpo|Mapoly0100s0058.1.p
UTEX1560|11597.1
Zci|Zci 03050.1
UTEX1559|03827.1
SAG698-1a|42860.1
SAG698-1a|34988.1
UTEX1560|04677.1
UTEX1559|03411.1
SAG698-1a|10475.1
Zci|Zci 00942.1
UTEX1560|04877.1
UTEX1559|08007.1
SAG698-1a|00380.1
UTEX1560|13888.1
Zci|Zci 07806.1
UTEX1559|13490.1
Men|ME000143S00929
UTEX1560|11596.1
UTEX1559|03829.1
Pma|pm004490g0020
Pma|pm000381g0050
Pma|pm004490g0030
Pma|pm000381g0040
RIB05198.1
RIB28333.1
KAF0417822.1
KAF0454755.1
KAF0533031.1
RIB22833.1
KAF0533032.1
KAF0540217.1
KAF0452387.1
KAF0437327.1
RIB23133.1
RIB23998.1
RIB23996.1
RIB13118.1
RIB13119.1
KAF0540216.1
RIB27657.1
KAF0400257.1
KAF0560065.1
RIB26443.1
RIB26442.1
RIB06906.1
KAF0460401.1
KAF0559587.1
WP 113675142.1
NIM48246.1
NBD38100.1
WP 188792646.1
WP 158820392.1
WP 158562338.1
RFU17141.1

Data S1-55

https://itol.embl.de/tree/2081272402227379166519770 6

GH35

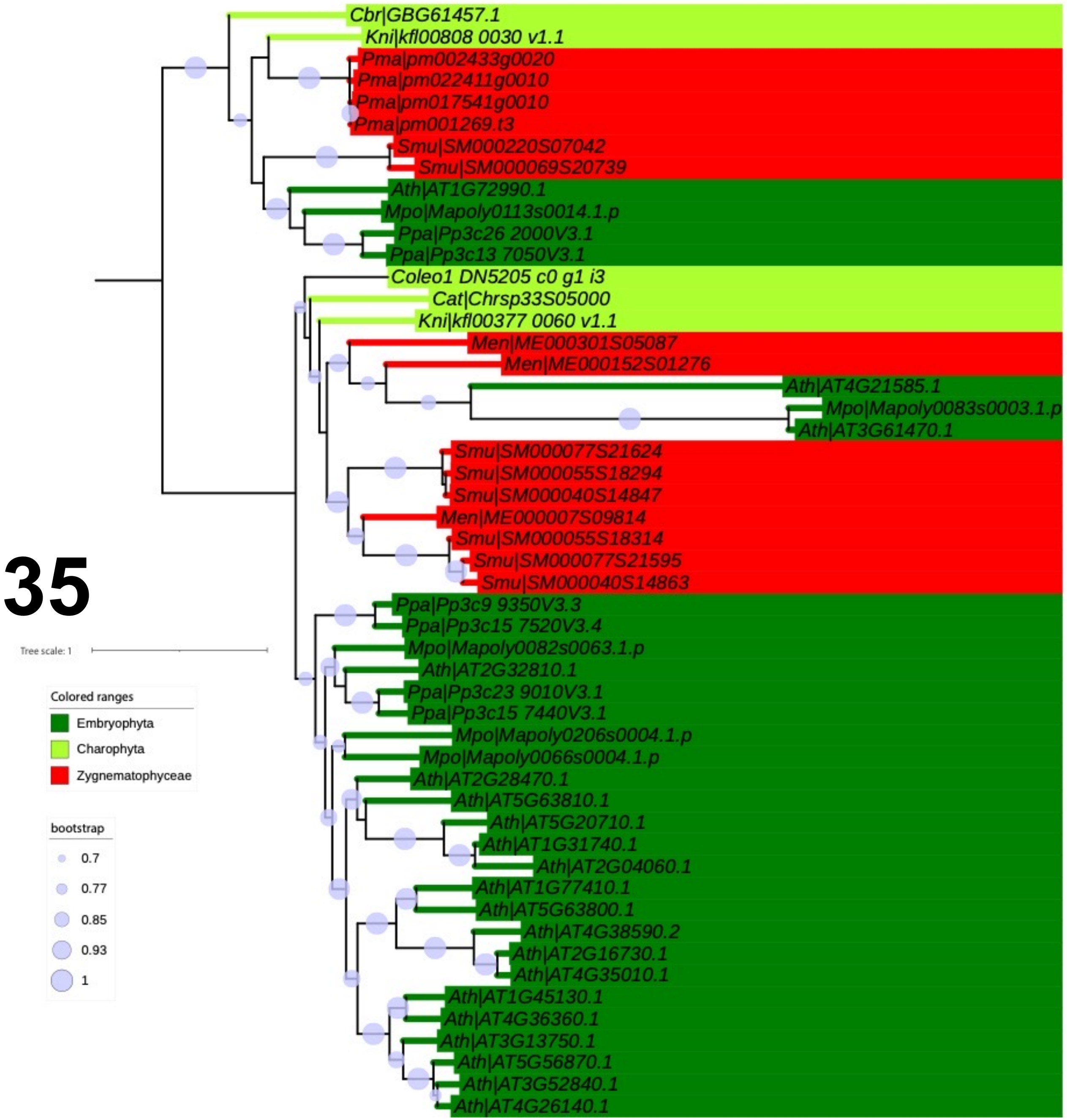

AT3G61470 (LHCA2): photosystem I light harvesting complex gene 2

AT4G21585 (ENDO4): Endonuclease 4

BGAL8 (AT3G28470)

BGAL10 (AT5g63810)

Kotake T, Dina S, Konishi T, Kaneko S, Igarashi K, Samejima M, Watanabe Y, Kimura K, Tsumuraya Y. Molecular cloning of a {beta}-galactosidase from radish that specifically hydrolyzes {beta}-(1->3)- and {beta}-(1->6)-galactosyl residues of Arabinogalactan protein. Plant Physiol. 2005 Jul;138(3):1563-76. doi: 10.1104/pp.105.062562.

Sampedro et al. AtBGAL10 is the main xyloglucan β-galactosidase in Arabidopsis, and its absence results in unusual xyloglucan subunits and growth defects. Plant Physiol. 2012 Mar;158(3):1146-57. doi: 10.1104/pp.111.192195.

Data S1-56

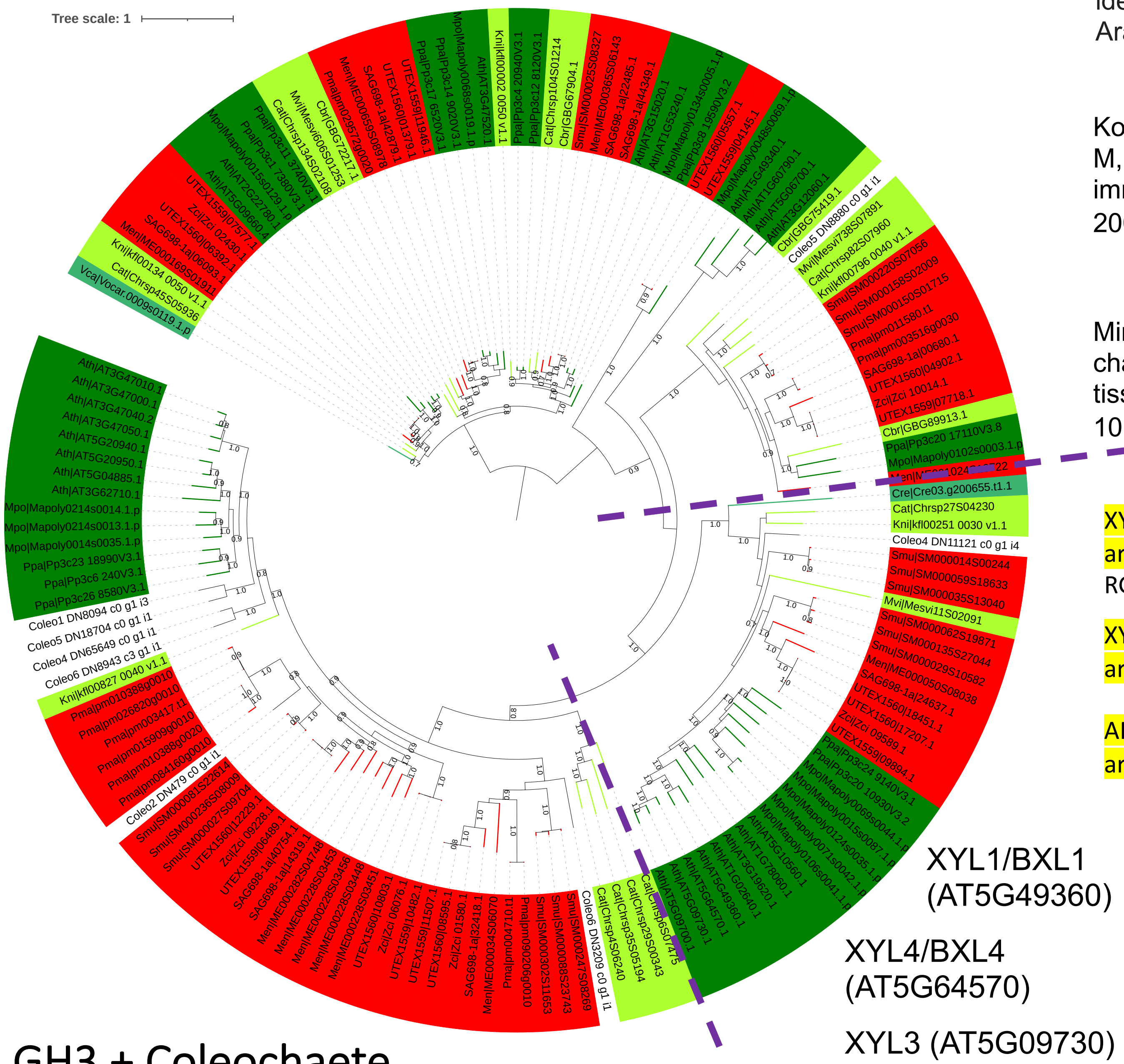

GH3 + Coleochaete

Minic et al. Purification, functional characterization, cloning, and identification of mutants of a seed-specific arabinan hydrolase in Arabidopsis. J Exp Bot. 2006;57(10):2339-51. doi: 10.1093/jxb/erj205.

Kotake T, Tsuchiya K, Aohara T, Konishi T, Kaneko S, Igarashi K, Samejima M, Tsumuraya Y. An alpha-L-arabinofuranosidase/beta-D-xylosidase from immature seeds of radish (Raphanus sativus L.). J Exp Bot. 2006;57(10):2353-62. doi: 10.1093/jxb/erj206.

Minic Z, Rihouey C, Do CT, Lerouge P, Jouanin L. Purification and characterization of enzymes exhibiting beta-D-xylosidase activities in stem tissues of Arabidopsis. Plant Physiol. 2004 Jun;135(2):867-78. doi: 10.1104/pp.104.041269.

XYL3: releases L-arabinose from (1-->5)-alpha-L-arabinofuranobiose, arabinoxylan, sugar beet arabinan, and debranched arabinan.
RG-I side chain degradation

XYL4: mainly D-Xyl from oat spelt xylan, rye arabinoxylan, wheat arabinoxylan, and oligoarabinoxylans.

ARAf and XYL1: also release D-Xyl; can further release L-Ara from arabinoxylans and arabinan

AtBXL1: putative bifunctional beta-d-xylosidase/alpha-l-arabinofuranosidase; implicated as a beta-d-xylosidase in vascular development.
GH3: hydrolytic activity on AGPs, pectic α-1,5-arabinan and arabinoxylan.

RsAraf1 from R. sativus immature seeds encodes an α-arabinofuranosidase in the GH3 family. When expressed in Arabidopsis, RsAraf1 hydrolyzed α-arabinofuranosyl residues of AGPs (Kotake et al., 2006)

Data S1-57

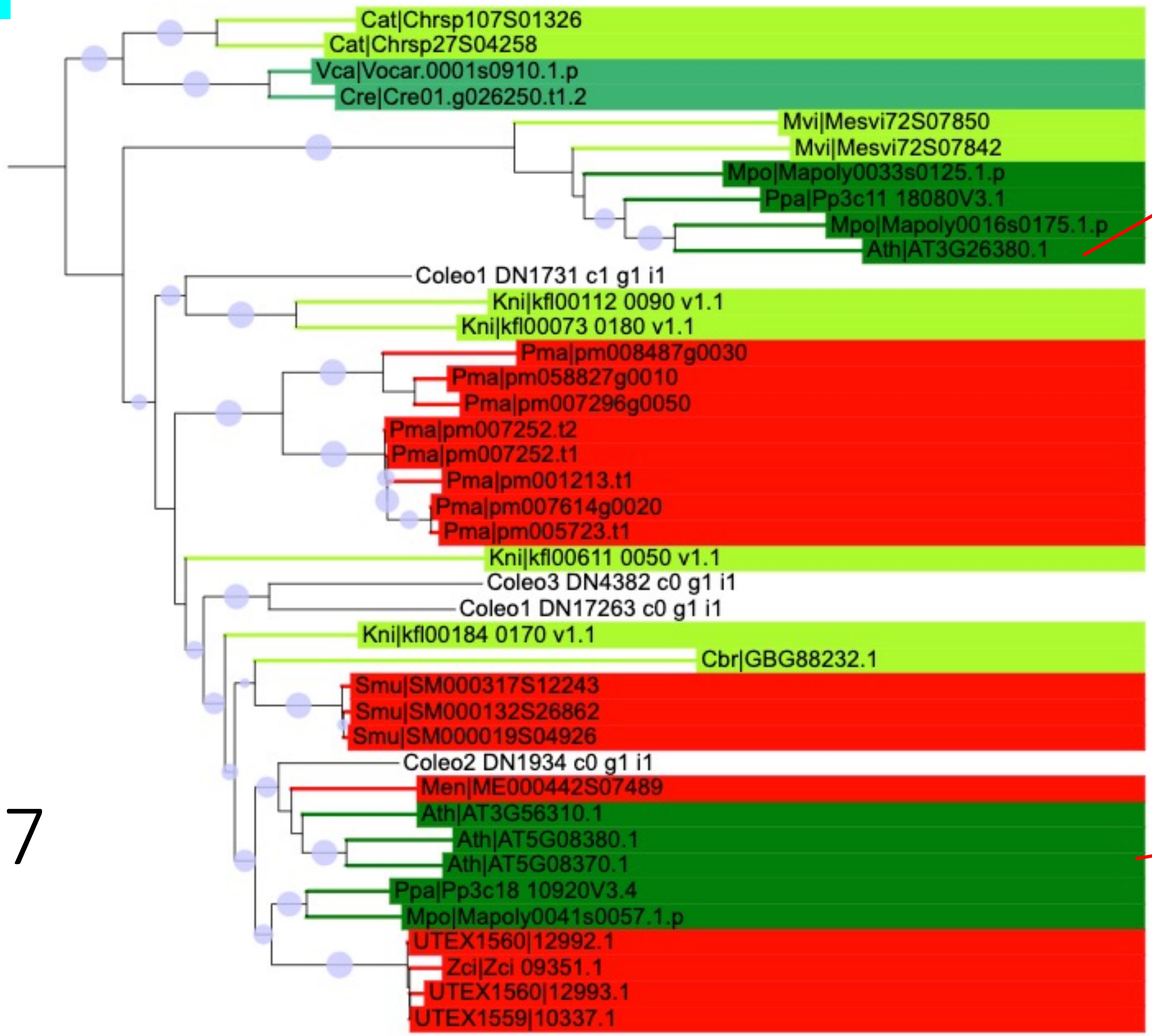

GH27

APSE (AT3G26380)

APSE and AGALs: contributions in hydrolysis of β-l-Arap residues of pectic α-1,3:1,5-arabinan & type I AG.

In Arabidopsis AGPs: β-l-Arap residues are hydrolysed mainly by APSE and partially by AGALs.

Imaizumi et al. Heterologous expression and characterization of an Arabidopsis β-l-arabinopyranosidase and α-d-galactosidases acting on β-l-arabinopyranosyl residues. J Exp Bot. 2017 Jul 20;68(16):4651-4661. doi: 10.1093/jxb/erx279.

AGAL1 (AT5G08380)
AGAL2 (AT5G08370)
AGAL3 (AT3G56310)

https://itol.embl.de/tree/208127240222424791665201864

Data S1-58

APSE: HGT from bacteria

GH27 nr

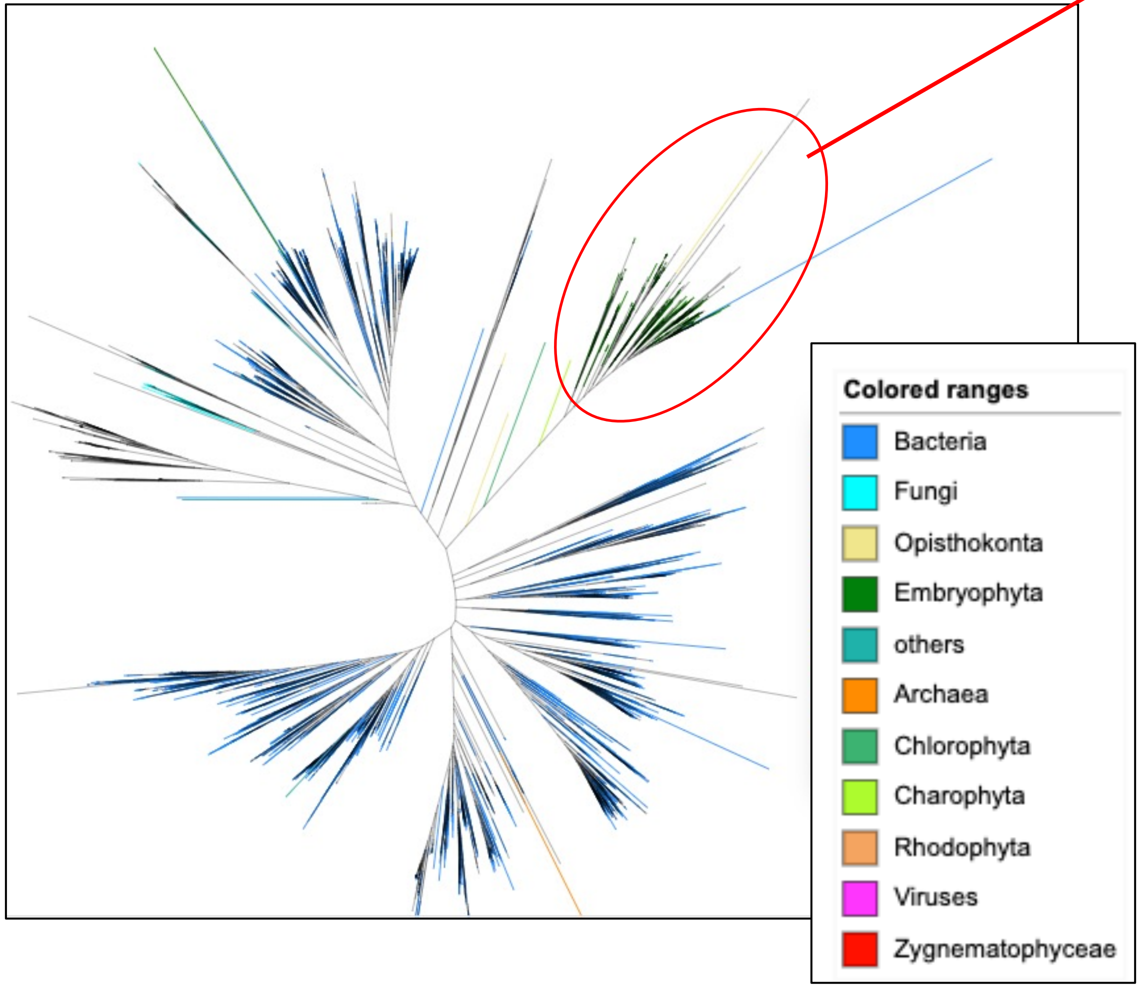

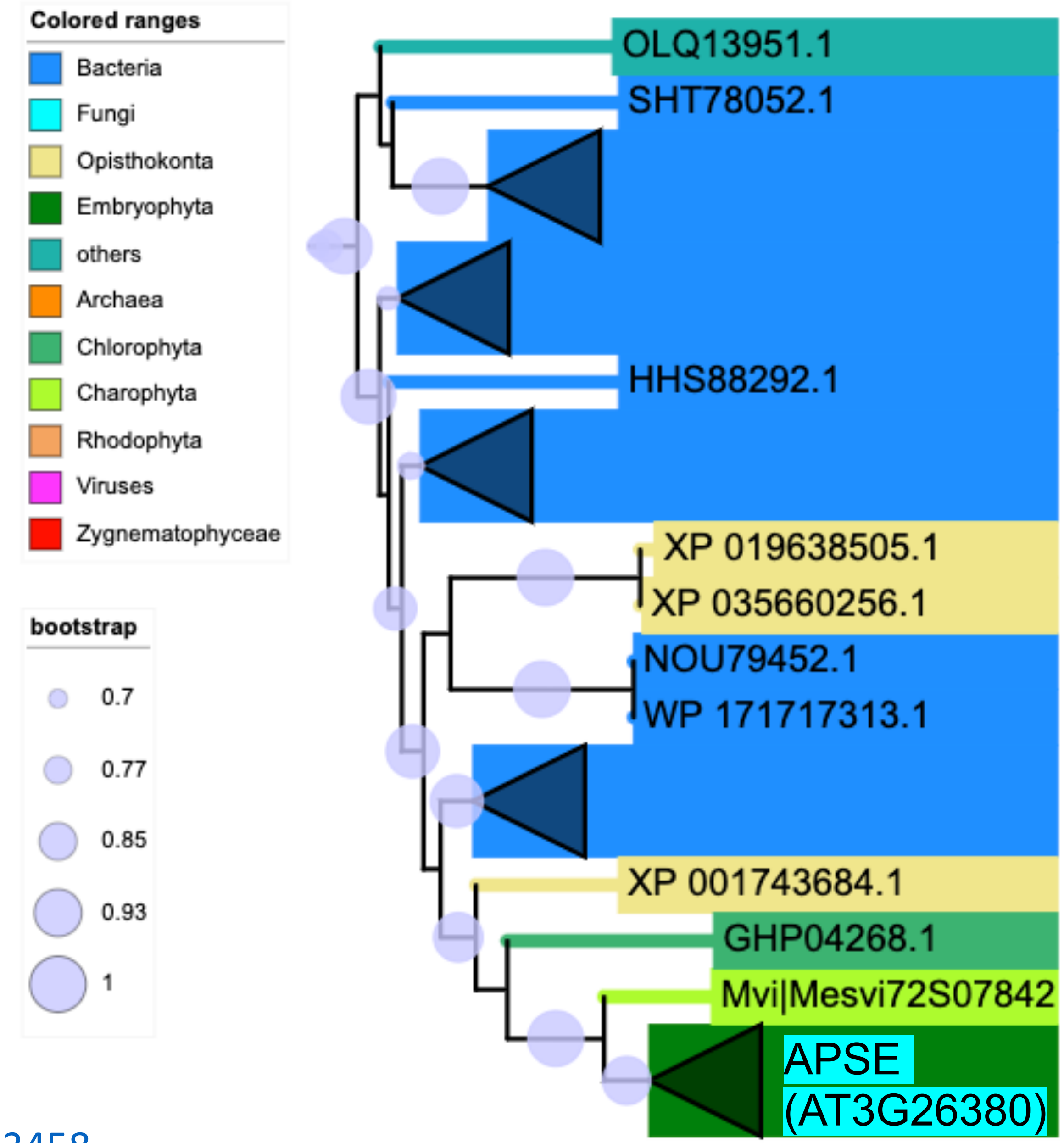

https://itol.embl.de/tree/20812724022256331665203458

Data S1-59

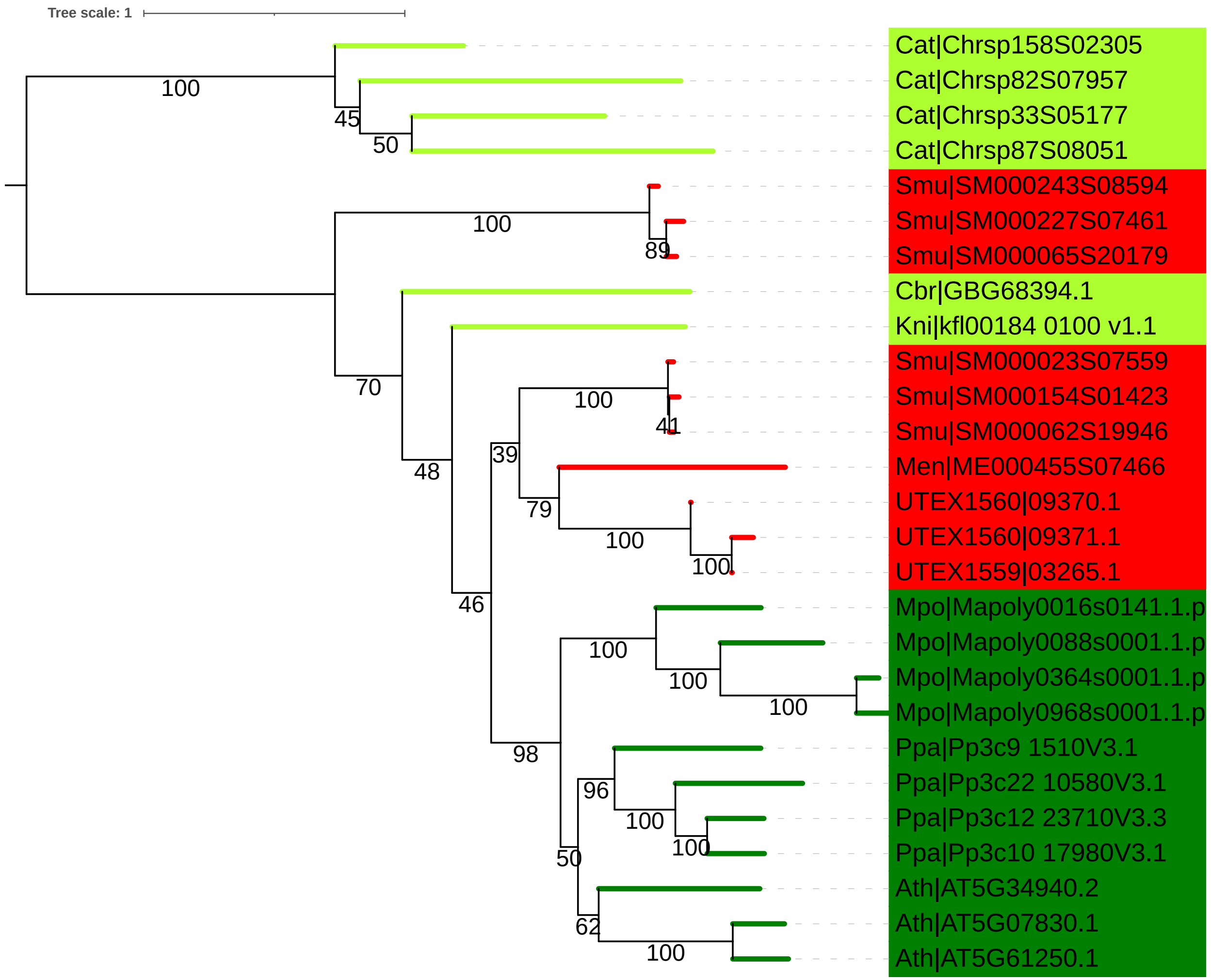

GH79

Eudes, A., Mouille, G., Thévenin, J., Goyallon, A., Minic, Z., and Jouanin, L. (2008). Purification, cloning and functional characterization of an endogenous beta-glucuronidase in Arabidopsis thaliana. Plant Cell Physiol. 49, 1331–1341. doi: 10.1093/pcp/pcn108

AtGUS1 (AT5G61250)

AtGUS2 (AT5G07830)

AtGUS3 (AT5G34940)

No GH79 was found in the Coleochaete transcriptome sequences.

Data S1-60

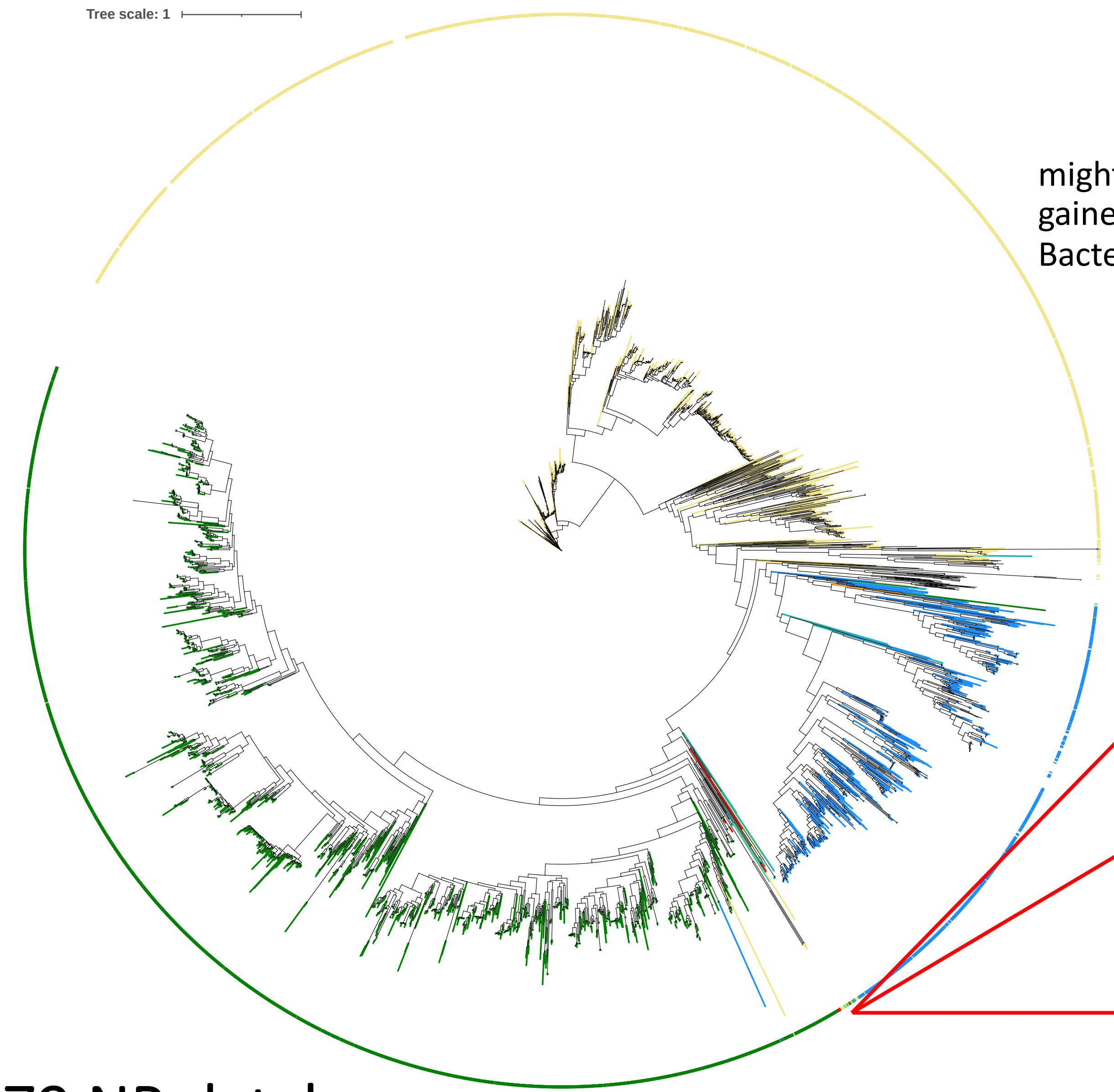

might have been gained via HGT from Bacteria

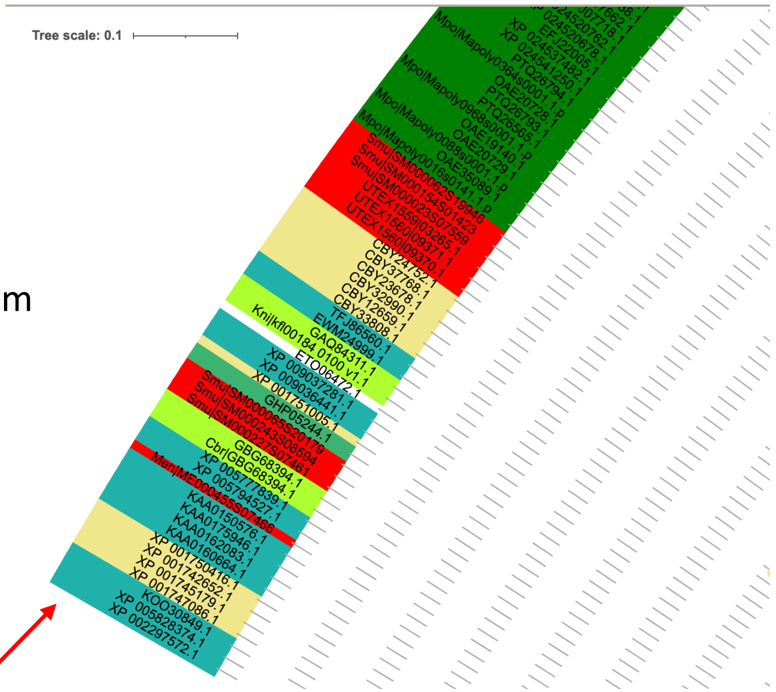

Zynema
(UTEX1559, UTEX1560)
Klebsormidium
Chara

Amorphea (Monosiga brevicollis)
Animalia (Oikopleura dioica)
SAR (Nannochloropsis salina, Aureococcus anophagefferens)
Chlorophyta (Pycnococcus provasolii)

GH79 NR database

Data S1-61

Homogalacturonan (HG)

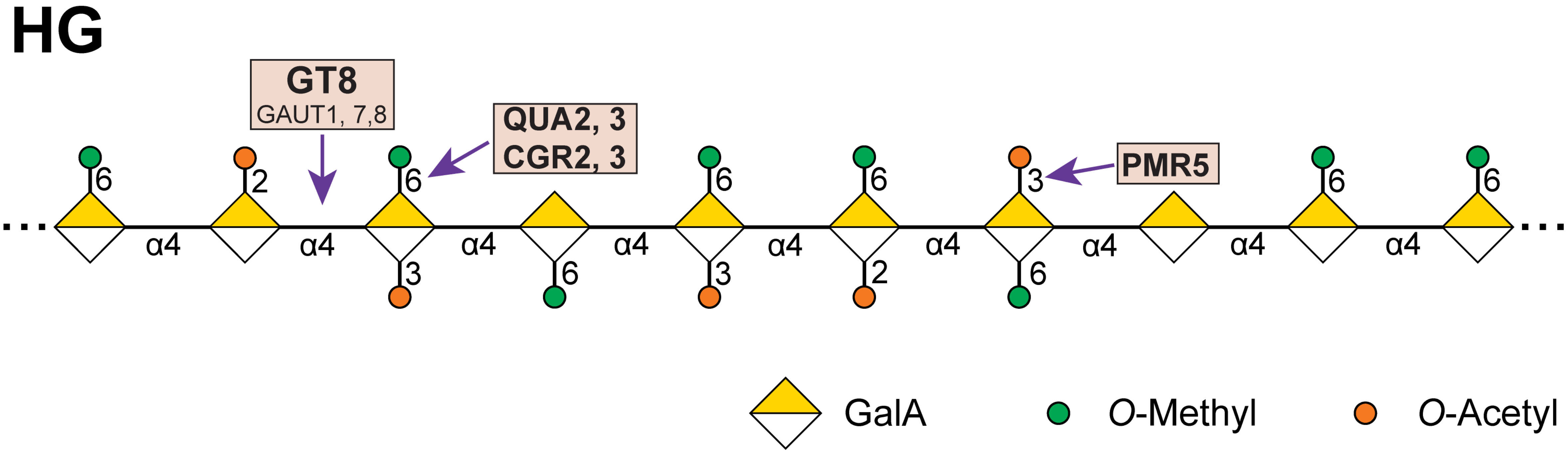

Data S1-62

GALACTURONOSYL- TRANSFERASE (GAUT)

https://itol.embl.de/tree/2081272402226352166535241 5

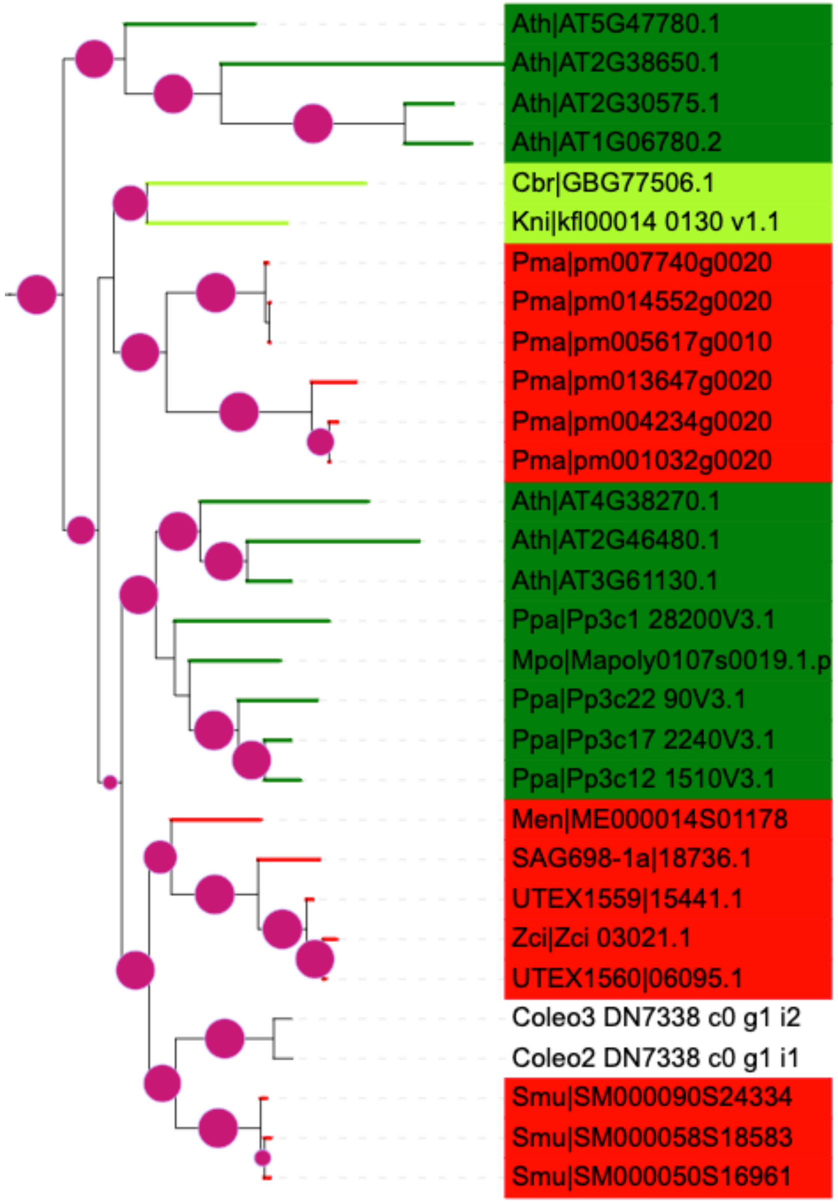

GAUT6 (AT1G06780)

GAUT5 (AT2G30575)

GAUT7 (AT2G38650)

GAUT4 (AT5G47780)

GAUT1 (AT3G61130)

GAUT2 (AT2G46480)

GAUT3 (AT4G38270)

Atmodjo et al. Galacturonosyltransferase (GAUT)1 and GAUT7 are the core of a plant cell wall pectin biosynthetic homogalacturonan:galacturonosyltransferase complex. Proc Natl Acad Sci U S A. 2011 Dec 13;108(50):20225-30. doi: 10.1073/pnas.1112816108.

Sterling et al. Functional identification of an Arabidopsis pectin biosynthetic homogalacturonan galacturonosyltransferase. Proc Natl Acad Sci U S A. 2006 Mar 28;103(13):5236-41. doi: 10.1073/pnas.0600120103.

Bouton et al. QUASIMODO1 encodes a putative membrane-bound glycosyltransferase required for normal pectin synthesis and cell adhesion in Arabidopsis. Plant Cell. 2002 Oct;14(10):2577-90. doi: 10.1105/tpc.004259.

GAUT8 (QUA1, AT3G25140)

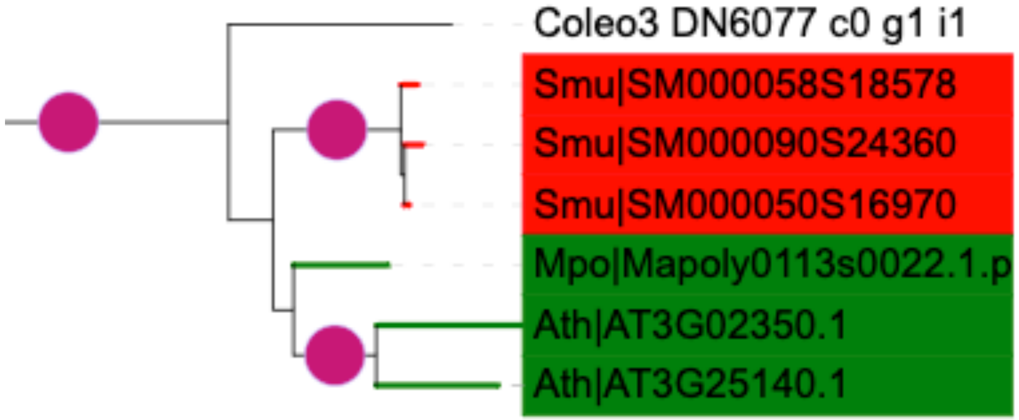

Data S1-63

QUA2 (AT1G78240)

Blast with 16genome + Coleochaete

HG: methylesterified by QUA2, QUA3 and GGR3

QUA2 might have been present in the LCA of Phragmoplastophyta

QUA3 (AT4G00740) might have first emerged in the LCA of Zygnematophyceae and Embryophyta

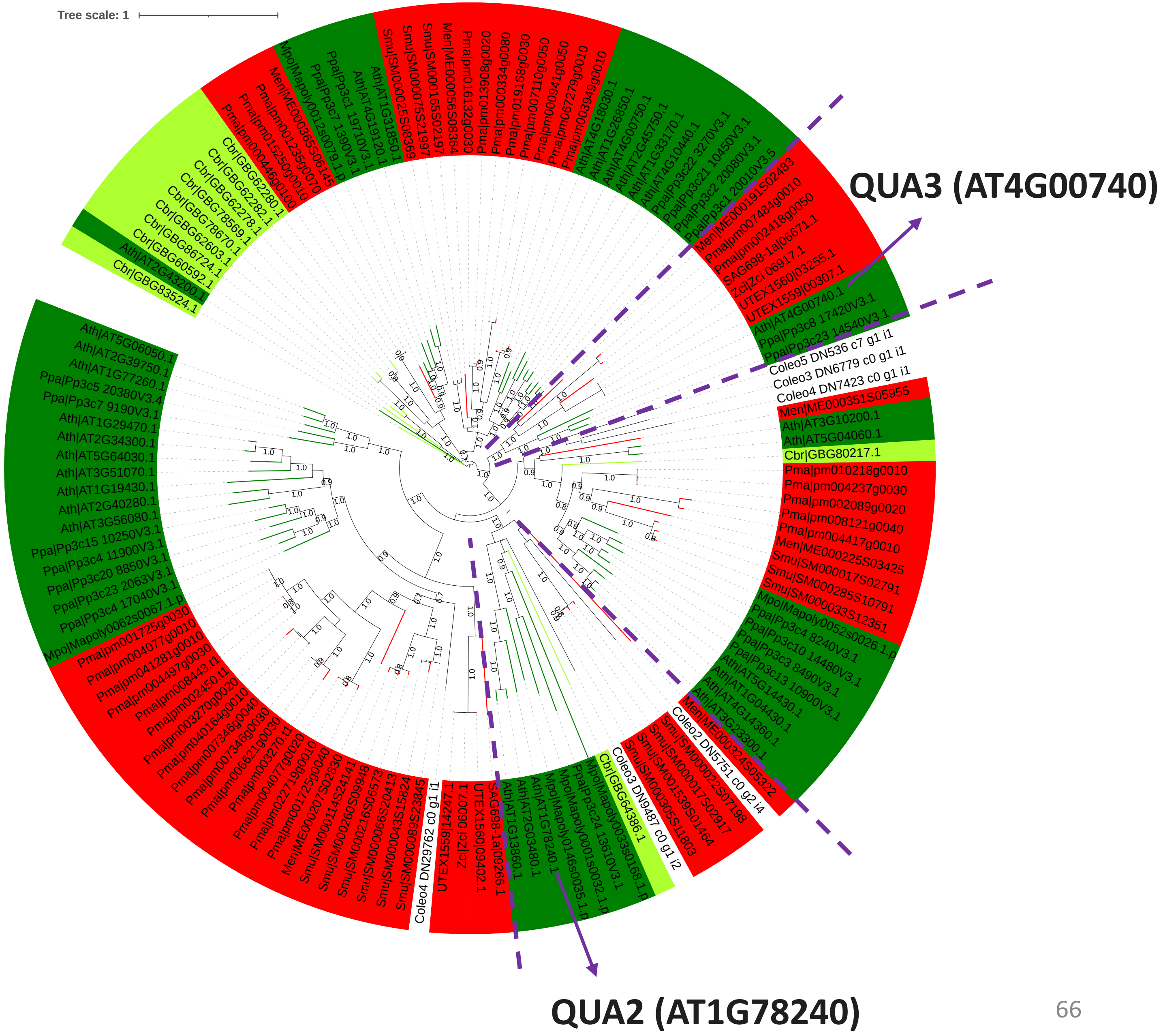

Data S1-64

Kim SJ, Held MA, Zemelis S, Wilkerson C, Brandizzi F. CGR2 and CGR3 have critical overlapping roles in pectin methylesterification and plant growth in Arabidopsis thaliana. Plant J. 2015 Apr;82(2):208-20. doi: 10.1111/tpj.12802.

CGR2/3 homologs (evalue<1e-15)

```
Ath|AT5G65810.1 CGR2|AT3G49720  86.590  261  32   1   1   258  1    261  1.59e-174  469
Ath|AT3G49720.1 CGR2|AT3G49720  100.000 261  0    0   1   261  1    261  0.0  534
Cbr|GBG71667.1  CGR2|AT3G49720  30.075  133  91   2   60  190  94   226  2.26e-15  64.7
Mpo|Mapoly0082s0061.1.p CGR2|AT3G49720  34.201  269  167  6   40  306  1    261  6.71e-49  151
Mvi|Mesvi21S04311       CGR2|AT3G49720  27.174  184  119  8   70  243  48   226  1.58e-15  61.6
Ppa|Pp3c12_13990V3.1    CGR2|AT3G49720  36.397  272  128  6   31  298  27   257  1.54e-55  168
Coleo1_DN62522_c0_g1_i1 CGR2|AT3G49720  29.487  78   55   0   12  89   149  226  1.55e-13  50.4
```

```
Ath|AT5G65810.1 GGR3|AT5G65810  100.000 258  0    0   1   258  1    258  0.0  529
Ath|AT3G49720.1 GGR3|AT5G65810  86.590  261  32   1   1   261  1    258  6.63e-174  469
Cbr|GBG71667.1  GGR3|AT5G65810  29.927  137  94   2   56  190  87   223  2.20e-14  63.5
Mpo|Mapoly0082s0061.1.p GGR3|AT5G65810  35.124  242  148  4   67  306  24   258  3.60e-49  153
Mvi|Mesvi21S04311       GGR3|AT5G65810  30.827  133  89   3   114 243  91   223  1.86e-13  57.0
Ppa|Pp3c12_13990V3.1    GGR3|AT5G65810  36.765  272  127  6   31  298  24   254  1.59e-59  180
Coleo1_DN62522_c0_g1_i1 GGR3|AT5G65810  29.487  78   55   0   12  89   146  223  8.25e-12  47.4
```

Data S1-65

PMR5 (AT5G58600)

BLAST with 16 genomes

+ Coleochaete

PMR5 (AT5G58600) might have been present in the LCA of Phragmoplastophyta

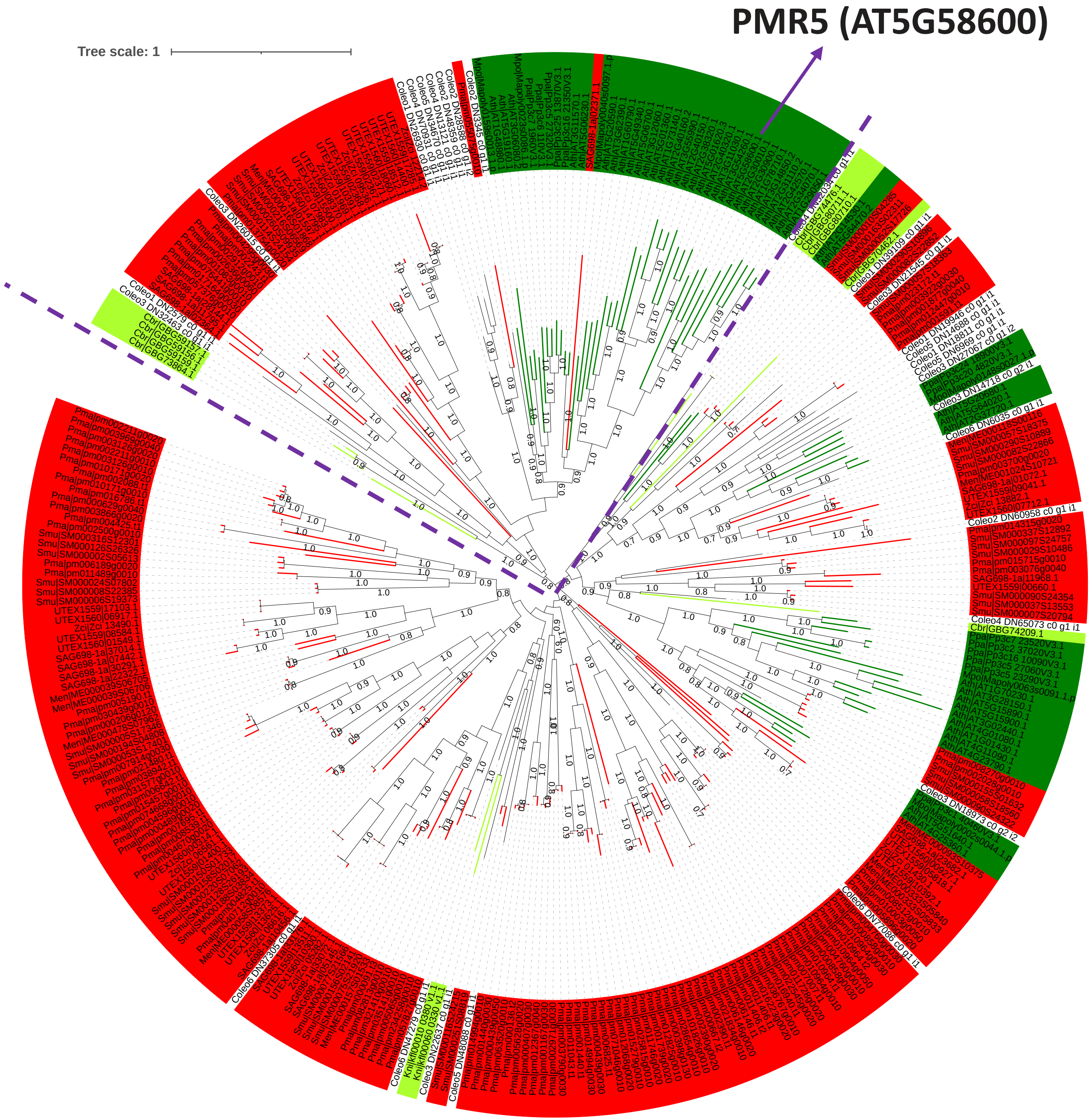

Chiniquy D, Underwood W, Corwin J, Ryan A, Szemenyei H, Lim CC, Stonebloom SH, Birdseye DS, Vogel J, Kliebenstein D, Scheller HV, Somerville S. PMR5, an acetylation protein at the intersection of pectin biosynthesis and defense against fungal pathogens. Plant J. 2019 Dec;100(5):1022-1035. doi: 10.1111/tpj.14497.

Data S1-66

https://itol.embl.de/tree/13423816812456541668314644

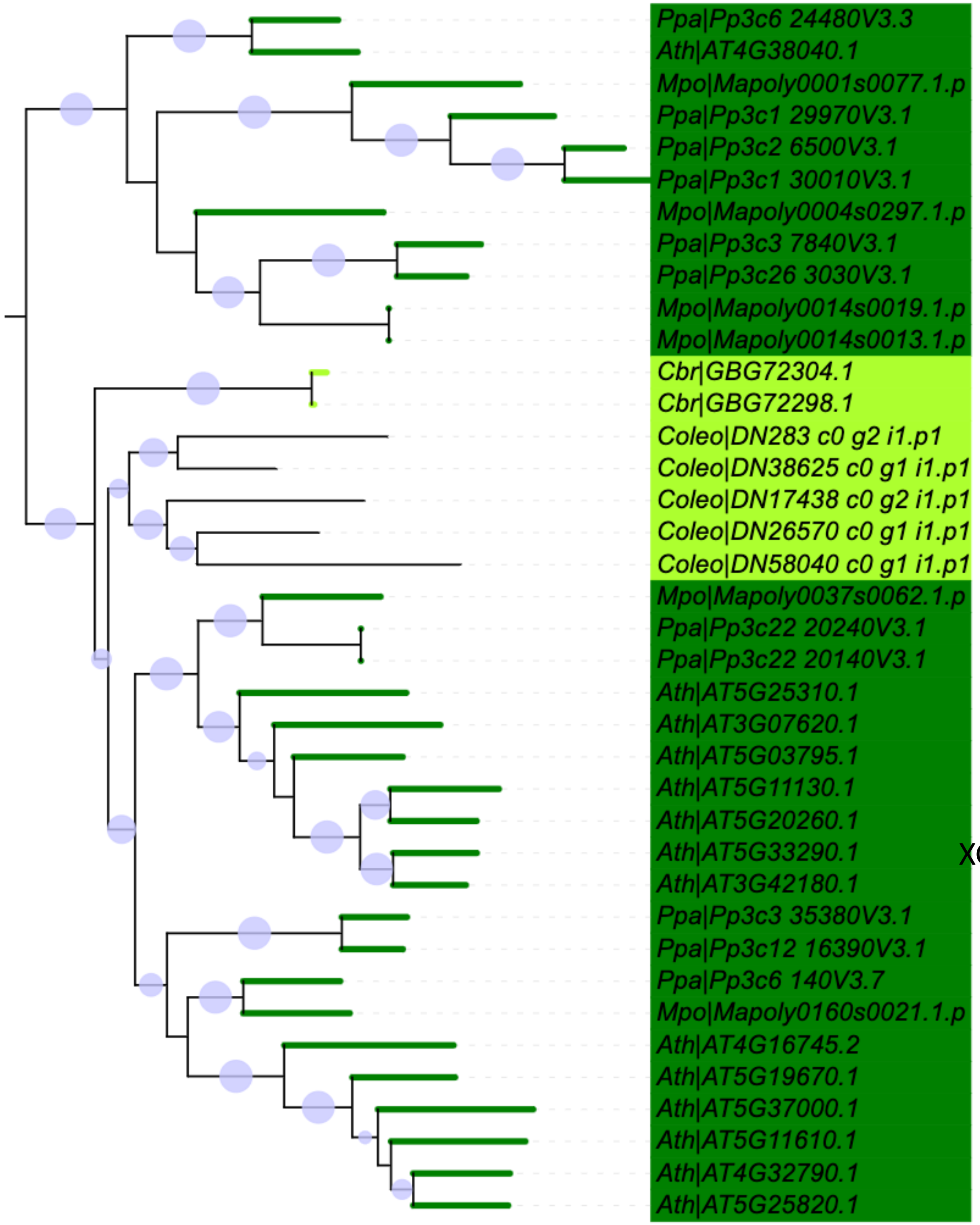

GT47 (Data S1-33)

Jensen et al. Identification of a xylogalacturonan xylosyltransferase involved in pectin biosynthesis in Arabidopsis. Plant Cell. 2008 May;20(5):1289-302. doi: 10.1105/tpc.107.050906

XGD1 (AT5G33290)

XGA

Data S1-67

https://itol.embl.de/tree/1342381641713026616669281511

CE8: pectin methylesterase

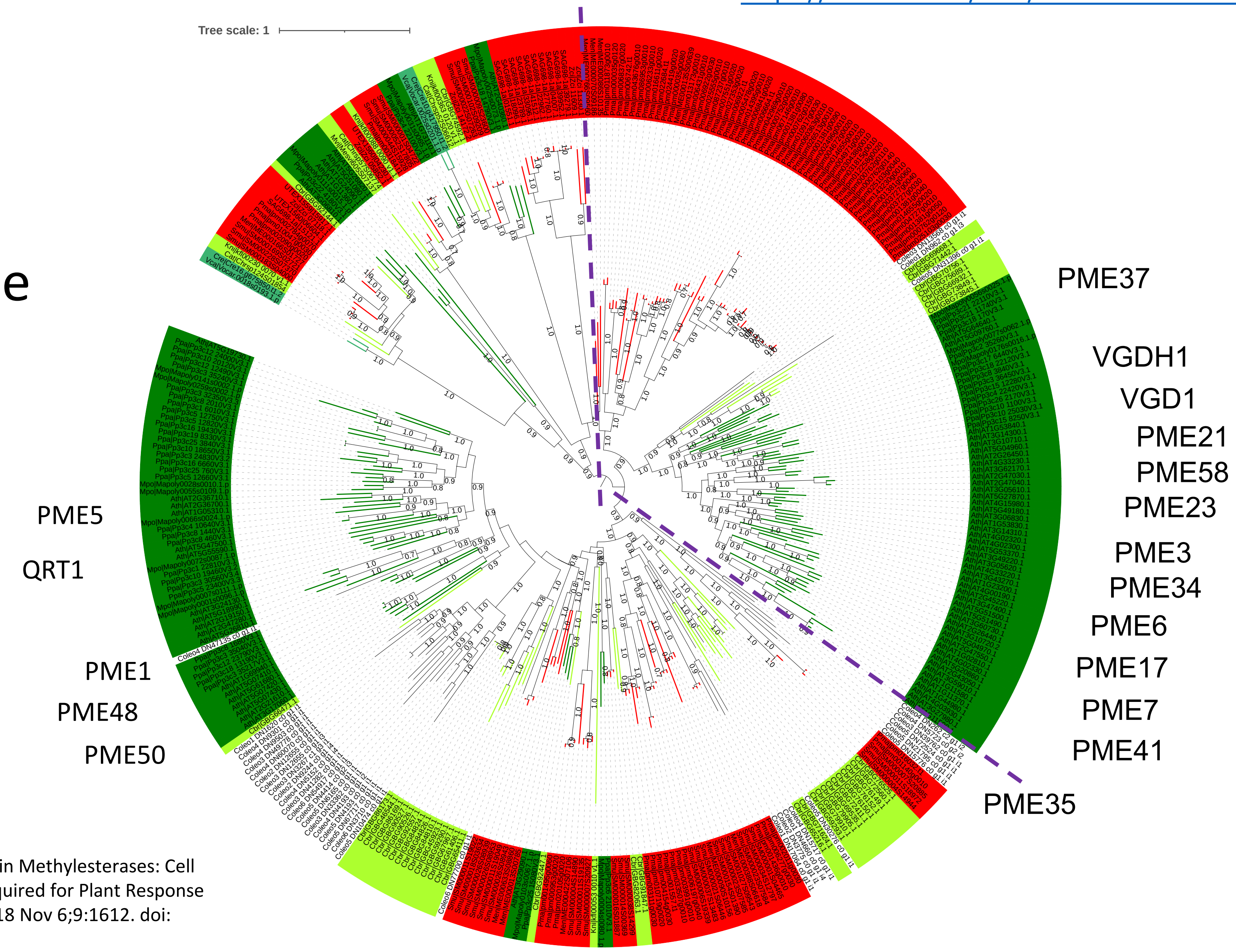

Wu HC, Bulgakov VP, Jinn TL. Pectin Methylesterases: Cell Wall Remodeling Proteins Are Required for Plant Response to Heat Stress. Front Plant Sci. 2018 Nov 6;9:1612. doi: 10.3389/fpls.2018.01612.

Data S1-68

CE13: pectin acetylesterase

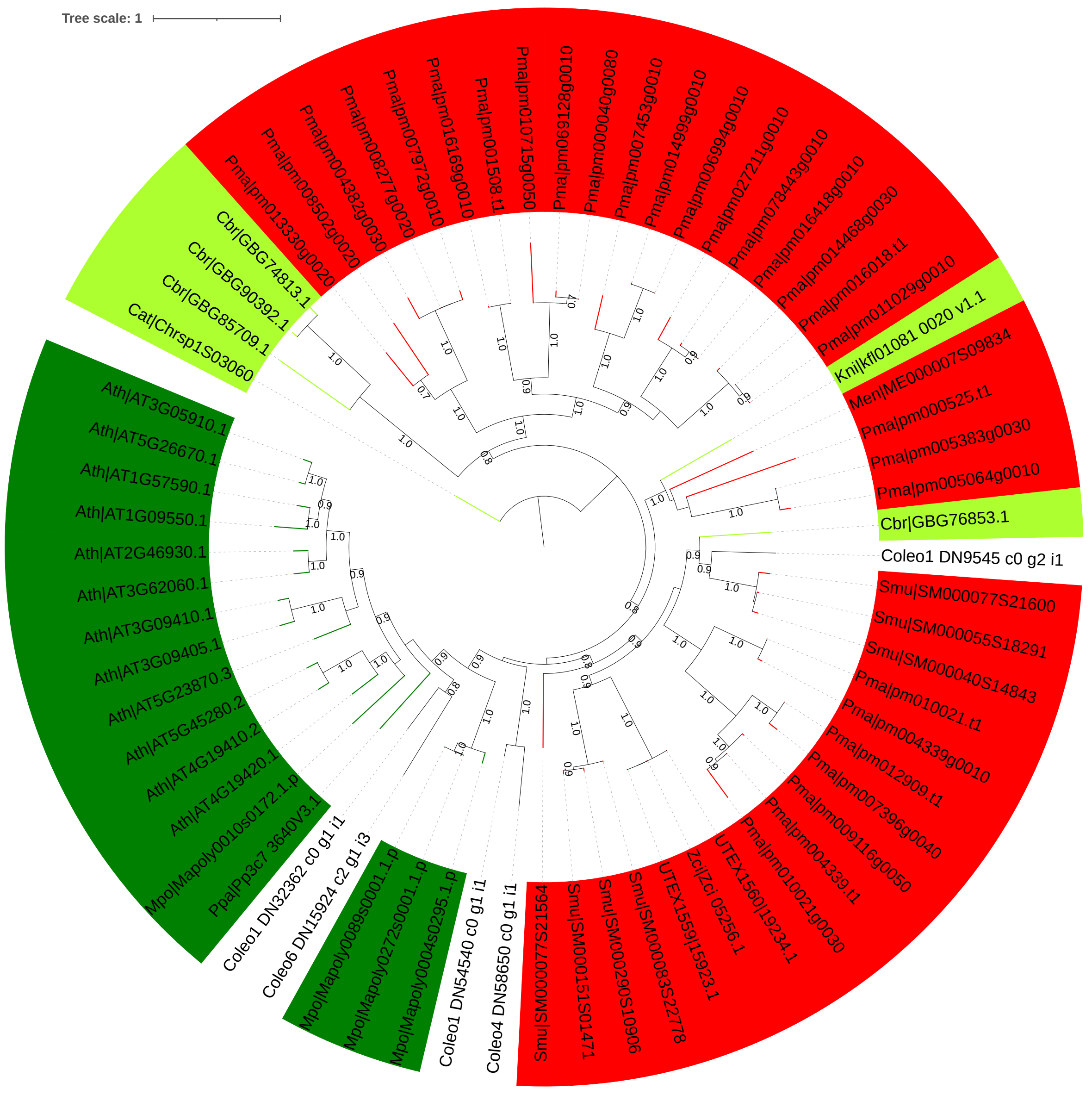

Philippe F, Pelloux J, Rayon C. Plant pectin acetylesterase structure and function: new insights from bioinformatic analysis. BMC Genomics. 2017 Jun 8;18(1):456. doi: 10.1186/s12864-017-3833-0.

Data S1-69

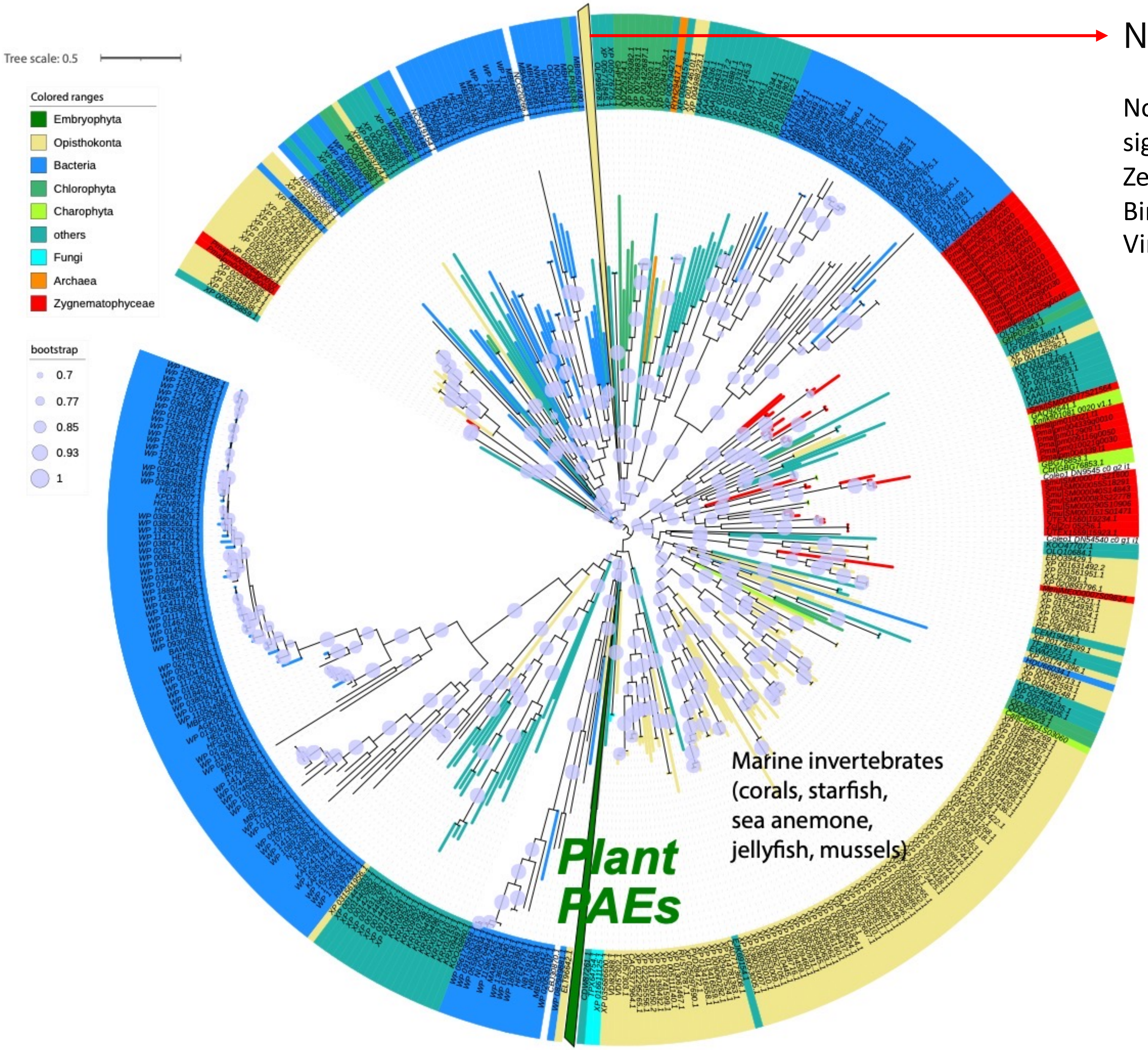

Notum from animals

Notum deacylates Wnt proteins to suppress signalling activity. Kakugawa S, Langton PF, Zebisch M, Howell SA, Chang TH, Liu Y, Feizi T, Bineva G, O'Reilly N, Snijders AP, Jones EY, Vincent JP. Nature 519, 187-92, (2015).

CE13 nr

Data S1-70

RG-I

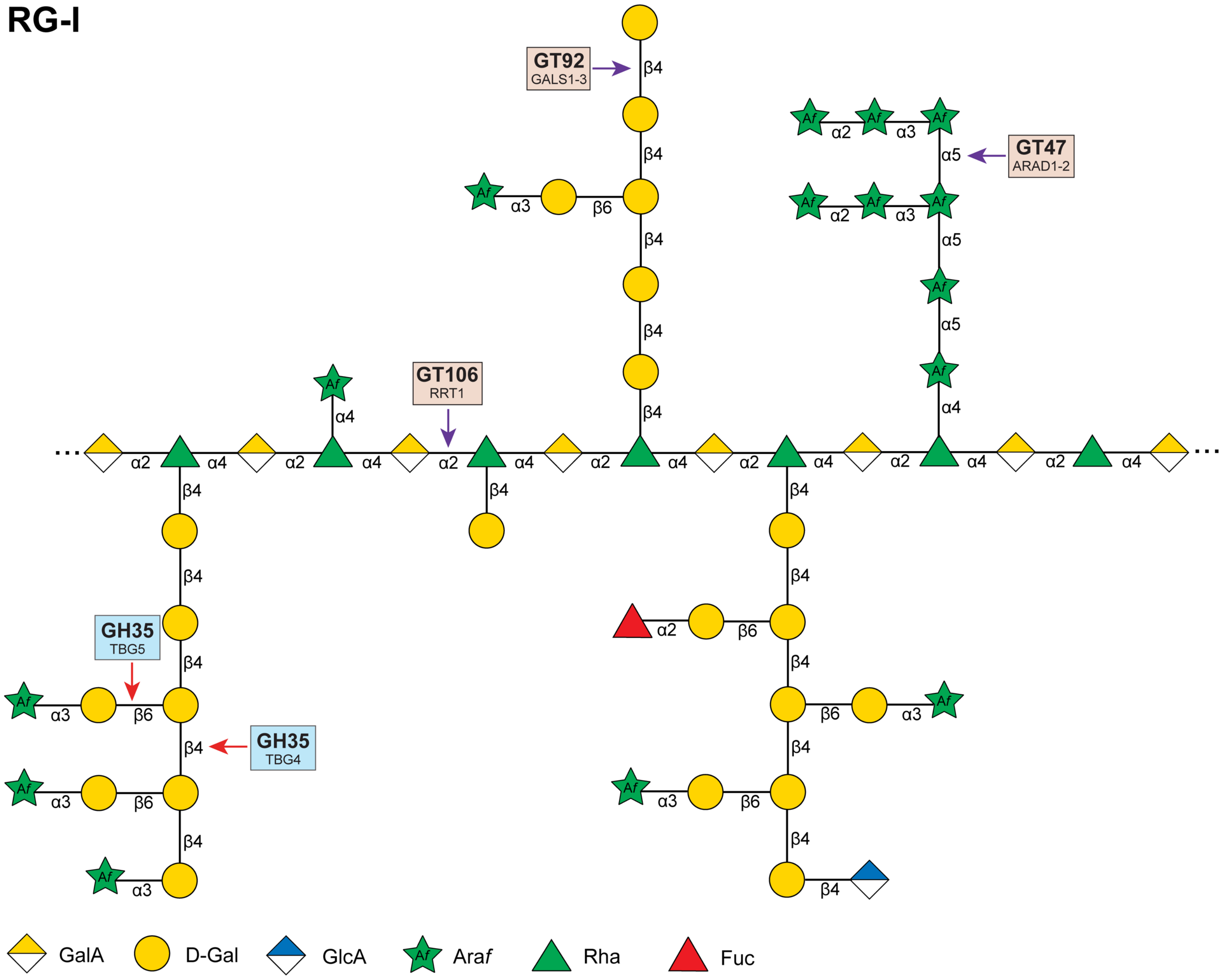

Rhamnogalacturonan I (RG-I)

- **RG-I: 20–35% pectin**
- GALACTAN SYNTHASE 1 (GALS1) of GT92: a β-1,4-galactan:β-1,4-GalT
- ARABINAN DEFICIENT 1 (ARAD1) & ARAD2: pectin arabinan biosynthesis

GT106 + Coleochaete

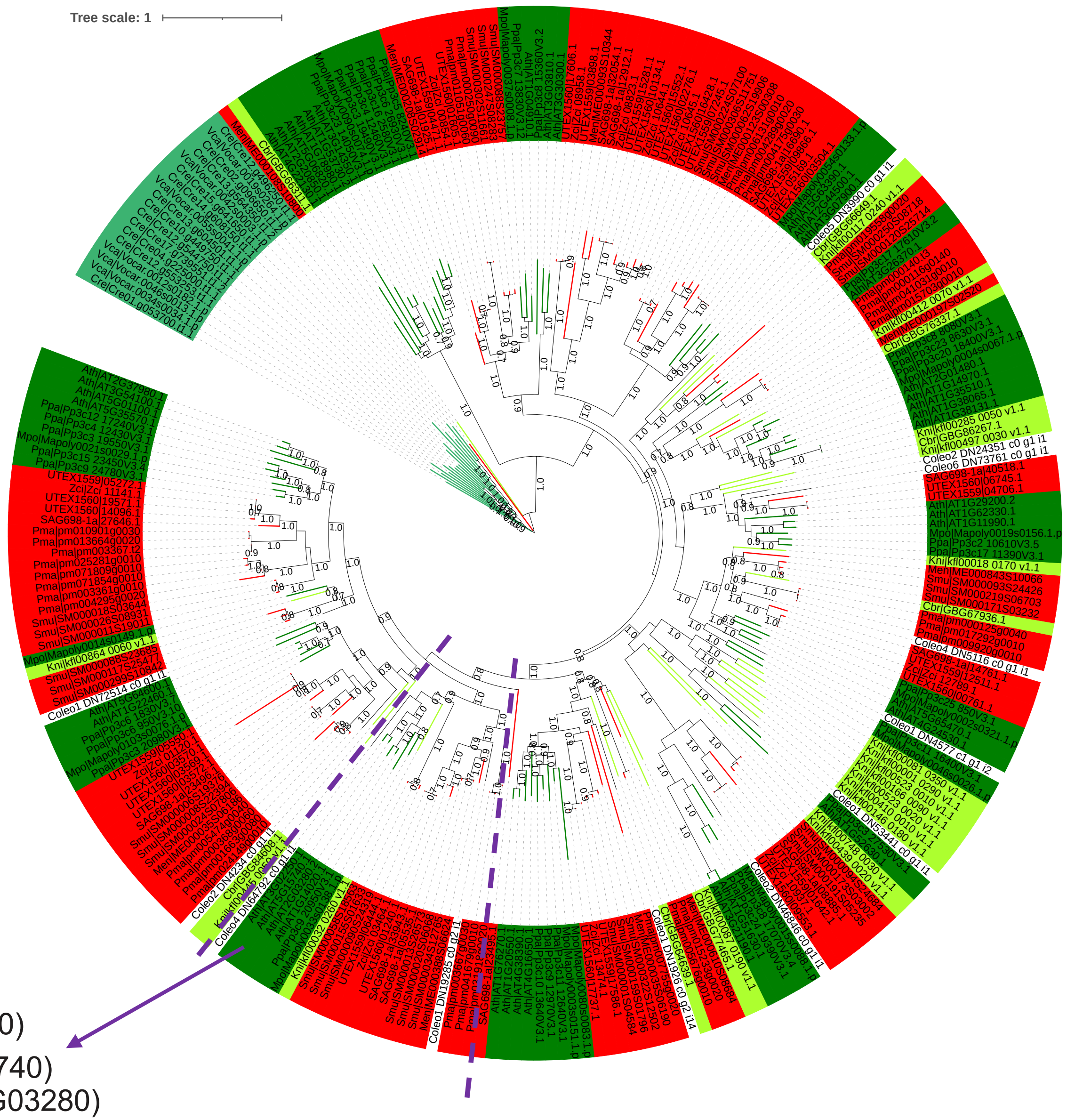

RRT2 (AT3G02250)

RRT1 (AT5G15740)
RRT3 (AT2G03280)
RRT4 (AT1G14020)

Takenaka Y, Kato K, Ogawa-Ohnishi M, Tsuruhama K, Kajiura H, Yagyu K, Takeda A, Takeda Y, Kunieda T, Hara-Nishimura I, Kuroha T, Nishitani K, Matsubayashi Y, Ishimizu T. Pectin **RG-I rhamnosyltransferases** represent a novel plant-specific glycosyltransferase family. Nat Plants. 2018 Sep;4(9):669-676. doi: 10.1038/s41477-018-0217-7.

Data S1-72

RG-I: GAT1 (GT116/DUF616)

Galaturonosyltransferase

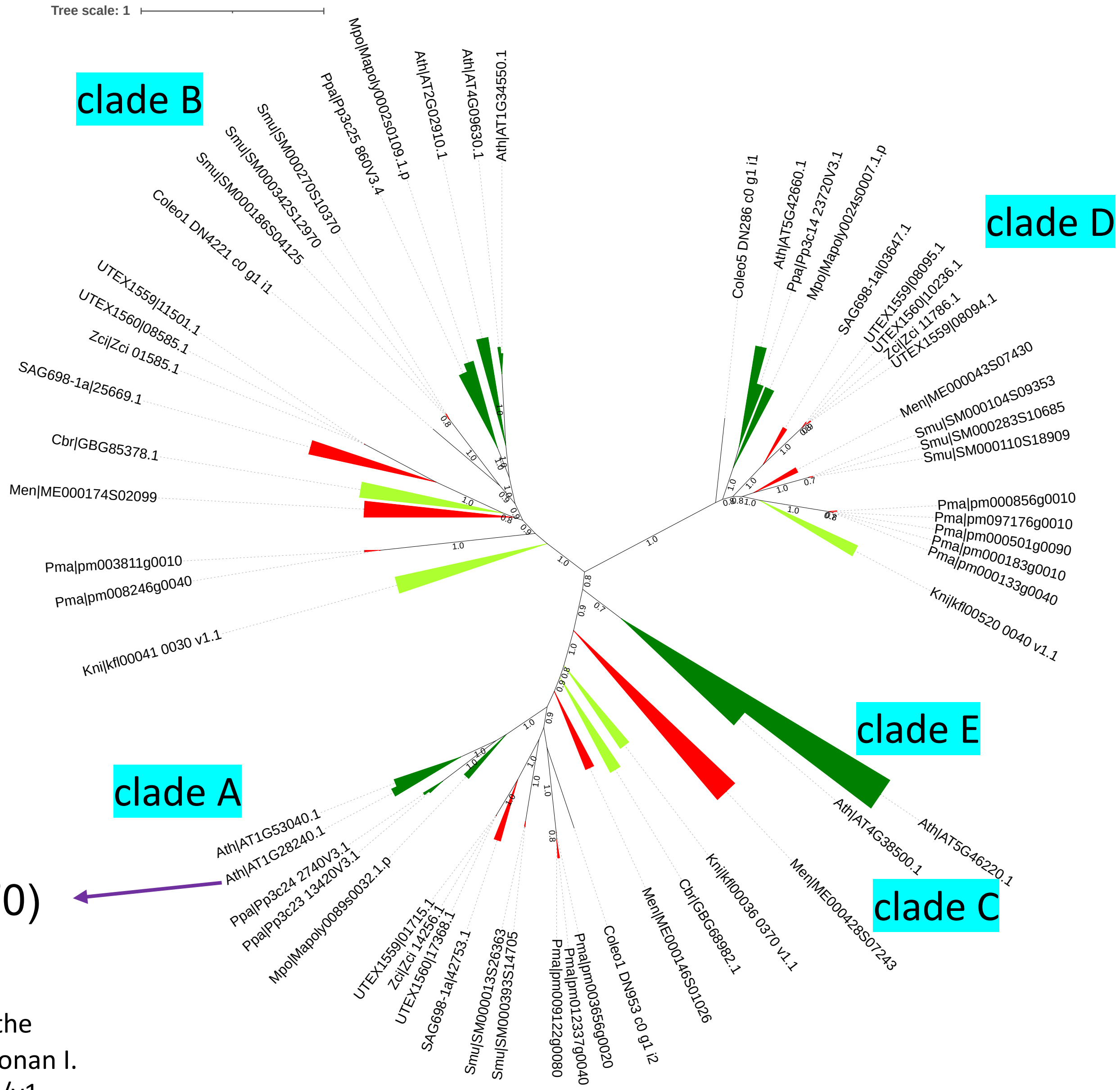

RGGAT1(AT1G28240/MUCI70)

Amos R, Atmodjo M, Huang C, et al. Polymerization of the backbone of the pectic polysaccharide rhamnogalacturonan I. Research Square; 2022. DOI: 10.21203/rs.3.rs-1475173/v1.

Data S1-73

GAT1(AT1G28240/MUCI70)

https://itol.embl.de/tree/1342381901478697166710271 3

RG-I: GAT1 (DUF616)
Galaturonosyltransferase

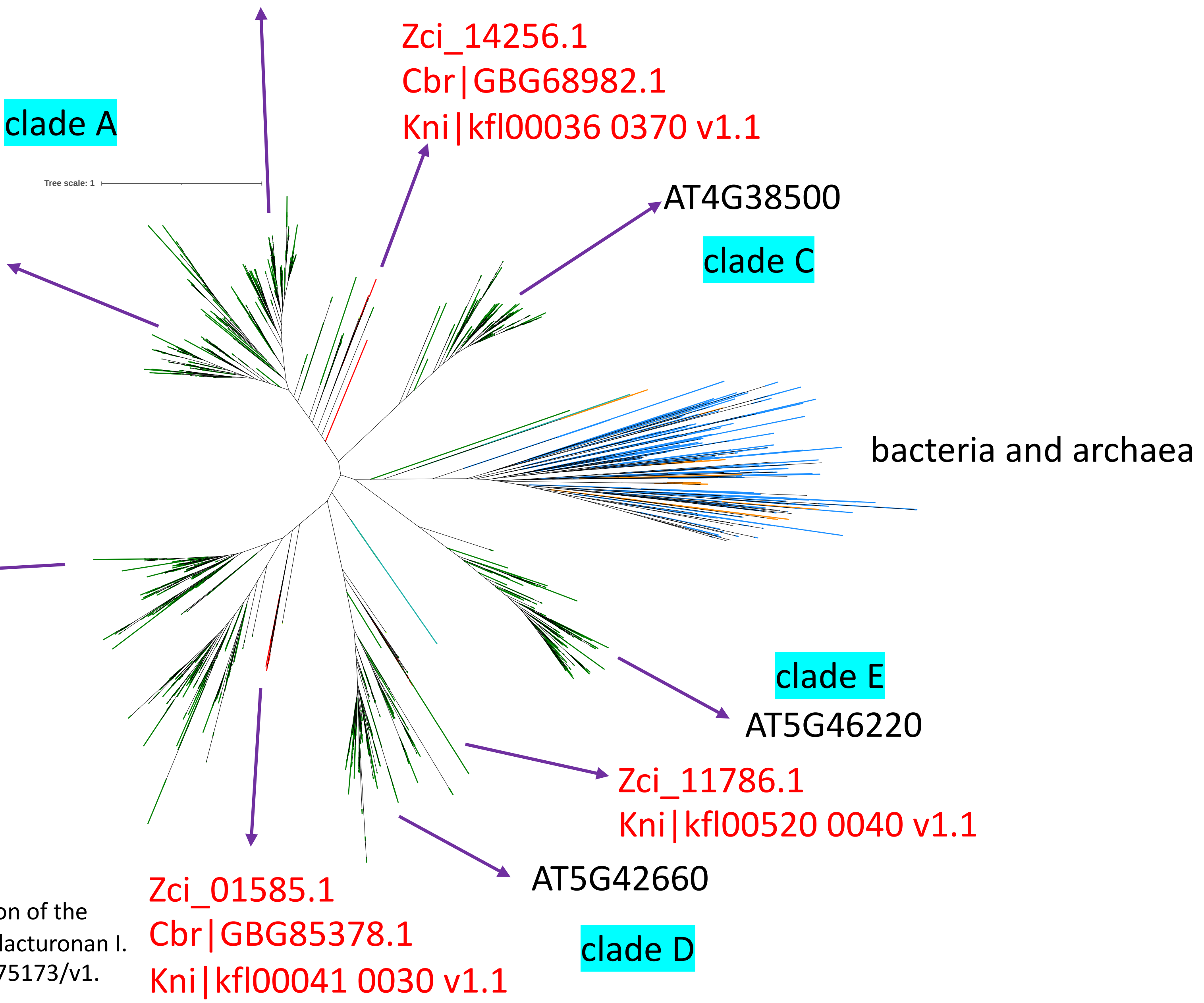

Amos R, Atmodjo M, Huang C, et al. Polymerization of the backbone of the pectic polysaccharide rhamnogalacturonan I. Research Square; 2022. DOI: 10.21203/rs.3.rs-1475173/v1.

Data S1-74

https://itol.embl.de/tree/13423816812456541668314644

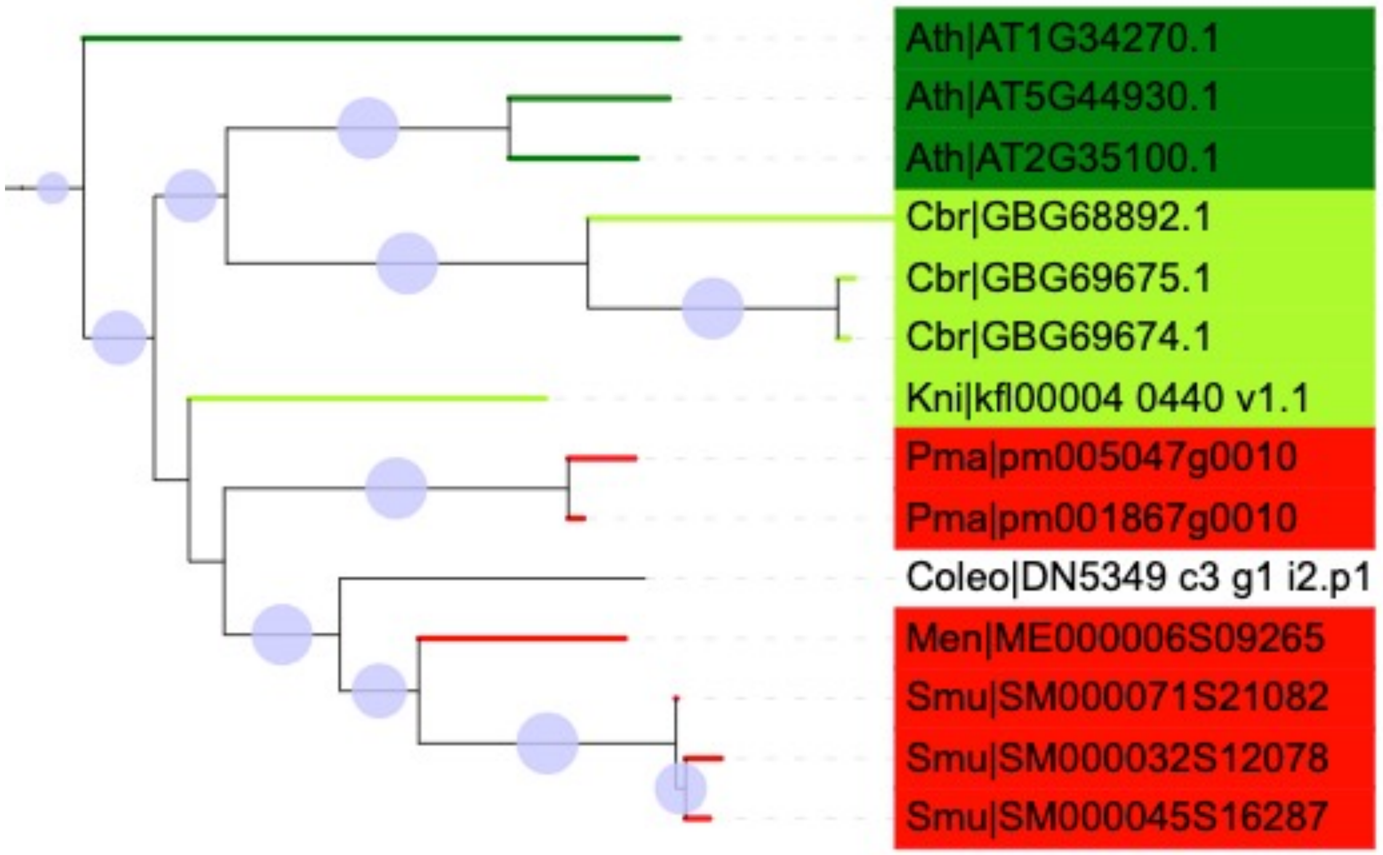

ARAD2 (AT5G44930)
ARAD1 (AT2G35100)

RG-I

Harholt et al. ARABINAN DEFICIENT 1 is a putative arabinosyltransferase involved in biosynthesis of pectic arabinan in Arabidopsis. Plant Physiol. 2006 Jan;140(1):49-58. doi: 10.1104/pp.105.072744.

GT47

Data S1-75

GT92 (PF01697)

e-value: e-10

GALS3 (AT4G20170)

GALS2 (AT5G44670)

GALS1 (AT2G33570)

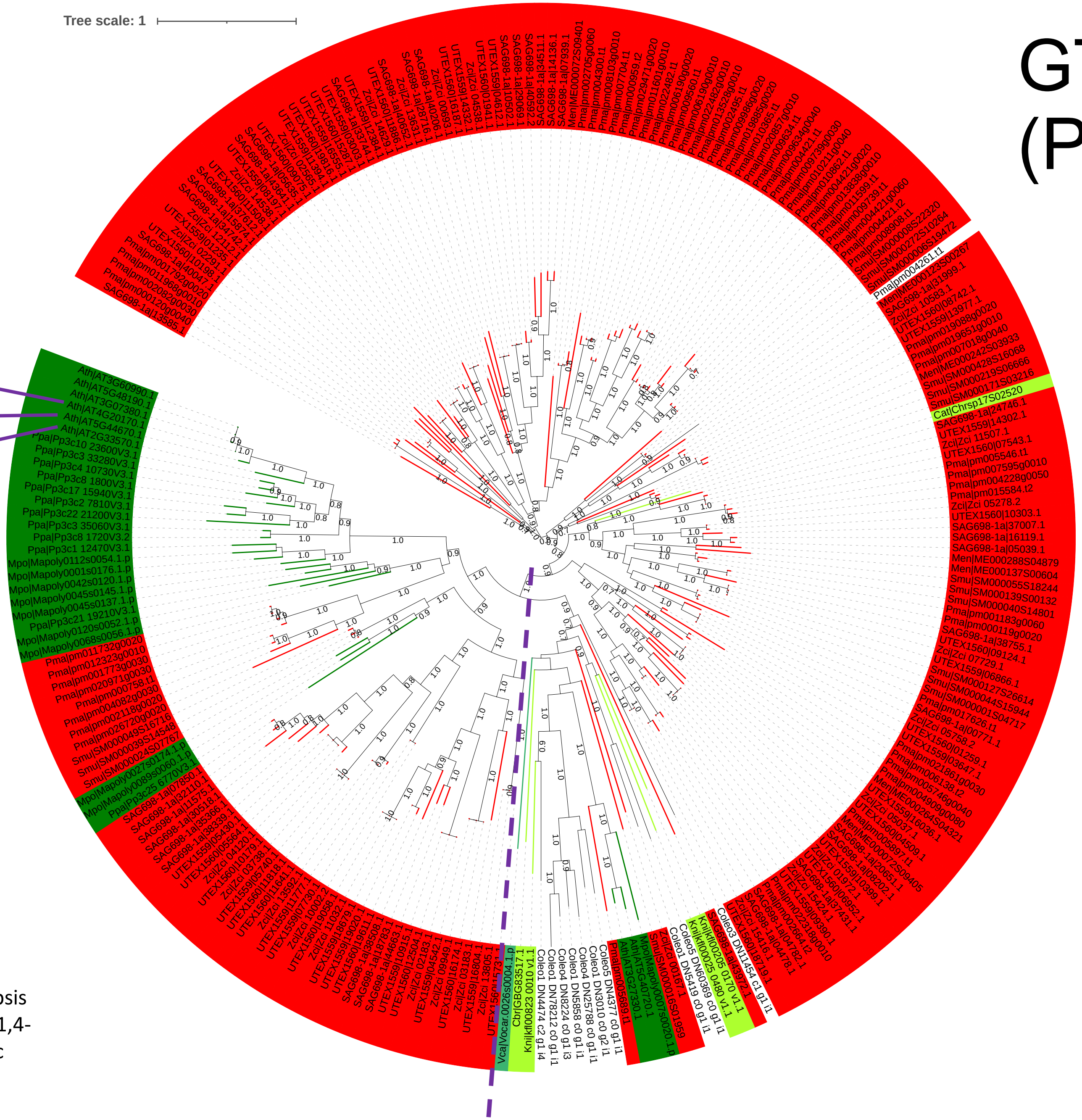

Gene number:
SAG 698-1b: 9
SAG 698-1a: 9
UTEX1559: 9
UTEX1560: 9

Liwanag et al. Pectin biosynthesis: GALS1 in Arabidopsis thaliana is a β-1,4-galactan β-1,4-galactosyltransferase. Plant Cell. 2012 Dec;24(12):5024-36. doi: 10.1105/tpc.112.106625.

Ebert et al. The Three Members of the Arabidopsis Glycosyltransferase Family 92 are Functional β-1,4-Galactan Synthases. Plant Cell Physiol. 2018 Dec 1;59(12):2624-2636. doi: 10.1093/pcp/pcy180.

Data S1-76

https://itol.embl.de/tree/208127240222183691667018617

Polysaccharide Lyase Family 4 / Subf 2
rhamnogalacturonan endolyase (EC 4.2.2.23)

Mokshina N, Makshakova O, Nazipova A, Gorshkov O, Gorshkova T. Flax rhamnogalacturonan lyases: phylogeny, differential expression and modeling of protein structure. Physiol Plant. 2019 Oct;167(2):173-187. doi: 10.1111/ppl.12880.

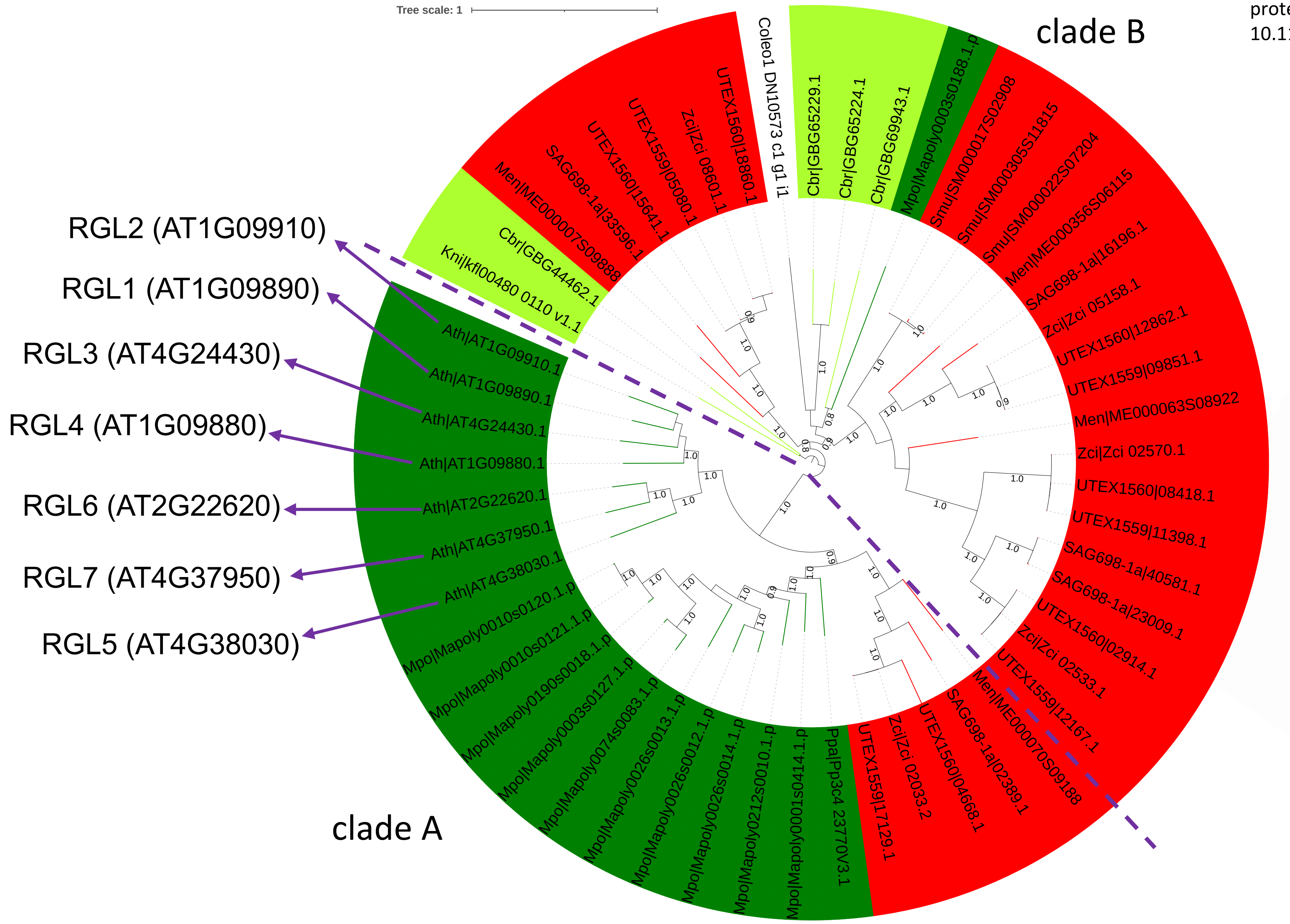

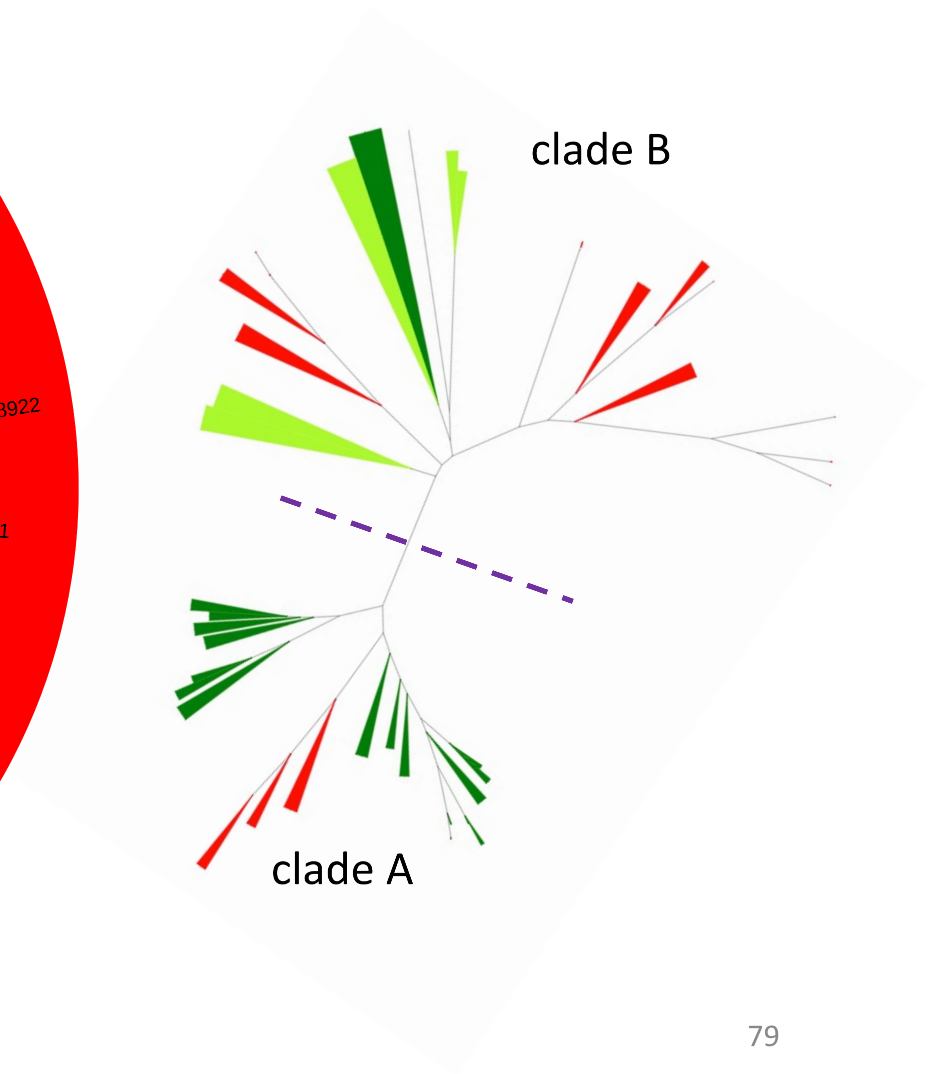

Data S1-77

https://itol.embl.de/tree/208127240222360251667019913

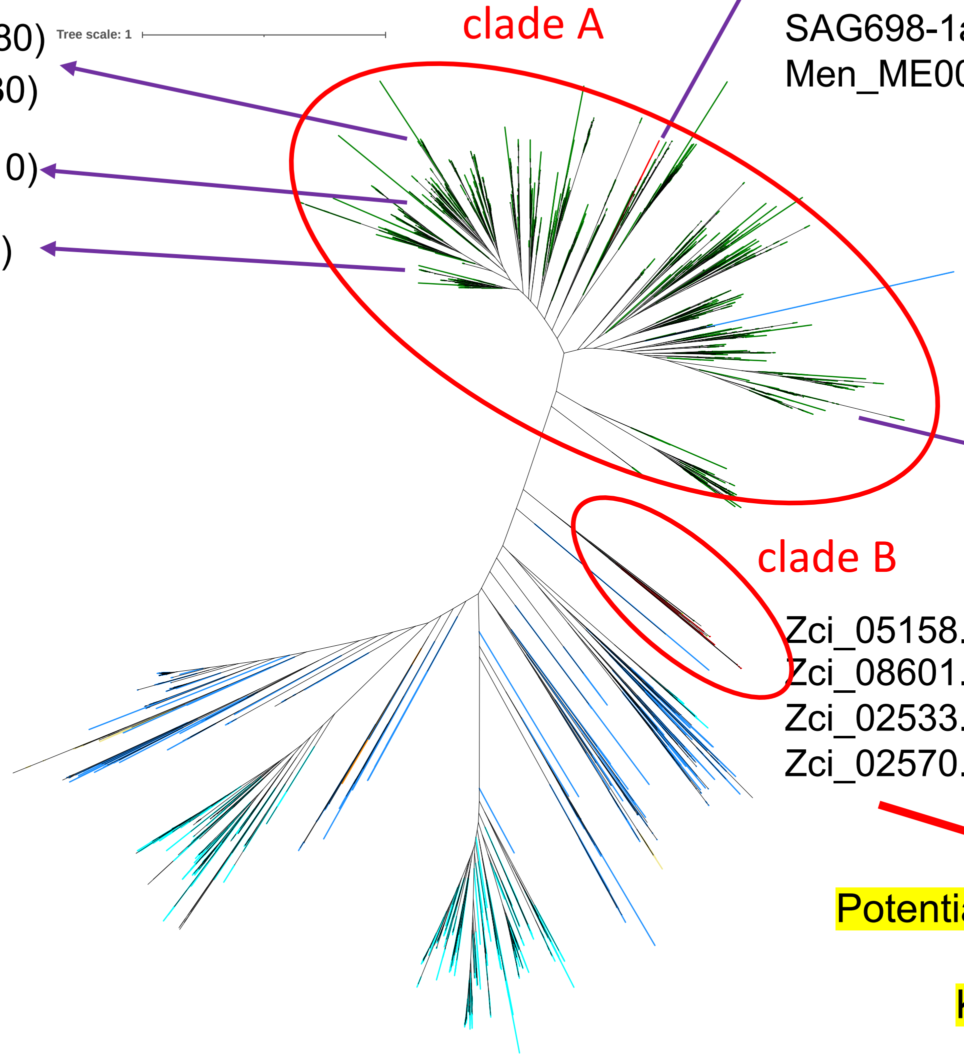

RGLs:

- clade B is the older clade
- clade A is younger, appearing first in a common ancestor of Zygnematophyceae and land plants

Data S1-78

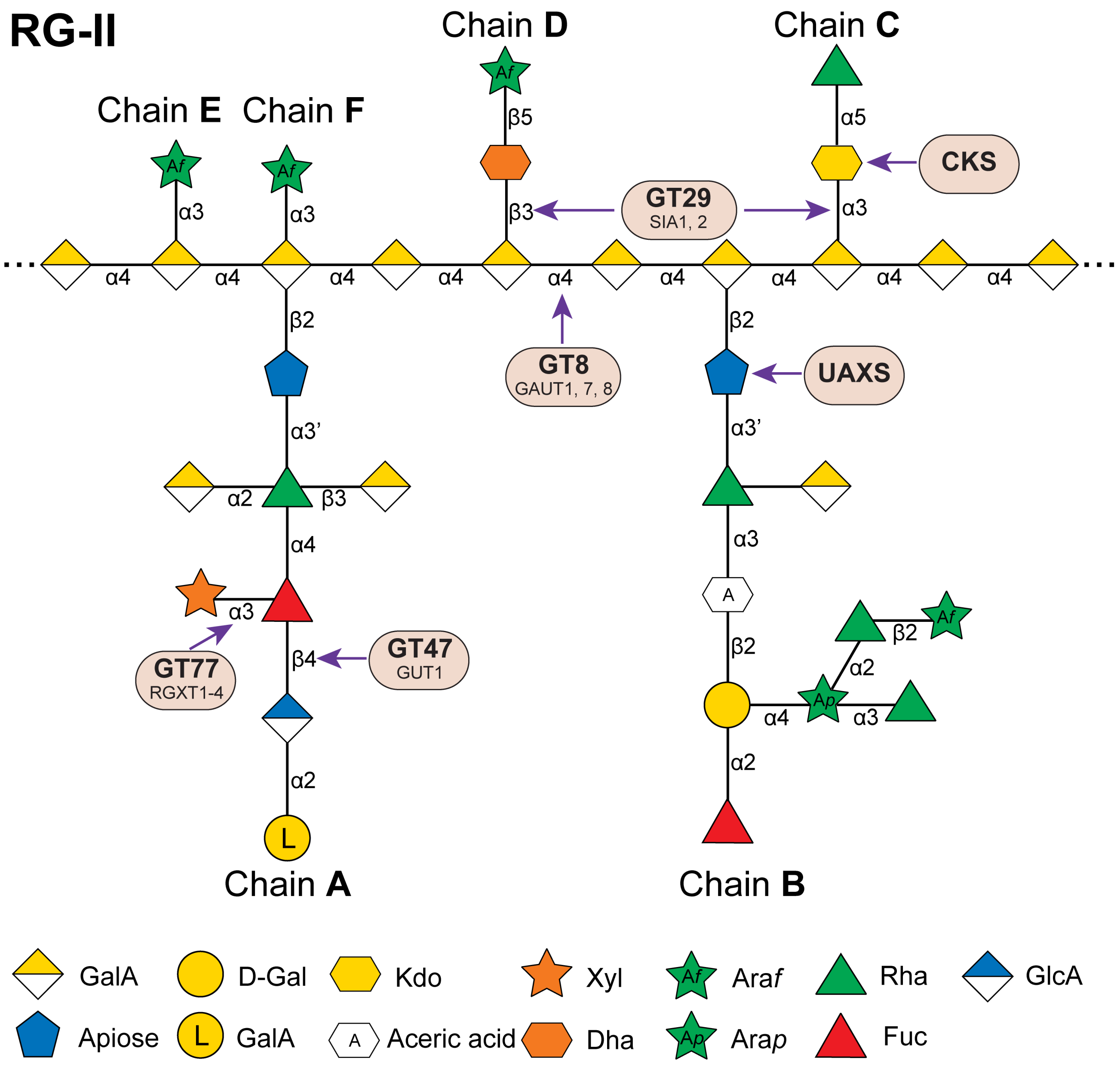

Rhamnogalacturonan II (RG-II)

- RHAMNOGALACTURONAN XYLOSYL- TRANSFERASES (RGXTs): RG- II:α-1,3-xylosyltransferase activity

Data S1-79

https://itol.embl.de/tree/134238164171236071665177614

GT77 + Coleochaete

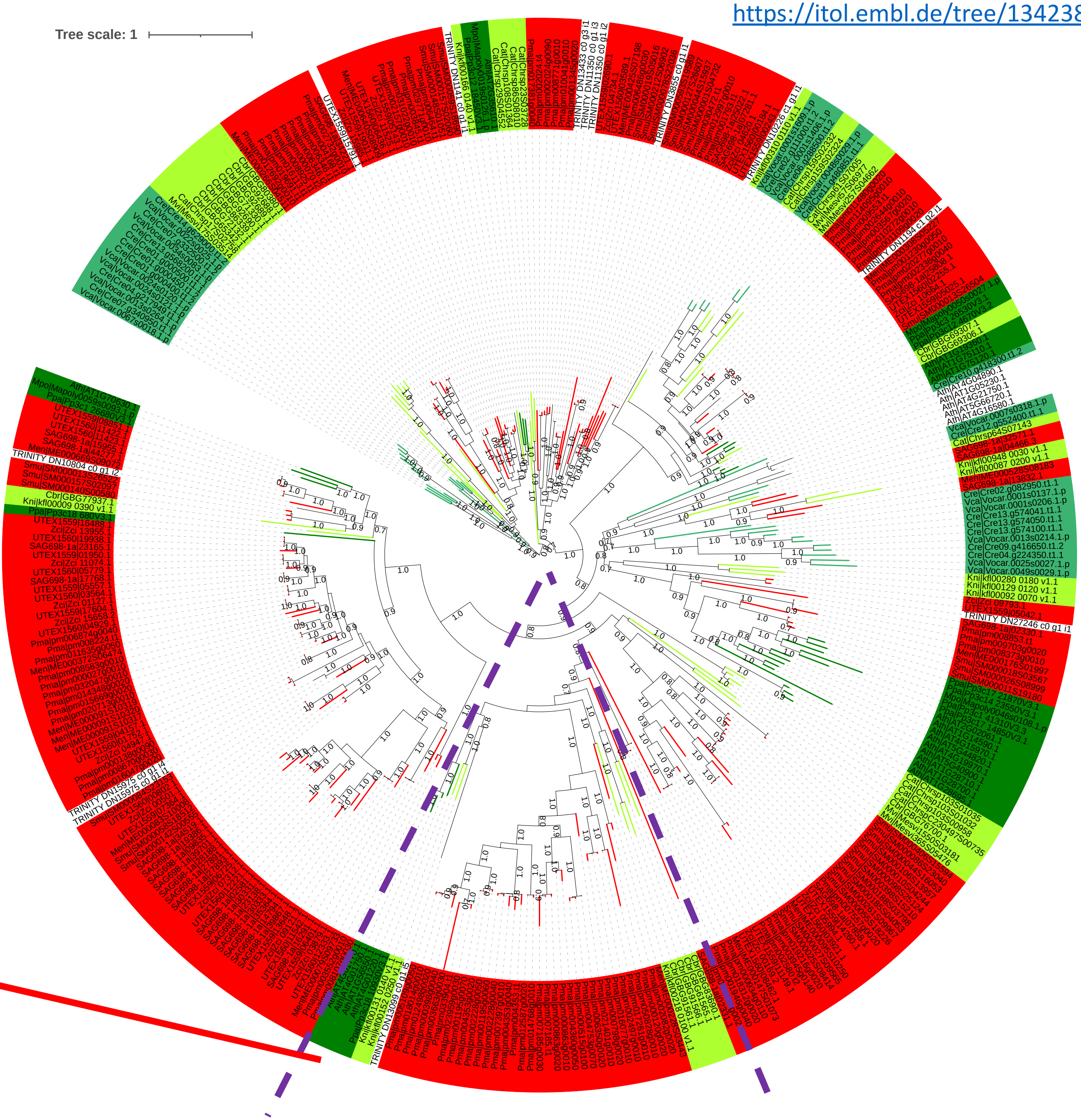

α-1,3-xylosyltransferases

pectic rhamnogalacturonan-II

Egelund et al. Arabidopsis thaliana RGXT1 and RGXT2 encode Golgi-localized (1,3)-alpha-D-xylosyltransferases involved in the synthesis of pectic rhamnogalacturonan-II. Plant Cell. 2006 Oct;18(10):2593-607. doi: 10.1105/tpc.105.036566.

RGXT3 (At1g56550)

RGXT4/MGP4 (At4g01220)

RGXT2 (At4g01750)

RGXT1 (At4g01770)

Data S1-80

Dumont M, Lehner A, Bouton S, Kiefer-Meyer MC, Voxeur A, Pelloux J, Lerouge P, Mollet JC. The cell wall pectic polymer rhamnogalacturonan-II is required for proper pollen tube elongation: implications of a putative sialyltransferase-like protein. Ann Bot. 2014 Oct;114(6):1177-88. doi: 10.1093/aob/mcu093.

GT29 + Coleochaete

https://itol.embl.de/tree/208127832353348311665116092

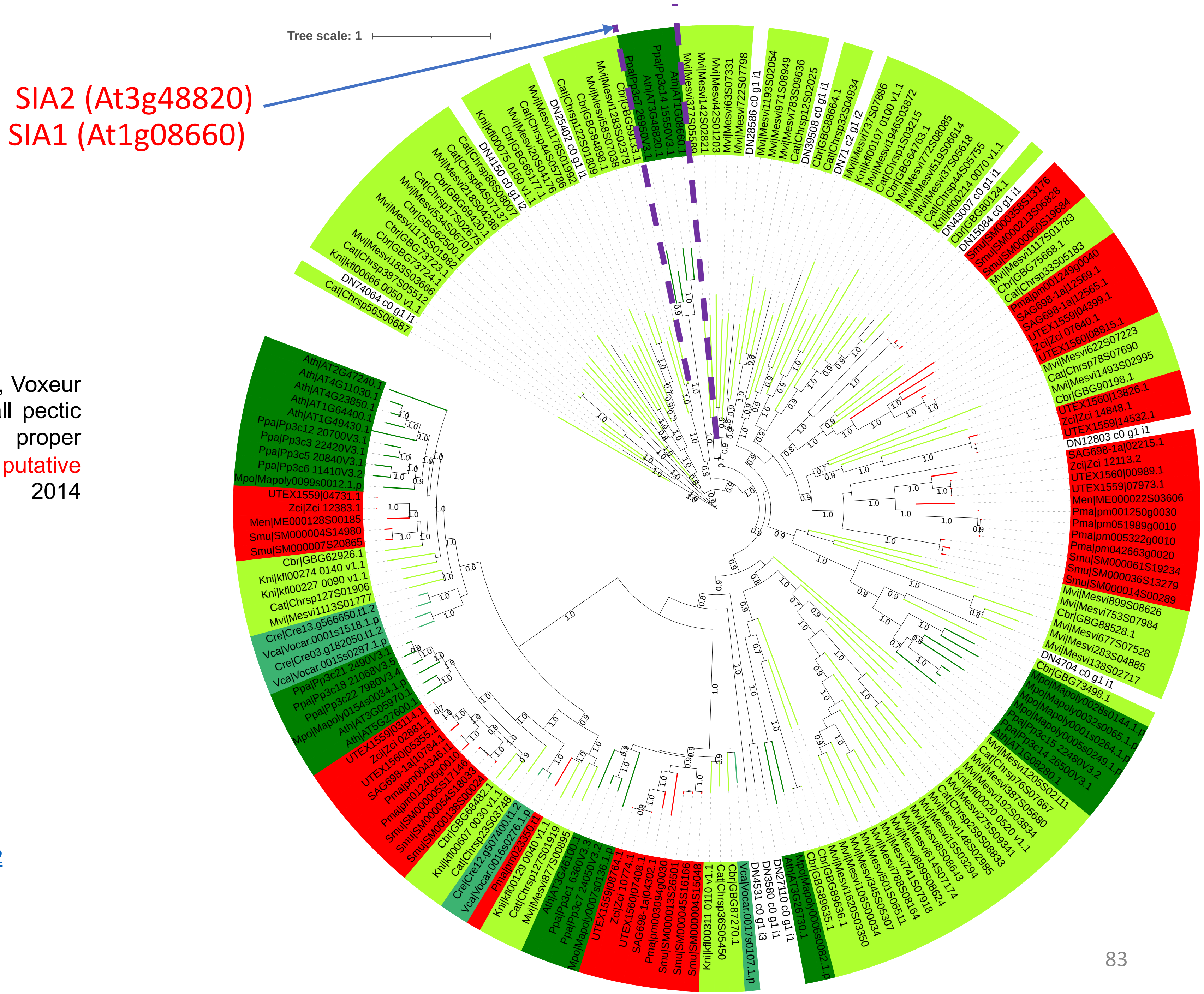

Data S1-81

GH28

PGF8 (Pectin lyase-like superfamily protein, AT3G57790)

AT1G19170(PGF1)
AT3G42950(PGF6)

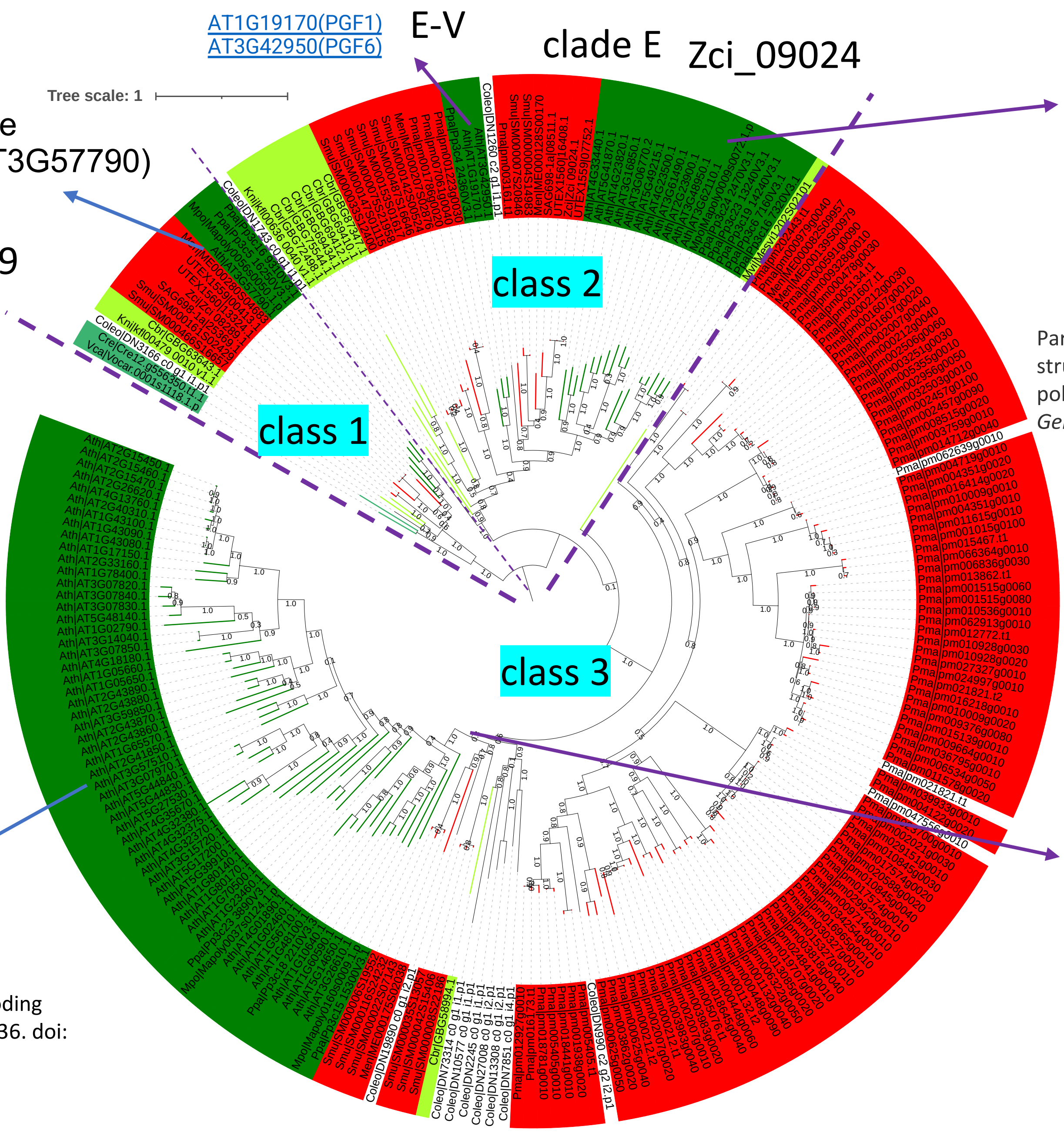

GH28.hmm (dbcan) from 16 genomes and Coleochaete with e-value < 1e-10

Rodríguez-Gacio Mdel C, Nicolás C, Matilla AJ. Cloning and analysis of a cDNA encoding an endo-polygalacturonase expressed during the desiccation period of the silique-valves of turnip-tops (Brassica rapa L. cv. Rapa). J Plant Physiol. 2004 Feb;161(2):219-27. doi: 10.1078/0176-1617-01153.

Gallego-Giraldo, L., Liu, C., Pose-Albacete, S., Pattathil, S., Peralta, A.G., Young, J. et al. (2020) ARABIDOPSIS DEHISCENCE ZONE POLYGALACTURONASE1 (ADPG1) releases latent defense signals in stems with reduced lignin content. Proceedings of the National Academy of Sciences of the United States of America, 117, 3281–3290

ADPG1 (At3g57510)

Torki, M., Mandaron, P., Mache, R., and Falconet, D. (2000). Characterization of a ubiquitous expressed gene family encoding polygalacturonase in Arabidopsis thaliana. Gene 242, 427–436. doi: 10.1016/S0378-1119(99)00497-7

AT4G33440(PGF14) AT5G41870(PGF15)
AT4G23820(PGF13)
AT3G16850(PGF5)
AT3G06770(PGF4)
AT5G49215(PGF16)
AT4G23500(PGF12)
AT3G61490(PGF9)
AT2G23900(PGF3)
AT3G48950(PGF7)
AT3G62110(PGF10)

PMID: 30154820

Park, KC., Kwon, SJ. & Kim, NS. Intron loss mediated structural dynamics and functional differentiation of the polygalacturonase gene family in land plants. *Genes Genom* **32**, 570–577 (2010).

67 Ath IDs

AT4G01890
AT1G02460
AT1G56710(PGL1)
AT1G48100(PGX3)
AT1G10640
AT1G60590
AT5G14650(PGLR)
AT3G26610(PGX1)
AT1G23460
AT1G70500
AT1G80170
AT1G80140
AT5G39910
AT5G17200
AT3G15720
AT4G32370

https://itol.embl.de/tree/1342381641712832116730287 15

Data S1-82a

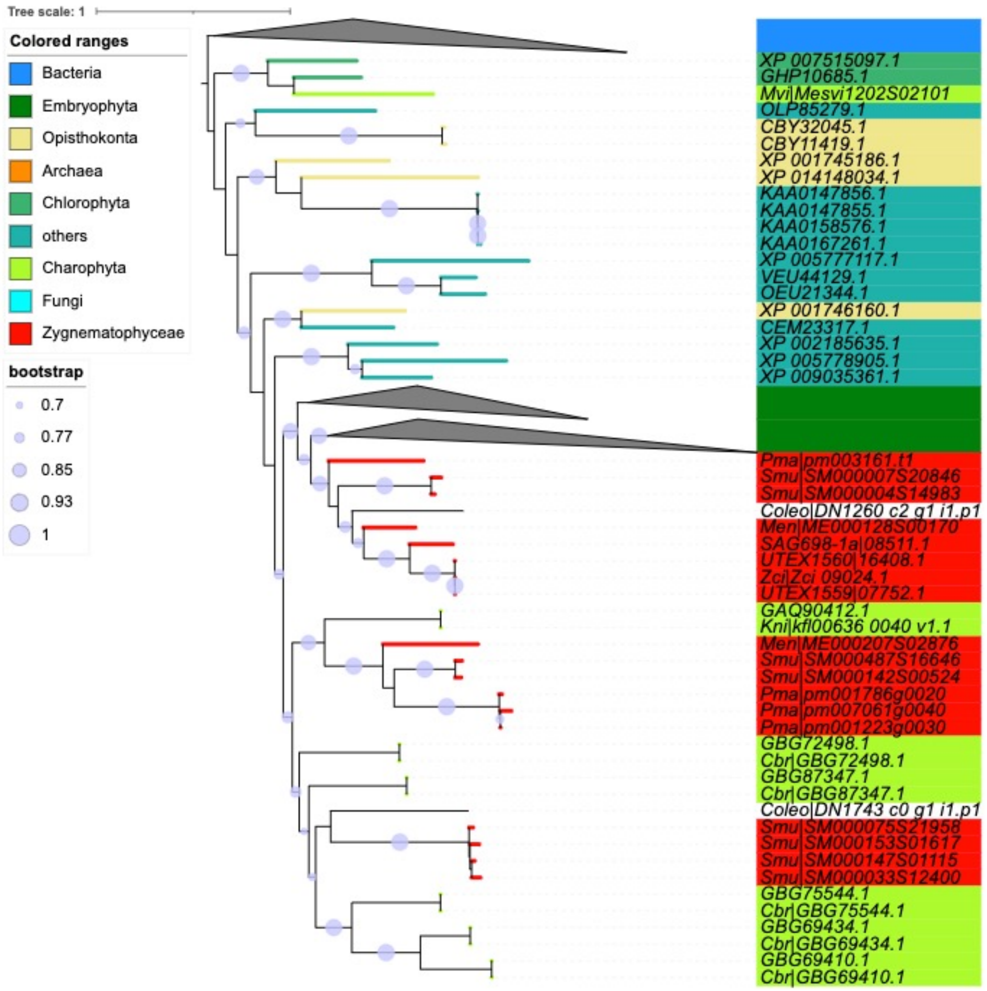

class 2

Zci_09024

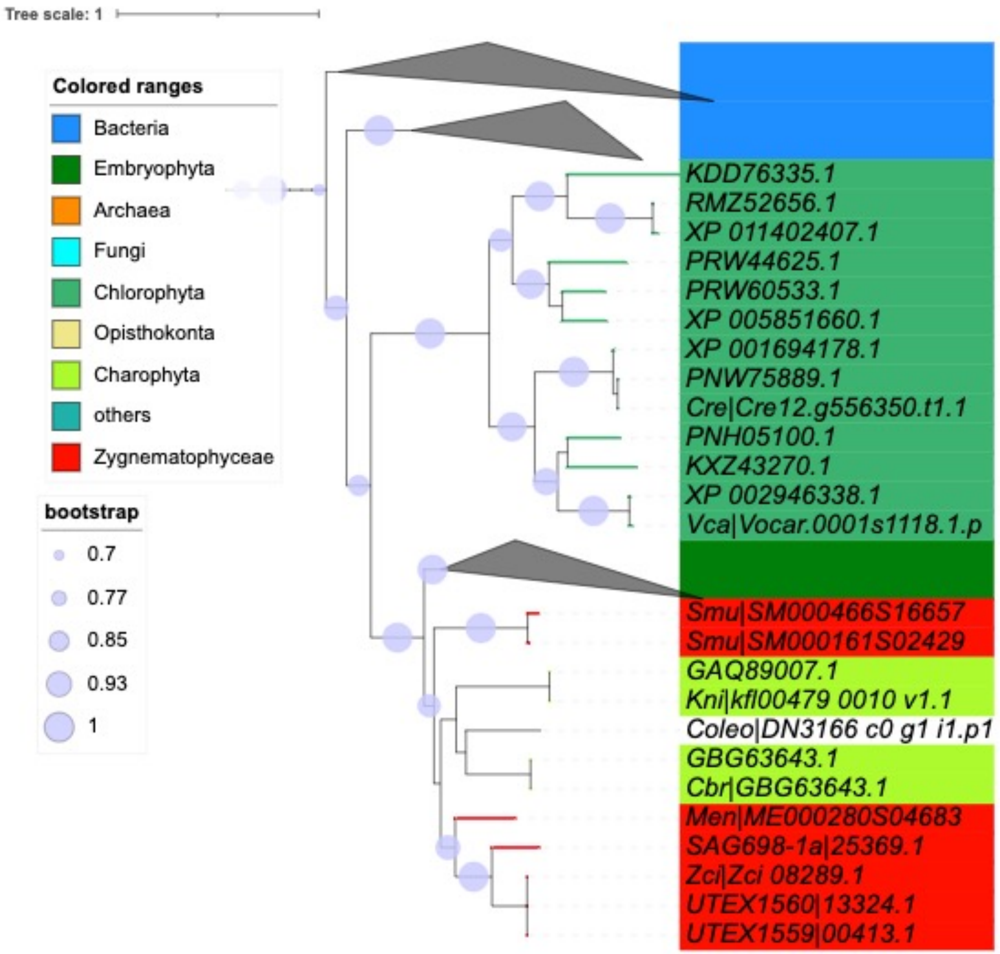

class 1

Zci_08289

Data S1-82b

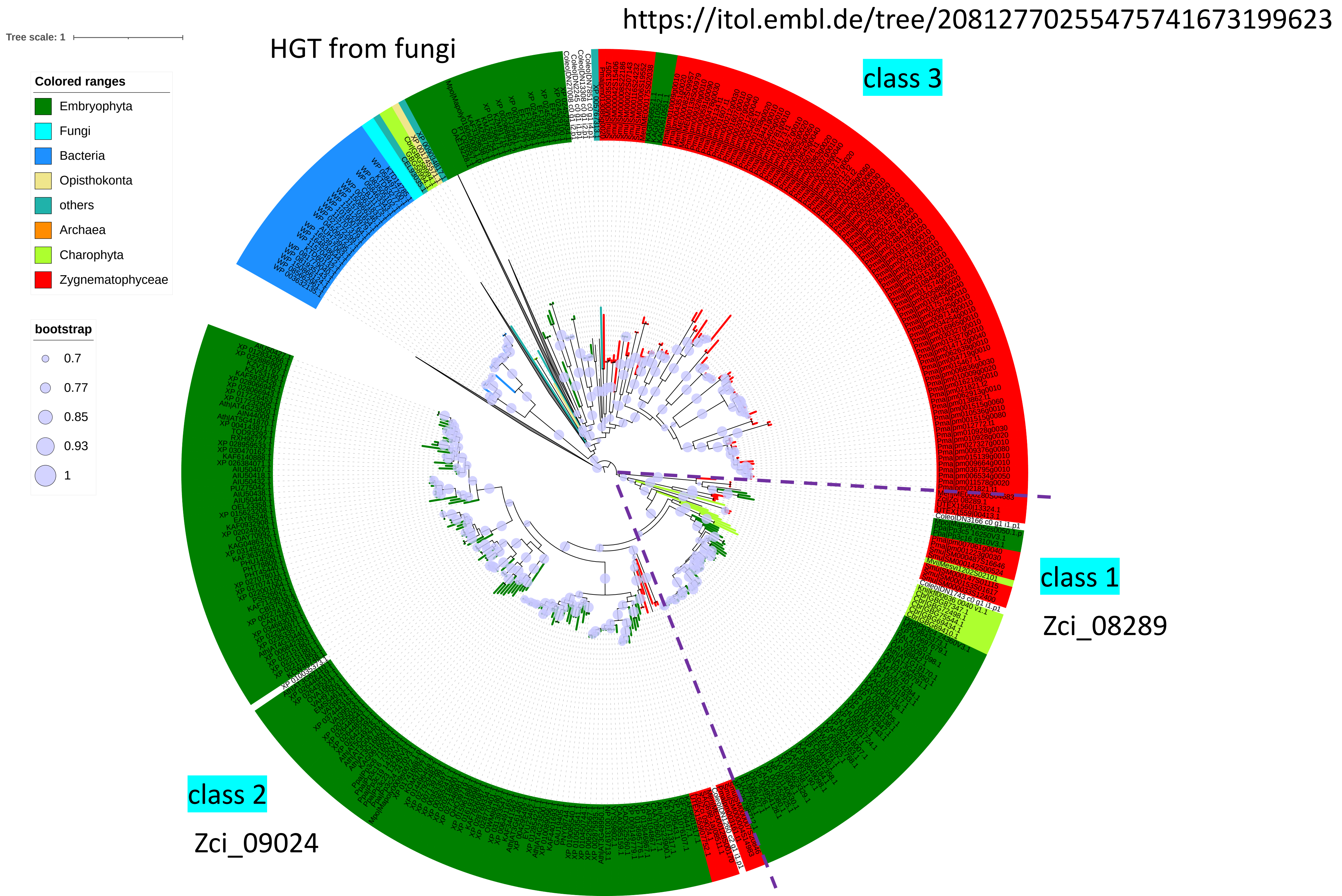
https://itol.embl.de/tree/2081277025547574167319962 3
HGT from fungi
Tree scale: 1
Colored ranges
Embryophyta
Fungi
Bacteria
Opisthokonta
others
Archaea
Charophyta
Zygnematophyceae
bootstrap
0.7
0.77
0.85
0.93
1
class 3
class 1
Zci_08289
class 2
Zci_09024

Data S1-83a

http://www.cazy.org/e1.html

http://www.cazy.org/PL1_12_characterized.html

At1g04680	PL1	AAB80622.1
At1g11920	PL1	AAC17625.1
At1g14420	PL1	AAF43942.1
At1g30350	PL1	AAG51103.1
At1g67750	PL1	AAL58893.1
At2g02720	PL1	AAC05350.1
At3g01270	PL1	AAF03499.1
At3g07010	PL1	AAF27005.1
At3g09540	PL1_12	AAF23285.1
At3g55140	PL1_12	CAB75748.1
At3g24230	PL1	BAB01365.1
At3g24670	PL1	BAB01216.1
At3g27400	PL1	NP_189376.1
At3g53190	PL1	BAD95042.1
At3g54920 (PMR6)	PL1	AAM97687.1
At4g13210	PL1	CAB41931.1
At4g13710	PL1	AAL11586.1
At4g22080	PL1	CAA18111.1
At4g22090	PL1	CAA18112.1
At4g24780 (A10)	PL1	AAK25850.1
At5g04310	PL1	NP_196051.2
At5g09280 (fragment)	PL1	CAC05454.1
At5g15110	PL1	CAC01830.1
At5g48900	PL1	AAK92730.1
At5g55720	PL1	BAB09239.1
At5g63180	PL1	AAL25610.1

Data S1-83b

PL1

https://itol.embl.de/tree/2081278868212161668015296

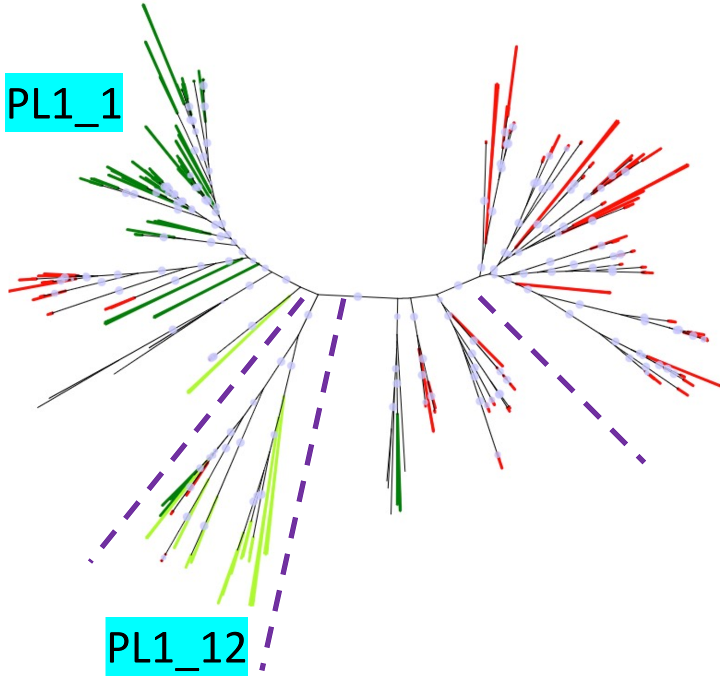

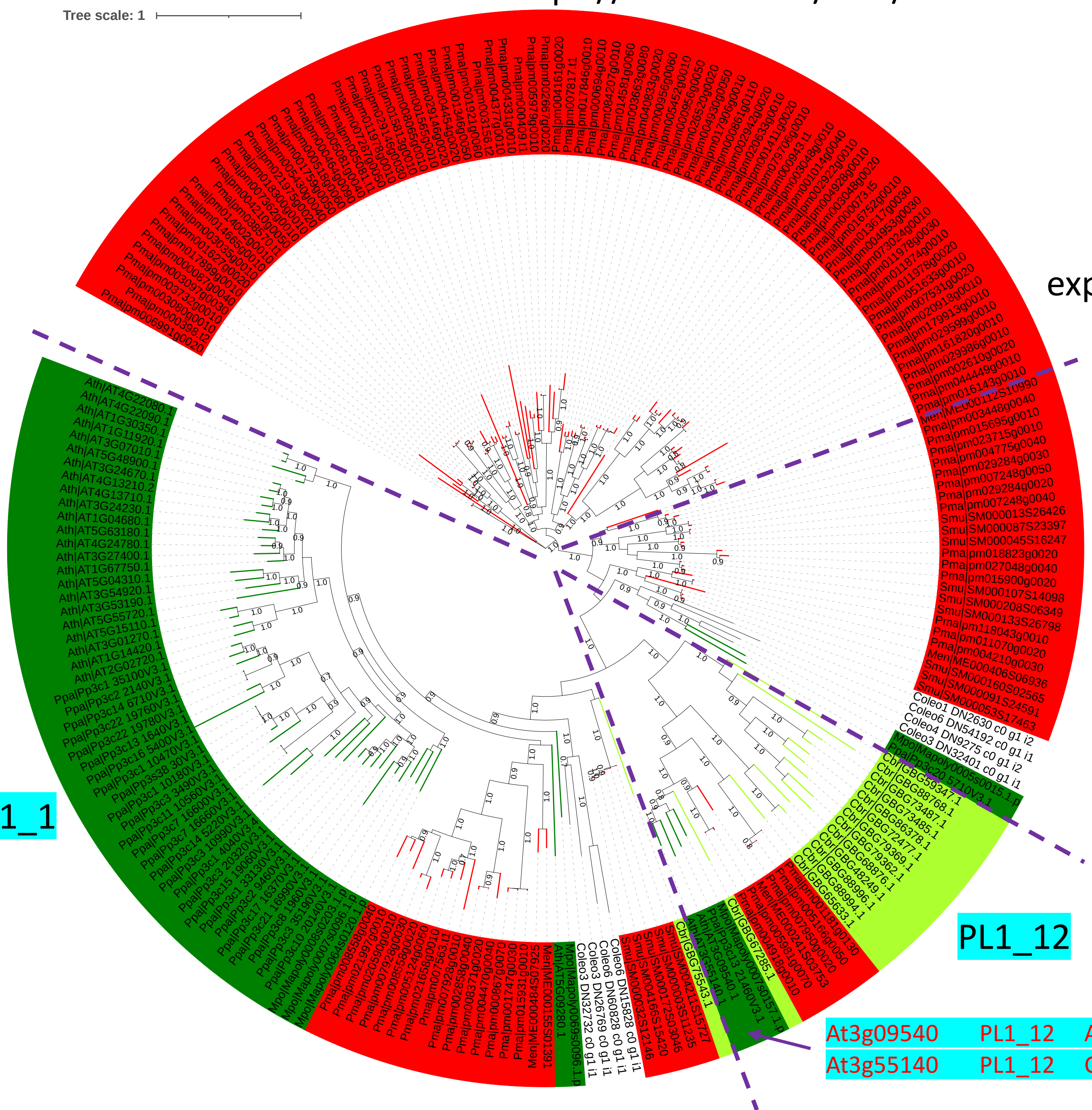

At3g09540	PL1_12	AAF23285.1
At3g55140	PL1_12	CAB75748.1

Data S1-84a

https://itol.embl.de/tree/2081278868154851668014154

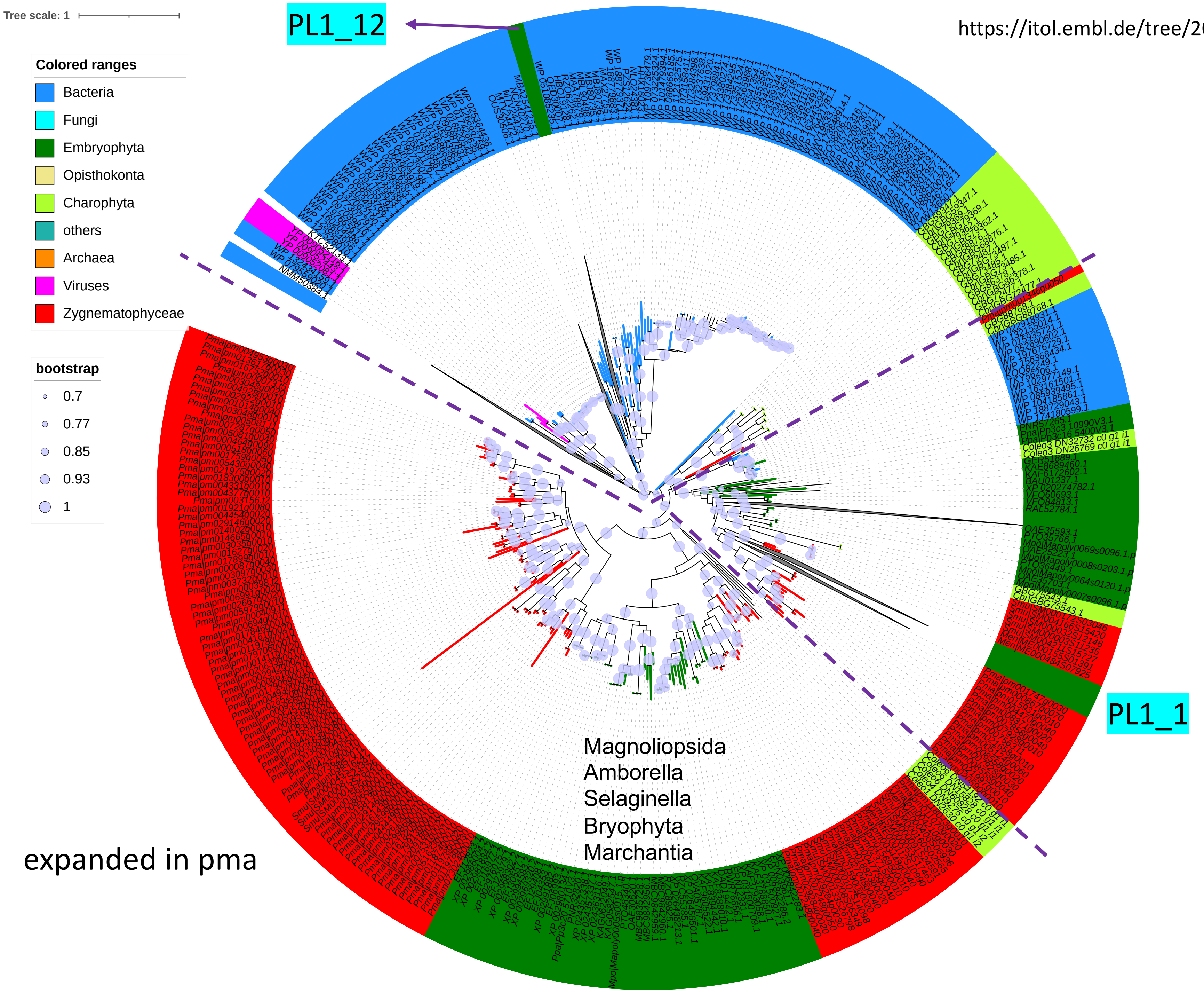

Data S1-84b

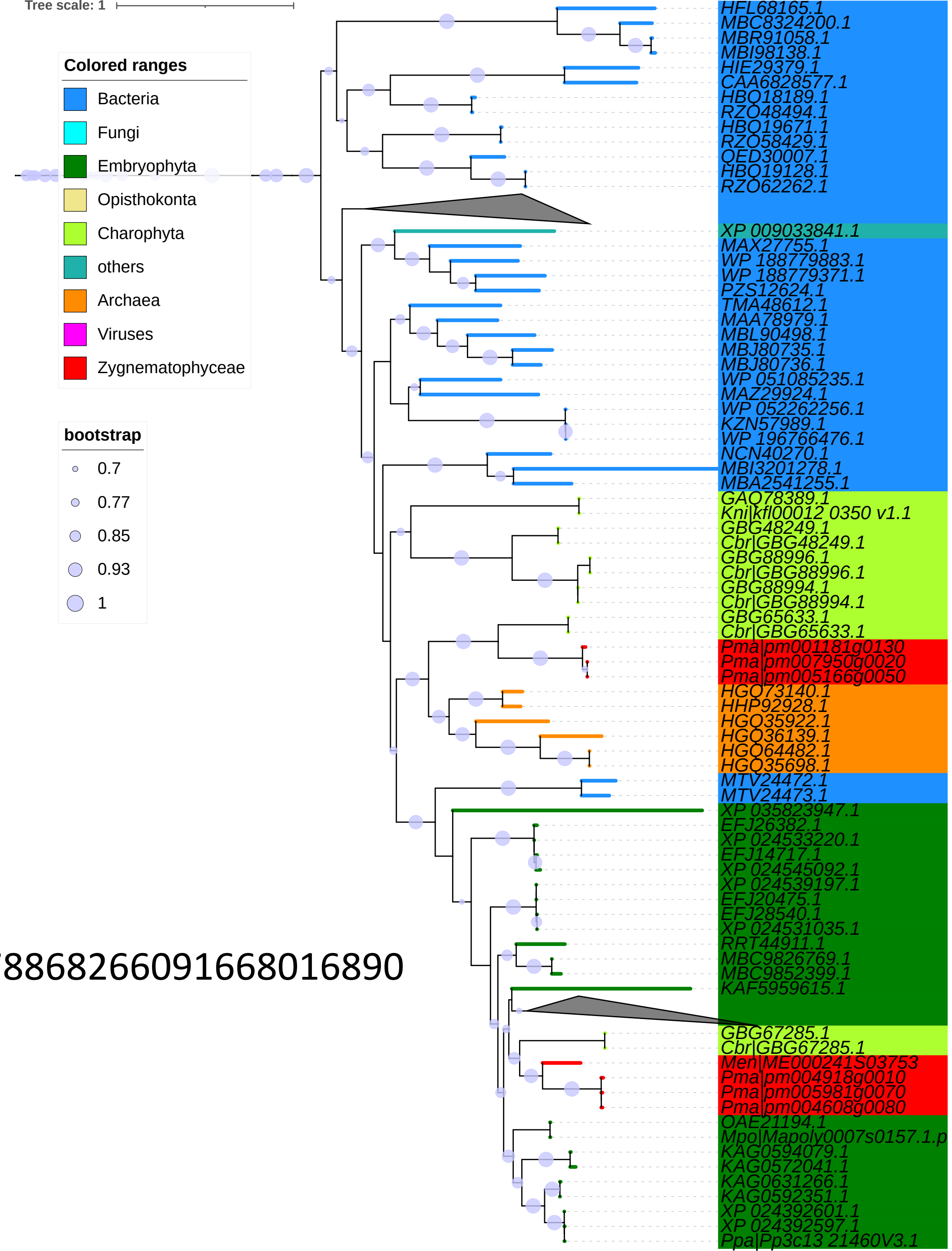

https://itol.embl.de/tree/2081278868266091668016890

At3g09540	PL1_12	AAF23285.1
At3g55140	PL1_12	CAB75748.1
